# Supplementary material for: Intercontinental Gut Microbiome Variances in IBD
Source: Int J Mol Sci. 2022 Sep 17;23(18):10868. doi: 10.3390/ijms231810868 (PMC9506019; doi:10.3390/ijms231810868)
Supplement: Supplementary file 1 [file ijms-23-10868-s001.zip › ijms-1914741-supplementary/supplementary_tableS1.pdf]

SampleID	country	gender	Host_Age	study	disease	BMI	hbi	cai	rutgeerts_score	cd_localization	cd_behaviour	uc_extent	dysbiosis_index	jejunum	ileum	cecum	colon	timepoint	patientID
ERR1620255	China	Male	48	Chinese	CD	22.3							-0.42	No	Yes	No	Yes	0	ERR1620255
ERR1620256	China	Male	22	Chinese	CD	18.2							0.04	No	Yes	-	No	0	ERR1620256
ERR1620257	China	Female	27	Chinese	CD	16.2							-0.68	No	Yes	-	Yes	0	ERR1620257
ERR1620258	China	Male	16	Chinese	CD	16.4							-0.92	No	Yes	-	Yes	0	ERR1620258
ERR1620259	China	Female	22	Chinese	CD	13.2							3.35	No	Yes	-	No	0	ERR1620259
ERR1620260	China	Female	39	Chinese	CD	17.6							0.16	No	Yes	-	Yes	0	ERR1620260
ERR1620261	China	Male	17	Chinese	CD	19.7							Inf	No	Yes	-	Yes	0	ERR1620261
ERR1620262	China	Female	43	Chinese	CD	14.6							1.4	-	-	-	-	0	ERR1620262
ERR1620263	China	Male	27	Chinese	CD	18.5							Inf	No	Yes	-	Yes	0	ERR1620263
ERR1620264	China	Male	27	Chinese	CD	16							-0.19	No	Yes	-	Yes	0	ERR1620264
ERR1620265	China	Male	22	Chinese	CD	18.4							0.2	Yes	Yes	Yes	Yes	0	ERR1620265
ERR1620266	China	Female	42	Chinese	CD	24.9							0.67	No	No	-	Yes	0	ERR1620266
ERR1620267	China	Male	29	Chinese	CD	16.5							0.72	No	Yes	-	Yes	0	ERR1620267
ERR1620268	China	Male	21	Chinese	CD	19.6							-0.48	-	-	-	-	0	ERR1620268
ERR1620269	China	Male	21	Chinese	CD	19.6							0.22	-	-	-	-	0	ERR1620269
ERR1620270	China	Male	31	Chinese	CD	17.9							-0.5	No	Yes	-	Yes	0	ERR1620270
ERR1620271	China	Female	28	Chinese	CD	16.8							0.73	No	Yes	-	Yes	0	ERR1620271
ERR1620272	China	Male	33	Chinese	CD	23.2							0.96	No	Yes	-	Yes	0	ERR1620272
ERR1620273	China	Male	33	Chinese	CD	22.5							Inf	-	-	-	-	0	ERR1620273
ERR1620274	China	Male	29	Chinese	CD	20.9							0.45	No	Yes	-	Yes	0	ERR1620274
ERR1620275	China	Male	29	Chinese	CD	15.4							Inf	No	Yes	-	Yes	0	ERR1620275
ERR1620276	China	Male	23	Chinese	CD	22.9							0.06	No	Yes	-	No	0	ERR1620276
ERR1620278	China	Female	44	Chinese	CD	20.9							Inf	-	-	-	-	0	ERR1620278
ERR1620279	China	Female	44	Chinese	CD	20.9							1.24	No	Yes	-	Yes	0	ERR1620279
ERR1620280	China	Male	36	Chinese	CD	17.5							-0.65	Yes	Yes	-	No	0	ERR1620280
ERR1620281	China	Male	36	Chinese	CD	17.5							0.28	-	-	-	-	0	ERR1620281
ERR1620282	China	Male	31	Chinese	CD	22							-1.26	No	Yes	-	Yes	0	ERR1620282

ERR1620283	China	Male	25	Chinese	CD	19	-0.01	No	Yes	-	Yes	0	ERR1620283
ERR1620284	China	Male	25	Chinese	CD	18.7	-0.4	-	-	-	-	0	ERR1620284
ERR1620285	China	Male	23	Chinese	CD	22.5	-0.1	No	Yes	-	Yes	0	ERR1620285
ERR1620286	China	Female	40	Chinese	CD	21.8	-0.01	No	Yes	-	Yes	0	ERR1620286
ERR1620287	China	Female	40	Chinese	CD	21.9	-0.56	-	-	-	-	0	ERR1620287
ERR1620288	China	Male	30	Chinese	CD	19.5	-0.33	No	Yes	-	No	0	ERR1620288
ERR1620289	China	Female	39	Chinese	CD	20	0.48	No	No	Yes	No	0	ERR1620289
ERR1620290	China	Male	27	Chinese	CD	19.4	1.21	No	Yes	-	Yes	0	ERR1620290
ERR1620291	China	Male	30	Chinese	CD	22	0.25	No	No	Yes	Yes	0	ERR1620291
ERR1620292	China	Male	35	Chinese	CD	20.6	1.39	No	Yes	-	Yes	0	ERR1620292
ERR1620293	China	Male	28	Chinese	CD	18	0.29	No	Yes	-	Yes	0	ERR1620293
ERR1620294	China	Male	24	Chinese	CD	23.3	0.39	No	Yes	-	Yes	0	ERR1620294
ERR1620295	China	Male	25	Chinese	CD	18.8	0.05	No	Yes	-	Yes	0	ERR1620295
ERR1620296	China	Male	18	Chinese	CD	23.7	0.31	No	Yes	-	Yes	0	ERR1620296
ERR1620298	China	Male	18	Chinese	CD	15.3	2.78	No	Yes	-	Yes	0	ERR1620298
ERR1620299	China	Male	18	Chinese	CD	15.3	0.67	-	-	-	-	0	ERR1620299
ERR1620300	China	Male	15	Chinese	CD	18.3	-0.19	No	Yes	Yes	Yes	0	ERR1620300
ERR1620301	China	Male	40	Chinese	CD	18.3	1.66	No	Yes	-	Yes	0	ERR1620301
ERR1620302	China	Male	35	Chinese	CD	24.4	-0.75	No	No	-	Yes	0	ERR1620302
ERR1620303	China	Male	42	Chinese	CD	17	0.52	No	Yes	-	No	0	ERR1620303
ERR1620304	China	Male	25	Chinese	CD	14	1.95	No	Yes	-	Yes	0	ERR1620304
ERR1620305	China	Male	25	Chinese	CD	14	Inf	-	-	-	-	0	ERR1620305
ERR1620306	China	Male	23	Chinese	CD	16.7	0.82	No	Yes	-	Yes	0	ERR1620306
ERR1620307	China	Male	23	Chinese	CD	16.7	0.36	-	-	-	-	0	ERR1620307
ERR1620308	China	Male	24	Chinese	CD	16.5	-0.8	No	No	Yes	No	0	ERR1620308
ERR1620309	China	Male	24	Chinese	CD	16.5	-0.25	-	-	-	-	0	ERR1620309
ERR1620310	China	Male	16	Chinese	CD	17.2	NA	NA	NA	NA	NA	0	ERR1620310
ERR1620311	China	Male	5	Chinese	CD	not recorded	NA	NA	NA	NA	NA	0	ERR1620311

ERR1620313	China	Female	23	Chinese	CD	15.8	-0.16	-	-	-	-	0	ERR1620313
ERR1620314	China	Female	23	Chinese	CD	15.8	1.48	Yes	Yes	-	No	0	ERR1620314
ERR1620315	China	Female	36	Chinese	CD	18.3	0.15	No	Yes	-	Yes	0	ERR1620315
ERR1620316	China	Male	22	Chinese	CD	19.4	0.14	-	-	-	-	0	ERR1620316
ERR1620317	China	Male	22	Chinese	CD	19.4	-0.56	-	-	-	-	0	ERR1620317
ERR1620318	China	Male	31	Chinese	CD	21	-0.65	No	Yes	-	Yes	0	ERR1620318
ERR1620319	China	Male	31	Chinese	CD	21	-0.52	-	-	-	-	0	ERR1620319
ERR1620320	China	Female	21	Chinese	CD	15.3	1.17	-	-	-	-	0	ERR1620320
ERR1620321	China	Female	21	Chinese	CD	15.3	-0.13	-	-	-	-	0	ERR1620321
ERR1620322	China	Male	26	Chinese	Healthy	21.3	-1.24	-	-	-	-	0	ERR1620322
ERR1620323	China	Male	44	Chinese	Healthy	25.4	-1.44	-	-	-	-	0	ERR1620323
ERR1620324	China	Male	24	Chinese	Healthy	23.7	-0.91	-	-	-	-	0	ERR1620324
ERR1620325	China	Male	24	Chinese	Healthy	22	-1.97	-	-	-	-	0	ERR1620325
ERR1620326	China	Female	22	Chinese	Healthy	20.7	-1.2	-	-	-	-	0	ERR1620326
ERR1620327	China	Male	23	Chinese	Healthy	18.7	-1.14	-	-	-	-	0	ERR1620327
ERR1620328	China	Male	22	Chinese	Healthy	22.3	-0.64	-	-	-	-	0	ERR1620328
ERR1620329	China	Male	24	Chinese	Healthy	21.5	-1.27	-	-	-	-	0	ERR1620329
ERR1620330	China	Male	23	Chinese	Healthy	21.3	-1.18	-	-	-	-	0	ERR1620330
ERR1620331	China	Male	24	Chinese	Healthy	20.2	-2.1	-	-	-	-	0	ERR1620331
ERR1620332	China	Male	24	Chinese	Healthy	22.5	-0.98	-	-	-	-	0	ERR1620332
ERR1620333	China	Male	26	Chinese	Healthy	20.5	-1.38	-	-	-	-	0	ERR1620333
ERR1620334	China	Male	14	Chinese	Healthy	21	-1.02	-	-	-	-	0	ERR1620334
ERR1620335	China	Male	13	Chinese	Healthy	17.4	-1.02	-	-	-	-	0	ERR1620335
ERR1620336	China	Male	13	Chinese	Healthy	17.6	-1.36	-	-	-	-	0	ERR1620336
ERR1620337	China	Male	14	Chinese	Healthy	20	-0.88	-	-	-	-	0	ERR1620337
ERR1620338	China	Male	13	Chinese	Healthy	17.1	-1.21	-	-	-	-	0	ERR1620338
ERR1620339	China	Male	14	Chinese	Healthy	18.8	-1.21	-	-	-	-	0	ERR1620339
ERR1620340	China	Male	14	Chinese	Healthy	23.5	-1.41	-	-	-	-	0	ERR1620340

ERR1620341	China	Male	13	Chine se	Healt hy	19.5	-1.18	-	-	-	-	0	ERR1620 341
ERR1620342	China	Male	14	Chine se	Healt hy	18.8	-1.46	-	-	-	-	0	ERR1620 342
ERR1620343	China	Male	16	Chine se	Healt hy	21.1	-1.73	-	-	-	-	0	ERR1620 343
ERR1620344	China	Male	16	Chine se	Healt hy	21	-1.15	-	-	-	-	0	ERR1620 344
ERR1620346	China	Male	16	Chine se	Healt hy	18.8	-1.41	-	-	-	-	0	ERR1620 346
ERR1620347	China	Male	17	Chine se	Healt hy	19	-0.81	-	-	-	-	0	ERR1620 347
ERR1620348	China	Male	16	Chine se	Healt hy	20.8	-1.34	-	-	-	-	0	ERR1620 348
ERR1620349	China	Male	16	Chine se	Healt hy	29.5	-0.77	-	-	-	-	0	ERR1620 349
ERR1620350	China	Male	17	Chine se	Healt hy	21.1	-1.45	-	-	-	-	0	ERR1620 350
ERR1620351	China	Male	16	Chine se	Healt hy	17.8	-1.4	-	-	-	-	0	ERR1620 351
ERR1620352	China	Male	16	Chine se	Healt hy	19.1	-1.09	-	-	-	-	0	ERR1620 352
ERR1620353	China	Male	16	Chine se	Healt hy	20.9	-0.96	-	-	-	-	0	ERR1620 353
ERR1620355	China	Male	17	Chine se	Healt hy	19.8	-1.53	-	-	-	-	0	ERR1620 355
ERR1620356	China	Male	17	Chine se	Healt hy	21	-1.33	-	-	-	-	0	ERR1620 356
ERR1620357	China	Male	18	Chine se	Healt hy	21.3	-1.53	-	-	-	-	0	ERR1620 357
ERR1620358	China	Male	20	Chine se	Healt hy	19.6	-0.89	-	-	-	-	0	ERR1620 358
ERR1620359	China	Male	19	Chine se	Healt hy	24.5	-1.54	-	-	-	-	0	ERR1620 359
ERR1620360	China	Male	18	Chine se	Healt hy	21.4	-1.36	-	-	-	-	0	ERR1620 360
ERR1620361	China	Male	18	Chine se	Healt hy	19.7	-0.98	-	-	-	-	0	ERR1620 361
ERR1620362	China	Male	17	Chine se	Healt hy	22	-1.37	-	-	-	-	0	ERR1620 362
ERR1620363	China	Male	17	Chine se	Healt hy	24.9	-0.51	-	-	-	-	0	ERR1620 363
ERR1620364	China	Male	19	Chine se	Healt hy	19.4	-1.5	-	-	-	-	0	ERR1620 364
ERR1620365	China	Male	40	Chine se	Healt hy	28.4	-1.52	-	-	-	-	0	ERR1620 365
ERR1620366	China	Male	23	Chine se	Healt hy	18.5	-0.59	-	-	-	-	0	ERR1620 366
ERR1620367	China	Male	17	Chine se	Healt hy	27.5	-1.7	-	-	-	-	0	ERR1620 367
ERR1620368	China	Male	17	Chine se	Healt hy	19.1	-1.32	-	-	-	-	0	ERR1620 368
ERR1620369	China	Male	17	Chine se	Healt hy	18	-0.93	-	-	-	-	0	ERR1620 369
ERR1620370	China	Male	19	Chine se	Healt hy	23.9	-1.42	-	-	-	-	0	ERR1620 370

ERR1620371	China	Male	23	Chinese	Healthy	30.9	-1.65	-	-	-	-	0	ERR1620371
ERR1620372	China	Male	25	Chinese	Healthy	19.3	-1.61	-	-	-	-	0	ERR1620372
ERR1620373	China	Female	39	Chinese	Healthy	18.7	-1.03	-	-	-	-	0	ERR1620373
ERR1620374	China	Male	26	Chinese	Healthy	25.4	-1.64	-	-	-	-	0	ERR1620374
ERR1620375	China	Female	51	Chinese	Healthy	26.4	-0.62	-	-	-	-	0	ERR1620375
ERR1620376	China	Male	24	Chinese	Healthy	18.5	-1.01	-	-	-	-	0	ERR1620376
ERR1620377	China	Male	27	Chinese	Healthy	29.4	-1.31	-	-	-	-	0	ERR1620377
V1.UC6.0	Spain	Female	38	MetaHit	HR	23.18						0	6HR
V1.UC7.0	Spain	Female	19	MetaHit	HR	23.05						0	7HR
V1.UC8.0	Spain	Male	22	MetaHit	HR	25.4						0	8HR
V1.UC9.0	Spain	Male	32	MetaHit	HR	30.37						0	9HR
V1.UC16.0	Spain	Male	25	MetaHit	HR	23.88						0	16HR
V1.UC18.0	Spain	Female	63	MetaHit	HR	28.67						0	18HR
V1.UC19.0	Spain	Female	37	MetaHit	HR	21.19						0	19HR
V1.UC24.0	Spain	Female	62	MetaHit	HR	33.33						0	24HR
V1.UC27.0	Spain	Male	66	MetaHit	HR	26.67						0	27HR
V1.UC28.0	Spain	Female	61	MetaHit	HR	24.56						0	28HR
V1.UC29.0	Spain	Male	46	MetaHit	HR	24.38						0	29HR
V1.UC30.0	Spain	Female	41	MetaHit	HR	23.26						0	30HR
V1.UC32.0	Spain	Male	56	MetaHit	HR	25.71						0	32HR
V1.UC33.0	Spain	Female	53	MetaHit	HR	26.53						0	33HR
V1.UC34.0	Spain	Male	31	MetaHit	HR	23.05						0	34HR
V1.UC36.0	Spain	Female	34	MetaHit	HR	20.43						0	36HR
V1.UC37.0	Spain	Male	19	MetaHit	HR	23.99						0	37HR
V1.UC41.0	Spain	Female	54	MetaHit	HR	23.92						0	41HR
V1.UC42.0	Spain	Female	32	MetaHit	HR	19.56						0	42HR
V1.UC43.0	Spain	Female	27	MetaHit	HR	17.71						0	43HR
V1.UC44.0	Spain	Male	20	MetaHit	HR	20.29						0	44HR

V1.UC46.0	Spain	Female	55	Meta Hit	HR	35.03				0	46HR
V1.UC48.0	Spain	Male	26	Meta Hit	HR	20.9				0	48HR
V1.UC57.0	Spain	Female	32	Meta Hit	HR	19.98				0	57HR
V1.UC60.0	Spain	Female	25	Meta Hit	HR	24.17				0	60HR
V1.UC61.0	Spain	Male	33	Meta Hit	HR	27.78				0	61HR
V1.UC62.0	Spain	Female	37	Meta Hit	HR	22.58				0	62HR
V1.UC63.0	Spain	Male	45	Meta Hit	HR	26.3				0	63HR
V1.UC64.0	Spain	Female	41	Meta Hit	HR	25.61				0	64HR
V1.UC1.0	Spain	Female	31	Meta Hit	UC	17.15	0		E2	0	1UC
V1.UC2.0	Spain	Male	62	Meta Hit	UC	27.76	2		E1	0	2UC
V1.UC3.0	Spain	Male	44	Meta Hit	UC	20.57	2		E3	0	3UC
V1.UC4.0	Spain	Female	47	Meta Hit	UC	26.37	1		E3	0	4UC
V1.UC5.0	Spain	Female	30	Meta Hit	UC	20.32	1		E3	0	5UC
V1.UC10.0	Spain	Male	45	Meta Hit	UC	27.31	1		E3	0	10UC
V1.UC11.0	Spain	Female	48	Meta Hit	UC	22.32	0		E3	0	11UC
V1.UC12.0	Spain	Female	41	Meta Hit	UC	19.9	0		E1	0	12UC
V1.UC13.0	Spain	Female	51	Meta Hit	UC	28.51	0		E1	0	13UC
V1.UC14.0	Spain	Female	53	Meta Hit	UC	20.25	0		E2	0	14UC
V1.UC15.0	Spain	Female	25	Meta Hit	UC	22.77	0		E2	0	15UC
V1.UC17.0	Spain	Female	41	Meta Hit	UC	24.46	0		E3	0	17UC
V1.UC21.0	Spain	Male	62	Meta Hit	UC	25.21	0		E3	0	21UC
V1.UC23.0	Spain	Male	43	Meta Hit	UC	28.73	0		E2	0	23UC
V1.UC25.0	Spain	Female	52	Meta Hit	UC	23.44	0		E2	0	25UC
V1.UC26.0	Spain	Female	36	Meta Hit	UC	28.58	1		E2	0	26UC
V1.UC31.0	Spain	Female	26	Meta Hit	UC	20.44	0		E3	0	31UC
V1.UC35.0	Spain	Female	33	Meta Hit	UC	19.43	0		E3	0	35UC
V1.UC38.0	Spain	Male	24	Meta Hit	UC	18.07	0		E3	0	38UC
V1.UC39.0	Spain	Male	26	Meta Hit	UC	22.4	1		E1	0	39UC

V1.UC40.0	Spain	Female	32	Meta Hit	UC	25.89	0	E3	0	40UC
V1.UC45.0	Spain	Female	50	Meta Hit	UC	23.05	1	E2	0	45UC
V1.UC47.0	Spain	Male	46	Meta Hit	UC	25.71	0	E1	0	47UC
V1.UC49.0	Spain	Female	25	Meta Hit	UC	19.83	2	E2	0	49UC
V1.UC51.0	Spain	Female	44	Meta Hit	UC	23.31	0	E1	0	51UC
V1.UC52.0	Spain	Female	43	Meta Hit	UC	29.34	0	E1	0	52UC
V1.UC53.0	Spain	Female	24	Meta Hit	UC	20.24	0	E2	0	53UC
V1.UC54.0	Spain	Male	32	Meta Hit	UC	35.62	0	E3	0	54UC
V1.UC55.0	Spain	Female	46	Meta Hit	UC	18.82	0	E1	0	55UC
V1.UC56.0	Spain	Female	35	Meta Hit	UC	18.83	0	E3	0	56UC
V1.UC58.0	Spain	Female	41	Meta Hit	UC	28.35	0	E1	0	58UC
V1.UC59.0	Spain	Female	46	Meta Hit	UC	23.44	1	E2	0	59UC
V1.UC50.0	Spain	Female	45	Meta Hit	UC	20.71	0	E2	0	50UC
V1.UC2.4	Spain	Male	62	Meta Hit	UC	30.02	8		4	2UC
V1.UC3.2	Spain	Male	44	Meta Hit	UC	19.27	13		2	3UC
V1.UC4.5	Spain	Female	47	Meta Hit	UC	26.37	0		5	4UC
V1.UC5.3	Spain	Female	30	Meta Hit	UC	20.7	0		3	5UC
V1.UC10.2	Spain	Male	45	Meta Hit	UC	27.31	7		2	10UC
V1.UC11.5	Spain	Female	48	Meta Hit	UC	23.03	2		5	11UC
V1.UC12.4	Spain	Female	41	Meta Hit	UC	19.79	6		4	12UC
V1.UC13.3	Spain	Female	51	Meta Hit	UC	28.95	7		3	13UC
V1.UC14.1	Spain	Female	53	Meta Hit	UC	20.06	7		1	14UC
V1.UC15.3	Spain	Female	25	Meta Hit	UC	23.19	6		3	15UC
V1.UC17.2	Spain	Female	41	Meta Hit	UC	24.84	9		2	17UC
V1.UC21.4	Spain	Male	62	Meta Hit	UC	26.26	1		4	21UC
V1.UC23.1	Spain	Male	43	Meta Hit	UC	26.49	8		1	23UC
V1.UC25.1	Spain	Female	52	Meta Hit	UC	23.05	7		1	25UC
V1.UC26.4	Spain	Female	36	Meta Hit	UC	26.29	8		4	26UC

V1.UC31.4	Spain	Female	26	Meta Hit	UC	22.22	2	4	31UC
V1.UC35.4	Spain	Female	33	Meta Hit	UC	20.43	0	4	35UC
V1.UC38.4	Spain	Male	24	Meta Hit	UC	19.26	0	4	38UC
V1.UC39.4	Spain	Male	26	Meta Hit	UC	21.5	1	4	39UC
V1.UC40.1	Spain	Female	32	Meta Hit	UC	27.12	7	1	40UC
V1.UC47.4	Spain	Male	46	Meta Hit	UC	26.45	0	4	47UC
V1.UC49.1	Spain	Female	25	Meta Hit	UC	20.2	4	1	49UC
V1.UC50.1	Spain	Female	45	Meta Hit	UC	20.71	5	1	50UC
V1.UC51.4	Spain	Female	44	Meta Hit	UC	23.31	0	4	51UC
V1.UC52.1	Spain	Female	43	Meta Hit	UC	29.34	5	1	52UC
V1.UC53.4	Spain	Female	24	Meta Hit	UC	19.59	0	4	53UC
V1.UC54.4	Spain	Male	32	Meta Hit	UC	35.93	0	4	54UC
V1.UC55.4	Spain	Female	46	Meta Hit	UC	18.82	0	4	55UC
V1.UC56.1	Spain	Female	35	Meta Hit	UC	18.83	6	1	56UC
V1.UC58.4	Spain	Female	41	Meta Hit	UC	28.76	1	4	58UC
V1.UC59.4	Spain	Female	46	Meta Hit	UC	23.83	8	4	59UC
V1.UC1.3	Spain	Female	31	Meta Hit	UC	18.48	0	3	1UC
V1.UC2.3	Spain	Male	62	Meta Hit	UC			3	2UC
V1.UC3.1	Spain	Male	44	Meta Hit	UC			1	3UC
V1.UC10.1	Spain	Male	45	Meta Hit	UC			1	10UC
V1.UC12.3	Spain	Female	41	Meta Hit	UC			3	12UC
V1.UC13.2	Spain	Female	51	Meta Hit	UC			2	13UC
V1.UC15.2	Spain	Female	25	Meta Hit	UC			2	15UC
V1.UC17.1	Spain	Female	41	Meta Hit	UC			1	17UC
V1.UC22.1	Spain	Male	37	Meta Hit	HR	22.53		1	22HR
V1.UC26.3	Spain	Female	36	Meta Hit	UC			3	26UC
V1.UC58.3	Spain	Female	41	Meta Hit	UC			3	58UC
V1.UC59.3	Spain	Female	46	Meta Hit	UC			3	59UC

V1.CD2.0.PN	Spain	Female	22	MetaHit	Healthy	23.92	0	2PNHealthy
V1.CD4.0.PN	Spain	Female	60	MetaHit	Healthy	19.98	0	4PNHealthy
V1.CD6.0.PN	Spain	Male	69	MetaHit	Healthy	28.09	0	6PNHealthy
V1.CD8.0.PN	Spain	Male	20	MetaHit	Healthy	21.55	0	8PNHealthy
V1.CD6.0.PT	Spain	Female	51	MetaHit	Healthy	23.03	0	6PTHealthy
V1.CD2.0	Spain	Male	49	MetaHit	Healthy	27.76	0	2Healthy
V1.CD3.0	Spain	Female	18	MetaHit	Healthy	21.51	0	3Healthy
V1.CD4.0	Spain	Female	46	MetaHit	Healthy	29.69	0	4Healthy
V1.CD8.0	Spain	Male	51	MetaHit	Healthy	29.38	0	8Healthy
V1.CD9.0	Spain	Female	48	MetaHit	Healthy	27.55	0	9Healthy
V1.CD11.0	Spain	Female	62	MetaHit	Healthy	35.46	0	11Healthy
V1.CD13.0	Spain	Male	68	MetaHit	Healthy	25.69	0	13Healthy
V1.CD14.0	Spain	Female	41	MetaHit	Healthy	23.12	0	14Healthy
V1.CD16.0	Spain	Female	68	MetaHit	Healthy	21.61	0	16Healthy
V1.CD19.0	Spain	Male	42	MetaHit	Healthy	25.95	0	19Healthy
V1.CD22.0	Spain	Male	68	MetaHit	Healthy	24.24	0	22Healthy
V1.CD24.0	Spain	Female	41	MetaHit	Healthy	20.58	0	24Healthy
V1.CD27.0	Spain	Male	36	MetaHit	Healthy	24.81	0	27Healthy
V1.CD28.0	Spain	Male	24	MetaHit	Healthy	21.46	0	28Healthy
V1.CD29.0	Spain	Male	60	MetaHit	Healthy	31.25	0	29Healthy
V1.CD30.0	Spain	Female	59	MetaHit	Healthy	19.82	0	30Healthy
V1.CD31.0	Spain	Female	28	MetaHit	Healthy	22.6	0	31Healthy
V1.CD34.0	Spain	Female	54	MetaHit	Healthy	23.61	0	34Healthy
V1.CD36.0	Spain	Male	20	MetaHit	Healthy	22.04	0	36Healthy
V1.CD38.0	Spain	Female	63	MetaHit	Healthy	26.84	0	38Healthy
V1.CD40.0	Spain	Female	46	MetaHit	Healthy	23.73	0	40Healthy
V1.CD42.0	Spain	Male	48	MetaHit	Healthy	24.82	0	42Healthy
V1.CD43.0	Spain	Female	45	MetaHit	Healthy	19.53	0	43Healthy

V1.CD45.0	Spain	Male	18	MetaHit	Healthy	29.6				0	45Healthy
V1.CD49.0	Spain	Female	52	MetaHit	Healthy	39.96				0	49Healthy
V1.CD50.0	Spain	Female	18	MetaHit	Healthy	21.48				0	50Healthy
V1.CD52.0	Spain	Male	55	MetaHit	Healthy	32.49				0	52Healthy
V1.CD55.0	Spain	Female	59	MetaHit	Healthy	32.46				0	55Healthy
V1.CD1.0.PN	Spain	Female	54	MetaHit	CD	25.97	3	L1	B1	0	1PNCD
V1.CD3.0.PN	Spain	Female	54	MetaHit	CD	19.03	1	L1	B2	0	3PNCD
V1.CD5.0.PN	Spain	Female	35	MetaHit	CD	28.83	2	L1	B2	0	5PNCD
V1.CD7.0.PN	Spain	Male	51	MetaHit	CD	22.5	2	L1	B2	0	7PNCD
V1.CD1.0.PT	Spain	Female	26	MetaHit	CD	17.9	1	L3	B2	0	1PTCD
V1.CD2.0.PT	Spain	Male	58	MetaHit	CD	20.4	2	L1	B2	0	2PTCD
V1.CD3.0.PT	Spain	Female	33	MetaHit	CD	25	1	L3	B2	0	3PTCD
V1.CD1.0	Spain	Female	25	MetaHit	CD	17.93	1	L3	B3	0	1CD
V1.CD6.0	Spain	Female	36	MetaHit	CD	18.52	0	L3	B2	0	6CD
V1.CD7.0	Spain	Male	22	MetaHit	CD	18.17	0	L1	B2	0	7CD
V1.CD10.0	Spain	Female	38	MetaHit	CD	29.38	0	L3	B2	0	10CD
V1.CD12.0	Spain	Female	41	MetaHit	CD	20.2	1	L3	B2	0	12CD
V1.CD15.0	Spain	Female	34	MetaHit	CD	19	0	L3	B2	0	15CD
V1.CD17.0	Spain	Male	21	MetaHit	CD	21.68	0	L3	B2	0	17CD
V1.CD18.0	Spain	Male	18	MetaHit	CD	19.6	0	L3	B3	0	18CD
V1.CD20.0	Spain	Male	26	MetaHit	CD	19.6	1	L3+L4	B3	0	20CD
V1.CD21.0	Spain	Female	34	MetaHit	CD	19.05		L3	B2	0	21CD
V1.CD25.0	Spain	Female	41	MetaHit	CD	16.94	0	L3	B2	0	25CD
V1.CD32.0	Spain	Female	56	MetaHit	CD	25.32	0	L3	B2	0	32CD
V1.CD35.0	Spain	Female	27	MetaHit	CD	28.72	1	L1	B2	0	35CD
V1.CD41.0	Spain	Female	25	MetaHit	CD	19.04	0	L1+L4	B2	0	41CD
V1.CD44.0	Spain	Female	47	MetaHit	CD	31.89	4	L3	B2	0	44CD
V1.CD46.0	Spain	Male	30	MetaHit	CD	22.99	0	L3	B3	0	46CD

V1.CD48.0	Spain	Female	23	MetaHit	CD	20.24	0	L3	B2P	0	48CD
V1.CD51.0	Spain	Male	29	MetaHit	CD	20.88	0	L3	B3	0	51CD
V1.CD53.0	Spain	Female	47	MetaHit	CD	19.71	4	L3	B2	0	53CD
V1.CD54.0	Spain	Female	27	MetaHit	CD	21.53	1	L3	B2	0	54CD
V1.CD4.0.PT	Spain	Male	61	MetaHit	Healthy	22.99				0	4PTHealthy
V1.CD7.0.PT	Spain	Male	53	MetaHit	CD	24.2	4	L1	B3	0	7PTCD
V1.CD8.0.PT	Spain	Female	62	MetaHit	Healthy	28.4				0	8PTHealthy
V1.CD9.0.PT	Spain	Female	45	MetaHit	CD	22.6	0	L3	B3	0	9PTCD
V1.CD23.0	Spain	Female	66	MetaHit	Healthy	24.39				0	23Healthy
V1.CD33.0	Spain	Male	26	MetaHit	Healthy	23.78				0	33Healthy
V1.CD5.0.PT	Spain	Male	22	MetaHit	CD	26.59	1	L3	B2	0	5PTCD
V1.CD26.0	Spain	Male	39	MetaHit	CD	26.81	5	L3	B1	0	26CD
V1.CD37.0	Spain	Male	35	MetaHit	CD	19.84	0	L1	B2P	0	37CD
V1.CD39.0	Spain	Female	20	MetaHit	CD	21.3	0	L1	B1P	0	39CD
V1.CD1.3.PN	Spain	Female	54	MetaHit	CD	25.97	2			3	1PNCD
V1.CD3.3.PN	Spain	Female	54	MetaHit	CD	20	1			3	3PNCD
V1.CD1.5.PT	Spain	Female	26	MetaHit	CD	18.69	0			5	1PTCD
V1.CD3.4.PT	Spain	Female	33	MetaHit	CD	27.34	0			4	3PTCD
V1.CD6.4	Spain	Female	36	MetaHit	CD	19.77	1			4	6CD
V1.CD7.4	Spain	Male	22	MetaHit	CD	19.49	0			4	7CD
V1.CD12.3	Spain	Female	41	MetaHit	CD	20.94	1			3	12CD
V1.CD15.3	Spain	Female	34	MetaHit	CD	19.36	1			3	15CD
V1.CD17.4	Spain	Male	21	MetaHit	CD	23.41	0			4	17CD
V1.CD18.3	Spain	Male	18	MetaHit	CD	17.96	3			3	18CD
V1.CD20.4	Spain	Male	26	MetaHit	CD	21.56	0			4	20CD
V1.CD21.4	Spain	Female	34	MetaHit	CD	19.05	2			4	21CD
V1.CD25.4	Spain	Female	41	MetaHit	CD	17.31	0			4	25CD
V1.CD32.4	Spain	Female	56	MetaHit	CD	24.52	3			4	32CD

V1.CD35.1	Spain	Female	27	MetaHit	CD		29.41	5			1	35CD
V1.CD37.4	Spain	Male	35	MetaHit	CD		22.23	0			4	37CD
V1.CD41.3	Spain	Female	25	MetaHit	CD		20.35	4			3	41CD
V1.CD44.4	Spain	Female	47	MetaHit	CD		32.95	5			4	44CD
V1.CD48.4	Spain	Female	23	MetaHit	CD		23.21	0			4	48CD
V1.CD53.2	Spain	Female	47	MetaHit	CD		19.33	7			2	53CD
V1.CD54.4	Spain	Female	27	MetaHit	CD		21.75	0			4	54CD
V1.CD3.5.PN	Spain	Female	54	MetaHit	CD		20.03	1			5	3PNCD
V1.CD5.4.PN	Spain	Female	35	MetaHit	CD		30.12	2			4	5PNCD
V1.CD7.6.PN	Spain	Male	51	MetaHit	CD		24.45	1			6	7PNCD
V1.CD5.4	Spain	Male	33	MetaHit	CD		29.07	0			4	5CD
V1.CD26.4	Spain	Male	39	MetaHit	CD		28.41	3			4	26CD
V1.CD51.4	Spain	Male	29	MetaHit	CD		20.88				4	51CD
V1.CD9.3.PT	Spain	Female	45	MetaHit	CD		22.6				3	9PTCD
V1.CD2.2.PT	Spain	Male	58	MetaHit	CD		20.4				2	2PTCD
V1.CD54.3	Spain	Female	27	MetaHit	CD		21.75				3	54CD
V1.CD7.2.PT	Spain	Male	53	MetaHit	CD		24.2				2	7PTCD
V1.CD5.5.PT	Spain	Male	22	MetaHit	CD		26.59				5	5PTCD
V1.UC45.2	Spain	Female	50	MetaHit	UC		23.05				2	45UC
V1.CD46.2	Spain	Male	30	MetaHit	CD		22.99				2	46CD
V1.UC5.4	Spain	Female	30	MetaHit	UC		20.32				4	5UC
V1.CD39.4	Spain	Female	20	MetaHit	CD		21.3				4	39CD
V1.CD15.4	Spain	Female	34	MetaHit	CD						4	15CD
CSM5FZ3N_P	USA	Female	43	HMP2	CD	NA		4	NA	L3	0	C3001
CSM5FZ3R_P	USA	Female	43	HMP2	CD	NA		4	NA	L3	2	C3001
CSM5YRY7_P	USA	Female	43	HMP2	CD	NA		5	NA	L3	4	C3001
CSM5FZ3V_P	USA	Female	43	HMP2	CD	NA		5	NA	L3	6	C3001
CSM5FZ4C_P	USA	Female	43	HMP2	CD	NA		3	NA	L3	8	C3001

CSM5MCVD_P	USA	Female	43	HMP2	CD	NA	5	NA	L3	12	C3001
CSM5MCVF_P	USA	Female	43	HMP2	CD	NA	3	NA	L3	14	C3001
CSM5MCVV_P	USA	Female	43	HMP2	CD	NA	3	NA	L3	16	C3001
CSM5MCWI_P	USA	Female	43	HMP2	CD	NA	5	NA	L3	18	C3001
CSM5MCXD	USA	Female	43	HMP2	CD	NA	5	NA	L3	20	C3001
CSM5MCYS	USA	Female	43	HMP2	CD	NA	4	NA	L3	24	C3001
CSM67U9J	USA	Female	43	HMP2	CD	NA	4	NA	L3	26	C3001
CSM67UA2	USA	Female	43	HMP2	CD	NA	4	NA	L3	28	C3001
CSM67UGC	USA	Female	43	HMP2	CD	NA	5	NA	L3	34	C3001
CSM79HG5	USA	Female	43	HMP2	CD	NA	7	NA	L3	36	C3001
CSM79HGP	USA	Female	43	HMP2	CD	NA	7	NA	L3	38	C3001
CSM5FZ3T_P	USA	Female	76	HMP2	CD	NA	9	NA	L3	0	C3002
CSM5FZ3X_P	USA	Female	76	HMP2	CD	NA	7	NA	L3	2	C3002
CSM5FZ3Z_P	USA	Female	76	HMP2	CD	NA	8	NA	L3	4	C3002
CSM5FZ42_P	USA	Female	76	HMP2	CD	NA	7	NA	L3	6	C3002
CSM5FZ44_P	USA	Female	76	HMP2	CD	NA	7	NA	L3	8	C3002
CSM5FZ46_P	USA	Female	76	HMP2	CD	NA	6	NA	L3	10	C3002
CSM5MCVJ_P	USA	Female	76	HMP2	CD	NA	8	NA	L3	12	C3002
CSM5MCVL	USA	Female	76	HMP2	CD	NA	11	NA	L3	14	C3002
CSM5MCVN	USA	Female	76	HMP2	CD	NA	4	NA	L3	16	C3002
CSM67UBF	USA	Female	76	HMP2	CD	NA	5	NA	L3	24	C3002
CSM67UBH	USA	Female	76	HMP2	CD	NA	5	NA	L3	26	C3002
CSM67UBN	USA	Female	76	HMP2	CD	NA	7	NA	L3	32	C3002
CSM67UBR	USA	Female	76	HMP2	CD	NA	8	NA	L3	34	C3002
CSM79HJW	USA	Female	76	HMP2	CD	NA	6	NA	L3	36	C3002
CSM79HJY	USA	Female	76	HMP2	CD	NA	7	NA	L3	38	C3002
CSM5FZ4E_P	USA	Female	43	HMP2	UC	NA	NA	2		2	C3003
CSM5FZ4G_P	USA	Female	43	HMP2	UC	NA	NA	2		3	C3003

CSM5FZ4K_P	USA	Female	43	HMP2	UC	NA	NA	2	7	C3003
CSM5FZ4M	USA	Female	43	HMP2	UC	NA	NA	4	9	C3003
CSM5MCWM_P	USA	Female	43	HMP2	UC	NA	NA	3	11	C3003
CSM5MCWQ	USA	Female	43	HMP2	UC	NA	NA	2	16	C3003
CSM67UBX	USA	Female	43	HMP2	UC	NA	NA	2	23	C3003
CSM67UBZ	USA	Female	43	HMP2	UC	NA	NA	2	26	C3003
CSM67UC6	USA	Female	43	HMP2	UC	NA	NA	3	34	C3003
CSM79HLM	USA	Female	43	HMP2	UC	NA	NA	1	38	C3003
CSM5FZ4A_P	USA	Female	47	HMP2	UC	NA	NA	0	0	C3004
CSM5MCU8_P	USA	Female	47	HMP2	UC	NA	NA	0	4	C3004
CSM5MCUA_P	USA	Female	47	HMP2	UC	NA	NA	0	6	C3004
CSM5MCUC_P	USA	Female	47	HMP2	UC	NA	NA	1	8	C3004
CSM5MCUE_P	USA	Female	47	HMP2	UC	NA	NA	1	10	C3004
CSM5MCXH	USA	Female	47	HMP2	UC	NA	NA	1	13	C3004
CSM5MCXJ	USA	Female	47	HMP2	UC	NA	NA	0	14	C3004
CSM5MCXL	USA	Female	47	HMP2	UC	NA	NA	1	16	C3004
CSM5MCXN	USA	Female	47	HMP2	UC	NA	NA	1	18	C3004
CSM5MCXP	USA	Female	47	HMP2	UC	NA	NA	1	20	C3004
CSM5MCXR	USA	Female	47	HMP2	UC	NA	NA	1	22	C3004
CSM67UDF	USA	Female	47	HMP2	UC	NA	NA	1	24	C3004
CSM67UDJ	USA	Female	47	HMP2	UC	NA	NA	2	26	C3004
CSM67UDN	USA	Female	47	HMP2	UC	NA	NA	4	28	C3004
CSM67UDR_TR	USA	Female	47	HMP2	UC	NA	NA	6	30	C3004
CSM67UDR	USA	Female	47	HMP2	UC	NA	NA	6	30	C3004
CSM67UDY	USA	Female	47	HMP2	UC	NA	NA	5	34	C3004
CSM79HLA_TR	USA	Female	47	HMP2	UC	NA	NA	4	36	C3004
CSM79HLA	USA	Female	47	HMP2	UC	NA	NA	4	36	C3004
CSM79HLG	USA	Female	47	HMP2	UC	NA	NA	2	38	C3004

CSM79HLE	USA	Female	47	HMP2	UC	NA		NA	6	40	C3004
CSM79HLC	USA	Female	47	HMP2	UC	NA		NA	2	42	C3004
CSM79HLI	USA	Female	47	HMP2	UC	NA		NA	3	44	C3004
CSM79HLK	USA	Female	47	HMP2	UC	NA		NA	6	46	C3004
CSM5MCUQ_P	USA	Female	76	HMP2	UC		30.9	NA	1	1	C3005
CSM5MCUS_P	USA	Female	76	HMP2	UC		30.9	NA	0	4	C3005
CSM5MCUW_P	USA	Female	76	HMP2	UC		30.9	NA	1	7	C3005
CSM5MCUY_P	USA	Female	76	HMP2	UC		30.9	NA	0	9	C3005
CSM5MCY4	USA	Female	76	HMP2	UC		30.9	NA	1	12	C3005
CSM5MCY8	USA	Female	76	HMP2	UC		30.9	NA	0	15	C3005
CSM67UE3	USA	Female	76	HMP2	UC		30.9	NA	0	24	C3005
CSM67UE7	USA	Female	76	HMP2	UC		30.9	NA	0	25	C3005
CSM67UEA	USA	Female	76	HMP2	UC		30.9	NA	1	27	C3005
CSM67UEM	USA	Female	76	HMP2	UC		30.9	NA	1	33	C3005
CSM67UEI	USA	Female	76	HMP2	UC		30.9	NA	0	36	C3005
CSM79HO1	USA	Female	76	HMP2	UC		30.9	NA	0	38	C3005
CSM5MCTZ_P	USA	Male	32	HMP2	UC		20.1	NA	2	0	C3006
CSM5MCUG_P	USA	Male	32	HMP2	UC		20.1	NA	3	1	C3006
CSM5MCUK_P	USA	Male	32	HMP2	UC		20.1	NA	1	6	C3006
CSM5MCUO	USA	Male	32	HMP2	UC		20.1	NA	1	9	C3006
CSM5MCX3	USA	Male	32	HMP2	UC		20.1	NA	1	15	C3006
CSM67UFV	USA	Male	32	HMP2	UC		20.1	NA	1	23	C3006
CSM67UFZ	USA	Male	32	HMP2	UC		20.1	NA	1	25	C3006
CSM67UG8	USA	Male	32	HMP2	UC		20.1	NA	0	33	C3006
CSM79HMN	USA	Male	32	HMP2	UC		20.1	NA	0	35	C3006
CSM79HMP	USA	Male	32	HMP2	UC		20.1	NA	0	37	C3006
CSM79HMT	USA	Male	32	HMP2	UC		20.1	NA	0	41	C3006
CSM5MCVB_P	USA	Female	NA	HMP2	CD	NA		7	NA	0	C3007

CSM5MCV1_P	USA	Female	NA	HMP2	CD	NA	3	NA		0	C3007
CSM5MCV5_P	USA	Female	NA	HMP2	CD	NA	6	NA		5	C3007
CSM5MCU4_P	USA	Female	53	HMP2	CD	21.7	0	NA		0	C3008
CSM5MCVZ_P	USA	Female	53	HMP2	CD	21.7	1	NA		4	C3008
CSM5MCW4_P	USA	Female	53	HMP2	CD	21.7	1	NA		7	C3008
CSM5MCW6	USA	Female	53	HMP2	CD	21.7	1	NA		8	C3008
CSM5MCYW	USA	Female	53	HMP2	CD	21.7	0	NA		10	C3008
CSM5MCZ3	USA	Female	53	HMP2	CD	21.7	1	NA		16	C3008
CSM5MCZ5	USA	Female	53	HMP2	CD	21.7	1	NA		24	C3008
CSM5MCZ7	USA	Female	53	HMP2	CD	21.7	1	NA		27	C3008
CSM79HKT	USA	Female	53	HMP2	CD	21.7	2	NA		32	C3008
CSM79HKV	USA	Female	53	HMP2	CD	21.7	1	NA		34	C3008
CSM79HQ9	USA	Female	53	HMP2	CD	21.7	2	NA		36	C3008
CSM79HQB	USA	Female	53	HMP2	CD	21.7	1	NA		38	C3008
CSM79HQF	USA	Female	53	HMP2	CD	21.7	1	NA		42	C3008
CSM5MCWA_P	USA	Male	56	HMP2	CD	NA	4	NA	L3	4	C3009
CSM5MCWC	USA	Male	56	HMP2	CD	NA	4	NA	L3	6	C3009
CSM5MCWE	USA	Male	56	HMP2	CD	NA	3	NA	L3	8	C3009
CSM5MCWG	USA	Male	56	HMP2	CD	NA	3	NA	L3	10	C3009
CSM67UAA	USA	Male	56	HMP2	CD	NA	2	NA	L3	16	C3009
CSM67UAG	USA	Male	56	HMP2	CD	NA	3	NA	L3	22	C3009
CSM79HI3	USA	Male	56	HMP2	CD	NA	3	NA	L3	26	C3009
CSM79HI7	USA	Male	56	HMP2	CD	NA	0	NA	L3	30	C3009
CSM79HIB	USA	Male	56	HMP2	CD	NA	0	NA	L3	34	C3009
CSM7KOJE	USA	Male	56	HMP2	CD	NA	1	NA	L3	36	C3009
CSM7KOJG	USA	Male	56	HMP2	CD	NA	1	NA	L3	38	C3009
CSM7KOJO	USA	Male	56	HMP2	CD	NA	1	NA	L3	46	C3009
CSM5MCWK_P	USA	Female	51	HMP2	CD	50.2	1	NA	L3	0	C3010

CSM5MCXT	USA	Female	51	HMP2	CD	50.2	1	NA	L3	2	C3010
CSM5MCXV	USA	Female	51	HMP2	CD	50.2	1	NA	L3	4	C3010
CSM5MCXX_P	USA	Female	51	HMP2	CD	50.2	1	NA	L3	5	C3010
CSM5MCXZ_P	USA	Female	51	HMP2	CD	50.2	2	NA	L3	7	C3010
CSM5MCY2	USA	Female	51	HMP2	CD	50.2	3	NA	L3	9	C3010
CSM67UCK	USA	Female	51	HMP2	CD	50.2	1	NA	L3	16	C3010
CSM79HK9	USA	Female	51	HMP2	CD	50.2	1	NA	L3	23	C3010
CSM79HKB	USA	Female	51	HMP2	CD	50.2	0	NA	L3	26	C3010
CSM79HOF	USA	Female	51	HMP2	CD	50.2	0	NA	L3	31	C3010
CSM79HOH	USA	Female	51	HMP2	CD	50.2	3	NA	L3	33	C3010
CSM7KOL4	USA	Female	51	HMP2	CD	50.2	0	NA	L3	35	C3010
CSM7KOLA	USA	Female	51	HMP2	CD	50.2	2	NA	L3	41	C3010
CSM7KOLE	USA	Female	51	HMP2	CD	50.2	2	NA	L3	45	C3010
CSM5MCXB_P	USA	Female	37	HMP2	UC	41.5	NA	2		0	C3011
CSM5MCYI_P	USA	Female	37	HMP2	UC	41.5	NA	2		1	C3011
CSM5MCYM_P	USA	Female	37	HMP2	UC	41.5	NA	2		3	C3011
CSM5MCYO_P	USA	Female	37	HMP2	UC	41.5	NA	3		6	C3011
CSM5MCYQ_P	USA	Female	37	HMP2	UC	41.5	NA	2		8	C3011
CSM67UEP_P	USA	Female	37	HMP2	UC	41.5	NA	4		10	C3011
CSM67UET_P	USA	Female	37	HMP2	UC	41.5	NA	4		12	C3011
CSM67UEW_TR	USA	Female	37	HMP2	UC	41.5	NA	2		14	C3011
CSM67UEW_P	USA	Female	37	HMP2	UC	41.5	NA	2		14	C3011
CSM67UEW	USA	Female	37	HMP2	UC	41.5	NA	2		14	C3011
CSM67UF1_P	USA	Female	37	HMP2	UC	41.5	NA	1		16	C3011
CSM67UF1	USA	Female	37	HMP2	UC	41.5	NA	1		16	C3011
CSM67UF5	USA	Female	37	HMP2	UC	41.5	NA	1		19	C3011
CSM79HM1	USA	Female	37	HMP2	UC	41.5	NA	1		24	C3011
CSM79HQT_P	USA	Female	37	HMP2	UC	41.5	NA	1		27	C3011

CSM79HM5_P	USA	Female	37	HMP2	UC	41.5	NA	2		31	C3011
CSM79HM7	USA	Female	37	HMP2	UC	41.5	NA	1		32	C3011
CSM79HM9_P	USA	Female	37	HMP2	UC	41.5	NA	1		34	C3011
CSM7KOMP	USA	Female	37	HMP2	UC	41.5	NA	2		37	C3011
CSM7KOMR_P	USA	Female	37	HMP2	UC	41.5	NA	4		39	C3011
CSM7KOMT	USA	Female	37	HMP2	UC	41.5	NA	1		41	C3011
CSM7KOMV_P	USA	Female	37	HMP2	UC	41.5	NA	1		44	C3011
CSM5MCXF_P	USA	Female	37	HMP2	CD	46.7	1	NA	L1	0	C3012
CSM5MCZB	USA	Female	37	HMP2	CD	46.7	3	NA	L1	3	C3012
CSM5MCZD	USA	Female	37	HMP2	CD	46.7	0	NA	L1	4	C3012
CSM5MCZF	USA	Female	37	HMP2	CD	46.7	0	NA	L1	6	C3012
CSM67U9B	USA	Female	37	HMP2	CD	46.7	2	NA	L1	8	C3012
CSM67U9D	USA	Female	37	HMP2	CD	46.7	0	NA	L1	10	C3012
CSM67UGO	USA	Female	37	HMP2	CD	46.7	0	NA	L1	16	C3012
CSM79HOJ	USA	Female	37	HMP2	CD	46.7	0	NA	L1	24	C3012
CSM79HOL	USA	Female	37	HMP2	CD	46.7	1	NA	L1	26	C3012
CSM79HOT	USA	Female	37	HMP2	CD	46.7	0	NA	L1	34	C3012
CSM7KOMX	USA	Female	37	HMP2	CD	46.7	0	NA	L1	36	C3012
CSM7KOMZ	USA	Female	37	HMP2	CD	46.7	2	NA	L1	38	C3012
CSM7KON2	USA	Female	37	HMP2	CD	46.7	1	NA	L1	40	C3012
CSM7KON8	USA	Female	37	HMP2	CD	46.7	0	NA	L1	46	C3012
CSM5MCYU_P	USA	Female	26	HMP2	UC	22.2	NA	3		0	C3013
CSM67U9H_P	USA	Female	26	HMP2	UC	22.2	NA	3		2	C3013
CSM67U9H	USA	Female	26	HMP2	UC	22.2	NA	3		2	C3013
CSM67U9N	USA	Female	26	HMP2	UC	22.2	NA	8		6	C3013
CSM67U9P_P	USA	Female	26	HMP2	UC	22.2	NA	5		8	C3013
CSM67U9R_P	USA	Female	26	HMP2	UC	22.2	NA	3		10	C3013
CSM79HGD_P	USA	Female	26	HMP2	UC	22.2	NA	3		12	C3013

CSM79HGF_P	USA	Female	26	HMP2	UC	22.2	NA	3	14	C3013
CSM79HGF	USA	Female	26	HMP2	UC	22.2	NA	3	14	C3013
CSM79HGH_P	USA	Female	26	HMP2	UC	22.2	NA	3	17	C3013
CSM79HGJ_P	USA	Female	26	HMP2	UC	22.2	NA	5	18	C3013
CSM79HGL_P	USA	Female	26	HMP2	UC	22.2	NA	4	20	C3013
CSM79HGN_P	USA	Female	26	HMP2	UC	22.2	NA	5	22	C3013
CSM79HPK	USA	Female	26	HMP2	UC	22.2	NA	6	24	C3013
CSM79HPM_P	USA	Female	26	HMP2	UC	22.2	NA	5	26	C3013
CSM79HPS	USA	Female	26	HMP2	UC	22.2	NA	7	31	C3013
CSM79HPQ_P	USA	Female	26	HMP2	UC	22.2	NA	9	32	C3013
CSM79HPO	USA	Female	26	HMP2	UC	22.2	NA	8	33	C3013
CSM79HPU	USA	Female	26	HMP2	UC	22.2	NA	9	34	C3013
CSM7KONS_P	USA	Female	26	HMP2	UC	22.2	NA	5	36	C3013
CSM7KONU	USA	Female	26	HMP2	UC	22.2	NA	7	38	C3013
CSM7KONW_P	USA	Female	26	HMP2	UC	22.2	NA	6	41	C3013
CSM67U9T_P	USA	Female	50	HMP2	UC	22.4	NA	3	0	C3015
CSM67UAK	USA	Female	50	HMP2	UC	22.4	NA	4	3	C3015
CSM67UAM	USA	Female	50	HMP2	UC	22.4	NA	0	4	C3015
CSM67UAO	USA	Female	50	HMP2	UC	22.4	NA	1	6	C3015
CSM67UAQ	USA	Female	50	HMP2	UC	22.4	NA	0	8	C3015
CSM67UAS	USA	Female	50	HMP2	UC	22.4	NA	1	10	C3015
CSM79HID	USA	Female	50	HMP2	UC	22.4	NA	0	12	C3015
CSM79HIF	USA	Female	50	HMP2	UC	22.4	NA	1	14	C3015
CSM79HIH	USA	Female	50	HMP2	UC	22.4	NA	1	16	C3015
CSM79HIJ	USA	Female	50	HMP2	UC	22.4	NA	0	18	C3015
CSM79HIL	USA	Female	50	HMP2	UC	22.4	NA	0	20	C3015
CSM79HIN	USA	Female	50	HMP2	UC	22.4	NA	0	22	C3015
CSM7KOJQ	USA	Female	50	HMP2	UC	22.4	NA	1	24	C3015

CSM7KOJS	USA	Female	50	HMP2	UC	22.4	NA	0	26	C3015
CSM7KOJU	USA	Female	50	HMP2	UC	22.4	NA	0	28	C3015
CSM7KOJW	USA	Female	50	HMP2	UC	22.4	NA	0	30	C3015
CSM7KOJY	USA	Female	50	HMP2	UC	22.4	NA	0	32	C3015
CSM7KOK1	USA	Female	50	HMP2	UC	22.4	NA	0	34	C3015
CSM7KOPG	USA	Female	50	HMP2	UC	22.4	NA	0	36	C3015
CSM7KOPI	USA	Female	50	HMP2	UC	22.4	NA	1	38	C3015
CSM7KOPK	USA	Female	50	HMP2	UC	22.4	NA	NA	40	C3015
CSM7KOPM	USA	Female	50	HMP2	UC	22.4	NA	1	42	C3015
CSM7KOPO	USA	Female	50	HMP2	UC	22.4	NA	0	44	C3015
CSM67U9V_P	USA	Female	32	HMP2	CD	24.7	5	NA	0	C3016
CSM67UAU	USA	Female	32	HMP2	CD	24.7	8	NA	2	C3016
CSM67UAW	USA	Female	32	HMP2	CD	24.7	7	NA	4	C3016
CSM67UAY	USA	Female	32	HMP2	CD	24.7	8	NA	6	C3016
CSM67UB1	USA	Female	32	HMP2	CD	24.7	2	NA	8	C3016
CSM67UB3	USA	Female	32	HMP2	CD	24.7	5	NA	10	C3016
CSM79HIR	USA	Female	32	HMP2	CD	24.7	4	NA	14	C3016
CSM79HIT	USA	Female	32	HMP2	CD	24.7	5	NA	16	C3016
CSM79HIV	USA	Female	32	HMP2	CD	24.7	4	NA	18	C3016
CSM79HIX	USA	Female	32	HMP2	CD	24.7	8	NA	20	C3016
CSM79HIZ	USA	Female	32	HMP2	CD	24.7	8	NA	22	C3016
CSM7KOK3	USA	Female	32	HMP2	CD	24.7	10	NA	24	C3016
CSM7KOK5	USA	Female	32	HMP2	CD	24.7	19	NA	26	C3016
CSM7KOK7	USA	Female	32	HMP2	CD	24.7	10	NA	28	C3016
CSM7KOKB	USA	Female	32	HMP2	CD	24.7	12	NA	32	C3016
CSM7KOKD	USA	Female	32	HMP2	CD	24.7	NA	NA	34	C3016
CSM7KOPS	USA	Female	32	HMP2	CD	24.7	NA	NA	36	C3016
CSM7KOPU	USA	Female	32	HMP2	CD	24.7	14	NA	38	C3016

CSM7KOPW	USA	Female	32	HMP2	CD		24.7	12	NA		40	C3016
CSM7KOQ1	USA	Female	32	HMP2	CD		24.7	9	NA		44	C3016
CSM67U9X_P	USA	Male	45	HMP2	CD		24.2	2	NA	L3	0	C3017
CSM67UB5_P	USA	Male	45	HMP2	CD		24.2	1	NA	L3	2	C3017
CSM67UB7_P	USA	Male	45	HMP2	CD		24.2	1	NA	L3	4	C3017
CSM67UB9_P	USA	Male	45	HMP2	CD		24.2	4	NA	L3	6	C3017
CSM67UB9	USA	Male	45	HMP2	CD		24.2	4	NA	L3	6	C3017
CSM67UBB	USA	Male	45	HMP2	CD		24.2	1	NA	L3	9	C3017
CSM79HJ2_P	USA	Male	45	HMP2	CD		24.2	1	NA	L3	12	C3017
CSM79HJ4_P	USA	Male	45	HMP2	CD		24.2	1	NA	L3	14	C3017
CSM79HJ6_P	USA	Male	45	HMP2	CD		24.2	1	NA	L3	16	C3017
CSM79HJ8_P	USA	Male	45	HMP2	CD		24.2	1	NA	L3	18	C3017
CSM79HJA	USA	Male	45	HMP2	CD		24.2	1	NA	L3	20	C3017
CSM79HJC_P	USA	Male	45	HMP2	CD		24.2	1	NA	L3	22	C3017
CSM7KOKF	USA	Male	45	HMP2	CD		24.2	1	NA	L3	24	C3017
CSM7KOKH_P	USA	Male	45	HMP2	CD		24.2	1	NA	L3	26	C3017
CSM7KOKJ	USA	Male	45	HMP2	CD		24.2	1	NA	L3	28	C3017
CSM7KOKL_P	USA	Male	45	HMP2	CD		24.2	4	NA	L3	30	C3017
CSM7KOKN	USA	Male	45	HMP2	CD		24.2	1	NA	L3	32	C3017
CSM7KOKP_P	USA	Male	45	HMP2	CD		24.2	1	NA	L3	34	C3017
CSM7KOQX	USA	Male	45	HMP2	CD		24.2	0	NA	L3	37	C3017
CSM7KOQZ_P	USA	Male	45	HMP2	CD		24.2	1	NA	L3	38	C3017
CSM7KOR2	USA	Male	45	HMP2	CD		24.2	1	NA	L3	40	C3017
CSM7KOR4_P	USA	Male	45	HMP2	CD		24.2	1	NA	L3	42	C3017
CSM7KOR8_P	USA	Male	45	HMP2	CD		24.2	5	NA	L3	46	C3017
CSM67UCU_P	USA	Female	39	HMP2	CD	NA		3	NA	L3	2	C3019
CSM67UAI_P	USA	Female	47	HMP2	CD	NA		2	NA	L3	0	C3020
CSM79HHO	USA	Female	38	HMP2	CD		25.6	5	NA	L2	1	C3021

CSM79HHM	USA	Female	38	HMP2	CD		25.6	3	NA	L2	4	C3021
CSM79HHU	USA	Female	38	HMP2	CD		25.6	0	NA	L2	7	C3021
CSM79HN2	USA	Female	38	HMP2	CD		25.6	0	NA	L2	9	C3021
CSM79HN6	USA	Female	38	HMP2	CD		25.6	0	NA	L2	13	C3021
CSM7KOLK	USA	Female	38	HMP2	CD		25.6	2	NA	L2	21	C3021
CSM7KOLM	USA	Female	38	HMP2	CD		25.6	0	NA	L2	24	C3021
CSM7KOSV	USA	Female	38	HMP2	CD		25.6	1	NA	L2	31	C3021
CSM7KOSX	USA	Female	38	HMP2	CD		25.6	0	NA	L2	33	C3021
CSM67UH7	USA	Male	69	HMP2	nonIBD	NA		NA	NA		0	C3022
CSM79HGR_P	USA	Male	69	HMP2	nonIBD	NA		NA	NA		2	C3022
CSM79HGV_P	USA	Male	69	HMP2	nonIBD	NA		NA	NA		6	C3022
CSM79HGX	USA	Male	69	HMP2	nonIBD	NA		NA	NA		8	C3022
CSM79HGZ	USA	Male	69	HMP2	nonIBD	NA		NA	NA		10	C3022
CSM79HOV	USA	Male	69	HMP2	nonIBD	NA		NA	NA		12	C3022
CSM79HOX	USA	Male	69	HMP2	nonIBD	NA		NA	NA		14	C3022
CSM79HOZ	USA	Male	69	HMP2	nonIBD	NA		NA	NA		16	C3022
CSM79HP2	USA	Male	69	HMP2	nonIBD	NA		NA	NA		18	C3022
CSM79HP4	USA	Male	69	HMP2	nonIBD	NA		NA	NA		21	C3022
CSM79HP6	USA	Male	69	HMP2	nonIBD	NA		NA	NA		23	C3022
CSM7KOOH	USA	Male	69	HMP2	nonIBD	NA		NA	NA		26	C3022
CSM7KOOJ	USA	Male	69	HMP2	nonIBD	NA		NA	NA		28	C3022
CSM7KOOL	USA	Male	69	HMP2	nonIBD	NA		NA	NA		30	C3022
CSM7KOON	USA	Male	69	HMP2	nonIBD	NA		NA	NA		32	C3022
CSM7KOOP	USA	Male	69	HMP2	nonIBD	NA		NA	NA		34	C3022
CSM7KOOR	USA	Male	69	HMP2	nonIBD	NA		NA	NA		36	C3022
CSMA8M9R	USA	Male	69	HMP2	nonIBD	NA		NA	NA		40	C3022
CSMAAEUA	USA	Male	69	HMP2	nonIBD	NA		NA	NA		42	C3022
CSMAE44D	USA	Male	69	HMP2	nonIBD	NA		NA	NA		44	C3022

CSMAG78W	USA	Male	69	HMP2	nonIBD	NA		NA	NA		46	C3022
CSMAHYLR	USA	Male	69	HMP2	nonIBD	NA		NA	NA		48	C3022
CSM79HH2_P	USA	Male	60	HMP2	CD		28	3	NA	L3	2	C3023
CSM79HH4	USA	Male	60	HMP2	CD		28	2	NA	L3	4	C3023
CSM79HH8	USA	Male	60	HMP2	CD		28	0	NA	L3	8	C3023
CSM79HHA	USA	Male	60	HMP2	CD		28	2	NA	L3	10	C3023
CSM79HPA_TR	USA	Male	60	HMP2	CD		28	1	NA	L3	14	C3023
CSM79HPA	USA	Male	60	HMP2	CD		28	1	NA	L3	14	C3023
CSM79HPC	USA	Male	60	HMP2	CD		28	1	NA	L3	16	C3023
CSM7KONA	USA	Male	60	HMP2	CD		28	3	NA	L3	24	C3023
CSM7KONK	USA	Male	60	HMP2	CD		28	3	NA	L3	34	C3023
CSM7KOTA	USA	Male	60	HMP2	CD		28	3	NA	L3	36	C3023
CSM7KOTC	USA	Male	60	HMP2	CD		28	1	NA	L3	38	C3023
CSM7KOTK	USA	Male	60	HMP2	CD		28	2	NA	L3	46	C3023
CSM79HG7_P	USA	Female	40	HMP2	CD	NA		4	NA		0	C3024
CSM79HHW_P	USA	Female	36	HMP2	CD	NA		3	NA		0	C3027
CSM79HHW	USA	Female	36	HMP2	CD	NA		3	NA		0	C3027
CSM79HJM	USA	Female	36	HMP2	CD	NA		1	NA		2	C3027
CSM79HJO	USA	Female	36	HMP2	CD	NA		1	NA		3	C3027
CSM79HJQ	USA	Female	36	HMP2	CD	NA		8	NA		5	C3027
CSM79HJS	USA	Female	36	HMP2	CD	NA		4	NA		8	C3027
CSM79HJU	USA	Female	36	HMP2	CD	NA		5	NA		9	C3027
CSM79HQV	USA	Female	36	HMP2	CD	NA		2	NA		11	C3027
CSM79HQX	USA	Female	36	HMP2	CD	NA		0	NA		13	C3027
CSM79HQZ	USA	Female	36	HMP2	CD	NA		3	NA		16	C3027
CSM79HR2	USA	Female	36	HMP2	CD	NA		0	NA		17	C3027
CSM79HR4	USA	Female	36	HMP2	CD	NA		0	NA		20	C3027
CSM79HR6	USA	Female	36	HMP2	CD	NA		2	NA		21	C3027

CSM7KOOT	USA	Female	36	HMP2	CD	NA	2	NA	23	C3027
CSM7KOOV	USA	Female	36	HMP2	CD	NA	2	NA	26	C3027
CSM7KOOX	USA	Female	36	HMP2	CD	NA	NA	NA	28	C3027
CSM7KOOZ	USA	Female	36	HMP2	CD	NA	2	NA	29	C3027
CSM7KOP2	USA	Female	36	HMP2	CD	NA	2	NA	31	C3027
CSMA88CB	USA	Female	36	HMP2	CD	NA	2	NA	36	C3027
CSMA9J65	USA	Female	36	HMP2	CD	NA	3	NA	38	C3027
CSMACTZP	USA	Female	36	HMP2	CD	NA	2	NA	39	C3027
CSMAF72L	USA	Female	36	HMP2	CD	NA	0	NA	41	C3027
CSMAH393	USA	Female	36	HMP2	CD	NA	NA	NA	44	C3027
CSMAIG7X	USA	Female	36	HMP2	CD	NA	2	NA	45	C3027
CSM79HKX	USA	Male	33	HMP2	CD	NA	2	NA	1	C3028
CSM79HKZ	USA	Male	33	HMP2	CD	NA	1	NA	4	C3028
CSM79HL4	USA	Male	33	HMP2	CD	NA	1	NA	8	C3028
CSM79HL6	USA	Male	33	HMP2	CD	NA	2	NA	11	C3028
CSM7KOKR	USA	Male	33	HMP2	CD	NA	2	NA	13	C3028
CSM7KOKT	USA	Male	33	HMP2	CD	NA	1	NA	15	C3028
CSM7KOKZ	USA	Male	33	HMP2	CD	NA	3	NA	21	C3028
CSM7KOL2	USA	Male	33	HMP2	CD	NA	2	NA	24	C3028
CSM7KORC	USA	Male	33	HMP2	CD	NA	4	NA	25	C3028
CSM7KORK	USA	Male	33	HMP2	CD	NA	2	NA	34	C3028
CSM7KORI	USA	Male	33	HMP2	CD	NA	2	NA	36	C3028
CSM7KORG	USA	Male	33	HMP2	CD	NA	2	NA	38	C3028
CSM79HNE	USA	Male	32	HMP2	UC	NA	NA	4	1	C3029
CSM79HNG_P	USA	Male	32	HMP2	UC	NA	NA	4	3	C3029
CSM79HNI	USA	Male	32	HMP2	UC	NA	NA	3	5	C3029
CSM79HNK	USA	Male	32	HMP2	UC	NA	NA	3	7	C3029
CSM79HNM	USA	Male	32	HMP2	UC	NA	NA	3	9	C3029

CSM7KOLY	USA	Male		32	HMP2	UC	NA		NA	2		15	C3029
CSM7KOS7	USA	Male		32	HMP2	UC	NA		NA	1		23	C3029
CSM7KOSH	USA	Male		32	HMP2	UC	NA		NA	1		33	C3029
CSM9X1Y5	USA	Male		32	HMP2	UC	NA		NA	0		35	C3029
CSM79HJI_P	USA	Male		44	HMP2	CD		21.2	1	NA	L2	0	C3030
CSM79HNO	USA	Male		44	HMP2	CD		21.2	2	NA	L2	1	C3030
CSM79HNU	USA	Male		44	HMP2	CD		21.2	2	NA	L2	7	C3030
CSM79HNW	USA	Male		44	HMP2	CD		21.2	3	NA	L2	9	C3030
CSM7KOMB	USA	Male		44	HMP2	CD		21.2	4	NA	L2	16	C3030
CSM7KOMH	USA	Male		44	HMP2	CD		21.2	3	NA	L2	21	C3030
CSM7KOSL	USA	Male		44	HMP2	CD		21.2	2	NA	L2	25	C3030
CSM7KOSP	USA	Male		44	HMP2	CD		21.2	2	NA	L2	29	C3030
CSM7KOST	USA	Male		44	HMP2	CD		21.2	2	NA	L2	34	C3030
CSM7KOSJ	USA	Male		44	HMP2	CD		21.2	2	NA	L2	37	C3030
CSM79HNY_P	USA	Female	NA		HMP2	CD	NA		3	NA		0	C3031
CSM79HR8	USA	Female	NA		HMP2	CD	NA		2	NA		5	C3031
CSM79HRC	USA	Female	NA		HMP2	CD	NA		2	NA		7	C3031
CSM79HRE	USA	Female	NA		HMP2	CD	NA		2	NA		9	C3031
CSM79HRG	USA	Female	NA		HMP2	CD	NA		1	NA		10	C3031
CSM7KOO5	USA	Female	NA		HMP2	CD	NA		1	NA		14	C3031
CSM7KOO9	USA	Female	NA		HMP2	CD	NA		1	NA		19	C3031
CSM7KOOD	USA	Female	NA		HMP2	CD	NA		3	NA		24	C3031
CSM7KOOF	USA	Female	NA		HMP2	CD	NA		4	NA		33	C3031
CSM9X1Z4	USA	Female	NA		HMP2	CD	NA		3	NA		38	C3031
CSM9X1ZC	USA	Female	NA		HMP2	CD	NA		NA	NA		49	C3031
CSM7KOP6	USA	Female		42	HMP2	UC		21.7	NA	2		2	C3032
CSM7KOP8	USA	Female		42	HMP2	UC		21.7	NA	1		4	C3032
CSM7KOPE	USA	Female		42	HMP2	UC		21.7	NA	1		10	C3032

CSM7KOU7_P	USA	Female	42	HMP2	UC		21.7	NA	0		12	C3032
CSM7KOU9	USA	Female	42	HMP2	UC		21.7	NA	0		14	C3032
CSM7KOUB	USA	Female	42	HMP2	UC		21.7	NA	0		16	C3032
CSM9X1ZO	USA	Female	42	HMP2	UC		21.7	NA	1		24	C3032
CSM9X1ZQ	USA	Female	42	HMP2	UC		21.7	NA	0		26	C3032
CSM9X1ZY	USA	Female	42	HMP2	UC		21.7	NA	0		33	C3032
CSM9X22G	USA	Female	42	HMP2	UC		21.7	NA	0		37	C3032
CSM9X22I	USA	Female	42	HMP2	UC		21.7	NA	1		38	C3032
CSM9X22K	USA	Female	42	HMP2	UC		21.7	NA	0		40	C3032
CSM7KOQP_P	USA	Female	40	HMP2	CD	NA		5	NA		8	C3033
CSM79HQR_P	USA	Female	36	HMP2	UC		19.5	NA	6		0	C3034
CSM7KORO	USA	Female	36	HMP2	UC		19.5	NA	4		2	C3034
CSM7KORM	USA	Female	36	HMP2	UC		19.5	NA	2		4	C3034
CSM7KORU	USA	Female	36	HMP2	UC		19.5	NA	2		8	C3034
CSM7KORS	USA	Female	36	HMP2	UC		19.5	NA	2		10	C3034
CSM9X1XU	USA	Female	36	HMP2	UC		19.5	NA	2		14	C3034
CSM9X1Y3	USA	Female	36	HMP2	UC		19.5	NA	1		26	C3034
CSM9X21J	USA	Female	36	HMP2	UC		19.5	NA	2		34	C3034
CSM9X21L	USA	Female	36	HMP2	UC		19.5	NA	3		36	C3034
CSM9X21N	USA	Female	36	HMP2	UC		19.5	NA	3		38	C3034
CSM7KOTO	USA	Male	62	HMP2	CD		24.4	8	NA	L3	4	C3035
CSM7KOTQ	USA	Male	62	HMP2	CD		24.4	5	NA	L3	6	C3035
CSM7KOTS	USA	Male	62	HMP2	CD		24.4	7	NA	L3	8	C3035
CSM7KOTU	USA	Male	62	HMP2	CD		24.4	9	NA	L3	12	C3035
CSM9X1YV	USA	Male	62	HMP2	CD		24.4	7	NA	L3	16	C3035
CSM9X21R	USA	Male	62	HMP2	CD		24.4	7	NA	L3	24	C3035
CSM9X21T	USA	Male	62	HMP2	CD		24.4	9	NA	L3	26	C3035
CSM9X222	USA	Male	62	HMP2	CD		24.4	NA	NA	L3	34	C3035

CSM9X233	USA	Male	62	HMP2	CD		24.4	7	NA	L3	36	C3035
CSM9X235	USA	Male	62	HMP2	CD		24.4	8	NA	L3	38	C3035
CSM9X237	USA	Male	62	HMP2	CD		24.4	NA	NA	L3	41	C3035
CSM9X23B	USA	Male	62	HMP2	CD		24.4	5	NA	L3	44	C3035
CSM7KOQ5_P	USA	Female	26	HMP2	UC	NA		NA	2		0	C3036
CSM7KOUJ_P	USA	Female	46	HMP2	UC		22.4	NA	0		2	C3037
CSM7KOUL	USA	Female	46	HMP2	UC		22.4	NA	0		4	C3037
CSM7KOUN	USA	Female	46	HMP2	UC		22.4	NA	1		6	C3037
CSM9X219	USA	Female	46	HMP2	UC		22.4	NA	1		13	C3037
CSM9X213	USA	Female	46	HMP2	UC		22.4	NA	0		14	C3037
CSM9X215	USA	Female	46	HMP2	UC		22.4	NA	0		16	C3037
CSM9X211	USA	Female	46	HMP2	UC		22.4	NA	0		22	C3037
CSM9X22S	USA	Female	46	HMP2	UC		22.4	NA	0		32	C3037
CSM9X22U	USA	Female	46	HMP2	UC		22.4	NA	2		34	C3037
CSM9X23H	USA	Female	46	HMP2	UC		22.4	NA	1		38	C3037
CSM9X23N	USA	Female	46	HMP2	UC		22.4	NA	3		44	C3037
ESM5MEDZ_P	USA	Female	8	HMP2	CD	NA		6	NA	L3+L4	0	E5001
ESM5MEE2_P	USA	Female	8	HMP2	CD	NA		6	NA	L3+L4	2	E5001
HSM5MEE5_P	USA	Female	8	HMP2	CD	NA		5	NA	L3+L4	4	E5001
ESM5MEE6_P	USA	Female	8	HMP2	CD	NA		4	NA	L3+L4	6	E5001
ESM5ME9D_P	USA	Female	8	HMP2	CD	NA		5	NA	L3+L4	12	E5001
ESM5ME9G_P	USA	Female	8	HMP2	CD	NA		NA	NA	L3+L4	14	E5001
ESM5MEBA_P	USA	Female	8	HMP2	CD	NA		4	NA	L3+L4	15	E5001
ESM5MEBE	USA	Female	8	HMP2	CD	NA		NA	NA	L3+L4	19	E5001
ESM5GEXY	USA	Female	8	HMP2	CD	NA		4	NA	L3+L4	24	E5001
ESM5MEBG	USA	Female	8	HMP2	CD	NA		4	NA	L3+L4	31	E5001
ESM5MECQ	USA	Female	8	HMP2	CD	NA		4	NA	L3+L4	33	E5001
ESM5MEBI	USA	Female	8	HMP2	CD	NA		3	NA	L3+L4	36	E5001

ESM5MECL	USA	Female	8	HMP2	CD	NA	3	NA	L3+L4	37	E5001
ESM5GEYU_P	USA	Female	15	HMP2	nonIBD	NA	NA	NA		2	E5002
ESM5GEZ1_P	USA	Female	15	HMP2	nonIBD	NA	NA	NA		4	E5002
ESM5GEZ3_P	USA	Female	15	HMP2	nonIBD	NA	NA	NA		5	E5002
ESM5MEBP_P	USA	Female	15	HMP2	nonIBD	NA	NA	NA		7	E5002
ESM5ME9H_P	USA	Female	15	HMP2	nonIBD	NA	NA	NA		9	E5002
ESM5GEYX_P	USA	Male	15	HMP2	CD	NA	1	NA	L3	1	E5003
ESM5MEEJ_P	USA	Female	7	HMP2	UC	16.3	NA	2		1	E5004
ESM5GEZ4_P	USA	Female	7	HMP2	UC	16.3	NA	1		3	E5004
ESM5GEZ6_P	USA	Female	7	HMP2	UC	16.3	NA	2		5	E5004
ESM5GEZA_P	USA	Female	7	HMP2	UC	16.3	NA	2		9	E5004
ESM5MEBS	USA	Female	7	HMP2	UC	16.3	NA	1		13	E5004
ESM5MEBU	USA	Female	7	HMP2	UC	16.3	NA	2		15	E5004
ESM5MEC3	USA	Female	7	HMP2	UC	16.3	NA	2		24	E5004
ESM5MEC5	USA	Female	7	HMP2	UC	16.3	NA	1		26	E5004
ESM5MEDU	USA	Female	7	HMP2	UC	16.3	NA	2		32	E5004
ESM5MEC9	USA	Female	7	HMP2	UC	16.3	NA	0		36	E5004
ESM718SY	USA	Female	7	HMP2	UC	16.3	NA	1		39	E5004
ESM5ME9U	USA	Female	7	HMP2	UC	16.3	NA	2		42	E5004
ESM5GEYY_P	USA	Male	NA	HMP2	CD	NA	7	NA		0	E5006
ESM5GEYW_P	USA	Male	NA	HMP2	CD	NA	3	NA		3	E5006
ESM5MEAB_P	USA	Male	13	HMP2	UC	NA	NA	0		1	E5008
ESM5MEA7_P	USA	Male	13	HMP2	UC	NA	NA	8		2	E5008
ESM5MEA9_P	USA	Male	13	HMP2	UC	NA	NA	0		4	E5008
ESM5MEDN	USA	Female	17	HMP2	CD	17.6	1	NA	L1	2	E5009
ESM5MEDP_P	USA	Female	17	HMP2	CD	17.6	2	NA	L1	4	E5009
ESM5MEDK	USA	Female	17	HMP2	CD	17.6	2	NA	L1	7	E5009
ESM5MEDD	USA	Female	17	HMP2	CD	17.6	2	NA	L1	9	E5009

ESM5MEDF	USA	Female		17	HMP2	CD		17.6	2	NA	L1	11	E5009
ESM718UH	USA	Female		17	HMP2	CD		17.6	2	NA	L1	15	E5009
ESM7F5AK	USA	Female		17	HMP2	CD		17.6	1	NA	L1	25	E5009
ESM7F5AM	USA	Female		17	HMP2	CD		17.6	7	NA	L1	26	E5009
ESM7F5CB	USA	Female		17	HMP2	CD		17.6	4	NA	L1	34	E5009
ESM7F5CD	USA	Female		17	HMP2	CD		17.6	3	NA	L1	36	E5009
ESM7F5CF	USA	Female		17	HMP2	CD		17.6	3	NA	L1	38	E5009
ESM5MEB9_P	USA	Female		15	HMP2	CD		22.4	1	NA	L1	0	E5013
ESM5MEB7	USA	Female		15	HMP2	CD		22.4	4	NA	L1	1	E5013
ESM5MED2	USA	Female		15	HMP2	CD		22.4	6	NA	L1	3	E5013
ESM718V8	USA	Female		15	HMP2	CD		22.4	0	NA	L1	7	E5013
ESM718V4	USA	Female		15	HMP2	CD		22.4	11	NA	L1	9	E5013
ESM718TK	USA	Female		15	HMP2	CD		22.4	10	NA	L1	14	E5013
ESM718TM	USA	Female		15	HMP2	CD		22.4	0	NA	L1	16	E5013
ESM718TF	USA	Female		15	HMP2	CD		22.4	8	NA	L1	21	E5013
ESM718T9	USA	Female		15	HMP2	CD		22.4	2	NA	L1	23	E5013
ESM718T7	USA	Female		15	HMP2	CD		22.4	0	NA	L1	26	E5013
ESM7F5C5	USA	Female		15	HMP2	CD		22.4	0	NA	L1	29	E5013
ESM7F5C7	USA	Female		15	HMP2	CD		22.4	0	NA	L1	31	E5013
ESM9IEP1	USA	Female		15	HMP2	CD		22.4	4	NA	L1	39	E5013
ESM7F5AE_P	USA	Male		8	HMP2	CD	NA		1	NA		0	E5019
ESM5MEC7_P	USA	Male		15	HMP2	UC	NA		NA	0		1	E5022
ESM718U9_P	USA	Female	NA		HMP2	CD	NA		4	NA		1	E5023
HSM5FZBQ_P	USA	Male		14	HMP2	CD		16.6	1	NA	L3+L4	5	H4001
HSM5FZBR_P	USA	Male		14	HMP2	CD		16.6	0	NA	L3+L4	7	H4001
HSM5FZBP_P	USA	Male		14	HMP2	CD		16.6	0	NA	L3+L4	10	H4001
HSM5MD7W_P	USA	Male		14	HMP2	CD		16.6	0	NA	L3+L4	11	H4001
HSM5MD5H_P	USA	Male		14	HMP2	CD		16.6	0	NA	L3+L4	13	H4001

HSM5MD5K_P	USA	Male	14	HMP2	CD	16.6	0	NA	L3+L4	15	H4001
HSM5FZC2_P	USA	Male	14	HMP2	CD	16.6	0	NA	L3+L4	17	H4001
HSM5FZBZ	USA	Male	14	HMP2	CD	16.6	2	NA	L3+L4	20	H4001
HSM5MD7J	USA	Male	14	HMP2	CD	16.6	0	NA	L3+L4	23	H4001
HSM5MD79	USA	Male	14	HMP2	CD	16.6	0	NA	L3+L4	33	H4001
HSM67VDZ	USA	Male	14	HMP2	CD	16.6	0	NA	L3+L4	36	H4001
HSM67VE4	USA	Male	14	HMP2	CD	16.6	1	NA	L3+L4	37	H4001
HSM5MD4U_P	USA	Male	14	HMP2	CD	18.6	5	NA	L3	2	H4004
HSM5MD4W_P	USA	Male	14	HMP2	CD	18.6	2	NA	L3	4	H4004
HSM5MD4Y	USA	Male	14	HMP2	CD	18.6	3	NA	L3	6	H4004
HSM5MD53	USA	Male	14	HMP2	CD	18.6	4	NA	L3	10	H4004
HSM5MD5P	USA	Male	14	HMP2	CD	18.6	0	NA	L3	16	H4004
HSM6XRSG	USA	Male	14	HMP2	CD	18.6	1	NA	L3	24	H4004
HSM6XRSI	USA	Male	14	HMP2	CD	18.6	0	NA	L3	26	H4004
HSM67VDP	USA	Male	14	HMP2	CD	18.6	3	NA	L3	34	H4004
HSM7CYZT	USA	Male	14	HMP2	CD	18.6	5	NA	L3	36	H4004
HSM7CYZV	USA	Male	14	HMP2	CD	18.6	4	NA	L3	38	H4004
HSM7CZ14	USA	Male	14	HMP2	CD	18.6	5	NA	L3	46	H4004
HSM5MD4B_P	USA	Male	8	HMP2	CD	26.6	3	NA	L2+L4	4	H4006
HSM5MD4A_P	USA	Male	8	HMP2	CD	26.6	0	NA	L2+L4	6	H4006
HSM5MD49_P	USA	Male	8	HMP2	CD	26.6	1	NA	L2+L4	8	H4006
HSM5MD48	USA	Male	8	HMP2	CD	26.6	2	NA	L2+L4	10	H4006
HSM5MD7K	USA	Male	8	HMP2	CD	26.6	2	NA	L2+L4	12	H4006
HSM5MD7M	USA	Male	8	HMP2	CD	26.6	7	NA	L2+L4	14	H4006
HSM5MD7O	USA	Male	8	HMP2	CD	26.6	8	NA	L2+L4	16	H4006
HSM5MD7Q	USA	Male	8	HMP2	CD	26.6	6	NA	L2+L4	18	H4006
HSM5MD7S	USA	Male	8	HMP2	CD	26.6	2	NA	L2+L4	20	H4006
HSM5MD7U	USA	Male	8	HMP2	CD	26.6	1	NA	L2+L4	22	H4006

HSM67VEC	USA	Male	8	HMP2	CD	26.6	0	NA	L2+L4	24	H4006
HSM67VEE	USA	Male	8	HMP2	CD	26.6	0	NA	L2+L4	26	H4006
HSM67VEG	USA	Male	8	HMP2	CD	26.6	4	NA	L2+L4	28	H4006
HSM67VEI	USA	Male	8	HMP2	CD	26.6	0	NA	L2+L4	32	H4006
HSM67VEK	USA	Male	8	HMP2	CD	26.6	0	NA	L2+L4	33	H4006
HSM67VEM	USA	Male	8	HMP2	CD	26.6	0	NA	L2+L4	34	H4006
HSM67VEM_TR	USA	Male	8	HMP2	CD	26.6	0	NA	L2+L4	34	H4006
HSM7CYX2	USA	Male	8	HMP2	CD	26.6	0	NA	L2+L4	36	H4006
HSM7CYX4	USA	Male	8	HMP2	CD	26.6	0	NA	L2+L4	38	H4006
HSM7CYX6	USA	Male	8	HMP2	CD	26.6	0	NA	L2+L4	40	H4006
HSM7CYX8	USA	Male	8	HMP2	CD	26.6	1	NA	L2+L4	42	H4006
HSM7CYXA	USA	Male	8	HMP2	CD	26.6	0	NA	L2+L4	44	H4006
HSM7CYXC	USA	Male	8	HMP2	CD	26.6	1	NA	L2+L4	46	H4006
HSM5MD7Z_P	USA	Female	15	HMP2	CD	20.4	3	NA	L3+L4	0	H4007
HSM5MD4P_P	USA	Female	15	HMP2	CD	20.4	4	NA	L3+L4	6	H4007
HSM5MD4O	USA	Female	15	HMP2	CD	20.4	2	NA	L3+L4	9	H4007
HSM5MD4N	USA	Female	15	HMP2	CD	20.4	2	NA	L3+L4	10	H4007
HSM6XRSN	USA	Female	15	HMP2	CD	20.4	1	NA	L3+L4	16	H4007
HSM6XRST	USA	Female	15	HMP2	CD	20.4	1	NA	L3+L4	22	H4007
HSM67VHQ	USA	Female	15	HMP2	CD	20.4	1	NA	L3+L4	24	H4007
HSM67VHS	USA	Female	15	HMP2	CD	20.4	1	NA	L3+L4	26	H4007
HSM67VHW	USA	Female	15	HMP2	CD	20.4	1	NA	L3+L4	31	H4007
HSM67VI1	USA	Female	15	HMP2	CD	20.4	1	NA	L3+L4	34	H4007
HSM7CYXQ	USA	Female	15	HMP2	CD	20.4	1	NA	L3+L4	37	H4007
HSM7CYXS	USA	Female	15	HMP2	CD	20.4	1	NA	L3+L4	38	H4007
HSM5MD8A_P	USA	Female	13	HMP2	nonIBD	35.6	NA	NA		0	H4008
HSM5MD57_P	USA	Female	13	HMP2	nonIBD	35.6	NA	NA		2	H4008
HSM5MD59_P	USA	Female	13	HMP2	nonIBD	35.6	NA	NA		4	H4008

HSM5MD5B_P	USA	Female	13	HMP2	nonIBD	35.6	NA	NA	6	H4008
HSM5MD5B	USA	Female	13	HMP2	nonIBD	35.6	NA	NA	6	H4008
HSM5MD5D_P	USA	Female	13	HMP2	nonIBD	35.6	NA	NA	8	H4008
HSM5MD5D	USA	Female	13	HMP2	nonIBD	35.6	NA	NA	8	H4008
HSM5MD5F_P	USA	Female	13	HMP2	nonIBD	35.6	NA	NA	10	H4008
HSM6XRSX_P	USA	Female	13	HMP2	nonIBD	35.6	NA	NA	11	H4008
HSM6XRSX	USA	Female	13	HMP2	nonIBD	35.6	NA	NA	11	H4008
HSM6XRT6_P	USA	Female	13	HMP2	nonIBD	35.6	NA	NA	14	H4008
HSM6XRSZ_P	USA	Female	13	HMP2	nonIBD	35.6	NA	NA	16	H4008
HSM6XRT4_P	USA	Female	13	HMP2	nonIBD	35.6	NA	NA	18	H4008
HSM6XRT2_P	USA	Female	13	HMP2	nonIBD	35.6	NA	NA	20	H4008
HSM67VFX_P	USA	Female	13	HMP2	nonIBD	35.6	NA	NA	24	H4008
HSM67VFX	USA	Female	13	HMP2	nonIBD	35.6	NA	NA	24	H4008
HSM67VFZ	USA	Female	13	HMP2	nonIBD	35.6	NA	NA	26	H4008
HSM67VG2_P	USA	Female	13	HMP2	nonIBD	35.6	NA	NA	28	H4008
HSM67VG6_P	USA	Female	13	HMP2	nonIBD	35.6	NA	NA	32	H4008
HSM67VG8	USA	Female	13	HMP2	nonIBD	35.6	NA	NA	34	H4008
CSM7CZ2F_P	USA	Female	13	HMP2	nonIBD	35.6	NA	NA	36	H4008
HSM7CZ2H	USA	Female	13	HMP2	nonIBD	35.6	NA	NA	38	H4008
HSM7CZ2J_P	USA	Female	13	HMP2	nonIBD	35.6	NA	NA	40	H4008
HSM7CZ2L_P	USA	Female	13	HMP2	nonIBD	35.6	NA	NA	42	H4008
HSM5MD82_P	USA	Female	6	HMP2	nonIBD	14	NA	NA	0	H4009
HSM5MD8P_P	USA	Female	6	HMP2	nonIBD	14	NA	NA	1	H4009
HSM5MD8N_P	USA	Female	6	HMP2	nonIBD	14	NA	NA	3	H4009
HSM5MD8L_P	USA	Female	6	HMP2	nonIBD	14	NA	NA	5	H4009
HSM5MD8B_P	USA	Female	6	HMP2	nonIBD	14	NA	NA	7	H4009
HSM5MD8D_P	USA	Female	6	HMP2	nonIBD	14	NA	NA	9	H4009
HSM6XRT8	USA	Female	6	HMP2	nonIBD	14	NA	NA	11	H4009

HSM6XRTA_P	USA	Female	6	HMP2	nonIBD	14	NA	NA	13	H4009
HSM6XRTC_P	USA	Female	6	HMP2	nonIBD	14	NA	NA	15	H4009
HSM6XRTE_P	USA	Female	6	HMP2	nonIBD	14	NA	NA	17	H4009
HSM6XRTG_P	USA	Female	6	HMP2	nonIBD	14	NA	NA	19	H4009
HSM6XRTG	USA	Female	6	HMP2	nonIBD	14	NA	NA	19	H4009
HSM67VGA_P	USA	Female	6	HMP2	nonIBD	14	NA	NA	23	H4009
HSM67VGA	USA	Female	6	HMP2	nonIBD	14	NA	NA	23	H4009
HSM67VGC	USA	Female	6	HMP2	nonIBD	14	NA	NA	27	H4009
HSM67VGE_P	USA	Female	6	HMP2	nonIBD	14	NA	NA	29	H4009
HSM67VGG	USA	Female	6	HMP2	nonIBD	14	NA	NA	31	H4009
HSM67VGI_P	USA	Female	6	HMP2	nonIBD	14	NA	NA	33	H4009
HSM67VGK	USA	Female	6	HMP2	nonIBD	14	NA	NA	35	H4009
HSM67VGK_TR	USA	Female	6	HMP2	nonIBD	14	NA	NA	35	H4009
HSM7CYYR_P	USA	Female	6	HMP2	nonIBD	14	NA	NA	37	H4009
HSM7CYYV_P	USA	Female	6	HMP2	nonIBD	14	NA	NA	41	H4009
HSM5MD87_P	USA	Male	13	HMP2	UC	22	NA	1	0	H4010
HSM5MD47_P	USA	Male	13	HMP2	UC	22	NA	1	2	H4010
HSM5MD44_P	USA	Male	13	HMP2	UC	22	NA	0	4	H4010
HSM5MD43_P	USA	Male	13	HMP2	UC	22	NA	0	6	H4010
HSM5MD43	USA	Male	13	HMP2	UC	22	NA	0	6	H4010
HSM5MD3Y	USA	Male	13	HMP2	UC	22	NA	0	11	H4010
HSM5MD41	USA	Male	13	HMP2	UC	22	NA	0	14	H4010
HSM6XRTM	USA	Male	13	HMP2	UC	22	NA	3	22	H4010
HSM6XRTQ	USA	Male	13	HMP2	UC	22	NA	0	26	H4010
HSM6XRTO	USA	Male	13	HMP2	UC	22	NA	0	33	H4010
HSM6XRTS	USA	Male	13	HMP2	UC	22	NA	0	35	H4010
HSM67VGY	USA	Male	13	HMP2	UC	22	NA	0	36	H4010
HSM67VH1	USA	Male	13	HMP2	UC	22	NA	1	47	H4010

HSM5MD8J_P	USA	Female	7	HMP2	CD	NA		4	NA	L3+L4	0	H4011
HSM5MD8F_P	USA	Female	16	HMP2	CD	NA		5	NA	L3	0	H4012
HSM5MD8H_P	USA	Male	8	HMP2	nonIBD		16.1	NA	NA		0	H4013
HSM5MD6E_P	USA	Male	8	HMP2	nonIBD		16.1	NA	NA		2	H4013
HSM5MD6I_P	USA	Male	8	HMP2	nonIBD		16.1	NA	NA		5	H4013
HSM5MD6I	USA	Male	8	HMP2	nonIBD		16.1	NA	NA		5	H4013
HSM5MD6K_P	USA	Male	8	HMP2	nonIBD		16.1	NA	NA		7	H4013
HSM5MD6K	USA	Male	8	HMP2	nonIBD		16.1	NA	NA		7	H4013
HSM5MD6M	USA	Male	8	HMP2	nonIBD		16.1	NA	NA		9	H4013
HSM6XRRJ	USA	Male	8	HMP2	nonIBD		16.1	NA	NA		15	H4013
HSM6XRVA	USA	Male	8	HMP2	nonIBD		16.1	NA	NA		24	H4013
HSM6XRVC	USA	Male	8	HMP2	nonIBD		16.1	NA	NA		25	H4013
HSM6XRVC_TR	USA	Male	8	HMP2	nonIBD		16.1	NA	NA		25	H4013
HSM6XRVK	USA	Male	8	HMP2	nonIBD		16.1	NA	NA		31	H4013
HSM7J4QZ	USA	Male	8	HMP2	nonIBD		16.1	NA	NA		36	H4013
HSM7J4R2	USA	Male	8	HMP2	nonIBD		16.1	NA	NA		41	H4013
HSM5MD3L_P	USA	Female	10	HMP2	CD		19.6	0	NA	L3+L4	0	H4014
HSM5MD66	USA	Female	10	HMP2	CD		19.6	2	NA	L3+L4	4	H4014
HSM5MD6A	USA	Female	10	HMP2	CD		19.6	1	NA	L3+L4	8	H4014
HSM5MD6A_TR	USA	Female	10	HMP2	CD		19.6	1	NA	L3+L4	8	H4014
HSM5MD6C	USA	Female	10	HMP2	CD		19.6	1	NA	L3+L4	10	H4014
HSM6XRRV	USA	Female	10	HMP2	CD		19.6	3	NA	L3+L4	16	H4014
HSM6XRS2	USA	Female	10	HMP2	CD		19.6	2	NA	L3+L4	23	H4014
HSM6XRVM	USA	Female	10	HMP2	CD		19.6	0	NA	L3+L4	25	H4014
HSM6XRVO	USA	Female	10	HMP2	CD		19.6	0	NA	L3+L4	27	H4014
HSM6XRVU	USA	Female	10	HMP2	CD		19.6	0	NA	L3+L4	36	H4014
HSM6XRVW	USA	Female	10	HMP2	CD		19.6	3	NA	L3+L4	38	H4014
HSM7J4QT	USA	Female	10	HMP2	CD		19.6	0	NA	L3+L4	50	H4014

HSM5MD5X_P	USA	Male	15	HMP2	CD	19.2	8	NA	L3+L4	0	H4015
HSM5MD62	USA	Male	15	HMP2	CD	19.2	1	NA	L3+L4	2	H4015
HSM5MD6Y	USA	Male	15	HMP2	CD	19.2	4	NA	L3+L4	4	H4015
HSM5MD71	USA	Male	15	HMP2	CD	19.2	1	NA	L3+L4	6	H4015
HSM5MD73	USA	Male	15	HMP2	CD	19.2	5	NA	L3+L4	8	H4015
HSM5MD75	USA	Male	15	HMP2	CD	19.2	3	NA	L3+L4	11	H4015
HSM6XRS4	USA	Male	15	HMP2	CD	19.2	2	NA	L3+L4	12	H4015
HSM6XRS6	USA	Male	15	HMP2	CD	19.2	2	NA	L3+L4	14	H4015
HSM6XRS8	USA	Male	15	HMP2	CD	19.2	3	NA	L3+L4	16	H4015
HSM6XRSE	USA	Male	15	HMP2	CD	19.2	1	NA	L3+L4	22	H4015
HSM7CYZ5	USA	Male	15	HMP2	CD	19.2	1	NA	L3+L4	24	H4015
HSM7CYZ7	USA	Male	15	HMP2	CD	19.2	2	NA	L3+L4	26	H4015
HSM7CYZ9	USA	Male	15	HMP2	CD	19.2	0	NA	L3+L4	28	H4015
HSM7CYZB	USA	Male	15	HMP2	CD	19.2	0	NA	L3+L4	30	H4015
HSM7CYZD	USA	Male	15	HMP2	CD	19.2	1	NA	L3+L4	32	H4015
HSM7CYZF	USA	Male	15	HMP2	CD	19.2	1	NA	L3+L4	34	H4015
HSM7J4QB	USA	Male	15	HMP2	CD	19.2	0	NA	L3+L4	36	H4015
HSM7J4QD	USA	Male	15	HMP2	CD	19.2	3	NA	L3+L4	38	H4015
HSM7J4QF	USA	Male	15	HMP2	CD	19.2	NA	NA	L3+L4	40	H4015
HSM7J4QH	USA	Male	15	HMP2	CD	19.2	NA	NA	L3+L4	42	H4015
HSM7J4QJ	USA	Male	15	HMP2	CD	19.2	NA	NA	L3+L4	44	H4015
HSM7J4QL	USA	Male	15	HMP2	CD	19.2	5	NA	L3+L4	46	H4015
HSM5MD5Z_P	USA	Female	10	HMP2	nonIBD	13.5	NA	NA		0	H4016
HSM5MD6O_P	USA	Female	10	HMP2	nonIBD	13.5	NA	NA		2	H4016
HSM5MD6Q	USA	Female	10	HMP2	nonIBD	13.5	NA	NA		5	H4016
HSM5MD6S_P	USA	Female	10	HMP2	nonIBD	13.5	NA	NA		5	H4016
HSM5MD6U_P	USA	Female	10	HMP2	nonIBD	13.5	NA	NA		8	H4016
HSM5MD6W	USA	Female	10	HMP2	nonIBD	13.5	NA	NA		10	H4016

HSM67VF3	USA	Female	10	HMP2	nonIBD	13.5	NA	NA		15	H4016
HSM7CYXE	USA	Female	10	HMP2	nonIBD	13.5	NA	NA		24	H4016
HSM7CYXG	USA	Female	10	HMP2	nonIBD	13.5	NA	NA		26	H4016
HSM7CYXI	USA	Female	10	HMP2	nonIBD	13.5	NA	NA		27	H4016
HSM7CYXO	USA	Female	10	HMP2	nonIBD	13.5	NA	NA		34	H4016
HSM7J4I3	USA	Female	10	HMP2	nonIBD	13.5	NA	NA		35	H4016
HSM7J4KO	USA	Female	10	HMP2	nonIBD	13.5	NA	NA		38	H4016
HSM7J4KQ	USA	Female	10	HMP2	nonIBD	13.5	NA	NA		47	H4016
HSM6XRQB_P	USA	Female	16	HMP2	CD	28.6	6	NA	L3+L4	0	H4017
HSM6XRQB	USA	Female	16	HMP2	CD	28.6	6	NA	L3+L4	0	H4017
HSM67VHK	USA	Female	16	HMP2	CD	28.6	14	NA	L3+L4	2	H4017
HSM6XRQI	USA	Female	16	HMP2	CD	28.6	3	NA	L3+L4	4	H4017
HSM6XRQK	USA	Female	16	HMP2	CD	28.6	4	NA	L3+L4	5	H4017
HSM6XRQM	USA	Female	16	HMP2	CD	28.6	2	NA	L3+L4	9	H4017
HSM6XRQO	USA	Female	16	HMP2	CD	28.6	4	NA	L3+L4	11	H4017
HSM67VF9	USA	Female	16	HMP2	CD	28.6	9	NA	L3+L4	13	H4017
HSM67VFD	USA	Female	16	HMP2	CD	28.6	6	NA	L3+L4	19	H4017
HSM67VFF	USA	Female	16	HMP2	CD	28.6	6	NA	L3+L4	21	H4017
HSM67VFH	USA	Female	16	HMP2	CD	28.6	7	NA	L3+L4	23	H4017
HSM67VFJ	USA	Female	16	HMP2	CD	28.6	3	NA	L3+L4	25	H4017
HSM7CYY3	USA	Female	16	HMP2	CD	28.6	4	NA	L3+L4	26	H4017
HSM7CYY5	USA	Female	16	HMP2	CD	28.6	5	NA	L3+L4	29	H4017
HSM7CYY7	USA	Female	16	HMP2	CD	28.6	4	NA	L3+L4	30	H4017
HSM7CYYD	USA	Female	16	HMP2	CD	28.6	9	NA	L3+L4	34	H4017
HSM7CYY9	USA	Female	16	HMP2	CD	28.6	3	NA	L3+L4	36	H4017
HSM7CYYB	USA	Female	16	HMP2	CD	28.6	13	NA	L3+L4	37	H4017
HSM6XRQC_P	USA	Female	13	HMP2	nonIBD	25.2	NA	NA		0	H4018
HSM6XRQS	USA	Female	13	HMP2	nonIBD	25.2	NA	NA		4	H4018

HSM6XRQU	USA	Female	13	HMP2	nonIBD	25.2	NA	NA	6	H4018
HSM6XRQW	USA	Female	13	HMP2	nonIBD	25.2	NA	NA	8	H4018
HSM6XRQY	USA	Female	13	HMP2	nonIBD	25.2	NA	NA	10	H4018
HSM67VFR	USA	Female	13	HMP2	nonIBD	25.2	NA	NA	18	H4018
HSM7CYYF	USA	Female	13	HMP2	nonIBD	25.2	NA	NA	24	H4018
HSM7CYYH	USA	Female	13	HMP2	nonIBD	25.2	NA	NA	26	H4018
HSM7CYYP	USA	Female	13	HMP2	nonIBD	25.2	NA	NA	34	H4018
HSM7J4LD	USA	Female	13	HMP2	nonIBD	25.2	NA	NA	36	H4018
HSM7J4LF	USA	Female	13	HMP2	nonIBD	25.2	NA	NA	38	H4018
HSM7J4LH	USA	Female	13	HMP2	nonIBD	25.2	NA	NA	40	H4018
HSM7J4LN	USA	Female	13	HMP2	nonIBD	25.2	NA	NA	46	H4018
HSM6XRQE_P	USA	Female	11	HMP2	UC	16.9	NA	4	0	H4019
HSM6XRR5	USA	Female	11	HMP2	UC	16.9	NA	4	2	H4019
HSM6XRR7	USA	Female	11	HMP2	UC	16.9	NA	1	4	H4019
HSM6XRR9	USA	Female	11	HMP2	UC	16.9	NA	2	6	H4019
HSM6XRRB	USA	Female	11	HMP2	UC	16.9	NA	3	9	H4019
HSM6XRRD	USA	Female	11	HMP2	UC	16.9	NA	1	10	H4019
HSM6XRUX	USA	Female	11	HMP2	UC	16.9	NA	1	12	H4019
HSM6XRUZ	USA	Female	11	HMP2	UC	16.9	NA	1	14	H4019
HSM6XRV2	USA	Female	11	HMP2	UC	16.9	NA	1	16	H4019
HSM6XRV4	USA	Female	11	HMP2	UC	16.9	NA	2	18	H4019
HSM6XRV6	USA	Female	11	HMP2	UC	16.9	NA	1	20	H4019
HSM6XRV8	USA	Female	11	HMP2	UC	16.9	NA	1	22	H4019
HSM7J4PC	USA	Female	11	HMP2	UC	16.9	NA	0	24	H4019
HSM7J4PE	USA	Female	11	HMP2	UC	16.9	NA	0	28	H4019
HSM7J4PG	USA	Female	11	HMP2	UC	16.9	NA	0	29	H4019
HSM7J4PI	USA	Female	11	HMP2	UC	16.9	NA	0	31	H4019
HSM7J4PK	USA	Female	11	HMP2	UC	16.9	NA	1	33	H4019

HSM7J4PM	USA	Female	11	HMP2	UC	16.9	NA	2		36	H4019
HSM7J4M4	USA	Female	11	HMP2	UC	16.9	NA	1		37	H4019
HSM7J4M6	USA	Female	11	HMP2	UC	16.9	NA	2		38	H4019
HSM7J4M8	USA	Female	11	HMP2	UC	16.9	NA	2		40	H4019
HSM7J4MA	USA	Female	11	HMP2	UC	16.9	NA	2		43	H4019
HSM7J4MC	USA	Female	11	HMP2	UC	16.9	NA	2		45	H4019
HSM7J4ME	USA	Female	11	HMP2	UC	16.9	NA	2		46	H4019
HSM6XRR3	USA	Male	13	HMP2	CD	27.7	3	NA	L1+L4	0	H4020
HSM67VCX_P	USA	Male	13	HMP2	CD	27.7	2	NA	L1+L4	2	H4020
HSM67VCZ	USA	Male	13	HMP2	CD	27.7	0	NA	L1+L4	4	H4020
HSM67VD2	USA	Male	13	HMP2	CD	27.7	2	NA	L1+L4	6	H4020
HSM67VD4	USA	Male	13	HMP2	CD	27.7	0	NA	L1+L4	8	H4020
HSM67VD6	USA	Male	13	HMP2	CD	27.7	1	NA	L1+L4	10	H4020
HSM67VHB	USA	Male	13	HMP2	CD	27.7	1	NA	L1+L4	13	H4020
HSM67VHD	USA	Male	13	HMP2	CD	27.7	2	NA	L1+L4	14	H4020
HSM67VHF	USA	Male	13	HMP2	CD	27.7	1	NA	L1+L4	16	H4020
HSM67VHH	USA	Male	13	HMP2	CD	27.7	4	NA	L1+L4	18	H4020
HSM67VHJ	USA	Male	13	HMP2	CD	27.7	2	NA	L1+L4	20	H4020
HSM6XRUV	USA	Male	13	HMP2	CD	27.7	2	NA	L1+L4	22	H4020
HSM7J4PO	USA	Male	13	HMP2	CD	27.7	2	NA	L1+L4	24	H4020
HSM7J4PQ	USA	Male	13	HMP2	CD	27.7	5	NA	L1+L4	26	H4020
HSM7J4PS	USA	Male	13	HMP2	CD	27.7	2	NA	L1+L4	28	H4020
HSM7J4PU	USA	Male	13	HMP2	CD	27.7	1	NA	L1+L4	30	H4020
HSM7J4PW	USA	Male	13	HMP2	CD	27.7	0.5	NA	L1+L4	32	H4020
HSM7J4PY	USA	Male	13	HMP2	CD	27.7	1	NA	L1+L4	35	H4020
HSM7J4IO	USA	Male	13	HMP2	CD	27.7	2	NA	L1+L4	37	H4020
HSM7J4IP	USA	Male	13	HMP2	CD	27.7	1	NA	L1+L4	38	H4020
HSM7J4IQ	USA	Male	13	HMP2	CD	27.7	1	NA	L1+L4	40	H4020

HSM7J4IR	USA	Male	13	HMP2	CD	27.7	2	NA	L1+L4	42	H4020
HSM7J4IS	USA	Male	13	HMP2	CD	27.7	2	NA	L1+L4	44	H4020
HSM67VDX_P	USA	Female	9	HMP2	nonIBD	18.9	NA	NA		0	H4022
HSM67VEO	USA	Female	9	HMP2	nonIBD	18.9	NA	NA		2	H4022
HSM67VEQ	USA	Female	9	HMP2	nonIBD	18.9	NA	NA		4	H4022
HSM67VES	USA	Female	9	HMP2	nonIBD	18.9	NA	NA		6	H4022
HSM67VEU	USA	Female	9	HMP2	nonIBD	18.9	NA	NA		8	H4022
HSM67VEW	USA	Female	9	HMP2	nonIBD	18.9	NA	NA		10	H4022
HSM7CYZJ	USA	Female	9	HMP2	nonIBD	18.9	NA	NA		14	H4022
HSM7CYZL	USA	Female	9	HMP2	nonIBD	18.9	NA	NA		16	H4022
HSM7CYZR	USA	Female	9	HMP2	nonIBD	18.9	NA	NA		22	H4022
HSM7J4RE	USA	Female	9	HMP2	nonIBD	18.9	NA	NA		26	H4022
HSM7J4G1	USA	Female	9	HMP2	nonIBD	18.9	NA	NA		28	H4022
HSM7J4G8	USA	Female	9	HMP2	nonIBD	18.9	NA	NA		34	H4022
HSM7J4IU	USA	Female	9	HMP2	nonIBD	18.9	NA	NA		36	H4022
HSM7J4IW	USA	Female	9	HMP2	nonIBD	18.9	NA	NA		38	H4022
HSM67VDR_P	USA	Male	16	HMP2	nonIBD	18.8	NA	NA		0	H4023
HSM6XRUL	USA	Male	16	HMP2	nonIBD	18.8	NA	NA		1	H4023
HSM6XRUN	USA	Male	16	HMP2	nonIBD	18.8	NA	NA		3	H4023
HSM6XRUR	USA	Male	16	HMP2	nonIBD	18.8	NA	NA		7	H4023
HSM6XRQ8	USA	Male	16	HMP2	nonIBD	18.8	NA	NA		9	H4023
HSM7CZ16	USA	Male	16	HMP2	nonIBD	18.8	NA	NA		11	H4023
HSM7CZ18	USA	Male	16	HMP2	nonIBD	18.8	NA	NA		13	H4023
HSM7CZ1A	USA	Male	16	HMP2	nonIBD	18.8	NA	NA		15	H4023
HSM7CZ1C	USA	Male	16	HMP2	nonIBD	18.8	NA	NA		17	H4023
HSM7CZ1E	USA	Male	16	HMP2	nonIBD	18.8	NA	NA		19	H4023
HSM7CZ1G	USA	Male	16	HMP2	nonIBD	18.8	NA	NA		21	H4023
HSM7J4HA	USA	Male	16	HMP2	nonIBD	18.8	NA	NA		23	H4023

HSM7J4HC	USA	Male	16	HMP2	nonIBD	18.8	NA	NA	24	H4023
HSM7J4HE	USA	Male	16	HMP2	nonIBD	18.8	NA	NA	25	H4023
HSM7J4HG	USA	Male	16	HMP2	nonIBD	18.8	NA	NA	29	H4023
HSM7J4HI	USA	Male	16	HMP2	nonIBD	18.8	NA	NA	31	H4023
HSM7J4HK	USA	Male	16	HMP2	nonIBD	18.8	NA	NA	33	H4023
HSM7J4KC	USA	Male	16	HMP2	nonIBD	18.8	NA	NA	35	H4023
HSM7J4KG	USA	Male	16	HMP2	nonIBD	18.8	NA	NA	39	H4023
HSM7J4KI	USA	Male	16	HMP2	nonIBD	18.8	NA	NA	41	H4023
HSM7J4KK	USA	Male	16	HMP2	nonIBD	18.8	NA	NA	43	H4023
HSM7J4KM	USA	Male	16	HMP2	nonIBD	18.8	NA	NA	45	H4023
HSM67VDT_P	USA	Male	11	HMP2	nonIBD	15.4	NA	NA	0	H4024
HSM67VDT	USA	Male	11	HMP2	nonIBD	15.4	NA	NA	0	H4024
HSM67VI3	USA	Male	11	HMP2	nonIBD	15.4	NA	NA	2	H4024
HSM67VI5	USA	Male	11	HMP2	nonIBD	15.4	NA	NA	3	H4024
HSM67VI7	USA	Male	11	HMP2	nonIBD	15.4	NA	NA	6	H4024
HSM67VI9	USA	Male	11	HMP2	nonIBD	15.4	NA	NA	7	H4024
HSM67VIB	USA	Male	11	HMP2	nonIBD	15.4	NA	NA	9	H4024
HSM7CZ24	USA	Male	11	HMP2	nonIBD	15.4	NA	NA	12	H4024
HSM7CZ26	USA	Male	11	HMP2	nonIBD	15.4	NA	NA	14	H4024
HSM7CZ28	USA	Male	11	HMP2	nonIBD	15.4	NA	NA	15	H4024
HSM7CZ2A	USA	Male	11	HMP2	nonIBD	15.4	NA	NA	18	H4024
HSM7CZ2E	USA	Male	11	HMP2	nonIBD	15.4	NA	NA	21	H4024
HSM7J4HW	USA	Male	11	HMP2	nonIBD	15.4	NA	NA	24	H4024
HSM7J4HY	USA	Male	11	HMP2	nonIBD	15.4	NA	NA	25	H4024
HSM7J4I5	USA	Male	11	HMP2	nonIBD	15.4	NA	NA	29	H4024
HSM7J4I7	USA	Male	11	HMP2	nonIBD	15.4	NA	NA	32	H4024
HSM7J4I9	USA	Male	11	HMP2	nonIBD	15.4	NA	NA	34	H4024
HSM7J4O1	USA	Male	11	HMP2	nonIBD	15.4	NA	NA	38	H4024

HSM7J4NY	USA	Male	11	HMP2	nonIBD	15.4	NA	NA		39	H4024
HSM7J4O3	USA	Male	11	HMP2	nonIBD	15.4	NA	NA		40	H4024
HSM7J4O5	USA	Male	11	HMP2	nonIBD	15.4	NA	NA		42	H4024
HSM7J4O7	USA	Male	11	HMP2	nonIBD	15.4	NA	NA		44	H4024
HSM7J4O9	USA	Male	11	HMP2	nonIBD	15.4	NA	NA		46	H4024
HSM7CZ1T_P	USA	Male	15	HMP2	UC	14.9	0	NA	L2+L4	2	H4027
HSM7CZ1V	USA	Male	15	HMP2	UC	14.9	0	NA	L2+L4	3	H4027
HSM7CZ1Z	USA	Male	15	HMP2	UC	14.9	1	NA	L2+L4	7	H4027
HSM7CZ22	USA	Male	15	HMP2	UC	14.9	3	NA	L2+L4	10	H4027
HSM7J4GP	USA	Male	15	HMP2	UC	14.9	NA	NA	L2+L4	15	H4027
HSM7J4GR	USA	Male	15	HMP2	UC	14.9	NA	3	L2+L4	17	H4027
HSM7J4NC	USA	Male	15	HMP2	UC	14.9	NA	5	L2+L4	24	H4027
HSM7J4NE	USA	Male	15	HMP2	UC	14.9	NA	2	L2+L4	25	H4027
HSM7J4NM	USA	Male	15	HMP2	UC	14.9	NA	0	L2+L4	33	H4027
HSMA33O1	USA	Male	15	HMP2	UC	14.9	NA	1	L2+L4	35	H4027
HSMA33O3	USA	Male	15	HMP2	UC	14.9	NA	2	L2+L4	37	H4027
HSM67VID	USA	Female	13	HMP2	CD	15.9	0	NA	L1+L4	1	H4028
HSM67VIF	USA	Female	13	HMP2	CD	15.9	0	NA	L1+L4	3	H4028
HSM67VIJ_P	USA	Female	13	HMP2	CD	15.9	0	NA	L1+L4	8	H4028
HSM67VIL	USA	Female	13	HMP2	CD	15.9	2	NA	L1+L4	9	H4028
HSM7J4GD	USA	Female	13	HMP2	CD	15.9	1	NA	L1+L4	15	H4028
HSM7J4MY	USA	Female	13	HMP2	CD	15.9	2	NA	L1+L4	24	H4028
HSM7J4N4	USA	Female	13	HMP2	CD	15.9	1	NA	L1+L4	28	H4028
HSM7J4N6	USA	Female	13	HMP2	CD	15.9	0	NA	L1+L4	32	H4028
HSM7J4N8	USA	Female	13	HMP2	CD	15.9	NA	NA	L1+L4	33	H4028
HSM7J4NA	USA	Female	13	HMP2	CD	15.9	0	NA	L1+L4	34	H4028
HSM7CYWS_P	USA	Male	16	HMP2	CD	21.1	5	NA	L1+L4	0	H4030
HSM7CZ2Z	USA	Male	16	HMP2	CD	21.1	0	NA	L1+L4	1	H4030

HSM7CZ32	USA	Male	16	HMP2	CD		21.1	0	NA	L1+L4	4	H4030
HSM7CZ36	USA	Male	16	HMP2	CD		21.1	2	NA	L1+L4	8	H4030
HSM7CZ38	USA	Male	16	HMP2	CD		21.1	NA	NA	L1+L4	9	H4030
HSM7J4L5	USA	Male	16	HMP2	CD		21.1	NA	NA	L1+L4	16	H4030
HSM7J4L9	USA	Male	16	HMP2	CD		21.1	NA	NA	L1+L4	19	H4030
HSM7J4ON	USA	Male	16	HMP2	CD		21.1	0	NA	L1+L4	24	H4030
HSM7J4OP	USA	Male	16	HMP2	CD		21.1	NA	NA	L1+L4	26	H4030
HSM7J4OT	USA	Male	16	HMP2	CD		21.1	NA	NA	L1+L4	31	H4030
HSM7J4OV	USA	Male	16	HMP2	CD		21.1	NA	NA	L1+L4	31	H4030
HSM7J4OX	USA	Male	16	HMP2	CD		21.1	NA	NA	L1+L4	35	H4030
HSMA33JZ	USA	Male	16	HMP2	CD		21.1	NA	NA	L1+L4	37	H4030
HSM7CZ2V_P	USA	Male	12	HMP2	CD	NA		4	NA	L1+L4	0	H4031
HSM7CZ3A	USA	Male	12	HMP2	CD	NA		4	NA	L1+L4	2	H4031
HSM7CZ3C	USA	Male	12	HMP2	CD	NA		1	NA	L1+L4	4	H4031
HSM7CZ3E	USA	Male	12	HMP2	CD	NA		1	NA	L1+L4	6	H4031
HSM7CZ3G	USA	Male	12	HMP2	CD	NA		2	NA	L1+L4	8	H4031
HSM7J4PA	USA	Male	12	HMP2	CD	NA		4	NA	L1+L4	10	H4031
HSM7J4MK	USA	Male	12	HMP2	CD	NA		NA	NA	L1+L4	16	H4031
HSM7J4OB	USA	Male	12	HMP2	CD	NA		1	NA	L1+L4	24	H4031
HSM7J4OE	USA	Male	12	HMP2	CD	NA		3	NA	L1+L4	26	H4031
HSM7J4OL	USA	Male	12	HMP2	CD	NA		6	NA	L1+L4	35	H4031
HSMA33JB	USA	Male	12	HMP2	CD	NA		0	NA	L1+L4	36	H4031
HSMA33JD	USA	Male	12	HMP2	CD	NA		1	NA	L1+L4	38	H4031
HSM7J4Q1	USA	Male	12	HMP2	CD		14.9	1	NA	L3+L4	1	H4032
HSM7J4Q3_P	USA	Male	12	HMP2	CD		14.9	0	NA	L3+L4	4	H4032
HSM7J4Q7	USA	Male	12	HMP2	CD		14.9	1	NA	L3+L4	7	H4032
HSM7J4Q9	USA	Male	12	HMP2	CD		14.9	1	NA	L3+L4	10	H4032
HSM7J4MS	USA	Male	12	HMP2	CD		14.9	0	NA	L3+L4	12	H4032

HSM7J4MW	USA	Male	12	HMP2	CD	14.9	1	NA	L3+L4	16	H4032
HSM7J4IC	USA	Male	12	HMP2	CD	14.9	0	NA	L3+L4	18	H4032
HSM7J4OZ	USA	Male	12	HMP2	CD	14.9	0	NA	L3+L4	23	H4032
HSM7J4P2	USA	Male	12	HMP2	CD	14.9	0	NA	L3+L4	27	H4032
HSMA33MV	USA	Male	12	HMP2	CD	14.9	NA	NA	L3+L4	34	H4032
HSMA33JN	USA	Male	12	HMP2	CD	14.9	NA	NA	L3+L4	35	H4032
HSMA33JP	USA	Male	12	HMP2	CD	14.9	NA	NA	L3+L4	37	H4032
HSMA33JR	USA	Male	12	HMP2	CD	14.9	1	NA	L3+L4	40	H4032
HSM7CZ2X_P	USA	Male	16	HMP2	UC	31	NA	3		0	H4035
HSM7J4HM	USA	Male	16	HMP2	UC	31	NA	1		2	H4035
HSM7J4HO	USA	Male	16	HMP2	UC	31	NA	2		4	H4035
HSM7J4HQ	USA	Male	16	HMP2	UC	31	NA	1		6	H4035
HSM7J4HS	USA	Male	16	HMP2	UC	31	NA	1		8	H4035
HSM7J4HU	USA	Male	16	HMP2	UC	31	NA	2		10	H4035
HSM7J4JZ	USA	Male	16	HMP2	UC	31	NA	1		12	H4035
HSM7J4K2	USA	Male	16	HMP2	UC	31	NA	1		15	H4035
HSM7J4K4	USA	Male	16	HMP2	UC	31	NA	2		16	H4035
HSM7J4K6	USA	Male	16	HMP2	UC	31	NA	3		18	H4035
HSM7J4K8	USA	Male	16	HMP2	UC	31	NA	2		20	H4035
HSM7J4KA	USA	Male	16	HMP2	UC	31	NA	3		22	H4035
HSMA33OP	USA	Male	16	HMP2	UC	31	NA	2		24	H4035
HSMA33OR	USA	Male	16	HMP2	UC	31	NA	5		26	H4035
HSMA33OT	USA	Male	16	HMP2	UC	31	NA	1		28	H4035
HSMA33OV	USA	Male	16	HMP2	UC	31	NA	0		30	H4035
HSMA33OX	USA	Male	16	HMP2	UC	31	NA	0		32	H4035
HSMA33OZ	USA	Male	16	HMP2	UC	31	NA	0		34	H4035
HSMA33MA	USA	Male	16	HMP2	UC	31	NA	1		37	H4035
HSMA33MC	USA	Male	16	HMP2	UC	31	NA	2		38	H4035

HSMA33ME	USA	Male	16	HMP2	UC	31	NA	1		40	H4035
HSMA33MG	USA	Male	16	HMP2	UC	31	NA	2		42	H4035
HSMA33MI	USA	Male	16	HMP2	UC	31	NA	1		44	H4035
HSMA33MK	USA	Male	16	HMP2	UC	31	NA	5		46	H4035
HSM7J4J7	USA	Male	15	HMP2	CD	17.3	3	NA	L3	2	H4038
HSM7J4J9	USA	Male	15	HMP2	CD	17.3	3	NA	L3	4	H4038
HSM7J4JD	USA	Male	15	HMP2	CD	17.3	0	NA	L3	8	H4038
HSM7J4JF	USA	Male	15	HMP2	CD	17.3	0	NA	L3	10	H4038
HSMA33NA	USA	Male	15	HMP2	CD	17.3	0	NA	L3	14	H4038
HSMA33NC	USA	Male	15	HMP2	CD	17.3	2	NA	L3	16	H4038
HSMA33NG_P	USA	Male	15	HMP2	CD	17.3	0	NA	L3	20	H4038
HSMA33KE	USA	Male	15	HMP2	CD	17.3	3	NA	L3	24	H4038
HSMA33KM	USA	Male	15	HMP2	CD	17.3	0	NA	L3	34	H4038
HSMA33KO	USA	Male	15	HMP2	CD	17.3	0	NA	L3	36	H4038
HSMA33PX	USA	Male	15	HMP2	CD	17.3	0	NA	L3	38	H4038
HSMA33PZ	USA	Male	15	HMP2	CD	17.3	4	NA	L3	41	H4038
HSMA33Q6	USA	Male	15	HMP2	CD	17.3	0	NA	L3	48	H4038
HSM7J4LP	USA	Female	12	HMP2	CD	16.9	3	NA	L3	0	H4039
HSM7J4JH	USA	Female	12	HMP2	CD	16.9	0	NA	L3	2	H4039
HSM7J4JJ	USA	Female	12	HMP2	CD	16.9	0	NA	L3	4	H4039
HSM7J4JN	USA	Female	12	HMP2	CD	16.9	NA	NA	L3	8	H4039
HSM7J4JP	USA	Female	12	HMP2	CD	16.9	0	NA	L3	10	H4039
HSMA33NO	USA	Female	12	HMP2	CD	16.9	NA	NA	L3	16	H4039
HSMA33NQ	USA	Female	12	HMP2	CD	16.9	NA	NA	L3	18	H4039
HSMA33KQ	USA	Female	12	HMP2	CD	16.9	NA	NA	L3	24	H4039
HSMA33KS	USA	Female	12	HMP2	CD	16.9	NA	NA	L3	26	H4039
HSMA33KU	USA	Female	12	HMP2	CD	16.9	NA	NA	L3	29	H4039
HSMA33L1	USA	Female	12	HMP2	CD	16.9	0	NA	L3	34	H4039

HSMA33PL	USA	Female	12	HMP2	CD	16.9	0	NA	L3	36	H4039
HSMA33PN	USA	Female	12	HMP2	CD	16.9	NA	NA	L3	38	H4039
HSM7J4JT_P	USA	Female	17	HMP2	UC	18.6	NA	0		0	H4040
HSM7J4NO	USA	Female	17	HMP2	UC	18.6	NA	1		2	H4040
HSM7J4NS	USA	Female	17	HMP2	UC	18.6	NA	0		7	H4040
HSM7J4NU	USA	Female	17	HMP2	UC	18.6	NA	0		9	H4040
HSMA33OD	USA	Female	17	HMP2	UC	18.6	NA	1		16	H4040
HSMA33OJ	USA	Female	17	HMP2	UC	18.6	NA	1		22	H4040
HSMA33OL	USA	Female	17	HMP2	UC	18.6	NA	3		26	H4040
HSMA33LX	USA	Female	17	HMP2	UC	18.6	NA	1		34	H4040
HSMA33LZ	USA	Female	17	HMP2	UC	18.6	NA	2		37	H4040
HSMA33M2	USA	Female	17	HMP2	UC	18.6	NA	2		38	H4040
HSMA33M8	USA	Female	17	HMP2	UC	18.6	NA	0		44	H4040
HSM7J4JV_P	USA	Male	15	HMP2	UC	30.7	NA	6		0	H4042
HSMA33MX	USA	Male	15	HMP2	UC	30.7	NA	7		2	H4042
HSMA33MZ	USA	Male	15	HMP2	UC	30.7	NA	7		4	H4042
HSMA33N4	USA	Male	15	HMP2	UC	30.7	NA	7		8	H4042
HSMA33IO	USA	Male	15	HMP2	UC	30.7	NA	5		16	H4042
HSMA33IS	USA	Male	15	HMP2	UC	30.7	NA	8		25	H4042
HSMA33RD	USA	Male	15	HMP2	UC	30.7	NA	NA		36	H4042
HSMA33RF	USA	Male	15	HMP2	UC	30.7	NA	8		39	H4042
HSMA33IE	USA	Female	14	HMP2	CD	22	NA	NA	L3	2	H4043
HSMA33IG	USA	Female	14	HMP2	CD	22	NA	NA	L3	4	H4043
HSMA33IK	USA	Female	14	HMP2	CD	22	1	NA	L3	8	H4043
HSMA33LP	USA	Female	14	HMP2	CD	22	1	NA	L3	16	H4043
HSMA33QY	USA	Female	14	HMP2	CD	22	2	NA	L3	25	H4043
HSMA33R1	USA	Female	14	HMP2	CD	22	6	NA	L3	26	H4043
HSMA33R5	USA	Female	14	HMP2	CD	22	2	NA	L3	30	H4043

HSMA33R7	USA	Female	14	HMP2	CD	22	2	NA	L3	33	H4043
HSMA33R9	USA	Female	14	HMP2	CD	22	1	NA	L3	35	H4043
HSMA33S4	USA	Female	14	HMP2	CD	22	1	NA	L3	38	H4043
HSMA33NW	USA	Male	16	HMP2	UC	22.3	NA	0		0	H4044
HSMA33P2_P	USA	Male	16	HMP2	UC	22.3	NA	3		2	H4044
HSMA33P6	USA	Male	16	HMP2	UC	22.3	NA	NA		6	H4044
HSMA33IA	USA	Male	16	HMP2	UC	22.3	NA	1		6	H4044
HSMA33IC	USA	Male	16	HMP2	UC	22.3	NA	NA		8	H4044
HSMA33LB	USA	Male	16	HMP2	UC	22.3	NA	3		16	H4044
HSMA33LH	USA	Male	16	HMP2	UC	22.3	NA	NA		23	H4044
HSMA33LH_TR	USA	Male	16	HMP2	UC	22.3	NA	NA		23	H4044
HSMA33LJ	USA	Male	16	HMP2	UC	22.3	NA	3		24	H4044
HSMA33RR	USA	Male	16	HMP2	UC	22.3	NA	NA		28	H4044
HSMA33RT	USA	Male	16	HMP2	UC	22.3	NA	NA		38	H4044
HSMA33RX	USA	Male	16	HMP2	UC	22.3	NA	NA		45	H4044
HSMA33RX_TR	USA	Male	16	HMP2	UC	22.3	NA	NA		45	H4044
HSMA33NY	USA	Female	14	HMP2	nonIBD	33.8	NA	NA		0	H4045
HSMA33J1_P	USA	Female	14	HMP2	nonIBD	33.8	NA	NA		1	H4045
HSMA33J3	USA	Female	14	HMP2	nonIBD	33.8	NA	NA		3	H4045
HSMA33J5	USA	Female	14	HMP2	nonIBD	33.8	NA	NA		5	H4045
HSMA33J7	USA	Female	14	HMP2	nonIBD	33.8	NA	NA		7	H4045
HSMA33J9	USA	Female	14	HMP2	nonIBD	33.8	NA	NA		10	H4045
HSMA33MS	USA	Female	14	HMP2	nonIBD	33.8	NA	NA		16	H4045
HSMA33QM	USA	Female	14	HMP2	nonIBD	33.8	NA	NA		23	H4045
HSMA33QO	USA	Female	14	HMP2	nonIBD	33.8	NA	NA		25	H4045
HSMA33SE	USA	Female	14	HMP2	nonIBD	33.8	NA	NA		33	H4045
HSMA33SG	USA	Female	14	HMP2	nonIBD	33.8	NA	NA		35	H4045
HSMA33SI	USA	Female	14	HMP2	nonIBD	33.8	NA	NA		37	H4045

HSMA33SK	USA	Female	14	HMP2	nonIBD		33.8	NA	NA		39	H4045
MSM5LLDI	USA	Female	30	HMP2	CD		24.1	NA	4		0	M2008
MSM5LLDK	USA	Female	30	HMP2	CD		24.1	NA	5		1	M2008
MSM5LLDM	USA	Female	30	HMP2	CD		24.1	NA	3		4	M2008
MSM5LLDQ	USA	Female	30	HMP2	CD		24.1	NA	2		8	M2008
MSM5LLDS	USA	Female	30	HMP2	CD		24.1	NA	2		10	M2008
MSM5LLDU	USA	Female	30	HMP2	CD		24.1	NA	3		12	M2008
MSM5LLHX_P	USA	Female	30	HMP2	CD		24.1	NA	5		14	M2008
MSM5LLI2_P	USA	Female	30	HMP2	CD		24.1	NA	2		18	M2008
MSM5LLI8_P	USA	Female	30	HMP2	CD		24.1	NA	2		21	M2008
MSM5LLI6_P	USA	Female	30	HMP2	CD		24.1	NA	2		22	M2008
MSM5LLI4_P	USA	Female	30	HMP2	CD		24.1	NA	3		25	M2008
MSM5LLHQ_P	USA	Female	30	HMP2	CD		24.1	NA	4		27	M2008
MSM5LLD6_P	USA	Female	30	HMP2	CD		24.1	NA	5		31	M2008
MSM5LLDC	USA	Female	30	HMP2	CD		24.1	NA	7		34	M2008
MSM5LLDE	USA	Female	30	HMP2	CD		24.1	NA	5		35	M2008
MSM5LLDA	USA	Female	30	HMP2	CD		24.1	NA	6		37	M2008
MSM6J2LB	USA	Female	30	HMP2	CD		24.1	6	NA		52	M2008
MSM5FZ9N_P	USA	Male	18	HMP2	CD	NA		NA	NA	L1	6	M2010
MSM5LLE3_P	USA	Male	18	HMP2	CD	NA		0	NA	L1	19	M2010
MSM5LLE9_P	USA	Male	18	HMP2	CD	NA		0	NA	L1	22	M2010
MSM5FZ9X_P	USA	Male	30	HMP2	CD		25.4	1	NA	L1	4	M2014
MSM5FZA2_P	USA	Male	30	HMP2	CD		25.4	3	NA	L1	8	M2014
MSM5ZOJY_P	USA	Male	30	HMP2	CD		25.4	1	NA	L1	10	M2014
MSM633FF_P	USA	Male	30	HMP2	CD		25.4	0	NA	L1	12	M2014
CSM5LLGB_P	USA	Male	30	HMP2	CD		25.4	0	NA	L1	14	M2014
MSM5LLGH_P	USA	Male	30	HMP2	CD		25.4	0	NA	L1	16	M2014
MSM5LLGF_P	USA	Male	30	HMP2	CD		25.4	1	NA	L1	18	M2014

MSM5LLGD_P	USA	Male	30	HMP2	CD		25.4	2	NA	L1	20	M2014
MSM5LLGJ_P	USA	Male	30	HMP2	CD		25.4	2	NA	L1	22	M2014
MSM5LLGL	USA	Male	30	HMP2	CD		25.4	2	NA	L1	24	M2014
MSM6J2IQ	USA	Male	30	HMP2	CD		25.4	0	NA	L1	26	M2014
MSM6J2IY	USA	Male	30	HMP2	CD		25.4	2	NA	L1	34	M2014
MSM6J2J1	USA	Male	30	HMP2	CD		25.4	4	NA	L1	36	M2014
MSM6J2QR	USA	Male	30	HMP2	CD		25.4	3	NA	L1	38	M2014
MSM5LLHR_P	USA	Male	26	HMP2	CD		19.1	2	NA	L2	0	M2021
MSM5LLIE_P	USA	Male	26	HMP2	CD		19.1	3	NA	L2	4	M2021
MSM5LLIG_P	USA	Male	26	HMP2	CD		19.1	1	NA	L2	6	M2021
MSM5LLIK_P	USA	Male	26	HMP2	CD		19.1	2	NA	L2	10	M2021
MSM5LLIM_P	USA	Male	26	HMP2	CD		19.1	1	NA	L2	12	M2021
MSM5LLEP	USA	Male	26	HMP2	CD		19.1	1	NA	L2	21	M2021
MSM5LLER	USA	Male	26	HMP2	CD		19.1	2	NA	L2	25	M2021
MSM6J2LT	USA	Male	26	HMP2	CD		19.1	2	NA	L2	34	M2021
MSM5LLHV_P	USA	Female	38	HMP2	UC	NA		NA	7		0	M2024
MSM5LLIC_P	USA	Female	43	HMP2	CD		19.3	NA	NA	L2	0	M2025
MSM5LLHE_P	USA	Female	43	HMP2	CD		19.3	NA	NA	L2	3	M2025
MSM5LLHG_P	USA	Female	43	HMP2	CD		19.3	5	NA	L2	6	M2025
MSM5LLHI_P	USA	Female	43	HMP2	CD		19.3	2	NA	L2	8	M2025
MSM5LLHO_P	USA	Female	43	HMP2	CD		19.3	6	NA	L2	14	M2025
MSM6J2J3	USA	Female	43	HMP2	CD		19.3	3	NA	L2	16	M2025
MSM6J2J5	USA	Female	43	HMP2	CD		19.3	7	NA	L2	18	M2025
MSM6J2JB	USA	Female	43	HMP2	CD		19.3	5	NA	L2	23	M2025
MSM6J2JD	USA	Female	43	HMP2	CD		19.3	2	NA	L2	24	M2025
MSM6J2PK	USA	Female	43	HMP2	CD		19.3	3	NA	L2	35	M2025
MSM6J2PM	USA	Female	43	HMP2	CD		19.3	6	NA	L2	38	M2025
MSM5LLIQ_P	USA	Female	21	HMP2	UC		20.3	NA	7		0	M2026

MSM5LLFK_P	USA	Female	21	HMP2	UC	20.3	NA	4		2	M2026
MSM5LLFM_P	USA	Female	21	HMP2	UC	20.3	NA	1		4	M2026
MSM5LLFO_P	USA	Female	21	HMP2	UC	20.3	NA	0		6	M2026
MSM5LLFU_P	USA	Female	21	HMP2	UC	20.3	NA	3		12	M2026
MSM6J2HB	USA	Female	21	HMP2	UC	20.3	NA	0		16	M2026
MSM6J2HD	USA	Female	21	HMP2	UC	20.3	NA	4		17	M2026
MSM6J2HF	USA	Female	21	HMP2	UC	20.3	NA	3		18	M2026
MSM6J2HH	USA	Female	21	HMP2	UC	20.3	NA	7		20	M2026
MSM6J2HJ	USA	Female	21	HMP2	UC	20.3	NA	0		22	M2026
MSM6J2HL	USA	Female	21	HMP2	UC	20.3	NA	0		24	M2026
MSM6J2OH	USA	Female	21	HMP2	UC	20.3	NA	0		26	M2026
MSM6J2OJ	USA	Female	21	HMP2	UC	20.3	NA	0		29	M2026
MSM6J2OL	USA	Female	21	HMP2	UC	20.3	NA	0		31	M2026
MSM6J2ON	USA	Female	21	HMP2	UC	20.3	NA	0		33	M2026
MSM6J2OP	USA	Female	21	HMP2	UC	20.3	NA	0		40	M2026
MSM79HEA	USA	Female	21	HMP2	UC	20.3	NA	0		45	M2026
MSM5LLIS_P	USA	Male	41	HMP2	CD	23.7	0	NA	L1	0	M2027
MSM5LLH2_P	USA	Male	41	HMP2	CD	23.7	5	NA	L1	2	M2027
MSM5LLH4_P	USA	Male	41	HMP2	CD	23.7	2	NA	L1	4	M2027
MSM5LLH8_P	USA	Male	41	HMP2	CD	23.7	2	NA	L1	8	M2027
MSM5LLHA_P	USA	Male	41	HMP2	CD	23.7	2	NA	L1	11	M2027
MSM5LLHC	USA	Male	41	HMP2	CD	23.7	2	NA	L1	15	M2027
MSM6J2KC	USA	Male	41	HMP2	CD	23.7	0	NA	L1	16	M2027
MSM6J2KE	USA	Male	41	HMP2	CD	23.7	2	NA	L1	18	M2027
MSM6J2KM	USA	Male	41	HMP2	CD	23.7	0	NA	L1	24	M2027
MSM6J2R2	USA	Male	41	HMP2	CD	23.7	0	NA	L1	26	M2027
MSM6J2R8	USA	Male	41	HMP2	CD	23.7	0	NA	L1	32	M2027
MSM6J2RA	USA	Male	41	HMP2	CD	23.7	0	NA	L1	36	M2027

MSM6J2RC	USA	Male	41	HMP2	CD	23.7	0	NA	L1	41	M2027
MSM5LLFG_P	USA	Female	24	HMP2	CD	20.9	3	NA	L1	0	M2028
MSM5LLGN_P	USA	Female	24	HMP2	CD	20.9	1	NA	L1	2	M2028
MSM5LLGR_P	USA	Female	24	HMP2	CD	20.9	2	NA	L1	6	M2028
MSM6J2IE	USA	Female	24	HMP2	CD	20.9	0	NA	L1	14	M2028
MSM6J2IG	USA	Female	24	HMP2	CD	20.9	4	NA	L1	17	M2028
MSM6J2II	USA	Female	24	HMP2	CD	20.9	1	NA	L1	19	M2028
MSM6J2IK	USA	Female	24	HMP2	CD	20.9	9	NA	L1	20	M2028
MSM6J2IM	USA	Female	24	HMP2	CD	20.9	0	NA	L1	23	M2028
MSM6J2IO	USA	Female	24	HMP2	CD	20.9	4	NA	L1	24	M2028
MSM6J2Q3	USA	Female	24	HMP2	CD	20.9	2	NA	L1	26	M2028
MSM6J2Q5	USA	Female	24	HMP2	CD	20.9	3	NA	L1	29	M2028
MSM6J2Q7	USA	Female	24	HMP2	CD	20.9	3	NA	L1	30	M2028
MSM6J2Q9	USA	Female	24	HMP2	CD	20.9	0	NA	L1	33	M2028
MSM6J2QB	USA	Female	24	HMP2	CD	20.9	0	NA	L1	36	M2028
MSM6J2QD	USA	Female	24	HMP2	CD	20.9	0	NA	L1	40	M2028
MSM79H63	USA	Female	24	HMP2	CD	20.9	2	NA	L1	41	M2028
MSM79H65	USA	Female	24	HMP2	CD	20.9	3	NA	L1	41	M2028
MSM79H67	USA	Female	24	HMP2	CD	20.9	2	NA	L1	44	M2028
MSM79H69	USA	Female	24	HMP2	CD	20.9	2	NA	L1	47	M2028
MSM79H6B	USA	Female	24	HMP2	CD	20.9	1	NA	L1	49	M2028
MSM5LLF2_P	USA	Female	22	HMP2	CD	31.2	1	NA	L3	5	M2034
MSM5LLF4	USA	Female	22	HMP2	CD	31.2	2	NA	L3	7	M2034
MSM5LLF6	USA	Female	22	HMP2	CD	31.2	6	NA	L3	10	M2034
MSM5LLF8	USA	Female	22	HMP2	CD	31.2	1	NA	L3	11	M2034
MSM6J2LH	USA	Female	22	HMP2	CD	31.2	4	NA	L3	14	M2034
MSM6J2LJ	USA	Female	22	HMP2	CD	31.2	1	NA	L3	15	M2034
MSM6J2LL	USA	Female	22	HMP2	CD	31.2	1	NA	L3	17	M2034

MSM6J2LN	USA	Female	22	HMP2	CD	31.2	3	NA	L3	19	M2034
MSM6J2LR	USA	Female	22	HMP2	CD	31.2	3	NA	L3	24	M2034
MSM6J2MB	USA	Female	22	HMP2	CD	31.2	2	NA	L3	25	M2034
MSM6J2MD	USA	Female	22	HMP2	CD	31.2	6	NA	L3	28	M2034
MSM6J2MF	USA	Female	22	HMP2	CD	31.2	3	NA	L3	30	M2034
MSM6J2MH	USA	Female	22	HMP2	CD	31.2	0	NA	L3	31	M2034
MSM6J2MJ	USA	Female	22	HMP2	CD	31.2	5	NA	L3	33	M2034
MSM6J2ML	USA	Female	22	HMP2	CD	31.2	5	NA	L3	36	M2034
MSM79HBN	USA	Female	22	HMP2	CD	31.2	2	NA	L3	38	M2034
MSM79HBP	USA	Female	22	HMP2	CD	31.2	3	NA	L3	39	M2034
MSM79HBR	USA	Female	22	HMP2	CD	31.2	2	NA	L3	42	M2034
MSM79HBT	USA	Female	22	HMP2	CD	31.2	5	NA	L3	43	M2034
MSM79HBV	USA	Female	22	HMP2	CD	31.2	1	NA	L3	45	M2034
CSM6J2H9_P	USA	Female	40	HMP2	nonIBD	40.6	NA	NA		0	M2039
MSM6J2HN	USA	Female	40	HMP2	nonIBD	40.6	NA	NA		2	M2039
MSM6J2HP	USA	Female	40	HMP2	nonIBD	40.6	NA	NA		5	M2039
MSM6J2HR	USA	Female	40	HMP2	nonIBD	40.6	NA	NA		7	M2039
MSM6J2HT	USA	Female	40	HMP2	nonIBD	40.6	NA	NA		10	M2039
MSM6J2QL	USA	Female	40	HMP2	nonIBD	40.6	NA	NA		16	M2039
MSM6J2QH	USA	Female	40	HMP2	nonIBD	40.6	NA	NA		18	M2039
MSM6J2QP	USA	Female	40	HMP2	nonIBD	40.6	NA	NA		20	M2039
MSM6J2QJ	USA	Female	40	HMP2	nonIBD	40.6	NA	NA		24	M2039
MSM6J2QF	USA	Female	40	HMP2	nonIBD	40.6	NA	NA		26	M2039
MSM79H5Q	USA	Female	40	HMP2	nonIBD	40.6	NA	NA		31	M2039
MSM79H5U	USA	Female	40	HMP2	nonIBD	40.6	NA	NA		33	M2039
MSM79H5Y	USA	Female	40	HMP2	nonIBD	40.6	NA	NA		35	M2039
MSM79H5S	USA	Female	40	HMP2	nonIBD	40.6	NA	NA		37	M2039
MSM79HAH	USA	Female	40	HMP2	nonIBD	40.6	NA	NA		48	M2039

MSM6J2JF_P	USA	Male	55	HMP2	nonIBD	22.7	NA	NA	0	M2041
MSM6J2JN	USA	Male	55	HMP2	nonIBD	22.7	NA	NA	2	M2041
MSM6J2JP	USA	Male	55	HMP2	nonIBD	22.7	NA	NA	3	M2041
MSM6J2JR	USA	Male	55	HMP2	nonIBD	22.7	NA	NA	8	M2041
MSM6J2JT	USA	Male	55	HMP2	nonIBD	22.7	NA	NA	9	M2041
MSM6J2LV	USA	Male	55	HMP2	nonIBD	22.7	NA	NA	16	M2041
MSM6J2SE	USA	Male	55	HMP2	nonIBD	22.7	NA	NA	18	M2041
MSM6J2SI	USA	Male	55	HMP2	nonIBD	22.7	NA	NA	24	M2041
MSM6J2SK	USA	Male	55	HMP2	nonIBD	22.7	NA	NA	25	M2041
MSM79HES	USA	Male	55	HMP2	nonIBD	22.7	NA	NA	33	M2041
MSM79HEU	USA	Male	55	HMP2	nonIBD	22.7	NA	NA	35	M2041
MSM79HEW	USA	Male	55	HMP2	nonIBD	22.7	NA	NA	36	M2041
MSM79HEY	USA	Male	55	HMP2	nonIBD	22.7	NA	NA	39	M2041
MSM79HB6	USA	Male	55	HMP2	nonIBD	22.7	NA	NA	50	M2041
MSM6J2JH_P	USA	Male	44	HMP2	nonIBD	24.5	NA	NA	0	M2042
MSM6J2JZ	USA	Male	44	HMP2	nonIBD	24.5	NA	NA	1	M2042
MSM6J2K2	USA	Male	44	HMP2	nonIBD	24.5	NA	NA	2	M2042
MSM6J2K4	USA	Male	44	HMP2	nonIBD	24.5	NA	NA	3	M2042
MSM6J2K6	USA	Male	44	HMP2	nonIBD	24.5	NA	NA	4	M2042
MSM6J2K8	USA	Male	44	HMP2	nonIBD	24.5	NA	NA	5	M2042
MSM6J2KA	USA	Male	44	HMP2	nonIBD	24.5	NA	NA	6	M2042
MSM6J2RK	USA	Male	44	HMP2	nonIBD	24.5	NA	NA	12	M2042
MSM6J2RM	USA	Male	44	HMP2	nonIBD	24.5	NA	NA	14	M2042
MSM6J2RO	USA	Male	44	HMP2	nonIBD	24.5	NA	NA	15	M2042
MSM6J2RQ	USA	Male	44	HMP2	nonIBD	24.5	NA	NA	16	M2042
MSM6J2RU	USA	Male	44	HMP2	nonIBD	24.5	NA	NA	19	M2042
MSM6J2RS	USA	Male	44	HMP2	nonIBD	24.5	NA	NA	20	M2042
MSM79H6D	USA	Male	44	HMP2	nonIBD	24.5	NA	NA	26	M2042

MSM79H6F	USA	Male	44	HMP2	nonIBD		24.5	NA	NA	27	M2042
MSM79H6J	USA	Male	44	HMP2	nonIBD		24.5	NA	NA	28	M2042
MSM79H6H	USA	Male	44	HMP2	nonIBD		24.5	NA	NA	29	M2042
MSM79H6L	USA	Male	44	HMP2	nonIBD		24.5	NA	NA	30	M2042
MSM79H6N	USA	Male	44	HMP2	nonIBD		24.5	NA	NA	31	M2042
MSM79H9Y	USA	Male	44	HMP2	nonIBD		24.5	NA	NA	34	M2042
MSM79HA1	USA	Male	44	HMP2	nonIBD		24.5	NA	NA	35	M2042
MSM79HA3	USA	Male	44	HMP2	nonIBD		24.5	NA	NA	40	M2042
MSM79HA7	USA	Male	44	HMP2	nonIBD		24.5	NA	NA	44	M2042
MSM6J2PO	USA	Male	57	HMP2	nonIBD		25.1	NA	NA	0	M2047
MSM6J2PQ_P	USA	Male	57	HMP2	nonIBD		25.1	NA	NA	2	M2047
MSM6J2PS	USA	Male	57	HMP2	nonIBD		25.1	NA	NA	4	M2047
MSM6J2PU	USA	Male	57	HMP2	nonIBD		25.1	NA	NA	7	M2047
MSM6J2PW	USA	Male	57	HMP2	nonIBD		25.1	NA	NA	10	M2047
MSM79HC4	USA	Male	57	HMP2	nonIBD		25.1	NA	NA	15	M2047
MSM79HC8	USA	Male	57	HMP2	nonIBD		25.1	NA	NA	20	M2047
MSM79H7O	USA	Male	57	HMP2	nonIBD		25.1	NA	NA	25	M2047
MSM79H7Q	USA	Male	57	HMP2	nonIBD		25.1	NA	NA	28	M2047
MSM79H7W	USA	Male	57	HMP2	nonIBD		25.1	NA	NA	32	M2047
MSM79H7Y	USA	Male	57	HMP2	nonIBD		25.1	NA	NA	33	M2047
MSM9VZNH	USA	Male	57	HMP2	nonIBD		25.1	NA	NA	35	M2047
MSM9VZNH_TR	USA	Male	57	HMP2	nonIBD		25.1	NA	NA	35	M2047
MSM9VZNL	USA	Male	57	HMP2	nonIBD		25.1	NA	NA	39	M2047
MSM6J2Q1	USA	Male	28	HMP2	nonIBD	NA		NA	NA	0	M2048
MSM6J2LW	USA	Male	28	HMP2	nonIBD	NA		NA	NA	2	M2048
MSM6J2LY	USA	Male	28	HMP2	nonIBD	NA		NA	NA	4	M2048
MSM6J2M3_P	USA	Male	28	HMP2	nonIBD	NA		NA	NA	9	M2048
MSM6J2M3	USA	Male	28	HMP2	nonIBD	NA		NA	NA	9	M2048

MSM79HBL	USA	Male		28	HMP2	nonIBD	NA		NA	NA		17	M2048
MSM79HFW	USA	Male		28	HMP2	nonIBD	NA		NA	NA		25	M2048
MSM79HFY	USA	Male		28	HMP2	nonIBD	NA		NA	NA		26	M2048
MSM79H6Y	USA	Male		28	HMP2	nonIBD	NA		NA	NA		33	M2048
MSM79HBB	USA	Male		28	HMP2	nonIBD	NA		NA	NA		38	M2048
MSM6J2RG_P	USA	Female		25	HMP2	CD	NA		NA	NA	L1	0	M2058
MSM6J2N6_P	USA	Male	NA		HMP2	UC	NA		NA	6		0	M2059
MSM79H52_P	USA	Female		62	HMP2	nonIBD		20.2	NA	NA		2	M2060
MSM79H54	USA	Female		62	HMP2	nonIBD		20.2	NA	NA		4	M2060
MSM79H58	USA	Female		62	HMP2	nonIBD		20.2	NA	NA		8	M2060
MSM79H5A	USA	Female		62	HMP2	nonIBD		20.2	NA	NA		10	M2060
MSM79H9A	USA	Female		62	HMP2	nonIBD		20.2	NA	NA		14	M2060
MSM79H9C	USA	Female		62	HMP2	nonIBD		20.2	NA	NA		16	M2060
MSM79H9G	USA	Female		62	HMP2	nonIBD		20.2	NA	NA		22	M2060
MSM79H9K	USA	Female		62	HMP2	nonIBD		20.2	NA	NA		23	M2060
MSM9VZF7	USA	Female		62	HMP2	nonIBD		20.2	NA	NA		27	M2060
MSM9VZFF	USA	Female		62	HMP2	nonIBD		20.2	NA	NA		34	M2060
MSM9VZFH	USA	Female		62	HMP2	nonIBD		20.2	NA	NA		36	M2060
MSM9VZPT	USA	Female		62	HMP2	nonIBD		20.2	NA	NA		38	M2060
MSM79H5E	USA	Male		56	HMP2	nonIBD		26.3	NA	NA		2	M2061
MSM79H5G	USA	Male		56	HMP2	nonIBD		26.3	NA	NA		4	M2061
MSM79H5K	USA	Male		56	HMP2	nonIBD		26.3	NA	NA		8	M2061
MSM79H5M	USA	Male		56	HMP2	nonIBD		26.3	NA	NA		10	M2061
MSM79H9M	USA	Male		56	HMP2	nonIBD		26.3	NA	NA		15	M2061
MSM79H9Q	USA	Male		56	HMP2	nonIBD		26.3	NA	NA		19	M2061
MSM79H9W	USA	Male		56	HMP2	nonIBD		26.3	NA	NA		25	M2061
MSM9VZGO	USA	Male		56	HMP2	nonIBD		26.3	NA	NA		30	M2061
MSM9VZGS	USA	Male		56	HMP2	nonIBD		26.3	NA	NA		34	M2061

MSM9VZGU	USA	Male	56	HMP2	nonIBD		26.3	NA	NA		36	M2061
MSM9VZLJ	USA	Male	56	HMP2	nonIBD		26.3	NA	NA		38	M2061
MSM9VZHB	USA	Male	56	HMP2	nonIBD		26.3	NA	NA		46	M2061
MSM9VZHF	USA	Male	56	HMP2	nonIBD		26.3	NA	NA		51	M2061
MSM79HF1_P	USA	Male	74	HMP2	UC		27.7	NA	4		0	M2064
MSM79HF3	USA	Male	74	HMP2	UC		27.7	NA	1		2	M2064
MSM79HF5	USA	Male	74	HMP2	UC		27.7	NA	3		4	M2064
MSM79HF7	USA	Male	74	HMP2	UC		27.7	NA	NA		6	M2064
MSM79HF9	USA	Male	74	HMP2	UC		27.7	NA	2		8	M2064
MSM79HF9_TR	USA	Male	74	HMP2	UC		27.7	NA	2		8	M2064
MSM79HFB	USA	Male	74	HMP2	UC		27.7	NA	NA		10	M2064
MSM79HAJ	USA	Male	74	HMP2	UC		27.7	NA	2		11	M2064
MSM79HAL	USA	Male	74	HMP2	UC		27.7	NA	3		14	M2064
MSM79HAN	USA	Male	74	HMP2	UC		27.7	NA	5		16	M2064
MSM79HAT	USA	Male	74	HMP2	UC		27.7	NA	3		20	M2064
MSM79HAR	USA	Male	74	HMP2	UC		27.7	NA	4		22	M2064
MSM9VZLL	USA	Male	74	HMP2	UC		27.7	NA	4		24	M2064
MSM9VZLN	USA	Male	74	HMP2	UC		27.7	NA	2		28	M2064
MSM9VZLP	USA	Male	74	HMP2	UC		27.7	NA	3		28	M2064
MSM9VZLR	USA	Male	74	HMP2	UC		27.7	NA	5		30	M2064
MSM9VZLV	USA	Male	74	HMP2	UC		27.7	NA	4		33	M2064
MSM9VZLT	USA	Male	74	HMP2	UC		27.7	NA	5		37	M2064
MSMA26ER	USA	Male	74	HMP2	UC		27.7	NA	4		41	M2064
MSMA26ET	USA	Male	74	HMP2	UC		27.7	NA	2		50	M2064
MSMA26EZ	USA	Male	74	HMP2	UC		27.7	NA	3		50	M2064
MSMA2688	USA	Male	74	HMP2	UC		27.7	NA	NA		53	M2064
MSM79H9U_P	USA	Male	35	HMP2	UC	NA		NA	NA		12	M2067
MSM79HBX_P	USA	Female	19	HMP2	CD		21.3	4	NA	L1	0	M2068

MSM79HDK	USA	Female	19	HMP2	CD	21.3	3	NA	L1	2	M2068
MSM79HDM	USA	Female	19	HMP2	CD	21.3	6	NA	L1	3	M2068
MSM79HDO	USA	Female	19	HMP2	CD	21.3	2	NA	L1	5	M2068
MSM79HDQ	USA	Female	19	HMP2	CD	21.3	5	NA	L1	8	M2068
MSM79HDQ_TR	USA	Female	19	HMP2	CD	21.3	5	NA	L1	8	M2068
MSM79HDS	USA	Female	19	HMP2	CD	21.3	3	NA	L1	9	M2068
MSM79HDU	USA	Female	19	HMP2	CD	21.3	3	NA	L1	11	M2068
MSM79H98	USA	Female	19	HMP2	CD	21.3	3	NA	L1	13	M2068
MSM9VZEK	USA	Female	19	HMP2	CD	21.3	4	NA	L1	15	M2068
MSM9VZEK_TR	USA	Female	19	HMP2	CD	21.3	4	NA	L1	15	M2068
MSM9VZEM	USA	Female	19	HMP2	CD	21.3	4	NA	L1	17	M2068
MSM9VZEO	USA	Female	19	HMP2	CD	21.3	1	NA	L1	20	M2068
MSM9VZEQ	USA	Female	19	HMP2	CD	21.3	NA	NA	L1	23	M2068
MSM9VZES	USA	Female	19	HMP2	CD	21.3	1	NA	L1	23	M2068
MSM7J16J	USA	Female	19	HMP2	CD	21.3	NA	NA	L1	26	M2068
MSM7J16L	USA	Female	19	HMP2	CD	21.3	2	NA	L1	28	M2068
MSM7J16N	USA	Female	19	HMP2	CD	21.3	3	NA	L1	30	M2068
MSM7J16P	USA	Female	19	HMP2	CD	21.3	5	NA	L1	32	M2068
MSM7J16R	USA	Female	19	HMP2	CD	21.3	5	NA	L1	34	M2068
MSM9VZJB	USA	Female	19	HMP2	CD	21.3	4	NA	L1	36	M2068
MSMA26AL	USA	Female	19	HMP2	CD	21.3	1	NA	L1	39	M2068
MSMA26AN	USA	Female	19	HMP2	CD	21.3	3	NA	L1	42	M2068
MSMA26AP	USA	Female	19	HMP2	CD	21.3	3	NA	L1	43	M2068
MSMA26AR	USA	Female	19	HMP2	CD	21.3	2	NA	L1	46	M2068
MSMA26AT	USA	Female	19	HMP2	CD	21.3	3	NA	L1	47	M2068
MSM79HBZ	USA	Female	29	HMP2	UC	19.1	NA	8		0	M2069
MSM79HD8_P	USA	Female	29	HMP2	UC	19.1	NA	1		2	M2069
MSM79HDA	USA	Female	29	HMP2	UC	19.1	NA	3		4	M2069

MSM79HDC	USA	Female	29	HMP2	UC		19.1	NA	3	6	M2069
MSM79HDE	USA	Female	29	HMP2	UC		19.1	NA	2	8	M2069
MSM79HDG	USA	Female	29	HMP2	UC		19.1	NA	NA	10	M2069
MSM79HDG_TR	USA	Female	29	HMP2	UC		19.1	NA	NA	10	M2069
MSM79HDI	USA	Female	29	HMP2	UC		19.1	NA	4	12	M2069
MSM9VZEU	USA	Female	29	HMP2	UC		19.1	NA	4	14	M2069
MSM9VZEW	USA	Female	29	HMP2	UC		19.1	NA	2	16	M2069
MSM9VZEY	USA	Female	29	HMP2	UC		19.1	NA	2	18	M2069
MSM9VZF1	USA	Female	29	HMP2	UC		19.1	NA	3	20	M2069
MSM9VZF3	USA	Female	29	HMP2	UC		19.1	NA	7	22	M2069
MSM9VZF5	USA	Female	29	HMP2	UC		19.1	NA	4	24	M2069
MSM9VZOY	USA	Female	29	HMP2	UC		19.1	NA	3	26	M2069
MSM9VZOU	USA	Female	29	HMP2	UC		19.1	NA	4	28	M2069
MSM9VZOW	USA	Female	29	HMP2	UC		19.1	NA	4	30	M2069
MSM9VZOS	USA	Female	29	HMP2	UC		19.1	NA	5	32	M2069
MSM9VZP1	USA	Female	29	HMP2	UC		19.1	NA	4	35	M2069
MSM9VZP3	USA	Female	29	HMP2	UC		19.1	NA	12	36	M2069
MSMA26EH	USA	Female	29	HMP2	UC		19.1	NA	6	39	M2069
MSMA26EJ	USA	Female	29	HMP2	UC		19.1	NA	5	41	M2069
MSMA26EL	USA	Female	29	HMP2	UC		19.1	NA	6	43	M2069
MSMA26EN	USA	Female	29	HMP2	UC		19.1	NA	6	45	M2069
MSMA26EP	USA	Female	29	HMP2	UC		19.1	NA	9	46	M2069
MSM79HD6_P	USA	Female	26	HMP2	UC	NA		NA	2	0	M2071
MSM79H7C	USA	Female	26	HMP2	UC	NA		NA	2	2	M2071
MSM79H7E	USA	Female	26	HMP2	UC	NA		NA	2	4	M2071
MSM79H7M	USA	Female	26	HMP2	UC	NA		NA	3	11	M2071
MSM79H7G	USA	Female	26	HMP2	UC	NA		NA	3	15	M2071
MSMA26AX	USA	Female	26	HMP2	UC	NA		NA	1	33	M2071

MSMA26AZ	USA	Female	26	HMP2	UC	NA		NA	4	34	M2071
MSMA26AZ_TR	USA	Female	26	HMP2	UC	NA		NA	4	34	M2071
MSMA26AV	USA	Female	26	HMP2	UC	NA		NA	1	39	M2071
MSMB4LZ4	USA	Female	26	HMP2	UC	NA		NA	2	57	M2071
MSM79HCG	USA	Male	51	HMP2	nonIBD		28.7	NA	NA	0	M2072
MSM79HCI	USA	Male	51	HMP2	nonIBD		28.7	NA	NA	4	M2072
MSM79HCK	USA	Male	51	HMP2	nonIBD		28.7	NA	NA	5	M2072
MSM79HCN_P	USA	Male	51	HMP2	nonIBD		28.7	NA	NA	6	M2072
MSM79HCP	USA	Male	51	HMP2	nonIBD		28.7	NA	NA	7	M2072
MSM79HCR	USA	Male	51	HMP2	nonIBD		28.7	NA	NA	9	M2072
MSM79H81	USA	Male	51	HMP2	nonIBD		28.7	NA	NA	11	M2072
MSM79H83	USA	Male	51	HMP2	nonIBD		28.7	NA	NA	13	M2072
MSM79H85	USA	Male	51	HMP2	nonIBD		28.7	NA	NA	15	M2072
MSM79H87	USA	Male	51	HMP2	nonIBD		28.7	NA	NA	17	M2072
MSM79H89	USA	Male	51	HMP2	nonIBD		28.7	NA	NA	19	M2072
MSM79H8B	USA	Male	51	HMP2	nonIBD		28.7	NA	NA	21	M2072
MSM9VZOG	USA	Male	51	HMP2	nonIBD		28.7	NA	NA	24	M2072
MSM9VZOI	USA	Male	51	HMP2	nonIBD		28.7	NA	NA	25	M2072
MSM9VZOK	USA	Male	51	HMP2	nonIBD		28.7	NA	NA	27	M2072
MSM9VZOM	USA	Male	51	HMP2	nonIBD		28.7	NA	NA	29	M2072
MSM9VZOO	USA	Male	51	HMP2	nonIBD		28.7	NA	NA	31	M2072
MSM9VZOQ	USA	Male	51	HMP2	nonIBD		28.7	NA	NA	33	M2072
MSM9VZHJ	USA	Male	51	HMP2	nonIBD		28.7	NA	NA	35	M2072
MSM9VZHL	USA	Male	51	HMP2	nonIBD		28.7	NA	NA	37	M2072
MSM9VZHN	USA	Male	51	HMP2	nonIBD		28.7	NA	NA	39	M2072
MSM9VZHP	USA	Male	51	HMP2	nonIBD		28.7	NA	NA	41	M2072
MSM9VZHR	USA	Male	51	HMP2	nonIBD		28.7	NA	NA	43	M2072
MSM9VZHT	USA	Male	51	HMP2	nonIBD		28.7	NA	NA	45	M2072

MSM79H8D	USA	Male	61	HMP2	nonIBD		27.2	NA	NA	0	M2075
MSM79H8F	USA	Male	61	HMP2	nonIBD		27.2	NA	NA	2	M2075
MSM79H8H	USA	Male	61	HMP2	nonIBD		27.2	NA	NA	3	M2075
MSM79H8J_P	USA	Male	61	HMP2	nonIBD		27.2	NA	NA	5	M2075
MSM79H8L	USA	Male	61	HMP2	nonIBD		27.2	NA	NA	7	M2075
MSM79H8N	USA	Male	61	HMP2	nonIBD		27.2	NA	NA	9	M2075
MSM9VZJZ	USA	Male	61	HMP2	nonIBD		27.2	NA	NA	14	M2075
MSM9VZGW	USA	Male	61	HMP2	nonIBD		27.2	NA	NA	24	M2075
MSM9VZGY	USA	Male	61	HMP2	nonIBD		27.2	NA	NA	26	M2075
MSM9VZH7	USA	Male	61	HMP2	nonIBD		27.2	NA	NA	34	M2075
MSMAPC7J	USA	Male	61	HMP2	nonIBD		27.2	NA	NA	37	M2075
MSM79H94_P	USA	Female	32	HMP2	nonIBD	NA		NA	NA	0	M2077
MSM9VZMA	USA	Female	32	HMP2	nonIBD	NA		NA	NA	5	M2077
MSM9VZMA_TR	USA	Female	32	HMP2	nonIBD	NA		NA	NA	5	M2077
MSM9VZME	USA	Female	32	HMP2	nonIBD	NA		NA	NA	9	M2077
MSM9VZMC	USA	Female	32	HMP2	nonIBD	NA		NA	NA	9	M2077
MSM9VZMI	USA	Female	32	HMP2	nonIBD	NA		NA	NA	16	M2077
MSM9VZHX	USA	Female	32	HMP2	nonIBD	NA		NA	NA	21	M2077
MSM9VZHZ	USA	Female	32	HMP2	nonIBD	NA		NA	NA	24	M2077
MSM9VZI2	USA	Female	32	HMP2	nonIBD	NA		NA	NA	26	M2077
MSM9VZI6	USA	Female	32	HMP2	nonIBD	NA		NA	NA	31	M2077
MSMAPC7P	USA	Female	32	HMP2	nonIBD	NA		NA	NA	36	M2077
MSMAPC7R	USA	Female	32	HMP2	nonIBD	NA		NA	NA	38	M2077
MSMAPC7T	USA	Female	32	HMP2	nonIBD	NA		NA	NA	39	M2077
MSMB4LZC	USA	Female	32	HMP2	nonIBD	NA		NA	NA	51	M2077
MSM9VZFJ_P	USA	Male	29	HMP2	nonIBD	NA		NA	NA	0	M2079
MSM9VZFL	USA	Male	29	HMP2	nonIBD	NA		NA	NA	1	M2079
MSM9VZFN	USA	Male	29	HMP2	nonIBD	NA		NA	NA	3	M2079

MSM9VZFR	USA	Male		29	HMP2	nonIBD	NA	NA	NA	8	M2079
MSM9VZFT	USA	Male		29	HMP2	nonIBD	NA	NA	NA	9	M2079
MSM9VZKC	USA	Male		29	HMP2	nonIBD	NA	NA	NA	15	M2079
MSM9VZKE	USA	Male		29	HMP2	nonIBD	NA	NA	NA	17	M2079
MSM9VZKI	USA	Male		29	HMP2	nonIBD	NA	NA	NA	21	M2079
MSMA26BN	USA	Male		29	HMP2	nonIBD	NA	NA	NA	28	M2079
MSMA26BR	USA	Male		29	HMP2	nonIBD	NA	NA	NA	32	M2079
MSMA26BT	USA	Male		29	HMP2	nonIBD	NA	NA	NA	34	M2079
MSMA26BV	USA	Male		29	HMP2	nonIBD	NA	NA	NA	36	M2079
MSMA26BX	USA	Male		29	HMP2	nonIBD	NA	NA	NA	38	M2079
MSMB4LZ8	USA	Male		29	HMP2	nonIBD	NA	NA	NA	57	M2079
MSM9VZN4_P	USA	Male		39	HMP2	UC	NA	NA	2	7	M2081
MSM9VZLX_P	USA	Male	NA		HMP2	CD	NA	0	NA	0	M2082
MSM9VZLZ	USA	Male		25	HMP2	UC	NA	NA	9	0	M2083
MSM9VZNR	USA	Male		25	HMP2	UC	NA	NA	2	3	M2083
MSM9VZNX	USA	Male		25	HMP2	UC	NA	NA	2	8	M2083
MSM9VZNZ	USA	Male		25	HMP2	UC	NA	NA	2	10	M2083
MSM9VZO2	USA	Male		25	HMP2	UC	NA	NA	1	13	M2083
MSM9VZIM	USA	Male		25	HMP2	UC	NA	NA	1	15	M2083
MSM9VZIO	USA	Male		25	HMP2	UC	NA	NA	0	17	M2083
MSM9VZIQ	USA	Male		25	HMP2	UC	NA	NA	1	20	M2083
MSM9VZIS	USA	Male		25	HMP2	UC	NA	NA	1	23	M2083
MSM9VZIU	USA	Male		25	HMP2	UC	NA	NA	0	25	M2083
MSM9VZIW	USA	Male		25	HMP2	UC	NA	NA	0	26	M2083
MSMAPC55	USA	Male		25	HMP2	UC	NA	NA	2	33	M2083
MSMAPC57	USA	Male		25	HMP2	UC	NA	NA	1	35	M2083
MSMAPC59	USA	Male		25	HMP2	UC	NA	NA	1	36	M2083
MSMAPC5B	USA	Male		25	HMP2	UC	NA	NA	1	37	M2083

MSMAPC5D	USA	Male	25	HMP2	UC	NA		NA	1		44	M2083
MSMB4LZP	USA	Male	25	HMP2	UC	NA		NA	0		46	M2083
MSMB4LZR	USA	Male	25	HMP2	UC	NA		NA	2		48	M2083
MSM9VZMM	USA	Female	23	HMP2	nonIBD		21.6	NA	NA		0	M2084
MSM9VZMO	USA	Female	23	HMP2	nonIBD		21.6	NA	NA		2	M2084
MSM9VZMS	USA	Female	23	HMP2	nonIBD		21.6	NA	NA		6	M2084
MSM9VZMU	USA	Female	23	HMP2	nonIBD		21.6	NA	NA		8	M2084
MSM9VZMW	USA	Female	23	HMP2	nonIBD		21.6	NA	NA		10	M2084
MSM9VZL7	USA	Female	23	HMP2	nonIBD		21.6	NA	NA		12	M2084
MSM9VZL9	USA	Female	23	HMP2	nonIBD		21.6	NA	NA		14	M2084
MSM9VZLB	USA	Female	23	HMP2	nonIBD		21.6	NA	NA		16	M2084
MSM9VZLD	USA	Female	23	HMP2	nonIBD		21.6	NA	NA		18	M2084
MSM9VZLF	USA	Female	23	HMP2	nonIBD		21.6	NA	NA		21	M2084
MSM9VZLH	USA	Female	23	HMP2	nonIBD		21.6	NA	NA		23	M2084
MSMA26BB	USA	Female	23	HMP2	nonIBD		21.6	NA	NA		25	M2084
MSMA26BD	USA	Female	23	HMP2	nonIBD		21.6	NA	NA		27	M2084
MSMA26BF	USA	Female	23	HMP2	nonIBD		21.6	NA	NA		29	M2084
MSMA26BH	USA	Female	23	HMP2	nonIBD		21.6	NA	NA		31	M2084
MSMA26BJ	USA	Female	23	HMP2	nonIBD		21.6	NA	NA		33	M2084
MSMA26BL	USA	Female	23	HMP2	nonIBD		21.6	NA	NA		35	M2084
MSMAPC6K	USA	Female	23	HMP2	nonIBD		21.6	NA	NA		37	M2084
MSMAPC6M	USA	Female	23	HMP2	nonIBD		21.6	NA	NA		39	M2084
MSMAPC6O	USA	Female	23	HMP2	nonIBD		21.6	NA	NA		41	M2084
MSMB4LZZ	USA	Female	23	HMP2	nonIBD		21.6	NA	NA		43	M2084
MSMB4LZV	USA	Female	23	HMP2	nonIBD		21.6	NA	NA		45	M2084
MSMB4LZX	USA	Female	23	HMP2	nonIBD		21.6	NA	NA		47	M2084
MSM9VZM4_P	USA	Male	23	HMP2	CD	NA		1	NA	L1	0	M2085
MSM9VZPH	USA	Male	23	HMP2	CD	NA		2	NA	L1	3	M2085

MSM9VZPL	USA	Male	23	HMP2	CD	NA	2	NA	L1	7	M2085
MSM9VZPN	USA	Male	23	HMP2	CD	NA	1	NA	L1	9	M2085
MSM9VZIY	USA	Male	23	HMP2	CD	NA	0	NA	L1	15	M2085
MSM9VZJ3	USA	Male	23	HMP2	CD	NA	0	NA	L1	19	M2085
MSMA26CV	USA	Male	23	HMP2	CD	NA	1	NA	L1	23	M2085
MSMA26CX	USA	Male	23	HMP2	CD	NA	4	NA	L1	26	M2085
MSMAPC6A	USA	Male	23	HMP2	CD	NA	0	NA	L1	31	M2085
MSMAPC6C	USA	Male	23	HMP2	CD	NA	0	NA	L1	33	M2085
MSMAPC6E	USA	Male	23	HMP2	CD	NA	0	NA	L1	34	M2085
MSMAPC6G	USA	Male	23	HMP2	CD	NA	2	NA	L1	36	M2085
MSMB4LXY	USA	Male	23	HMP2	CD	NA	2	NA	L1	40	M2085
MSMB4LYB	USA	Male	23	HMP2	CD	NA	0	NA	L1	50	M2085
MSM9VZJJ_P	USA	Male	22	HMP2	CD	NA	2	NA	L1	8	M2086
MSM9VZJF_P	USA	Male	56	HMP2	UC	NA	NA	2		0	M2091
MSM9VZL5	USA	Male	21	HMP2	nonIBD	NA	NA	NA		0	M2097
MSMA26DM	USA	Male	21	HMP2	nonIBD	NA	NA	NA		4	M2097
MSMA26DG	USA	Male	21	HMP2	nonIBD	NA	NA	NA		9	M2097
MSMA26DI	USA	Male	21	HMP2	nonIBD	NA	NA	NA		11	M2097
MSMA26DO	USA	Male	21	HMP2	nonIBD	NA	NA	NA		12	M2097
MSMA26DK	USA	Male	21	HMP2	nonIBD	NA	NA	NA		14	M2097
MSMAPC5H	USA	Male	21	HMP2	nonIBD	NA	NA	NA		22	M2097
MSMAPC5L	USA	Male	21	HMP2	nonIBD	NA	NA	NA		24	M2097
MSMB4LXW	USA	Male	21	HMP2	nonIBD	NA	NA	NA		31	M2097
MSMB4LXS	USA	Male	21	HMP2	nonIBD	NA	NA	NA		32	M2097
MSMB4LZK	USA	Male	21	HMP2	nonIBD	NA	NA	NA		38	M2097
MSMA267V	USA	Female	35	HMP2	UC	NA	NA	3		2	M2103
MSMA267X	USA	Female	35	HMP2	UC	NA	NA	1		5	M2103
MSMA2684	USA	Female	35	HMP2	UC	NA	NA	1		11	M2103

MSMAPC5Z	USA	Female	35	HMP2	UC	NA		NA	2		17	M2103
MSMAPC66	USA	Female	35	HMP2	UC	NA		NA	2		24	M2103
MSMAPC64	USA	Female	35	HMP2	UC	NA		NA	3		26	M2103
MSMB4LYH	USA	Female	35	HMP2	UC	NA		NA	1		35	M2103
PSM6XBQM_P	USA	Male	11	HMP2	CD		14.3	6	NA	L3+L4	0	P6005
PSM6XBQS	USA	Male	11	HMP2	CD		14.3	5	NA	L3+L4	2	P6005
PSM6XBQU	USA	Male	11	HMP2	CD		14.3	1	NA	L3+L4	4	P6005
PSM6XBQY	USA	Male	11	HMP2	CD		14.3	2	NA	L3+L4	8	P6005
PSM6XBQY_TR	USA	Male	11	HMP2	CD		14.3	2	NA	L3+L4	8	P6005
PSM6XBR1	USA	Male	11	HMP2	CD		14.3	1	NA	L3+L4	10	P6005
PSM6XBSS	USA	Male	11	HMP2	CD		14.3	1	NA	L3+L4	16	P6005
PSM6XBSU	USA	Male	11	HMP2	CD		14.3	0	NA	L3+L4	18	P6005
PSM6XBSU_TR	USA	Male	11	HMP2	CD		14.3	0	NA	L3+L4	18	P6005
PSM6XBT1	USA	Male	11	HMP2	CD		14.3	1	NA	L3+L4	24	P6005
PSM7J1C8	USA	Male	11	HMP2	CD		14.3	0	NA	L3+L4	26	P6005
PSM7J1CC	USA	Male	11	HMP2	CD		14.3	0	NA	L3+L4	30	P6005
PSM7J1CG	USA	Male	11	HMP2	CD		14.3	1	NA	L3+L4	34	P6005
PSM7J1CI	USA	Male	11	HMP2	CD		14.3	0	NA	L3+L4	37	P6005
PSM7J136	USA	Male	11	HMP2	CD		14.3	0	NA	L3+L4	41	P6005
PSM7J13E	USA	Male	11	HMP2	CD		14.3	NA	NA	L3+L4	49	P6005
PSM6XBRK_P	USA	Male	16	HMP2	CD		22.1	3	NA	L2	0	P6009
PSM6XBRK	USA	Male	16	HMP2	CD		22.1	3	NA	L2	0	P6009
PSM6XBRK_TR	USA	Male	16	HMP2	CD		22.1	3	NA	L2	0	P6009
PSM6XBS2_P	USA	Male	16	HMP2	CD		22.1	1	NA	L2	1	P6009
PSM6XBS4_P	USA	Male	16	HMP2	CD		22.1	2	NA	L2	3	P6009
PSM6XBS4	USA	Male	16	HMP2	CD		22.1	2	NA	L2	3	P6009
PSM6XBS8	USA	Male	16	HMP2	CD		22.1	6	NA	L2	7	P6009
PSM6XBSA	USA	Male	16	HMP2	CD		22.1	6	NA	L2	10	P6009

PSM6XBSC_P	USA	Male	16	HMP2	CD	22.1	4	NA	L2	11	P6009
PSM6XBTF_P	USA	Male	16	HMP2	CD	22.1	3	NA	L2	13	P6009
PSM6XBTH_P	USA	Male	16	HMP2	CD	22.1	1	NA	L2	15	P6009
PSM6XBTL	USA	Male	16	HMP2	CD	22.1	5	NA	L2	19	P6009
PSM6XBTN_P	USA	Male	16	HMP2	CD	22.1	2	NA	L2	21	P6009
PSM6XBTP	USA	Male	16	HMP2	CD	22.1	4	NA	L2	23	P6009
PSM7J1CK_P	USA	Male	16	HMP2	CD	22.1	5	NA	L2	25	P6009
PSM7J1CS_P	USA	Male	16	HMP2	CD	22.1	5	NA	L2	33	P6009
PSM7J1CU	USA	Male	16	HMP2	CD	22.1	4	NA	L2	36	P6009
PSM7J13U_P	USA	Male	16	HMP2	CD	22.1	4	NA	L2	39	P6009
PSM7J13Y_P	USA	Male	16	HMP2	CD	22.1	5	NA	L2	43	P6009
PSM7J13Y	USA	Male	16	HMP2	CD	22.1	5	NA	L2	43	P6009
PSM7J141	USA	Male	16	HMP2	CD	22.1	4	NA	L2	44	P6009
PSM7J143_P	USA	Male	16	HMP2	CD	22.1	2	NA	L2	46	P6009
PSM6XBRM_P	USA	Male	10	HMP2	CD	19	4	NA	L3+L4	0	P6010
PSM6XBSI	USA	Male	10	HMP2	CD	19	7	NA	L3+L4	2	P6010
PSM6XBSO	USA	Male	10	HMP2	CD	19	6	NA	L3+L4	4	P6010
PSM6XBSM	USA	Male	10	HMP2	CD	19	3	NA	L3+L4	6	P6010
PSM6XBSK	USA	Male	10	HMP2	CD	19	4	NA	L3+L4	9	P6010
PSM6XBV2	USA	Male	10	HMP2	CD	19	3	NA	L3+L4	10	P6010
PSM6XBV4	USA	Male	10	HMP2	CD	19	3	NA	L3+L4	12	P6010
PSM6XBUG	USA	Male	10	HMP2	CD	19	4	NA	L3+L4	15	P6010
PSM6XBUI	USA	Male	10	HMP2	CD	19	8	NA	L3+L4	17	P6010
PSM6XBUM	USA	Male	10	HMP2	CD	19	1	NA	L3+L4	20	P6010
PSM6XBUK	USA	Male	10	HMP2	CD	19	3	NA	L3+L4	21	P6010
PSM6XBUQ	USA	Male	10	HMP2	CD	19	6	NA	L3+L4	23	P6010
PSM6XBUO	USA	Male	10	HMP2	CD	19	2	NA	L3+L4	25	P6010
PSM7J18E	USA	Male	10	HMP2	CD	19	2	NA	L3+L4	27	P6010

PSM7J18G	USA	Male	10	HMP2	CD	19	3	NA	L3+L4	29	P6010
PSM7J18I	USA	Male	10	HMP2	CD	19	3	NA	L3+L4	31	P6010
PSM7J18K	USA	Male	10	HMP2	CD	19	3	NA	L3+L4	34	P6010
PSM7J18M	USA	Male	10	HMP2	CD	19	3	NA	L3+L4	35	P6010
PSM7J14L	USA	Male	10	HMP2	CD	19	6	NA	L3+L4	40	P6010
PSM7J14N	USA	Male	10	HMP2	CD	19	8	NA	L3+L4	41	P6010
PSM7J14P	USA	Male	10	HMP2	CD	19	6	NA	L3+L4	44	P6010
PSM7J14R	USA	Male	10	HMP2	CD	19	5	NA	L3+L4	45	P6010
PSM7J14T	USA	Male	10	HMP2	CD	19	2	NA	L3+L4	47	P6010
PSM6XBSE_P	USA	Female	16	HMP2	UC	21.1	NA	6		0	P6012
PSM6XBSE	USA	Female	16	HMP2	UC	21.1	NA	6		0	P6012
PSM6XBVI	USA	Female	16	HMP2	UC	21.1	NA	4		2	P6012
PSM6XBVK	USA	Female	16	HMP2	UC	21.1	NA	6		4	P6012
PSM6XBVM	USA	Female	16	HMP2	UC	21.1	NA	5		6	P6012
PSM6XBVO	USA	Female	16	HMP2	UC	21.1	NA	4		8	P6012
PSM6XBVQ	USA	Female	16	HMP2	UC	21.1	NA	4		10	P6012
PSM6XBVS	USA	Female	16	HMP2	UC	21.1	NA	3		12	P6012
PSM7J1B7	USA	Female	16	HMP2	UC	21.1	NA	4		15	P6012
PSM7J1B9	USA	Female	16	HMP2	UC	21.1	NA	5		17	P6012
PSM7J1BB	USA	Female	16	HMP2	UC	21.1	NA	4		19	P6012
PSM7J1BD	USA	Female	16	HMP2	UC	21.1	NA	7		23	P6012
PSM7J1BF	USA	Female	16	HMP2	UC	21.1	NA	7		23	P6012
PSM7J1BH	USA	Female	16	HMP2	UC	21.1	NA	9		26	P6012
PSM7J18Q	USA	Female	16	HMP2	UC	21.1	NA	3		35	P6012
PSM6XBSG_P	USA	Male	6	HMP2	UC	13.5	NA	2		0	P6013
PSM6XBT3	USA	Male	6	HMP2	UC	13.5	NA	3		2	P6013
PSM6XBT5	USA	Male	6	HMP2	UC	13.5	NA	3		4	P6013
PSM6XBT7	USA	Male	6	HMP2	UC	13.5	NA	3		6	P6013

PSM6XBT9	USA	Male	6	HMP2	UC	13.5	NA	2	8	P6013
PSM6XBTB	USA	Male	6	HMP2	UC	13.5	NA	3	10	P6013
PSM6XBTD	USA	Male	6	HMP2	UC	13.5	NA	3	12	P6013
PSM7J1AM	USA	Male	6	HMP2	UC	13.5	NA	3	14	P6013
PSM7J1AS	USA	Male	6	HMP2	UC	13.5	NA	3	16	P6013
PSM7J1AU	USA	Male	6	HMP2	UC	13.5	NA	3	18	P6013
PSM7J1AO	USA	Male	6	HMP2	UC	13.5	NA	3	20	P6013
PSM7J1AQ	USA	Male	6	HMP2	UC	13.5	NA	2	22	P6013
PSM7J1AW	USA	Male	6	HMP2	UC	13.5	NA	1	24	P6013
PSM7J193	USA	Male	6	HMP2	UC	13.5	NA	NA	26	P6013
PSM7J127	USA	Male	6	HMP2	UC	13.5	NA	NA	28	P6013
PSM7J129	USA	Male	6	HMP2	UC	13.5	NA	1	30	P6013
PSM7J12B	USA	Male	6	HMP2	UC	13.5	NA	2	33	P6013
PSM7J12D	USA	Male	6	HMP2	UC	13.5	NA	2	35	P6013
PSM7J12F	USA	Male	6	HMP2	UC	13.5	NA	3	36	P6013
PSM7J169	USA	Male	6	HMP2	UC	13.5	NA	3	38	P6013
PSM7J16F	USA	Male	6	HMP2	UC	13.5	NA	2	40	P6013
PSM7J16B	USA	Male	6	HMP2	UC	13.5	NA	6	44	P6013
PSM7J16H	USA	Male	6	HMP2	UC	13.5	NA	6	46	P6013
PSM6XBTR	USA	Male	15	HMP2	nonIBD	23.8	NA	NA	2	P6014
PSM6XBTT	USA	Male	15	HMP2	nonIBD	23.8	NA	NA	3	P6014
PSM6XBTX	USA	Male	15	HMP2	nonIBD	23.8	NA	NA	8	P6014
PSM6XBTZ_P	USA	Male	15	HMP2	nonIBD	23.8	NA	NA	9	P6014
PSM6XBU2	USA	Male	15	HMP2	nonIBD	23.8	NA	NA	12	P6014
PSM7J1DF	USA	Male	15	HMP2	nonIBD	23.8	NA	NA	17	P6014
PSM7J1DL	USA	Male	15	HMP2	nonIBD	23.8	NA	NA	22	P6014
PSM7J14X	USA	Male	15	HMP2	nonIBD	23.8	NA	NA	28	P6014
PSM7J154	USA	Male	15	HMP2	nonIBD	23.8	NA	NA	33	P6014

PSM7J156	USA	Male	15	HMP2	nonIBD		23.8	NA	NA		36	P6014
PSM6XBVY_P	USA	Male	16	HMP2	CD		14.7	2	NA	L3+L4	0	P6016
PSM7J199	USA	Male	16	HMP2	CD		14.7	2	NA	L3+L4	2	P6016
PSM7J19B	USA	Male	16	HMP2	CD		14.7	1	NA	L3+L4	4	P6016
PSM7J19F	USA	Male	16	HMP2	CD		14.7	1	NA	L3+L4	8	P6016
PSM7J19H	USA	Male	16	HMP2	CD		14.7	NA	NA	L3+L4	10	P6016
PSM7J19J	USA	Male	16	HMP2	CD		14.7	0	NA	L3+L4	12	P6016
PSM7J17L	USA	Male	16	HMP2	CD		14.7	1	NA	L3+L4	16	P6016
PSM7J17T	USA	Male	16	HMP2	CD		14.7	0	NA	L3+L4	24	P6016
PSM7J158	USA	Male	16	HMP2	CD		14.7	NA	NA	L3+L4	26	P6016
PSM7J15A	USA	Male	16	HMP2	CD		14.7	1	NA	L3+L4	28	P6016
PSM7J15G	USA	Male	16	HMP2	CD		14.7	0	NA	L3+L4	34	P6016
PSM7J15I	USA	Male	16	HMP2	CD		14.7	0	NA	L3+L4	36	P6016
PSMA265N	USA	Male	16	HMP2	CD		14.7	0	NA	L3+L4	38	P6016
PSMA265T	USA	Male	16	HMP2	CD		14.7	0	NA	L3+L4	45	P6016
PSM6XBW1_P	USA	Male	11	HMP2	nonIBD	NA		NA	NA		0	P6017
PSM7J19N	USA	Male	11	HMP2	nonIBD	NA		NA	NA		5	P6017
PSM7J19P	USA	Male	11	HMP2	nonIBD	NA		NA	NA		6	P6017
PSM7J19R	USA	Male	11	HMP2	nonIBD	NA		NA	NA		7	P6017
PSM7J19T	USA	Male	11	HMP2	nonIBD	NA		NA	NA		9	P6017
PSM7J16U	USA	Male	11	HMP2	nonIBD	NA		NA	NA		16	P6017
PSM7J16W	USA	Male	11	HMP2	nonIBD	NA		NA	NA		22	P6017
PSM7J16Y	USA	Male	11	HMP2	nonIBD	NA		NA	NA		30	P6017
PSM7J171	USA	Male	11	HMP2	nonIBD	NA		NA	NA		32	P6017
PSM7J173	USA	Male	11	HMP2	nonIBD	NA		NA	NA		35	P6017
PSM6XBW3	USA	Female	17	HMP2	nonIBD		20.9	NA	NA		0	P6018
PSM7J19X_P	USA	Female	17	HMP2	nonIBD		20.9	NA	NA		2	P6018
PSM7J19Z	USA	Female	17	HMP2	nonIBD		20.9	NA	NA		4	P6018

PSM7J1A2	USA	Female	17	HMP2	nonIBD	20.9	NA	NA		6	P6018
PSM7J1A4	USA	Female	17	HMP2	nonIBD	20.9	NA	NA		8	P6018
PSM7J1A6	USA	Female	17	HMP2	nonIBD	20.9	NA	NA		10	P6018
PSM7J1A8	USA	Female	17	HMP2	nonIBD	20.9	NA	NA		12	P6018
PSM7J17V	USA	Female	17	HMP2	nonIBD	20.9	NA	NA		14	P6018
PSM7J17X	USA	Female	17	HMP2	nonIBD	20.9	NA	NA		16	P6018
PSM7J17Z	USA	Female	17	HMP2	nonIBD	20.9	NA	NA		18	P6018
PSM7J182	USA	Female	17	HMP2	nonIBD	20.9	NA	NA		20	P6018
PSM7J184	USA	Female	17	HMP2	nonIBD	20.9	NA	NA		22	P6018
PSM7J186	USA	Female	17	HMP2	nonIBD	20.9	NA	NA		24	P6018
PSM7J15K	USA	Female	17	HMP2	nonIBD	20.9	NA	NA		26	P6018
PSM7J15M	USA	Female	17	HMP2	nonIBD	20.9	NA	NA		28	P6018
PSM7J15O	USA	Female	17	HMP2	nonIBD	20.9	NA	NA		30	P6018
PSM7J15Q	USA	Female	17	HMP2	nonIBD	20.9	NA	NA		32	P6018
PSM7J15S	USA	Female	17	HMP2	nonIBD	20.9	NA	NA		34	P6018
PSM7J15U	USA	Female	17	HMP2	nonIBD	20.9	NA	NA		36	P6018
PSMA265D	USA	Female	17	HMP2	nonIBD	20.9	NA	NA		38	P6018
PSMA265F	USA	Female	17	HMP2	nonIBD	20.9	NA	NA		40	P6018
PSMA265L	USA	Female	17	HMP2	nonIBD	20.9	NA	NA		42	P6018
PSMA265J	USA	Female	17	HMP2	nonIBD	20.9	NA	NA		44	P6018
PSMA265J_TR	USA	Female	17	HMP2	nonIBD	20.9	NA	NA		44	P6018
PSMA265H	USA	Female	17	HMP2	nonIBD	20.9	NA	NA		46	P6018
PSM7J1BJ	USA	Male	16	HMP2	CD	28.4	1	NA	L3+L4	2	P6024
PSM7J1BL	USA	Male	16	HMP2	CD	28.4	1	NA	L3+L4	4	P6024
PSM7J1BN_P	USA	Male	16	HMP2	CD	28.4	2	NA	L3+L4	6	P6024
PSM7J1BP	USA	Male	16	HMP2	CD	28.4	2	NA	L3+L4	8	P6024
PSM7J1BR	USA	Male	16	HMP2	CD	28.4	1	NA	L3+L4	10	P6024
PSM7J12J	USA	Male	16	HMP2	CD	28.4	2	NA	L3+L4	15	P6024

PSM7J12R	USA	Male	16	HMP2	CD	28.4	1	NA	L3+L4	23	P6024
PSMA263M	USA	Male	16	HMP2	CD	28.4	0	NA	L3+L4	25	P6024
PSMA263S	USA	Male	16	HMP2	CD	28.4	1	NA	L3+L4	32	P6024
PSMA263U	USA	Male	16	HMP2	CD	28.4	1	NA	L3+L4	35	P6024
PSMA263W	USA	Male	16	HMP2	CD	28.4	1	NA	L3+L4	37	P6024
PSM7J1BV	USA	Male	17	HMP2	UC	19.6	NA	1		2	P6025
PSM7J1BX	USA	Male	17	HMP2	UC	19.6	NA	0		4	P6025
PSM7J1C2_P	USA	Male	17	HMP2	UC	19.6	NA	0		8	P6025
PSM7J1C4	USA	Male	17	HMP2	UC	19.6	NA	2		10	P6025
PSM7J12V	USA	Male	17	HMP2	UC	19.6	NA	0		16	P6025
PSM7J12Z	USA	Male	17	HMP2	UC	19.6	NA	0		23	P6025
PSMA264K	USA	Male	17	HMP2	UC	19.6	NA	2		38	P6025
PSM7J1B3_P	USA	Male	9	HMP2	CD	15.9	6	NA	L1+L4	0	P6028
PSM7J177	USA	Male	9	HMP2	CD	15.9	7	NA	L1+L4	2	P6028
PSM7J179	USA	Male	9	HMP2	CD	15.9	8	NA	L1+L4	4	P6028
PSM7J17B	USA	Male	9	HMP2	CD	15.9	5	NA	L1+L4	6	P6028
PSM7J17D	USA	Male	9	HMP2	CD	15.9	3	NA	L1+L4	8	P6028
PSM7J17F	USA	Male	9	HMP2	CD	15.9	3	NA	L1+L4	10	P6028
PSM7J15W	USA	Male	9	HMP2	CD	15.9	0	NA	L1+L4	19	P6028
PSM7J161	USA	Male	9	HMP2	CD	15.9	2	NA	L1+L4	23	P6028
PSM7J163	USA	Male	9	HMP2	CD	15.9	2	NA	L1+L4	25	P6028
PSMA2668	USA	Male	9	HMP2	CD	15.9	NA	NA	L1+L4	33	P6028
PSMA266C	USA	Male	9	HMP2	CD	15.9	NA	NA	L1+L4	38	P6028
PSM7J13I	USA	Male	15	HMP2	CD	16.4	3	NA	L3	3	P6033
PSM7J13K	USA	Male	15	HMP2	CD	16.4	3	NA	L3	4	P6033
PSM7J13Q	USA	Male	15	HMP2	CD	16.4	1	NA	L3	8	P6033
PSM7J13M	USA	Male	15	HMP2	CD	16.4	2	NA	L3	10	P6033
PSMA2651_P	USA	Male	15	HMP2	CD	16.4	2	NA	L3	15	P6033

PSMA2653	USA	Male	15	HMP2	CD		16.4	2	NA	L3	17	P6033
PSMA2659	USA	Male	15	HMP2	CD		16.4	1	NA	L3	24	P6033
PSMA265B	USA	Male	15	HMP2	CD		16.4	0	NA	L3	26	P6033
PSMA267D	USA	Male	15	HMP2	CD		16.4	3	NA	L3	34	P6033
PSMA267F	USA	Male	15	HMP2	CD		16.4	3	NA	L3	36	P6033
PSMA267H	USA	Male	15	HMP2	CD		16.4	3	NA	L3	38	P6033
PSMA264O	USA	Male	16	HMP2	UC	NA		NA	0		2	P6035
PSMA264Q	USA	Male	16	HMP2	UC	NA		NA	0		4	P6035
PSMA264S	USA	Male	16	HMP2	UC	NA		NA	2		6	P6035
PSMA264U	USA	Male	16	HMP2	UC	NA		NA	1		8	P6035
PSMA264W	USA	Male	16	HMP2	UC	NA		NA	1		10	P6035
PSMA267J	USA	Male	16	HMP2	UC	NA		NA	1		15	P6035
PSMA267P	USA	Male	16	HMP2	UC	NA		NA	2		23	P6035
PSMA267R	USA	Male	16	HMP2	UC	NA		NA	1		25	P6035
PSMB4MBK	USA	Male	16	HMP2	UC	NA		NA	2		34	P6035
PSMB4MBI	USA	Male	16	HMP2	UC	NA		NA	1		36	P6035
PSMB4MC7	USA	Male	16	HMP2	UC	NA		NA	1		38	P6035
PSM7J4EF	USA	Male	15	HMP2	CD	NA		4	NA	L3	0	P6037
PSMA266I	USA	Male	15	HMP2	CD	NA		0	NA	L3	3	P6037
PSMA266M	USA	Male	15	HMP2	CD	NA		1	NA	L3	8	P6037
PSMA266O	USA	Male	15	HMP2	CD	NA		0	NA	L3	10	P6037
PSMA266Q	USA	Male	15	HMP2	CD	NA		0	NA	L3	12	P6037
PSMA269G	USA	Male	15	HMP2	CD	NA		0	NA	L3	16	P6037
PSMA269O	USA	Male	15	HMP2	CD	NA		2	NA	L3	24	P6037
PSMB4MBS	USA	Male	15	HMP2	CD	NA		1	NA	L3	33	P6037
PSMA265X	USA	Female	16	HMP2	UC	NA		NA	2		0	P6038
PSMA266U	USA	Female	16	HMP2	UC	NA		NA	0		2	P6038
PSMA266Y	USA	Female	16	HMP2	UC	NA		NA	2		7	P6038

PSMA2671	USA	Female	16	HMP2	UC	NA	NA	1	9	P6038
PSMA2675	USA	Female	16	HMP2	UC	NA	NA	1	13	P6038
PSMA269S	USA	Female	16	HMP2	UC	NA	NA	2	15	P6038
PSMA269W	USA	Female	16	HMP2	UC	NA	NA	2	20	P6038
PSMA26A1	USA	Female	16	HMP2	UC	NA	NA	2	24	P6038
PSMA26A3	USA	Female	16	HMP2	UC	NA	NA	2	26	P6038
PSMB4MC1	USA	Female	16	HMP2	UC	NA	NA	0	34	P6038
PSMB4MC3	USA	Female	16	HMP2	UC	NA	NA	0	35	P6038
PSMB4MC5	USA	Female	16	HMP2	UC	NA	NA	2	37	P6038
