# Supplementary material for: Intercontinental Gut Microbiome Variances in IBD
Source: Int J Mol Sci. 2022 Sep 17;23(18):10868. doi: 10.3390/ijms231810868 (PMC9506019; doi:10.3390/ijms231810868)
Supplement: Supplementary file 1 [file ijms-23-10868-s001.zip › ijms-1914741-supplementary/supplementary_tableS2.pdf]

feature	metadata	value	coef	stderr	N	pval	qval
Ruminococcus_bromii	disease	Healthy	0.02786745	0.00693793	972	9.5593E-05	0.03012975
Escherichia_coli	disease	Healthy	-0.03824046	0.00976204	972	0.00017723	0.03012975
Ruminococcus_bicirculans	disease	Healthy	0.00551447	0.00149246	972	0.00036795	0.04170083
