# Supplementary material for: Intercontinental Gut Microbiome Variances in IBD
Source: Int J Mol Sci. 2022 Sep 17;23(18):10868. doi: 10.3390/ijms231810868 (PMC9506019; doi:10.3390/ijms231810868)
Supplement: Supplementary file 1 [file ijms-23-10868-s001.zip › ijms-1914741-supplementary/supplementary_tableS3.pdf]

feature	metadata	value	coef	stderr	N	pval	qval
Coprococcus_comes	country	USA	-0.01326947	0.00244658	409	6.006E-07	0.0002042
Fusicatenibacter_saccharivorans	country	USA	-0.00824545	0.00192803	409	6.9259E-05	0.00588702
Dorea_longicatena	country	USA	-0.00980416	0.00221651	409	5.0832E-05	0.00588702
Dorea_formicigenerans	country	USA	-0.00424869	0.00100388	409	6.4422E-05	0.00588702
