# Supplementary material for: Intercontinental Gut Microbiome Variances in IBD
Source: Int J Mol Sci. 2022 Sep 17;23(18):10868. doi: 10.3390/ijms231810868 (PMC9506019; doi:10.3390/ijms231810868)
Supplement: Supplementary file 1 [file ijms-23-10868-s001.zip › ijms-1914741-supplementary/supplementary_tableS4.pdf]

feature	metadata	value	coef	stderr	N	pval	qval
Dorea_formicigenerans	country	USA	-0.008099557	0.001542897	384	4.03905E-06	0.00137328
Coprococcus_comes	country	USA	-0.013097298	0.002572559	384	8.62408E-06	0.00146609
Eubacterium_hallii	country	USA	-0.014583599	0.002962656	384	1.33176E-05	0.00150932
Dorea_longicatena	country	USA	-0.009890715	0.002095227	384	4.11886E-05	0.00350103
Anaerostipes_hadrus	country	USA	-0.026187401	0.006272325	384	0.000144077	0.00708693
Agathobaculum_butyriciproducens	country	USA	-0.002581345	0.000750119	384	0.001268838	0.0431405
Coprococcus_catus	country	USA	-0.00159868	0.000454756	384	0.001145586	0.0431405
Blautia_wexlerae	country	USA	-0.005246616	0.001490123	384	0.001017441	0.0431405
