# Supplementary material for: Intercontinental Gut Microbiome Variances in IBD
Source: Int J Mol Sci. 2022 Sep 17;23(18):10868. doi: 10.3390/ijms231810868 (PMC9506019; doi:10.3390/ijms231810868)
Supplement: Supplementary file 1 [file ijms-23-10868-s001.zip › ijms-1914741-supplementary/supplementary_tableS5.pdf]

SampleID	country	gender	Age	study	disease	BMI	hbi	cai	cd localization	cd behaviour	uc extent	dysbiosisindex	Time point	patientID	extraction protocol	shannon	chao
ERR1620322	China	Male	26	Chinese	Healthy	21,3	#N/D	#N/D	#N/D	#N/D	#N/D	-1,24	0	ERR1620322	Godon	2,594041287	56
ERR1620323	China	Male	44	Chinese	Healthy	25,4	#N/D	#N/D	#N/D	#N/D	#N/D	-1,44	0	ERR1620323	Godon	2,645716727	43
ERR1620324	China	Male	24	Chinese	Healthy	23,7	#N/D	#N/D	#N/D	#N/D	#N/D	-0,91	0	ERR1620324	Godon	2,565068329	47
ERR1620325	China	Male	24	Chinese	Healthy	22	#N/D	#N/D	#N/D	#N/D	#N/D	-1,97	0	ERR1620325	Godon	2,151116111	50
ERR1620326	China	Female	22	Chinese	Healthy	20,7	#N/D	#N/D	#N/D	#N/D	#N/D	-1,2	0	ERR1620326	Godon	3,130638096	60
ERR1620327	China	Male	23	Chinese	Healthy	18,7	#N/D	#N/D	#N/D	#N/D	#N/D	-1,14	0	ERR1620327	Godon	2,09992335	57
ERR1620329	China	Male	24	Chinese	Healthy	21,5	#N/D	#N/D	#N/D	#N/D	#N/D	-1,27	0	ERR1620329	Godon	2,580378041	61
ERR1620330	China	Male	23	Chinese	Healthy	21,3	#N/D	#N/D	#N/D	#N/D	#N/D	-1,18	0	ERR1620330	Godon	2,324924596	50
ERR1620331	China	Male	24	Chinese	Healthy	20,2	#N/D	#N/D	#N/D	#N/D	#N/D	-2,1	0	ERR1620331	Godon	2,886419942	64
ERR1620332	China	Male	24	Chinese	Healthy	22,5	#N/D	#N/D	#N/D	#N/D	#N/D	-0,98	0	ERR1620332	Godon	2,844431378	55
ERR1620334	China	Male	14	Chinese	Healthy	21	#N/D	#N/D	#N/D	#N/D	#N/D	-1,02	0	ERR1620334	Godon	2,575957921	49
ERR1620335	China	Male	13	Chinese	Healthy	17,4	#N/D	#N/D	#N/D	#N/D	#N/D	-1,02	0	ERR1620335	Godon	2,558359739	45
ERR1620336	China	Male	13	Chinese	Healthy	17,6	#N/D	#N/D	#N/D	#N/D	#N/D	-1,36	0	ERR1620336	Godon	2,02394749	42
ERR1620337	China	Male	14	Chinese	Healthy	20	#N/D	#N/D	#N/D	#N/D	#N/D	-0,88	0	ERR1620337	Godon	2,802049923	60
ERR1620338	China	Male	13	Chinese	Healthy	17,1	#N/D	#N/D	#N/D	#N/D	#N/D	-1,21	0	ERR1620338	Godon	3,01689686	65

SampleID	country	gender	Age	study	disease	BMI	hbi	cai	cd localization	cd behaviour	uc extent	dysbiosisindex	Time point	patientID	extraction protocol	shannon	chao
ERR1620339	China	Male	14	Chinese	Healthy	18,8	#N/D	#N/D	#N/D	#N/D	#N/D	-1,21	0	ERR1620339	Godon	3,047409557	55
ERR1620340	China	Male	14	Chinese	Healthy	23,5	#N/D	#N/D	#N/D	#N/D	#N/D	-1,41	0	ERR1620340	Godon	2,725365823	62
ERR1620341	China	Male	13	Chinese	Healthy	19,5	#N/D	#N/D	#N/D	#N/D	#N/D	-1,18	0	ERR1620341	Godon	3,147485371	65
ERR1620342	China	Male	14	Chinese	Healthy	18,8	#N/D	#N/D	#N/D	#N/D	#N/D	-1,46	0	ERR1620342	Godon	3,129324428	64
ERR1620343	China	Male	16	Chinese	Healthy	21,1	#N/D	#N/D	#N/D	#N/D	#N/D	-1,73	0	ERR1620343	Godon	2,520604558	59
ERR1620344	China	Male	16	Chinese	Healthy	21	#N/D	#N/D	#N/D	#N/D	#N/D	-1,15	0	ERR1620344	Godon	3,052201271	66
ERR1620346	China	Male	16	Chinese	Healthy	18,8	#N/D	#N/D	#N/D	#N/D	#N/D	-1,41	0	ERR1620346	Godon	2,78402354	50
ERR1620347	China	Male	17	Chinese	Healthy	19	#N/D	#N/D	#N/D	#N/D	#N/D	-0,81	0	ERR1620347	Godon	3,176671816	62
ERR1620348	China	Male	16	Chinese	Healthy	20,8	#N/D	#N/D	#N/D	#N/D	#N/D	-1,34	0	ERR1620348	Godon	2,460437	39
ERR1620349	China	Male	16	Chinese	Healthy	29,5	#N/D	#N/D	#N/D	#N/D	#N/D	-0,77	0	ERR1620349	Godon	2,390101756	44
ERR1620350	China	Male	17	Chinese	Healthy	21,1	#N/D	#N/D	#N/D	#N/D	#N/D	-1,45	0	ERR1620350	Godon	2,273044172	39
ERR1620351	China	Male	16	Chinese	Healthy	17,8	#N/D	#N/D	#N/D	#N/D	#N/D	-1,4	0	ERR1620351	Godon	2,41633199	45
ERR1620352	China	Male	16	Chinese	Healthy	19,1	#N/D	#N/D	#N/D	#N/D	#N/D	-1,09	0	ERR1620352	Godon	2,5337573	55
ERR1620353	China	Male	16	Chinese	Healthy	20,9	#N/D	#N/D	#N/D	#N/D	#N/D	-0,96	0	ERR1620353	Godon	2,562272711	61
ERR1620355	China	Male	17	Chinese	Healthy	19,8	#N/D	#N/D	#N/D	#N/D	#N/D	-1,53	0	ERR1620355	Godon	2,719000745	63

SampleID	country	gender	Age	study	disease	BMI	hbi	cai	cd localization	cd behaviour	uc extent	dysbiosisindex	Time point	patientID	extraction protocol	shannon	chao
ERR1620356	China	Male	17	Chinese	Healthy	21	#N/D	#N/D	#N/D	#N/D	#N/D	-1,33	0	ERR1620356	Godon	2,783005066	61
ERR1620357	China	Male	18	Chinese	Healthy	21,3	#N/D	#N/D	#N/D	#N/D	#N/D	-1,53	0	ERR1620357	Godon	2,422835583	61
ERR1620358	China	Male	20	Chinese	Healthy	19,6	#N/D	#N/D	#N/D	#N/D	#N/D	-0,89	0	ERR1620358	Godon	1,866023802	50
ERR1620359	China	Male	19	Chinese	Healthy	24,5	#N/D	#N/D	#N/D	#N/D	#N/D	-1,54	0	ERR1620359	Godon	2,232225489	43
ERR1620360	China	Male	18	Chinese	Healthy	21,4	#N/D	#N/D	#N/D	#N/D	#N/D	-1,36	0	ERR1620360	Godon	3,052247981	63
ERR1620361	China	Male	18	Chinese	Healthy	19,7	#N/D	#N/D	#N/D	#N/D	#N/D	-0,98	0	ERR1620361	Godon	2,442687962	42
ERR1620362	China	Male	17	Chinese	Healthy	22	#N/D	#N/D	#N/D	#N/D	#N/D	-1,37	0	ERR1620362	Godon	2,542676031	45
ERR1620363	China	Male	17	Chinese	Healthy	24,9	#N/D	#N/D	#N/D	#N/D	#N/D	-0,51	0	ERR1620363	Godon	2,377171852	56
ERR1620364	China	Male	19	Chinese	Healthy	19,4	#N/D	#N/D	#N/D	#N/D	#N/D	-1,5	0	ERR1620364	Godon	2,572807753	55
ERR1620365	China	Male	40	Chinese	Healthy	28,4	#N/D	#N/D	#N/D	#N/D	#N/D	-1,52	0	ERR1620365	Godon	2,813555799	60
ERR1620366	China	Male	23	Chinese	Healthy	18,5	#N/D	#N/D	#N/D	#N/D	#N/D	-0,59	0	ERR1620366	Godon	2,706924162	50
ERR1620367	China	Male	17	Chinese	Healthy	27,5	#N/D	#N/D	#N/D	#N/D	#N/D	-1,7	0	ERR1620367	Godon	2,823294532	65
ERR1620368	China	Male	17	Chinese	Healthy	19,1	#N/D	#N/D	#N/D	#N/D	#N/D	-1,32	0	ERR1620368	Godon	3,019858852	62
ERR1620369	China	Male	17	Chinese	Healthy	18	#N/D	#N/D	#N/D	#N/D	#N/D	-0,93	0	ERR1620369	Godon	1,902154486	59
ERR1620370	China	Male	19	Chinese	Healthy	23,9	#N/D	#N/D	#N/D	#N/D	#N/D	-1,42	0	ERR1620370	Godon	2,386144937	61

SampleID	country	gender	Age	study	disease	BMI	hbi	cai	cd localization	cd behaviour	uc extent	dysbiosisindex	Time point	patientID	extraction protocol	shannon	chao
ERR1620371	China	Male	23	Chinese	Healthy	30,9	#N/D	#N/D	#N/D	#N/D	#N/D	-1,65	0	ERR1620371	Godon	2,859772943	68
ERR1620372	China	Male	25	Chinese	Healthy	19,3	#N/D	#N/D	#N/D	#N/D	#N/D	-1,61	0	ERR1620372	Godon	2,676853206	48
ERR1620373	China	Female	39	Chinese	Healthy	18,7	#N/D	#N/D	#N/D	#N/D	#N/D	-1,03	0	ERR1620373	Godon	2,601550439	47
ERR1620374	China	Male	26	Chinese	Healthy	25,4	#N/D	#N/D	#N/D	#N/D	#N/D	-1,64	0	ERR1620374	Godon	2,769602516	64,5
ERR1620375	China	Female	51	Chinese	Healthy	26,4	#N/D	#N/D	#N/D	#N/D	#N/D	-0,62	0	ERR1620375	Godon	2,765903865	58
ERR1620376	China	Male	24	Chinese	Healthy	18,5	#N/D	#N/D	#N/D	#N/D	#N/D	-1,01	0	ERR1620376	Godon	2,511847527	47
ERR1620377	China	Male	27	Chinese	Healthy	29,4	#N/D	#N/D	#N/D	#N/D	#N/D	-1,31	0	ERR1620377	Godon	2,825570522	58
V1.CD1.0.PN	Spain	Female	54	Meta Hit	CD	25,97	3	#N/D	L1	B1	#N/D	#N/D	0	1PNCD	Godon	2,902984879	55
V1.CD3.0.PN	Spain	Female	54	Meta Hit	CD	19,03	1	#N/D	L1	B2	#N/D	#N/D	0	3PNCD	Godon	2,151847545	34
V1.CD5.0.PN	Spain	Female	35	Meta Hit	CD	28,83	2	#N/D	L1	B2	#N/D	#N/D	0	5PNCD	Godon	2,06584836	20
V1.CD7.0.PN	Spain	Male	51	Meta Hit	CD	22,5	2	#N/D	L1	B2	#N/D	#N/D	0	7PNCD	Godon	0,752416296	7,5
V1.CD1.0.PT	Spain	Female	26	Meta Hit	CD	17,9	1	#N/D	L3	B2	#N/D	#N/D	0	1PTCD	Godon	2,842917482	47,5
V1.CD2.0.PT	Spain	Male	58	Meta Hit	CD	20,4	2	#N/D	L1	B2	#N/D	#N/D	0	2PTCD	Godon	2,213140368	34
V1.CD3.0.PT	Spain	Female	33	Meta Hit	CD	25	1	#N/D	L3	B2	#N/D	#N/D	0	3PTCD	Godon	1,315009236	31,5
V1.CD1.0	Spain	Female	25	Meta Hit	CD	17,93	1	#N/D	L3	B3	#N/D	#N/D	0	1CD	Godon	2,150260323	29

SampleID	country	gender	Age	study	disease	BMI	hbi	cai	cd localization	cd behaviour	uc extent	dysbiosisindex	Time point	patientID	extraction protocol	shannon	chao
V1.CD6.0	Spain	Female	36	Meta Hit	CD	18,52	0	#N/D	L3	B2	#N/D	#N/D	0	6CD	Godon	2,494119872	38
V1.CD7.0	Spain	Male	22	Meta Hit	CD	18,17	0	#N/D	L1	B2	#N/D	#N/D	0	7CD	Godon	1,987282828	39
V1.CD10.0	Spain	Female	38	Meta Hit	CD	29,38	0	#N/D	L3	B2	#N/D	#N/D	0	10CD	Godon	1,910148243	32
V1.CD12.0	Spain	Female	41	Meta Hit	CD	20,2	1	#N/D	L3	B2	#N/D	#N/D	0	12CD	Godon	1,429311242	15
V1.CD15.0	Spain	Female	34	Meta Hit	CD	19	0	#N/D	L3	B2	#N/D	#N/D	0	15CD	Godon	2,32340956	25
V1.CD17.0	Spain	Male	21	Meta Hit	CD	21,68	0	#N/D	L3	B2	#N/D	#N/D	0	17CD	Godon	2,977077689	42
V1.CD18.0	Spain	Male	18	Meta Hit	CD	19,6	0	#N/D	L3	B3	#N/D	#N/D	0	18CD	Godon	2,041074233	23
V1.CD20.0	Spain	Male	26	Meta Hit	CD	19,6	1	#N/D	L3+L4	B3	#N/D	#N/D	0	20CD	Godon	2,558325572	41
V1.CD25.0	Spain	Female	41	Meta Hit	CD	16,94	0	#N/D	L3	B2	#N/D	#N/D	0	25CD	Godon	2,485875679	33
V1.CD32.0	Spain	Female	56	Meta Hit	CD	25,32	0	#N/D	L3	B2	#N/D	#N/D	0	32CD	Godon	2,758687629	42
V1.CD35.0	Spain	Female	27	Meta Hit	CD	28,72	1	#N/D	L1	B2	#N/D	#N/D	0	35CD	Godon	1,503950166	26
V1.CD41.0	Spain	Female	25	Meta Hit	CD	19,04	0	#N/D	L1+L4	B2	#N/D	#N/D	0	41CD	Godon	2,582872615	51
V1.CD44.0	Spain	Female	47	Meta Hit	CD	31,89	4	#N/D	L3	B2	#N/D	#N/D	0	44CD	Godon	1,531714519	46
V1.CD46.0	Spain	Male	30	Meta Hit	CD	22,99	0	#N/D	L3	B3	#N/D	#N/D	0	46CD	Godon	2,244826714	38
V1.CD48.0	Spain	Female	23	Meta Hit	CD	20,24	0	#N/D	L3	B2P	#N/D	#N/D	0	48CD	Godon	2,262510395	38

SampleID	country	gender	Age	study	disease	BMI	hbi	cai	cd localization	cd behaviour	uc extent	dysbiosisindex	Time point	patientID	extraction protocol	shannon	chao
V1.CD51.0	Spain	Male	29	MetaHit	CD	20,88	0	#N/D	L3	B3	#N/D	#N/D	0	51CD	Godon	3,211724501	70
V1.CD53.0	Spain	Female	47	MetaHit	CD	19,71	4	#N/D	L3	B2	#N/D	#N/D	0	53CD	Godon	1,898390025	38
V1.CD54.0	Spain	Female	27	MetaHit	CD	21,53	1	#N/D	L3	B2	#N/D	#N/D	0	54CD	Godon	2,593689897	43,5
V1.CD7.0.PT	Spain	Male	53	MetaHit	CD	24,2	4	#N/D	L1	B3	#N/D	#N/D	0	7PTCD	Godon	1,9413837	48,5
V1.CD9.0.PT	Spain	Female	45	MetaHit	CD	22,6	0	#N/D	L3	B3	#N/D	#N/D	0	9PTCD	Godon	0,69194211	16,25
V1.CD5.0.PT	Spain	Male	22	MetaHit	CD	26,59	1	#N/D	L3	B2	#N/D	#N/D	0	5PTCD	Godon	1,136919117	14
V1.CD26.0	Spain	Male	39	MetaHit	CD	26,81	5	#N/D	L3	B1	#N/D	#N/D	0	26CD	Godon	1,727693513	22
V1.CD37.0	Spain	Male	35	MetaHit	CD	19,84	0	#N/D	L1	B2P	#N/D	#N/D	0	37CD	Godon	2,804193894	40
V1.CD39.0	Spain	Female	20	MetaHit	CD	21,3	0	#N/D	L1	B1P	#N/D	#N/D	0	39CD	Godon	1,837912467	17
V1.CD1.3.PN	Spain	Female	54	MetaHit	CD	25,97	2	#N/D	L1	#N/D	#N/D	#N/D	3	1PNCD	Godon	3,139963618	54
V1.CD3.3.PN	Spain	Female	54	MetaHit	CD	20	1	#N/D	L1	#N/D	#N/D	#N/D	3	3PNCD	Godon	1,77077406	20
V1.CD1.5.PT	Spain	Female	26	MetaHit	CD	18,69	0	#N/D	L3	#N/D	#N/D	#N/D	5	1PTCD	Godon	2,663492784	40
V1.CD3.4.PT	Spain	Female	33	MetaHit	CD	27,34	0	#N/D	L3	#N/D	#N/D	#N/D	4	3PTCD	Godon	1,452256786	15
V1.CD6.4	Spain	Female	36	MetaHit	CD	19,77	1	#N/D	L3	#N/D	#N/D	#N/D	4	6CD	Godon	2,864021509	56
V1.CD7.4	Spain	Male	22	MetaHit	CD	19,49	0	#N/D	L1	#N/D	#N/D	#N/D	4	7CD	Godon	2,379583533	33

SampleID	country	gender	Age	study	disease	BMI	hbi	cai	cd localization	cd behaviour	uc extent	dysbiosisindex	Time point	patientID	extraction protocol	shannon	chao
V1.CD12.3	Spain	Female	41	MetaHit	CD	20,94	1	#N/D	L3	#N/D	#N/D	#N/D	3	12CD	Godon	1,880917086	37
V1.CD15.3	Spain	Female	34	MetaHit	CD	19,36	1	#N/D	L3	#N/D	#N/D	#N/D	3	15CD	Godon	2,806075505	37
V1.CD17.4	Spain	Male	21	MetaHit	CD	23,41	0	#N/D	L3	#N/D	#N/D	#N/D	4	17CD	Godon	2,871623612	44,33333333
V1.CD18.3	Spain	Male	18	MetaHit	CD	17,96	3	#N/D	L3	#N/D	#N/D	#N/D	3	18CD	Godon	1,694243155	28
V1.CD20.4	Spain	Male	26	MetaHit	CD	21,56	0	#N/D	L3+L4	#N/D	#N/D	#N/D	4	20CD	Godon	2,377162972	53
V1.CD21.4	Spain	Female	34	MetaHit	CD	19,05	2	#N/D	L3	#N/D	#N/D	#N/D	4	21CD	Godon	1,541551075	26
V1.CD25.4	Spain	Female	41	MetaHit	CD	17,31	0	#N/D	L3	#N/D	#N/D	#N/D	4	25CD	Godon	2,51969583	35
V1.CD32.4	Spain	Female	56	MetaHit	CD	24,52	3	#N/D	L3	#N/D	#N/D	#N/D	4	32CD	Godon	2,307105535	41
V1.CD35.1	Spain	Female	27	MetaHit	CD	29,41	5	#N/D	L1	#N/D	#N/D	#N/D	1	35CD	Godon	2,03268894	26
V1.CD37.4	Spain	Male	35	MetaHit	CD	22,23	0	#N/D	L1	#N/D	#N/D	#N/D	4	37CD	Godon	2,22172059	39
V1.CD41.3	Spain	Female	25	MetaHit	CD	20,35	4	#N/D	L1+L4	#N/D	#N/D	#N/D	3	41CD	Godon	2,520198259	57
V1.CD44.4	Spain	Female	47	MetaHit	CD	32,95	5	#N/D	L3	#N/D	#N/D	#N/D	4	44CD	Godon	1,939968752	48
V1.CD48.4	Spain	Female	23	MetaHit	CD	23,21	0	#N/D	L3	#N/D	#N/D	#N/D	4	48CD	Godon	2,441989564	41
V1.CD53.2	Spain	Female	47	MetaHit	CD	19,33	7	#N/D	L3	#N/D	#N/D	#N/D	2	53CD	Godon	1,932024999	29
V1.CD54.4	Spain	Female	27	MetaHit	CD	21,75	0	#N/D	L3	#N/D	#N/D	#N/D	4	54CD	Godon	2,317573341	31

SampleID	country	gender	Age	study	disease	BMI	hbi	cai	cd localization	cd behaviour	uc extent	dysbiosisindex	Time point	patientID	extraction protocol	shannon	chao
V1.CD3.5.PN	Spain	Female	54	Meta Hit	CD	20,03	1	#N/D	L1	#N/D	#N/D	#N/D	5	3PNCD	Godon	1,22178212	24,5
V1.CD5.4.PN	Spain	Female	35	Meta Hit	CD	30,12	2	#N/D	L1	#N/D	#N/D	#N/D	4	5PNCD	Godon	1,190318463	26
V1.CD7.6.PN	Spain	Male	51	Meta Hit	CD	24,45	1	#N/D	L1	#N/D	#N/D	#N/D	6	7PNCD	Godon	0,950107218	10
V1.CD26.4	Spain	Male	39	Meta Hit	CD	28,41	3	#N/D	L3	#N/D	#N/D	#N/D	4	26CD	Godon	2,550834139	31,5
CSM5MCWK_P	USA	Female	51	HMP2	CD	50,2	1	#N/D	L3	#N/D	#N/D	#N/D	0	C3010	Chemagic	2,382584973	48
CSM5MCXT	USA	Female	51	HMP2	CD	50,2	1	#N/D	L3	#N/D	#N/D	#N/D	2	C3010	Chemagic	2,450643244	37,5
CSM5MCXV	USA	Female	51	HMP2	CD	50,2	1	#N/D	L3	#N/D	#N/D	#N/D	4	C3010	Chemagic	2,35772815	43
CSM5MCXX_P	USA	Female	51	HMP2	CD	50,2	1	#N/D	L3	#N/D	#N/D	#N/D	5	C3010	Chemagic	2,426208256	47
CSM5MCXZ_P	USA	Female	51	HMP2	CD	50,2	2	#N/D	L3	#N/D	#N/D	#N/D	7	C3010	Chemagic	2,318647644	35
CSM5MCY2	USA	Female	51	HMP2	CD	50,2	3	#N/D	L3	#N/D	#N/D	#N/D	9	C3010	Chemagic	2,37578846	47
CSM67UCK	USA	Female	51	HMP2	CD	50,2	1	#N/D	L3	#N/D	#N/D	#N/D	16	C3010	Chemagic	2,460577997	31
CSM79HK9	USA	Female	51	HMP2	CD	50,2	1	#N/D	L3	#N/D	#N/D	#N/D	23	C3010	Chemagic	2,363954169	40
CSM79HKB	USA	Female	51	HMP2	CD	50,2	0	#N/D	L3	#N/D	#N/D	#N/D	26	C3010	Chemagic	2,124443038	31
CSM79HOF	USA	Female	51	HMP2	CD	50,2	0	#N/D	L3	#N/D	#N/D	#N/D	31	C3010	Chemagic	2,413893327	33
CSM79HOH	USA	Female	51	HMP2	CD	50,2	3	#N/D	L3	#N/D	#N/D	#N/D	33	C3010	Chemagic	2,168138384	45

SampleID	country	gender	Age	study	disease	BMI	hbi	cai	cd localization	cd behaviour	uc extent	dysbiosisindex	Time point	patientID	extraction protocol	shannon	chao
CSM7KOL4	USA	Female	51	HMP2	CD	50,2	0	#N/D	L3	#N/D	#N/D	#N/D	35	C3010	Chemagic	2,282597092	38
CSM7KOLA	USA	Female	51	HMP2	CD	50,2	2	#N/D	L3	#N/D	#N/D	#N/D	41	C3010	Chemagic	2,697097195	47
CSM7KOLE	USA	Female	51	HMP2	CD	50,2	2	#N/D	L3	#N/D	#N/D	#N/D	45	C3010	Chemagic	2,260289002	40
CSM5MCXF_P	USA	Female	37	HMP2	CD	46,7	1	#N/D	L1	#N/D	#N/D	#N/D	0	C3012	Chemagic	2,358028281	32
CSM5MCZB	USA	Female	37	HMP2	CD	46,7	3	#N/D	L1	#N/D	#N/D	#N/D	3	C3012	Chemagic	2,183984386	40
CSM5MCZD	USA	Female	37	HMP2	CD	46,7	0	#N/D	L1	#N/D	#N/D	#N/D	4	C3012	Chemagic	2,555484679	38
CSM5MCZF	USA	Female	37	HMP2	CD	46,7	0	#N/D	L1	#N/D	#N/D	#N/D	6	C3012	Chemagic	2,46583514	35
CSM67U9B	USA	Female	37	HMP2	CD	46,7	2	#N/D	L1	#N/D	#N/D	#N/D	8	C3012	Chemagic	2,586693064	39
CSM67U9D	USA	Female	37	HMP2	CD	46,7	0	#N/D	L1	#N/D	#N/D	#N/D	10	C3012	Chemagic	2,618973033	37
CSM67UGO	USA	Female	37	HMP2	CD	46,7	0	#N/D	L1	#N/D	#N/D	#N/D	16	C3012	Chemagic	2,39656249	35
CSM79HOJ	USA	Female	37	HMP2	CD	46,7	0	#N/D	L1	#N/D	#N/D	#N/D	24	C3012	Chemagic	2,071643079	39
CSM79HOL	USA	Female	37	HMP2	CD	46,7	1	#N/D	L1	#N/D	#N/D	#N/D	26	C3012	Chemagic	2,465026214	38
CSM79HOT	USA	Female	37	HMP2	CD	46,7	0	#N/D	L1	#N/D	#N/D	#N/D	34	C3012	Chemagic	2,444932223	37
CSM7KOMX	USA	Female	37	HMP2	CD	46,7	0	#N/D	L1	#N/D	#N/D	#N/D	36	C3012	Chemagic	2,337831429	34
CSM7KOMZ	USA	Female	37	HMP2	CD	46,7	2	#N/D	L1	#N/D	#N/D	#N/D	38	C3012	Chemagic	2,387050591	32

SampleID	country	gender	Age	study	disease	BMI	hbi	cai	cd localization	cd behaviour	uc extent	dysbiosisindex	Time point	patientID	extraction protocol	shannon	chao
CSM7KON2	USA	Female	37	HMP2	CD	46,7	1	#N/D	L1	#N/D	#N/D	#N/D	40	C3012	Chemagic	2,53060069	36
CSM7KON8	USA	Female	37	HMP2	CD	46,7	0	#N/D	L1	#N/D	#N/D	#N/D	46	C3012	Chemagic	2,548250583	35
CSM67U9X_P	USA	Male	45	HMP2	CD	24,2	2	#N/D	L3	#N/D	#N/D	#N/D	0	C3017	Chemagic	1,668828655	55
CSM67UB5_P	USA	Male	45	HMP2	CD	24,2	1	#N/D	L3	#N/D	#N/D	#N/D	2	C3017	Chemagic	2,31321275	41
CSM67UB7_P	USA	Male	45	HMP2	CD	24,2	1	#N/D	L3	#N/D	#N/D	#N/D	4	C3017	Chemagic	2,378440957	44
CSM67UB9_P	USA	Male	45	HMP2	CD	24,2	4	#N/D	L3	#N/D	#N/D	#N/D	6	C3017	Chemagic	1,217787011	24
CSM67UB9	USA	Male	45	HMP2	CD	24,2	4	#N/D	L3	#N/D	#N/D	#N/D	6	C3017	Chemagic	1,170338986	21
CSM67UBB	USA	Male	45	HMP2	CD	24,2	1	#N/D	L3	#N/D	#N/D	#N/D	9	C3017	Chemagic	1,698835242	30
CSM79HJ2_P	USA	Male	45	HMP2	CD	24,2	1	#N/D	L3	#N/D	#N/D	#N/D	12	C3017	Chemagic	1,76410778	29
CSM79HJ4_P	USA	Male	45	HMP2	CD	24,2	1	#N/D	L3	#N/D	#N/D	#N/D	14	C3017	Chemagic	1,873474762	32
CSM79HJ6_P	USA	Male	45	HMP2	CD	24,2	1	#N/D	L3	#N/D	#N/D	#N/D	16	C3017	Chemagic	2,418237825	38
CSM79HJ8_P	USA	Male	45	HMP2	CD	24,2	1	#N/D	L3	#N/D	#N/D	#N/D	18	C3017	Chemagic	2,612247354	38
CSM79HJA	USA	Male	45	HMP2	CD	24,2	1	#N/D	L3	#N/D	#N/D	#N/D	20	C3017	Chemagic	2,331022846	36
CSM79HJC_P	USA	Male	45	HMP2	CD	24,2	1	#N/D	L3	#N/D	#N/D	#N/D	22	C3017	Chemagic	2,117402164	48
CSM7KOKF	USA	Male	45	HMP2	CD	24,2	1	#N/D	L3	#N/D	#N/D	#N/D	24	C3017	Chemagic	2,068185112	31

SampleID	country	gender	Age	study	disease	BMI	hbi	cai	cd localization	cd behaviour	uc extent	dysbiosisindex	Time point	patientID	extraction protocol	shannon	chao
CSM7KOKH_P	USA	Male	45	HMP2	CD	24,2	1	#N/D	L3	#N/D	#N/D	#N/D	26	C3017	Chemagic	2,072414801	34
CSM7KOKJ	USA	Male	45	HMP2	CD	24,2	1	#N/D	L3	#N/D	#N/D	#N/D	28	C3017	Chemagic	1,808498001	35
CSM7KOKL_P	USA	Male	45	HMP2	CD	24,2	4	#N/D	L3	#N/D	#N/D	#N/D	30	C3017	Chemagic	2,513389728	49
CSM7KOKN	USA	Male	45	HMP2	CD	24,2	1	#N/D	L3	#N/D	#N/D	#N/D	32	C3017	Chemagic	2,08544445	33
CSM7KOKP_P	USA	Male	45	HMP2	CD	24,2	1	#N/D	L3	#N/D	#N/D	#N/D	34	C3017	Chemagic	2,095385229	38
CSM7KOQX	USA	Male	45	HMP2	CD	24,2	0	#N/D	L3	#N/D	#N/D	#N/D	37	C3017	Chemagic	2,529421333	32
CSM7KOQZ_P	USA	Male	45	HMP2	CD	24,2	1	#N/D	L3	#N/D	#N/D	#N/D	38	C3017	Chemagic	2,36225765	32
CSM7KOR2	USA	Male	45	HMP2	CD	24,2	1	#N/D	L3	#N/D	#N/D	#N/D	40	C3017	Chemagic	2,058784007	39
CSM7KOR4_P	USA	Male	45	HMP2	CD	24,2	1	#N/D	L3	#N/D	#N/D	#N/D	42	C3017	Chemagic	2,359551615	38
CSM7KOR8_P	USA	Male	45	HMP2	CD	24,2	5	#N/D	L3	#N/D	#N/D	#N/D	46	C3017	Chemagic	2,425188598	35
CSM79HHO	USA	Female	38	HMP2	CD	25,6	5	#N/D	L2	#N/D	#N/D	#N/D	1	C3021	Chemagic	2,473578529	47
CSM79HHM	USA	Female	38	HMP2	CD	25,6	3	#N/D	L2	#N/D	#N/D	#N/D	4	C3021	Chemagic	2,392989929	45
CSM79HHU	USA	Female	38	HMP2	CD	25,6	0	#N/D	L2	#N/D	#N/D	#N/D	7	C3021	Chemagic	2,348546148	37
CSM79HN2	USA	Female	38	HMP2	CD	25,6	0	#N/D	L2	#N/D	#N/D	#N/D	9	C3021	Chemagic	2,772121695	47
CSM79HN6	USA	Female	38	HMP2	CD	25,6	0	#N/D	L2	#N/D	#N/D	#N/D	13	C3021	Chemagic	2,387917197	47

SampleID	country	gender	Age	study	disease	BMI	hbi	cai	cd localization	cd behaviour	uc extent	dysbiosisindex	Time point	patientID	extraction protocol	shannon	chao
CSM7KOLK	USA	Female	38	HMP2	CD	25,6	2	#N/D	L2	#N/D	#N/D	#N/D	21	C3021	Chemagic	2,45913602	36
CSM7KOLM	USA	Female	38	HMP2	CD	25,6	0	#N/D	L2	#N/D	#N/D	#N/D	24	C3021	Chemagic	2,183557601	39
CSM7KOSV	USA	Female	38	HMP2	CD	25,6	1	#N/D	L2	#N/D	#N/D	#N/D	31	C3021	Chemagic	2,365077075	37
CSM7KOSX	USA	Female	38	HMP2	CD	25,6	0	#N/D	L2	#N/D	#N/D	#N/D	33	C3021	Chemagic	2,375643282	45
CSM79HH2_P	USA	Male	60	HMP2	CD	28	3	#N/D	L3	#N/D	#N/D	#N/D	2	C3023	Chemagic	2,840685997	59
CSM79HH4	USA	Male	60	HMP2	CD	28	2	#N/D	L3	#N/D	#N/D	#N/D	4	C3023	Chemagic	2,526901996	37
CSM79HH8	USA	Male	60	HMP2	CD	28	0	#N/D	L3	#N/D	#N/D	#N/D	8	C3023	Chemagic	2,624793249	46
CSM79HHA	USA	Male	60	HMP2	CD	28	2	#N/D	L3	#N/D	#N/D	#N/D	10	C3023	Chemagic	2,226714557	39
CSM79HPA_TR	USA	Male	60	HMP2	CD	28	1	#N/D	L3	#N/D	#N/D	#N/D	14	C3023	Chemagic	2,5237986	42
CSM79HPA	USA	Male	60	HMP2	CD	28	1	#N/D	L3	#N/D	#N/D	#N/D	14	C3023	Chemagic	2,674844428	50
CSM79HPC	USA	Male	60	HMP2	CD	28	1	#N/D	L3	#N/D	#N/D	#N/D	16	C3023	Chemagic	2,681965779	36
CSM7KONA	USA	Male	60	HMP2	CD	28	3	#N/D	L3	#N/D	#N/D	#N/D	24	C3023	Chemagic	2,437217547	25
CSM7KONK	USA	Male	60	HMP2	CD	28	3	#N/D	L3	#N/D	#N/D	#N/D	34	C3023	Chemagic	2,473266405	35
CSM7KOTA	USA	Male	60	HMP2	CD	28	3	#N/D	L3	#N/D	#N/D	#N/D	36	C3023	Chemagic	2,344112884	27
CSM7KOTC	USA	Male	60	HMP2	CD	28	1	#N/D	L3	#N/D	#N/D	#N/D	38	C3023	Chemagic	2,549711334	38

SampleID	country	gender	Age	study	disease	BMI	hbi	cai	cd localization	cd behaviour	uc extent	dysbiosisindex	Time point	patientID	extraction protocol	shannon	chao
CSM7KOTK	USA	Male	60	HMP2	CD	28	2	#N/D	L3	#N/D	#N/D	#N/D	46	C3023	Chemagic	2,60621799	44
CSM79HJI_P	USA	Male	44	HMP2	CD	21,2	1	#N/D	L2	#N/D	#N/D	#N/D	0	C3030	Chemagic	2,921483102	62
CSM79HNO	USA	Male	44	HMP2	CD	21,2	2	#N/D	L2	#N/D	#N/D	#N/D	1	C3030	Chemagic	1,824102234	10
CSM79HNU	USA	Male	44	HMP2	CD	21,2	2	#N/D	L2	#N/D	#N/D	#N/D	7	C3030	Chemagic	1,55420997	12
CSM79HNW	USA	Male	44	HMP2	CD	21,2	3	#N/D	L2	#N/D	#N/D	#N/D	9	C3030	Chemagic	1,690786448	8
CSM7KOMB	USA	Male	44	HMP2	CD	21,2	4	#N/D	L2	#N/D	#N/D	#N/D	16	C3030	Chemagic	2,591158026	44
CSM7KOMH	USA	Male	44	HMP2	CD	21,2	3	#N/D	L2	#N/D	#N/D	#N/D	21	C3030	Chemagic	1,864500539	15
CSM7KOSL	USA	Male	44	HMP2	CD	21,2	2	#N/D	L2	#N/D	#N/D	#N/D	25	C3030	Chemagic	1,660687747	12
CSM7KOSP	USA	Male	44	HMP2	CD	21,2	2	#N/D	L2	#N/D	#N/D	#N/D	29	C3030	Chemagic	2,283145408	21
CSM7KOST	USA	Male	44	HMP2	CD	21,2	2	#N/D	L2	#N/D	#N/D	#N/D	34	C3030	Chemagic	2,283226122	17
CSM7KOSJ	USA	Male	44	HMP2	CD	21,2	2	#N/D	L2	#N/D	#N/D	#N/D	37	C3030	Chemagic	2,167895661	14
CSM7KOTO	USA	Male	62	HMP2	CD	24,4	8	#N/D	L3	#N/D	#N/D	#N/D	4	C3035	Chemagic	2,235677045	25
CSM7KOTQ	USA	Male	62	HMP2	CD	24,4	5	#N/D	L3	#N/D	#N/D	#N/D	6	C3035	Chemagic	2,201566241	33
CSM7KOTS	USA	Male	62	HMP2	CD	24,4	7	#N/D	L3	#N/D	#N/D	#N/D	8	C3035	Chemagic	2,390050209	34
CSM7KOTU	USA	Male	62	HMP2	CD	24,4	9	#N/D	L3	#N/D	#N/D	#N/D	12	C3035	Chemagic	2,432643346	34

SampleID	country	gender	Age	study	disease	BMI	hbi	cai	cd localization	cd behaviour	uc extent	dysbiosisindex	Time point	patientID	extraction protocol	shannon	chao
CSM9X1YV	USA	Male	62	HMP2	CD	24,4	7	#N/D	L3	#N/D	#N/D	#N/D	16	C3035	Chemagic	2,302903329	33
CSM9X21R	USA	Male	62	HMP2	CD	24,4	7	#N/D	L3	#N/D	#N/D	#N/D	24	C3035	Chemagic	2,106555094	32
CSM9X21T	USA	Male	62	HMP2	CD	24,4	9	#N/D	L3	#N/D	#N/D	#N/D	26	C3035	Chemagic	2,348504633	35
CSM9X233	USA	Male	62	HMP2	CD	24,4	7	#N/D	L3	#N/D	#N/D	#N/D	36	C3035	Chemagic	2,116529463	34
CSM9X235	USA	Male	62	HMP2	CD	24,4	8	#N/D	L3	#N/D	#N/D	#N/D	38	C3035	Chemagic	2,44359362	36
CSM9X23B	USA	Male	62	HMP2	CD	24,4	5	#N/D	L3	#N/D	#N/D	#N/D	44	C3035	Chemagic	2,437093333	36
ESM5MEDN	USA	Female	17	HMP2	CD	17,6	1	#N/D	L1	#N/D	#N/D	#N/D	2	E5009	Chemagic	2,175468843	45
ESM5MEDP_P	USA	Female	17	HMP2	CD	17,6	2	#N/D	L1	#N/D	#N/D	#N/D	4	E5009	Chemagic	1,727437016	48
ESM5MEDK	USA	Female	17	HMP2	CD	17,6	2	#N/D	L1	#N/D	#N/D	#N/D	7	E5009	Chemagic	2,33175152	53
ESM5MEDD	USA	Female	17	HMP2	CD	17,6	2	#N/D	L1	#N/D	#N/D	#N/D	9	E5009	Chemagic	2,320814949	55
ESM5MEDF	USA	Female	17	HMP2	CD	17,6	2	#N/D	L1	#N/D	#N/D	#N/D	11	E5009	Chemagic	3,046990228	54
ESM718UH	USA	Female	17	HMP2	CD	17,6	2	#N/D	L1	#N/D	#N/D	#N/D	15	E5009	Chemagic	2,094959753	54
ESM7F5AK	USA	Female	17	HMP2	CD	17,6	1	#N/D	L1	#N/D	#N/D	#N/D	25	E5009	Chemagic	2,587318161	55
ESM7F5AM	USA	Female	17	HMP2	CD	17,6	7	#N/D	L1	#N/D	#N/D	#N/D	26	E5009	Chemagic	2,064950543	52
ESM7F5CB	USA	Female	17	HMP2	CD	17,6	4	#N/D	L1	#N/D	#N/D	#N/D	34	E5009	Chemagic	1,834575385	38

SampleID	country	gender	Age	study	disease	BMI	hbi	cai	cd localization	cd behaviour	uc extent	dysbiosisindex	Time point	patientID	extraction protocol	shannon	chao
ESM7F5CD	USA	Female	17	HMP2	CD	17,6	3	#N/D	L1	#N/D	#N/D	#N/D	36	E5009	Chemagic	2,152153107	45
ESM7F5CF	USA	Female	17	HMP2	CD	17,6	3	#N/D	L1	#N/D	#N/D	#N/D	38	E5009	Chemagic	1,913623155	45
ESM5MEB9_P	USA	Female	15	HMP2	CD	22,4	1	#N/D	L1	#N/D	#N/D	#N/D	0	E5013	Chemagic	2,40465974	42
ESM5MEB7	USA	Female	15	HMP2	CD	22,4	4	#N/D	L1	#N/D	#N/D	#N/D	1	E5013	Chemagic	1,674913203	49
ESM5MED2	USA	Female	15	HMP2	CD	22,4	6	#N/D	L1	#N/D	#N/D	#N/D	3	E5013	Chemagic	1,348906484	52
ESM718V8	USA	Female	15	HMP2	CD	22,4	0	#N/D	L1	#N/D	#N/D	#N/D	7	E5013	Chemagic	1,432203339	52
ESM718V4	USA	Female	15	HMP2	CD	22,4	11	#N/D	L1	#N/D	#N/D	#N/D	9	E5013	Chemagic	0,848563948	49
ESM718TK	USA	Female	15	HMP2	CD	22,4	10	#N/D	L1	#N/D	#N/D	#N/D	14	E5013	Chemagic	0,641919018	42
ESM718TM	USA	Female	15	HMP2	CD	22,4	0	#N/D	L1	#N/D	#N/D	#N/D	16	E5013	Chemagic	1,268997978	39
ESM718TF	USA	Female	15	HMP2	CD	22,4	8	#N/D	L1	#N/D	#N/D	#N/D	21	E5013	Chemagic	1,217105294	51
ESM718T9	USA	Female	15	HMP2	CD	22,4	2	#N/D	L1	#N/D	#N/D	#N/D	23	E5013	Chemagic	1,183671195	44
ESM718T7	USA	Female	15	HMP2	CD	22,4	0	#N/D	L1	#N/D	#N/D	#N/D	26	E5013	Chemagic	1,166359749	44
ESM7F5C5	USA	Female	15	HMP2	CD	22,4	0	#N/D	L1	#N/D	#N/D	#N/D	29	E5013	Chemagic	1,481354923	41
ESM7F5C7	USA	Female	15	HMP2	CD	22,4	0	#N/D	L1	#N/D	#N/D	#N/D	31	E5013	Chemagic	1,580779469	50
ESM9IEP1	USA	Female	15	HMP2	CD	22,4	4	#N/D	L1	#N/D	#N/D	#N/D	39	E5013	Chemagic	1,445897267	36

SampleID	country	gender	Age	study	disease	BMI	hbi	cai	cd localization	cd behaviour	uc extent	dysbiosisindex	Time point	patientID	extraction protocol	shannon	chao
HSM5FZBQ_P	USA	Male	14	HMP2	CD	16,6	1	#N/D	L3+L4	#N/D	#N/D	#N/D	5	H4001	Chemagic	1,537486039	19
HSM5FZBR_P	USA	Male	14	HMP2	CD	16,6	0	#N/D	L3+L4	#N/D	#N/D	#N/D	7	H4001	Chemagic	1,39260556	30
HSM5FZBP_P	USA	Male	14	HMP2	CD	16,6	0	#N/D	L3+L4	#N/D	#N/D	#N/D	10	H4001	Chemagic	1,500589554	35
HSM5MD7W_P	USA	Male	14	HMP2	CD	16,6	0	#N/D	L3+L4	#N/D	#N/D	#N/D	11	H4001	Chemagic	0,862678592	34
HSM5MD5H_P	USA	Male	14	HMP2	CD	16,6	0	#N/D	L3+L4	#N/D	#N/D	#N/D	13	H4001	Chemagic	1,70211669	19
HSM5MD5K_P	USA	Male	14	HMP2	CD	16,6	0	#N/D	L3+L4	#N/D	#N/D	#N/D	15	H4001	Chemagic	0,593014297	23
HSM5FZC2_P	USA	Male	14	HMP2	CD	16,6	0	#N/D	L3+L4	#N/D	#N/D	#N/D	17	H4001	Chemagic	1,475203276	28
HSM5FZBZ	USA	Male	14	HMP2	CD	16,6	2	#N/D	L3+L4	#N/D	#N/D	#N/D	20	H4001	Chemagic	1,059921921	27
HSM5MD7J	USA	Male	14	HMP2	CD	16,6	0	#N/D	L3+L4	#N/D	#N/D	#N/D	23	H4001	Chemagic	0,753962361	28
HSM5MD79	USA	Male	14	HMP2	CD	16,6	0	#N/D	L3+L4	#N/D	#N/D	#N/D	33	H4001	Chemagic	1,882610818	23
HSM67VDZ	USA	Male	14	HMP2	CD	16,6	0	#N/D	L3+L4	#N/D	#N/D	#N/D	36	H4001	Chemagic	0,429070141	18
HSM67VE4	USA	Male	14	HMP2	CD	16,6	1	#N/D	L3+L4	#N/D	#N/D	#N/D	37	H4001	Chemagic	0,830593455	24
HSM5MD4U_P	USA	Male	14	HMP2	CD	18,6	5	#N/D	L3	#N/D	#N/D	#N/D	2	H4004	Chemagic	0,695728867	15
HSM5MD4W_P	USA	Male	14	HMP2	CD	18,6	2	#N/D	L3	#N/D	#N/D	#N/D	4	H4004	Chemagic	0,494343724	22
HSM5MD4Y	USA	Male	14	HMP2	CD	18,6	3	#N/D	L3	#N/D	#N/D	#N/D	6	H4004	Chemagic	1,872948868	21

SampleID	country	gender	Age	study	disease	BMI	hbi	cai	cd localization	cd behaviour	uc extent	dysbiosisindex	Time point	patientID	extraction protocol	shannon	chao
HSM5MD53	USA	Male	14	HMP2	CD	18,6	4	#N/D	L3	#N/D	#N/D	#N/D	10	H4004	Chemagic	1,867979058	23
HSM5MD5P	USA	Male	14	HMP2	CD	18,6	0	#N/D	L3	#N/D	#N/D	#N/D	16	H4004	Chemagic	2,028050946	25
HSM6XRSG	USA	Male	14	HMP2	CD	18,6	1	#N/D	L3	#N/D	#N/D	#N/D	24	H4004	Chemagic	0,234662005	6
HSM6XRSI	USA	Male	14	HMP2	CD	18,6	0	#N/D	L3	#N/D	#N/D	#N/D	26	H4004	Chemagic	0,632983074	9
HSM67VDP	USA	Male	14	HMP2	CD	18,6	3	#N/D	L3	#N/D	#N/D	#N/D	34	H4004	Chemagic	0,974777588	6
HSM7CYZT	USA	Male	14	HMP2	CD	18,6	5	#N/D	L3	#N/D	#N/D	#N/D	36	H4004	Chemagic	0,821011981	8
HSM7CYZV	USA	Male	14	HMP2	CD	18,6	4	#N/D	L3	#N/D	#N/D	#N/D	38	H4004	Chemagic	0,405574795	9
HSM7CZ14	USA	Male	14	HMP2	CD	18,6	5	#N/D	L3	#N/D	#N/D	#N/D	46	H4004	Chemagic	0,964064124	13
HSM5MD4B_P	USA	Male	8	HMP2	CD	26,6	3	#N/D	L2+L4	#N/D	#N/D	#N/D	4	H4006	Chemagic	1,823179373	29
HSM5MD4A_P	USA	Male	8	HMP2	CD	26,6	0	#N/D	L2+L4	#N/D	#N/D	#N/D	6	H4006	Chemagic	1,974116126	32
HSM5MD49_P	USA	Male	8	HMP2	CD	26,6	1	#N/D	L2+L4	#N/D	#N/D	#N/D	8	H4006	Chemagic	2,030663499	38
HSM5MD48	USA	Male	8	HMP2	CD	26,6	2	#N/D	L2+L4	#N/D	#N/D	#N/D	10	H4006	Chemagic	1,780600237	27
HSM5MD7K	USA	Male	8	HMP2	CD	26,6	2	#N/D	L2+L4	#N/D	#N/D	#N/D	12	H4006	Chemagic	2,324159607	40
HSM5MD7M	USA	Male	8	HMP2	CD	26,6	7	#N/D	L2+L4	#N/D	#N/D	#N/D	14	H4006	Chemagic	1,776952362	23
HSM5MD7O	USA	Male	8	HMP2	CD	26,6	8	#N/D	L2+L4	#N/D	#N/D	#N/D	16	H4006	Chemagic	1,346028203	5

SampleID	country	gender	Age	study	disease	BMI	hbi	cai	cd localization	cd behaviour	uc extent	dysbiosisindex	Time point	patientID	extraction protocol	shannon	chao
HSM5MD7Q	USA	Male	8	HMP2	CD	26,6	6	#N/D	L2+L4	#N/D	#N/D	#N/D	18	H4006	Chemagic	0,702297025	5
HSM5MD7S	USA	Male	8	HMP2	CD	26,6	2	#N/D	L2+L4	#N/D	#N/D	#N/D	20	H4006	Chemagic	1,132315288	21
HSM5MD7U	USA	Male	8	HMP2	CD	26,6	1	#N/D	L2+L4	#N/D	#N/D	#N/D	22	H4006	Chemagic	1,657686119	32
HSM67VEC	USA	Male	8	HMP2	CD	26,6	0	#N/D	L2+L4	#N/D	#N/D	#N/D	24	H4006	Chemagic	0,789933853	28
HSM67VEE	USA	Male	8	HMP2	CD	26,6	0	#N/D	L2+L4	#N/D	#N/D	#N/D	26	H4006	Chemagic	1,530793295	29
HSM67VEG	USA	Male	8	HMP2	CD	26,6	4	#N/D	L2+L4	#N/D	#N/D	#N/D	28	H4006	Chemagic	2,322763716	30
HSM67VEI	USA	Male	8	HMP2	CD	26,6	0	#N/D	L2+L4	#N/D	#N/D	#N/D	32	H4006	Chemagic	2,05763759	31
HSM67VEK	USA	Male	8	HMP2	CD	26,6	0	#N/D	L2+L4	#N/D	#N/D	#N/D	33	H4006	Chemagic	1,910124598	31
HSM67VEM	USA	Male	8	HMP2	CD	26,6	0	#N/D	L2+L4	#N/D	#N/D	#N/D	34	H4006	Chemagic	2,09766299	34
HSM67VEM_TR	USA	Male	8	HMP2	CD	26,6	0	#N/D	L2+L4	#N/D	#N/D	#N/D	34	H4006	Chemagic	2,028771967	33
HSM7CYX2	USA	Male	8	HMP2	CD	26,6	0	#N/D	L2+L4	#N/D	#N/D	#N/D	36	H4006	Chemagic	2,294144544	40
HSM7CYX4	USA	Male	8	HMP2	CD	26,6	0	#N/D	L2+L4	#N/D	#N/D	#N/D	38	H4006	Chemagic	2,227654399	35
HSM7CYX6	USA	Male	8	HMP2	CD	26,6	0	#N/D	L2+L4	#N/D	#N/D	#N/D	40	H4006	Chemagic	2,508412262	36
HSM7CYX8	USA	Male	8	HMP2	CD	26,6	1	#N/D	L2+L4	#N/D	#N/D	#N/D	42	H4006	Chemagic	2,284600869	35
HSM7CYXA	USA	Male	8	HMP2	CD	26,6	0	#N/D	L2+L4	#N/D	#N/D	#N/D	44	H4006	Chemagic	2,166299686	36

SampleID	country	gender	Age	study	disease	BMI	hbi	cai	cd localization	cd behaviour	uc extent	dysbiosisindex	Time point	patientID	extraction protocol	shannon	chao
HSM7CYXC	USA	Male	8	HMP2	CD	26,6	1	#N/D	L2+L4	#N/D	#N/D	#N/D	46	H4006	Chemagic	2,023505439	34
HSM5MD7Z_P	USA	Female	15	HMP2	CD	20,4	3	#N/D	L3+L4	#N/D	#N/D	#N/D	0	H4007	Chemagic	2,065124201	43
HSM5MD4P_P	USA	Female	15	HMP2	CD	20,4	4	#N/D	L3+L4	#N/D	#N/D	#N/D	6	H4007	Chemagic	1,957635003	42
HSM5MD4O	USA	Female	15	HMP2	CD	20,4	2	#N/D	L3+L4	#N/D	#N/D	#N/D	9	H4007	Chemagic	2,15638069	46
HSM5MD4N	USA	Female	15	HMP2	CD	20,4	2	#N/D	L3+L4	#N/D	#N/D	#N/D	10	H4007	Chemagic	2,035894083	40
HSM6XRSN	USA	Female	15	HMP2	CD	20,4	1	#N/D	L3+L4	#N/D	#N/D	#N/D	16	H4007	Chemagic	1,931845456	43
HSM6XRST	USA	Female	15	HMP2	CD	20,4	1	#N/D	L3+L4	#N/D	#N/D	#N/D	22	H4007	Chemagic	2,068010502	46,5
HSM67VHQ	USA	Female	15	HMP2	CD	20,4	1	#N/D	L3+L4	#N/D	#N/D	#N/D	24	H4007	Chemagic	2,094313151	38
HSM67VHS	USA	Female	15	HMP2	CD	20,4	1	#N/D	L3+L4	#N/D	#N/D	#N/D	26	H4007	Chemagic	1,917122003	38
HSM67VHW	USA	Female	15	HMP2	CD	20,4	1	#N/D	L3+L4	#N/D	#N/D	#N/D	31	H4007	Chemagic	2,062178996	42
HSM67VI1	USA	Female	15	HMP2	CD	20,4	1	#N/D	L3+L4	#N/D	#N/D	#N/D	34	H4007	Chemagic	2,265352672	61
HSM7CYXQ	USA	Female	15	HMP2	CD	20,4	1	#N/D	L3+L4	#N/D	#N/D	#N/D	37	H4007	Chemagic	2,402092127	43
HSM7CYXS	USA	Female	15	HMP2	CD	20,4	1	#N/D	L3+L4	#N/D	#N/D	#N/D	38	H4007	Chemagic	2,468190367	42
HSM5MD3L_P	USA	Female	10	HMP2	CD	19,6	0	#N/D	L3+L4	#N/D	#N/D	#N/D	0	H4014	Chemagic	2,788899705	39
HSM5MD66	USA	Female	10	HMP2	CD	19,6	2	#N/D	L3+L4	#N/D	#N/D	#N/D	4	H4014	Chemagic	2,307441686	30

SampleID	country	gender	Age	study	disease	BMI	hbi	cai	cd localization	cd behaviour	uc extent	dysbiosisindex	Time point	patientID	extraction protocol	shannon	chao
HSM5MD6A	USA	Female	10	HMP2	CD	19,6	1	#N/D	L3+L4	#N/D	#N/D	#N/D	8	H4014	Chemagic	2,65367128	38
HSM5MD6A_TR	USA	Female	10	HMP2	CD	19,6	1	#N/D	L3+L4	#N/D	#N/D	#N/D	8	H4014	Chemagic	2,593009037	41
HSM5MD6C	USA	Female	10	HMP2	CD	19,6	1	#N/D	L3+L4	#N/D	#N/D	#N/D	10	H4014	Chemagic	2,703053248	41
HSM6XRRV	USA	Female	10	HMP2	CD	19,6	3	#N/D	L3+L4	#N/D	#N/D	#N/D	16	H4014	Chemagic	2,615460116	40
HSM6XRS2	USA	Female	10	HMP2	CD	19,6	2	#N/D	L3+L4	#N/D	#N/D	#N/D	23	H4014	Chemagic	2,752449949	34
HSM6XRVM	USA	Female	10	HMP2	CD	19,6	0	#N/D	L3+L4	#N/D	#N/D	#N/D	25	H4014	Chemagic	2,804529545	40
HSM6XRVO	USA	Female	10	HMP2	CD	19,6	0	#N/D	L3+L4	#N/D	#N/D	#N/D	27	H4014	Chemagic	2,050757828	35
HSM6XRVU	USA	Female	10	HMP2	CD	19,6	0	#N/D	L3+L4	#N/D	#N/D	#N/D	36	H4014	Chemagic	2,561906819	38
HSM6XRVW	USA	Female	10	HMP2	CD	19,6	3	#N/D	L3+L4	#N/D	#N/D	#N/D	38	H4014	Chemagic	2,376519323	39
HSM7J4QT	USA	Female	10	HMP2	CD	19,6	0	#N/D	L3+L4	#N/D	#N/D	#N/D	50	H4014	Chemagic	2,522903033	37
HSM5MD5X_P	USA	Male	15	HMP2	CD	19,2	8	#N/D	L3+L4	#N/D	#N/D	#N/D	0	H4015	Chemagic	2,316778052	57,5
HSM5MD62	USA	Male	15	HMP2	CD	19,2	1	#N/D	L3+L4	#N/D	#N/D	#N/D	2	H4015	Chemagic	2,155052126	41
HSM5MD6Y	USA	Male	15	HMP2	CD	19,2	4	#N/D	L3+L4	#N/D	#N/D	#N/D	4	H4015	Chemagic	2,182076737	41
HSM5MD71	USA	Male	15	HMP2	CD	19,2	1	#N/D	L3+L4	#N/D	#N/D	#N/D	6	H4015	Chemagic	2,340751005	38
HSM5MD73	USA	Male	15	HMP2	CD	19,2	5	#N/D	L3+L4	#N/D	#N/D	#N/D	8	H4015	Chemagic	2,405843618	44

SampleID	country	gender	Age	study	disease	BMI	hbi	cai	cd localization	cd behaviour	uc extent	dysbiosisindex	Time point	patientID	extraction protocol	shannon	chao
HSM5MD75	USA	Male	15	HMP2	CD	19,2	3	#N/D	L3+L4	#N/D	#N/D	#N/D	11	H4015	Chemagic	1,896691255	25
HSM6XRS4	USA	Male	15	HMP2	CD	19,2	2	#N/D	L3+L4	#N/D	#N/D	#N/D	12	H4015	Chemagic	0,788976072	5
HSM6XRS6	USA	Male	15	HMP2	CD	19,2	2	#N/D	L3+L4	#N/D	#N/D	#N/D	14	H4015	Chemagic	1,088108371	6
HSM6XRS8	USA	Male	15	HMP2	CD	19,2	3	#N/D	L3+L4	#N/D	#N/D	#N/D	16	H4015	Chemagic	1,278259044	5
HSM6XRSE	USA	Male	15	HMP2	CD	19,2	1	#N/D	L3+L4	#N/D	#N/D	#N/D	22	H4015	Chemagic	2,083139137	30
HSM7CYZ5	USA	Male	15	HMP2	CD	19,2	1	#N/D	L3+L4	#N/D	#N/D	#N/D	24	H4015	Chemagic	1,462975616	16
HSM7CYZ7	USA	Male	15	HMP2	CD	19,2	2	#N/D	L3+L4	#N/D	#N/D	#N/D	26	H4015	Chemagic	1,603720809	17
HSM7CYZ9	USA	Male	15	HMP2	CD	19,2	0	#N/D	L3+L4	#N/D	#N/D	#N/D	28	H4015	Chemagic	1,664563837	27
HSM7CYZB	USA	Male	15	HMP2	CD	19,2	0	#N/D	L3+L4	#N/D	#N/D	#N/D	30	H4015	Chemagic	1,661541173	19
HSM7CYZD	USA	Male	15	HMP2	CD	19,2	1	#N/D	L3+L4	#N/D	#N/D	#N/D	32	H4015	Chemagic	1,782097887	17
HSM7CYZF	USA	Male	15	HMP2	CD	19,2	1	#N/D	L3+L4	#N/D	#N/D	#N/D	34	H4015	Chemagic	2,244818484	29
HSM7J4QB	USA	Male	15	HMP2	CD	19,2	0	#N/D	L3+L4	#N/D	#N/D	#N/D	36	H4015	Chemagic	2,107260004	19
HSM7J4QD	USA	Male	15	HMP2	CD	19,2	3	#N/D	L3+L4	#N/D	#N/D	#N/D	38	H4015	Chemagic	1,749223777	14
HSM7J4QL	USA	Male	15	HMP2	CD	19,2	5	#N/D	L3+L4	#N/D	#N/D	#N/D	46	H4015	Chemagic	2,096902779	24
HSM6XRQB_P	USA	Female	16	HMP2	CD	28,6	6	#N/D	L3+L4	#N/D	#N/D	#N/D	0	H4017	Chemagic	2,664344951	29

SampleID	country	gender	Age	study	disease	BMI	hbi	cai	cd localization	cd behaviour	uc extent	dysbiosisindex	Time point	patientID	extraction protocol	shannon	chao
HSM6XRQB	USA	Female	16	HMP2	CD	28,6	6	#N/D	L3+L4	#N/D	#N/D	#N/D	0	H4017	Chemagic	2,610364583	28
HSM67VHK	USA	Female	16	HMP2	CD	28,6	14	#N/D	L3+L4	#N/D	#N/D	#N/D	2	H4017	Chemagic	1,264863515	8
HSM6XRQI	USA	Female	16	HMP2	CD	28,6	3	#N/D	L3+L4	#N/D	#N/D	#N/D	4	H4017	Chemagic	1,920627907	21
HSM6XRQK	USA	Female	16	HMP2	CD	28,6	4	#N/D	L3+L4	#N/D	#N/D	#N/D	5	H4017	Chemagic	1,756836701	23
HSM6XRQM	USA	Female	16	HMP2	CD	28,6	2	#N/D	L3+L4	#N/D	#N/D	#N/D	9	H4017	Chemagic	1,865439615	30
HSM6XRQO	USA	Female	16	HMP2	CD	28,6	4	#N/D	L3+L4	#N/D	#N/D	#N/D	11	H4017	Chemagic	2,070625623	28
HSM67VF9	USA	Female	16	HMP2	CD	28,6	9	#N/D	L3+L4	#N/D	#N/D	#N/D	13	H4017	Chemagic	1,110092144	13
HSM67VFD	USA	Female	16	HMP2	CD	28,6	6	#N/D	L3+L4	#N/D	#N/D	#N/D	19	H4017	Chemagic	0,52825946	2
HSM67VFF	USA	Female	16	HMP2	CD	28,6	6	#N/D	L3+L4	#N/D	#N/D	#N/D	21	H4017	Chemagic	1,660383068	24
HSM67VFH	USA	Female	16	HMP2	CD	28,6	7	#N/D	L3+L4	#N/D	#N/D	#N/D	23	H4017	Chemagic	1,80204264	15
HSM67VFJ	USA	Female	16	HMP2	CD	28,6	3	#N/D	L3+L4	#N/D	#N/D	#N/D	25	H4017	Chemagic	1,856347726	17
HSM7CYY3	USA	Female	16	HMP2	CD	28,6	4	#N/D	L3+L4	#N/D	#N/D	#N/D	26	H4017	Chemagic	1,998766935	16
HSM7CYY5	USA	Female	16	HMP2	CD	28,6	5	#N/D	L3+L4	#N/D	#N/D	#N/D	29	H4017	Chemagic	2,320519464	53
HSM7CYY7	USA	Female	16	HMP2	CD	28,6	4	#N/D	L3+L4	#N/D	#N/D	#N/D	30	H4017	Chemagic	1,890779953	26
HSM7CYYD	USA	Female	16	HMP2	CD	28,6	9	#N/D	L3+L4	#N/D	#N/D	#N/D	34	H4017	Chemagic	2,178713089	35

SampleID	country	gender	Age	study	disease	BMI	hbi	cai	cd localization	cd behaviour	uc extent	dysbiosisindex	Time point	patientID	extraction protocol	shannon	chao
HSM7CYY9	USA	Female	16	HMP2	CD	28,6	3	#N/D	L3+L4	#N/D	#N/D	#N/D	36	H4017	Chemagic	1,971520127	33
HSM7CYYB	USA	Female	16	HMP2	CD	28,6	13	#N/D	L3+L4	#N/D	#N/D	#N/D	37	H4017	Chemagic	0,145688053	8
HSM6XRR3	USA	Male	13	HMP2	CD	27,7	3	#N/D	L1+L4	#N/D	#N/D	#N/D	0	H4020	Chemagic	2,072463051	29
HSM67VCX_P	USA	Male	13	HMP2	CD	27,7	2	#N/D	L1+L4	#N/D	#N/D	#N/D	2	H4020	Chemagic	1,753275985	23
HSM67VCZ	USA	Male	13	HMP2	CD	27,7	0	#N/D	L1+L4	#N/D	#N/D	#N/D	4	H4020	Chemagic	2,008126775	37
HSM67VD2	USA	Male	13	HMP2	CD	27,7	2	#N/D	L1+L4	#N/D	#N/D	#N/D	6	H4020	Chemagic	2,186596658	33
HSM67VD4	USA	Male	13	HMP2	CD	27,7	0	#N/D	L1+L4	#N/D	#N/D	#N/D	8	H4020	Chemagic	1,638498499	31
HSM67VD6	USA	Male	13	HMP2	CD	27,7	1	#N/D	L1+L4	#N/D	#N/D	#N/D	10	H4020	Chemagic	2,208397485	35
HSM67VHB	USA	Male	13	HMP2	CD	27,7	1	#N/D	L1+L4	#N/D	#N/D	#N/D	13	H4020	Chemagic	2,202339574	37
HSM67VHD	USA	Male	13	HMP2	CD	27,7	2	#N/D	L1+L4	#N/D	#N/D	#N/D	14	H4020	Chemagic	2,41936594	33
HSM67VHF	USA	Male	13	HMP2	CD	27,7	1	#N/D	L1+L4	#N/D	#N/D	#N/D	16	H4020	Chemagic	2,267594177	35
HSM67VHH	USA	Male	13	HMP2	CD	27,7	4	#N/D	L1+L4	#N/D	#N/D	#N/D	18	H4020	Chemagic	2,27580286	35
HSM67VHJ	USA	Male	13	HMP2	CD	27,7	2	#N/D	L1+L4	#N/D	#N/D	#N/D	20	H4020	Chemagic	2,275243583	36
HSM6XRUV	USA	Male	13	HMP2	CD	27,7	2	#N/D	L1+L4	#N/D	#N/D	#N/D	22	H4020	Chemagic	2,196224307	34
HSM7J4PO	USA	Male	13	HMP2	CD	27,7	2	#N/D	L1+L4	#N/D	#N/D	#N/D	24	H4020	Chemagic	2,699095143	37

SampleID	country	gender	Age	study	disease	BMI	hbi	cai	cd localization	cd behaviour	uc extent	dysbiosisindex	Time point	patientID	extraction protocol	shannon	chao
HSM7J4PQ	USA	Male	13	HMP2	CD	27,7	5	#N/D	L1+L4	#N/D	#N/D	#N/D	26	H4020	Chemagic	2,030461448	33
HSM7J4PS	USA	Male	13	HMP2	CD	27,7	2	#N/D	L1+L4	#N/D	#N/D	#N/D	28	H4020	Chemagic	2,360243588	34
HSM7J4PU	USA	Male	13	HMP2	CD	27,7	1	#N/D	L1+L4	#N/D	#N/D	#N/D	30	H4020	Chemagic	2,074766199	27
HSM7J4PW	USA	Male	13	HMP2	CD	27,7	0	#N/D	L1+L4	#N/D	#N/D	#N/D	32	H4020	Chemagic	2,588596852	37
HSM7J4PY	USA	Male	13	HMP2	CD	27,7	1	#N/D	L1+L4	#N/D	#N/D	#N/D	35	H4020	Chemagic	2,175686384	34
HSM7J4IO	USA	Male	13	HMP2	CD	27,7	2	#N/D	L1+L4	#N/D	#N/D	#N/D	37	H4020	Chemagic	1,957369478	43
HSM7J4IP	USA	Male	13	HMP2	CD	27,7	1	#N/D	L1+L4	#N/D	#N/D	#N/D	38	H4020	Chemagic	2,38826343	32
HSM7J4IQ	USA	Male	13	HMP2	CD	27,7	1	#N/D	L1+L4	#N/D	#N/D	#N/D	40	H4020	Chemagic	2,254277522	46
HSM7J4IR	USA	Male	13	HMP2	CD	27,7	2	#N/D	L1+L4	#N/D	#N/D	#N/D	42	H4020	Chemagic	2,275644854	41
HSM7J4IS	USA	Male	13	HMP2	CD	27,7	2	#N/D	L1+L4	#N/D	#N/D	#N/D	44	H4020	Chemagic	2,148312359	38
HSM67VID	USA	Female	13	HMP2	CD	15,9	0	#N/D	L1+L4	#N/D	#N/D	#N/D	1	H4028	Chemagic	1,301570566	31
HSM67VIF	USA	Female	13	HMP2	CD	15,9	0	#N/D	L1+L4	#N/D	#N/D	#N/D	3	H4028	Chemagic	1,413329306	38
HSM67VIJ_P	USA	Female	13	HMP2	CD	15,9	0	#N/D	L1+L4	#N/D	#N/D	#N/D	8	H4028	Chemagic	1,687857776	45
HSM67VIL	USA	Female	13	HMP2	CD	15,9	2	#N/D	L1+L4	#N/D	#N/D	#N/D	9	H4028	Chemagic	0,752169848	26,33333333
HSM7J4GD	USA	Female	13	HMP2	CD	15,9	1	#N/D	L1+L4	#N/D	#N/D	#N/D	15	H4028	Chemagic	1,722168255	32,5

SampleID	country	gender	Age	study	disease	BMI	hbi	cai	cd localization	cd behaviour	uc extent	dysbiosisindex	Time point	patientID	extraction protocol	shannon	chao
HSM7J4MY	USA	Female	13	HMP2	CD	15,9	2	#N/D	L1+L4	#N/D	#N/D	#N/D	24	H4028	Chemagic	1,709039793	32
HSM7J4N4	USA	Female	13	HMP2	CD	15,9	1	#N/D	L1+L4	#N/D	#N/D	#N/D	28	H4028	Chemagic	2,219951271	37
HSM7J4N6	USA	Female	13	HMP2	CD	15,9	0	#N/D	L1+L4	#N/D	#N/D	#N/D	32	H4028	Chemagic	1,009185484	35
HSM7J4NA	USA	Female	13	HMP2	CD	15,9	0	#N/D	L1+L4	#N/D	#N/D	#N/D	34	H4028	Chemagic	2,380190661	38
HSM7CYWS_P	USA	Male	16	HMP2	CD	21,1	5	#N/D	L1+L4	#N/D	#N/D	#N/D	0	H4030	Chemagic	2,41193606	64
HSM7CZ2Z	USA	Male	16	HMP2	CD	21,1	0	#N/D	L1+L4	#N/D	#N/D	#N/D	1	H4030	Chemagic	2,376870943	32
HSM7CZ32	USA	Male	16	HMP2	CD	21,1	0	#N/D	L1+L4	#N/D	#N/D	#N/D	4	H4030	Chemagic	1,763684587	24
HSM7CZ36	USA	Male	16	HMP2	CD	21,1	2	#N/D	L1+L4	#N/D	#N/D	#N/D	8	H4030	Chemagic	2,00957961	21
HSM7J4ON	USA	Male	16	HMP2	CD	21,1	0	#N/D	L1+L4	#N/D	#N/D	#N/D	24	H4030	Chemagic	1,691475562	23
HSM7J4Q1	USA	Male	12	HMP2	CD	14,9	1	#N/D	L3+L4	#N/D	#N/D	#N/D	1	H4032	Chemagic	2,420526345	47
HSM7J4Q3_P	USA	Male	12	HMP2	CD	14,9	0	#N/D	L3+L4	#N/D	#N/D	#N/D	4	H4032	Chemagic	2,319556366	45
HSM7J4Q7	USA	Male	12	HMP2	CD	14,9	1	#N/D	L3+L4	#N/D	#N/D	#N/D	7	H4032	Chemagic	2,370868214	52
HSM7J4Q9	USA	Male	12	HMP2	CD	14,9	1	#N/D	L3+L4	#N/D	#N/D	#N/D	10	H4032	Chemagic	2,253359979	48
HSM7J4MS	USA	Male	12	HMP2	CD	14,9	0	#N/D	L3+L4	#N/D	#N/D	#N/D	12	H4032	Chemagic	2,341354955	34
HSM7J4MW	USA	Male	12	HMP2	CD	14,9	1	#N/D	L3+L4	#N/D	#N/D	#N/D	16	H4032	Chemagic	2,15906234	35

SampleID	country	gender	Age	study	disease	BMI	hbi	cai	cd localization	cd behaviour	uc extent	dysbiosisindex	Time point	patientID	extraction protocol	shannon	chao
HSM7J4IC	USA	Male	12	HMP2	CD	14,9	0	#N/D	L3+L4	#N/D	#N/D	#N/D	18	H4032	Chemagic	2,352181042	37
HSM7J4OZ	USA	Male	12	HMP2	CD	14,9	0	#N/D	L3+L4	#N/D	#N/D	#N/D	23	H4032	Chemagic	2,515528181	36
HSM7J4P2	USA	Male	12	HMP2	CD	14,9	0	#N/D	L3+L4	#N/D	#N/D	#N/D	27	H4032	Chemagic	2,325197366	41
HSMA33JR	USA	Male	12	HMP2	CD	14,9	1	#N/D	L3+L4	#N/D	#N/D	#N/D	40	H4032	Chemagic	2,545738791	44
HSM7J4J7	USA	Male	15	HMP2	CD	17,3	3	#N/D	L3	#N/D	#N/D	#N/D	2	H4038	Chemagic	1,747685127	34
HSM7J4J9	USA	Male	15	HMP2	CD	17,3	3	#N/D	L3	#N/D	#N/D	#N/D	4	H4038	Chemagic	1,782570667	32
HSM7J4JD	USA	Male	15	HMP2	CD	17,3	0	#N/D	L3	#N/D	#N/D	#N/D	8	H4038	Chemagic	2,049568795	36
HSM7J4JF	USA	Male	15	HMP2	CD	17,3	0	#N/D	L3	#N/D	#N/D	#N/D	10	H4038	Chemagic	2,139777181	37
HSMA33NA	USA	Male	15	HMP2	CD	17,3	0	#N/D	L3	#N/D	#N/D	#N/D	14	H4038	Chemagic	2,124988574	33
HSMA33NC	USA	Male	15	HMP2	CD	17,3	2	#N/D	L3	#N/D	#N/D	#N/D	16	H4038	Chemagic	2,330290338	36
HSMA33NG_P	USA	Male	15	HMP2	CD	17,3	0	#N/D	L3	#N/D	#N/D	#N/D	20	H4038	Chemagic	1,908202798	32
HSMA33KE	USA	Male	15	HMP2	CD	17,3	3	#N/D	L3	#N/D	#N/D	#N/D	24	H4038	Chemagic	2,551147495	37
HSMA33KM	USA	Male	15	HMP2	CD	17,3	0	#N/D	L3	#N/D	#N/D	#N/D	34	H4038	Chemagic	2,293971589	38
HSMA33KO	USA	Male	15	HMP2	CD	17,3	0	#N/D	L3	#N/D	#N/D	#N/D	36	H4038	Chemagic	2,183558058	33
HSMA33PX	USA	Male	15	HMP2	CD	17,3	0	#N/D	L3	#N/D	#N/D	#N/D	38	H4038	Chemagic	2,056813817	36

SampleID	country	gender	Age	study	disease	BMI	hbi	cai	cd localization	cd behaviour	uc extent	dysbiosisindex	Time point	patientID	extraction protocol	shannon	chao
HSMA33PZ	USA	Male	15	HMP2	CD	17,3	4	#N/D	L3	#N/D	#N/D	#N/D	41	H4038	Chemagic	1,227260499	19
HSMA33Q6	USA	Male	15	HMP2	CD	17,3	0	#N/D	L3	#N/D	#N/D	#N/D	48	H4038	Chemagic	1,748627604	30
HSM7J4LP	USA	Female	12	HMP2	CD	16,9	3	#N/D	L3	#N/D	#N/D	#N/D	0	H4039	Chemagic	0,040041746	2
HSM7J4JH	USA	Female	12	HMP2	CD	16,9	0	#N/D	L3	#N/D	#N/D	#N/D	2	H4039	Chemagic	1,802962712	16
HSM7J4JJ	USA	Female	12	HMP2	CD	16,9	0	#N/D	L3	#N/D	#N/D	#N/D	4	H4039	Chemagic	1,93555911	24
HSM7J4JP	USA	Female	12	HMP2	CD	16,9	0	#N/D	L3	#N/D	#N/D	#N/D	10	H4039	Chemagic	1,434402662	25
HSMA33L1	USA	Female	12	HMP2	CD	16,9	0	#N/D	L3	#N/D	#N/D	#N/D	34	H4039	Chemagic	2,538811547	24
HSMA33PL	USA	Female	12	HMP2	CD	16,9	0	#N/D	L3	#N/D	#N/D	#N/D	36	H4039	Chemagic	2,614459749	24
HSMA33IK	USA	Female	14	HMP2	CD	22	1	#N/D	L3	#N/D	#N/D	#N/D	8	H4043	Chemagic	2,39311301	39
HSMA33LP	USA	Female	14	HMP2	CD	22	1	#N/D	L3	#N/D	#N/D	#N/D	16	H4043	Chemagic	2,723429461	51
HSMA33QY	USA	Female	14	HMP2	CD	22	2	#N/D	L3	#N/D	#N/D	#N/D	25	H4043	Chemagic	2,663909115	41
HSMA33R1	USA	Female	14	HMP2	CD	22	6	#N/D	L3	#N/D	#N/D	#N/D	26	H4043	Chemagic	2,365016605	35
HSMA33R5	USA	Female	14	HMP2	CD	22	2	#N/D	L3	#N/D	#N/D	#N/D	30	H4043	Chemagic	2,530663558	44,5
HSMA33R7	USA	Female	14	HMP2	CD	22	2	#N/D	L3	#N/D	#N/D	#N/D	33	H4043	Chemagic	2,138828289	42
HSMA33R9	USA	Female	14	HMP2	CD	22	1	#N/D	L3	#N/D	#N/D	#N/D	35	H4043	Chemagic	2,653655552	46

SampleID	country	gender	Age	study	disease	BMI	hbi	cai	cd localization	cd behaviour	uc extent	dysbiosisindex	Time point	patientID	extraction protocol	shannon	chao
HSMA33S4	USA	Female	14	HMP2	CD	22	1	#N/D	L3	#N/D	#N/D	#N/D	38	H4043	Chemagic	2,39937536	40
MSM5FZ9X_P	USA	Male	30	HMP2	CD	25,4	1	#N/D	L1	#N/D	#N/D	#N/D	4	M2014	Chemagic	2,01762808	30
MSM5FZA2_P	USA	Male	30	HMP2	CD	25,4	3	#N/D	L1	#N/D	#N/D	#N/D	8	M2014	Chemagic	2,05706689	25
MSM5ZOJY_P	USA	Male	30	HMP2	CD	25,4	1	#N/D	L1	#N/D	#N/D	#N/D	10	M2014	Chemagic	1,978151509	23
MSM633FF_P	USA	Male	30	HMP2	CD	25,4	0	#N/D	L1	#N/D	#N/D	#N/D	12	M2014	Chemagic	2,17096811	20
CSM5LLGB_P	USA	Male	30	HMP2	CD	25,4	0	#N/D	L1	#N/D	#N/D	#N/D	14	M2014	Chemagic	1,705256423	35
MSM5LLGH_P	USA	Male	30	HMP2	CD	25,4	0	#N/D	L1	#N/D	#N/D	#N/D	16	M2014	Chemagic	1,883350534	30
MSM5LLGF_P	USA	Male	30	HMP2	CD	25,4	1	#N/D	L1	#N/D	#N/D	#N/D	18	M2014	Chemagic	1,944289781	31
MSM5LLGD_P	USA	Male	30	HMP2	CD	25,4	2	#N/D	L1	#N/D	#N/D	#N/D	20	M2014	Chemagic	1,80626245	27
MSM5LLGJ_P	USA	Male	30	HMP2	CD	25,4	2	#N/D	L1	#N/D	#N/D	#N/D	22	M2014	Chemagic	2,071875509	30
MSM5LLGL	USA	Male	30	HMP2	CD	25,4	2	#N/D	L1	#N/D	#N/D	#N/D	24	M2014	Chemagic	1,936541918	31
MSM6J2IQ	USA	Male	30	HMP2	CD	25,4	0	#N/D	L1	#N/D	#N/D	#N/D	26	M2014	Chemagic	2,136486897	26
MSM6J2IY	USA	Male	30	HMP2	CD	25,4	2	#N/D	L1	#N/D	#N/D	#N/D	34	M2014	Chemagic	1,89540937	39
MSM6J2J1	USA	Male	30	HMP2	CD	25,4	4	#N/D	L1	#N/D	#N/D	#N/D	36	M2014	Chemagic	2,177384844	35
MSM6J2QR	USA	Male	30	HMP2	CD	25,4	3	#N/D	L1	#N/D	#N/D	#N/D	38	M2014	Chemagic	1,927931992	33

SampleID	country	gender	Age	study	disease	BMI	hbi	cai	cd localization	cd behaviour	uc extent	dysbiosisindex	Time point	patientID	extraction protocol	shannon	chao
MSM5LLHR_P	USA	Male	26	HMP2	CD	19,1	2	#N/D	L2	#N/D	#N/D	#N/D	0	M2021	Chemagic	2,422197307	52
MSM5LLIE_P	USA	Male	26	HMP2	CD	19,1	3	#N/D	L2	#N/D	#N/D	#N/D	4	M2021	Chemagic	2,463676367	38
MSM5LLIG_P	USA	Male	26	HMP2	CD	19,1	1	#N/D	L2	#N/D	#N/D	#N/D	6	M2021	Chemagic	2,146200648	36
MSM5LLIK_P	USA	Male	26	HMP2	CD	19,1	2	#N/D	L2	#N/D	#N/D	#N/D	10	M2021	Chemagic	1,714182555	30
MSM5LLIM_P	USA	Male	26	HMP2	CD	19,1	1	#N/D	L2	#N/D	#N/D	#N/D	12	M2021	Chemagic	2,037923851	46
MSM5LLEP	USA	Male	26	HMP2	CD	19,1	1	#N/D	L2	#N/D	#N/D	#N/D	21	M2021	Chemagic	2,337531098	42
MSM5LLER	USA	Male	26	HMP2	CD	19,1	2	#N/D	L2	#N/D	#N/D	#N/D	25	M2021	Chemagic	2,194715762	34
MSM6J2LT	USA	Male	26	HMP2	CD	19,1	2	#N/D	L2	#N/D	#N/D	#N/D	34	M2021	Chemagic	2,277647882	38
MSM5LLHG_P	USA	Female	43	HMP2	CD	19,3	5	#N/D	L2	#N/D	#N/D	#N/D	6	M2025	Chemagic	2,728521891	50,5
MSM5LLHI_P	USA	Female	43	HMP2	CD	19,3	2	#N/D	L2	#N/D	#N/D	#N/D	8	M2025	Chemagic	2,677408836	46
MSM5LLHO_P	USA	Female	43	HMP2	CD	19,3	6	#N/D	L2	#N/D	#N/D	#N/D	14	M2025	Chemagic	2,6245571	44
MSM6J2J3	USA	Female	43	HMP2	CD	19,3	3	#N/D	L2	#N/D	#N/D	#N/D	16	M2025	Chemagic	2,183822205	50
MSM6J2J5	USA	Female	43	HMP2	CD	19,3	7	#N/D	L2	#N/D	#N/D	#N/D	18	M2025	Chemagic	2,803089663	48
MSM6J2JB	USA	Female	43	HMP2	CD	19,3	5	#N/D	L2	#N/D	#N/D	#N/D	23	M2025	Chemagic	2,385163057	41
MSM6J2JD	USA	Female	43	HMP2	CD	19,3	2	#N/D	L2	#N/D	#N/D	#N/D	24	M2025	Chemagic	2,426171048	43

SampleID	country	gender	Age	study	disease	BMI	hbi	cai	cd localization	cd behaviour	uc extent	dysbiosisindex	Time point	patientID	extraction protocol	shannon	chao
MSM6J2PK	USA	Female	43	HMP2	CD	19,3	3	#N/D	L2	#N/D	#N/D	#N/D	35	M2025	Chemagic	2,577688881	32
MSM6J2PM	USA	Female	43	HMP2	CD	19,3	6	#N/D	L2	#N/D	#N/D	#N/D	38	M2025	Chemagic	2,694139244	42
MSM5LLIS_P	USA	Male	41	HMP2	CD	23,7	0	#N/D	L1	#N/D	#N/D	#N/D	0	M2027	Chemagic	0,62098028	32
MSM5LLH2_P	USA	Male	41	HMP2	CD	23,7	5	#N/D	L1	#N/D	#N/D	#N/D	2	M2027	Chemagic	1,379558213	41
MSM5LLH4_P	USA	Male	41	HMP2	CD	23,7	2	#N/D	L1	#N/D	#N/D	#N/D	4	M2027	Chemagic	0,767210372	44
MSM5LLH8_P	USA	Male	41	HMP2	CD	23,7	2	#N/D	L1	#N/D	#N/D	#N/D	8	M2027	Chemagic	0,649293902	32
MSM5LLHA_P	USA	Male	41	HMP2	CD	23,7	2	#N/D	L1	#N/D	#N/D	#N/D	11	M2027	Chemagic	0,915161226	41
MSM5LLHC	USA	Male	41	HMP2	CD	23,7	2	#N/D	L1	#N/D	#N/D	#N/D	15	M2027	Chemagic	1,160536815	46
MSM6J2KC	USA	Male	41	HMP2	CD	23,7	0	#N/D	L1	#N/D	#N/D	#N/D	16	M2027	Chemagic	0,810220331	41
MSM6J2KE	USA	Male	41	HMP2	CD	23,7	2	#N/D	L1	#N/D	#N/D	#N/D	18	M2027	Chemagic	0,971479569	38
MSM6J2KM	USA	Male	41	HMP2	CD	23,7	0	#N/D	L1	#N/D	#N/D	#N/D	24	M2027	Chemagic	1,193949911	41
MSM6J2R2	USA	Male	41	HMP2	CD	23,7	0	#N/D	L1	#N/D	#N/D	#N/D	26	M2027	Chemagic	0,903415409	41
MSM6J2R8	USA	Male	41	HMP2	CD	23,7	0	#N/D	L1	#N/D	#N/D	#N/D	32	M2027	Chemagic	1,153953447	44
MSM6J2RA	USA	Male	41	HMP2	CD	23,7	0	#N/D	L1	#N/D	#N/D	#N/D	36	M2027	Chemagic	1,556634745	50
MSM6J2RC	USA	Male	41	HMP2	CD	23,7	0	#N/D	L1	#N/D	#N/D	#N/D	41	M2027	Chemagic	1,513279331	47

SampleID	country	gender	Age	study	disease	BMI	hbi	cai	cd localization	cd behaviour	uc extent	dysbiosisindex	Time point	patientID	extraction protocol	shannon	chao
MSM5LLFG_P	USA	Female	24	HMP2	CD	20,9	3	#N/D	L1	#N/D	#N/D	#N/D	0	M2028	Chemagic	2,503685971	37
MSM5LLGN_P	USA	Female	24	HMP2	CD	20,9	1	#N/D	L1	#N/D	#N/D	#N/D	2	M2028	Chemagic	2,035479192	30
MSM5LLGR_P	USA	Female	24	HMP2	CD	20,9	2	#N/D	L1	#N/D	#N/D	#N/D	6	M2028	Chemagic	2,123547394	44
MSM6J2IE	USA	Female	24	HMP2	CD	20,9	0	#N/D	L1	#N/D	#N/D	#N/D	14	M2028	Chemagic	2,188202014	32
MSM6J2IG	USA	Female	24	HMP2	CD	20,9	4	#N/D	L1	#N/D	#N/D	#N/D	17	M2028	Chemagic	2,648735614	33
MSM6J2II	USA	Female	24	HMP2	CD	20,9	1	#N/D	L1	#N/D	#N/D	#N/D	19	M2028	Chemagic	2,730210561	32
MSM6J2IK	USA	Female	24	HMP2	CD	20,9	9	#N/D	L1	#N/D	#N/D	#N/D	20	M2028	Chemagic	2,678662416	35
MSM6J2IM	USA	Female	24	HMP2	CD	20,9	0	#N/D	L1	#N/D	#N/D	#N/D	23	M2028	Chemagic	2,869833922	37
MSM6J2IO	USA	Female	24	HMP2	CD	20,9	4	#N/D	L1	#N/D	#N/D	#N/D	24	M2028	Chemagic	2,460359978	38
MSM6J2Q3	USA	Female	24	HMP2	CD	20,9	2	#N/D	L1	#N/D	#N/D	#N/D	26	M2028	Chemagic	2,136805165	35
MSM6J2Q5	USA	Female	24	HMP2	CD	20,9	3	#N/D	L1	#N/D	#N/D	#N/D	29	M2028	Chemagic	2,40485551	38
MSM6J2Q7	USA	Female	24	HMP2	CD	20,9	3	#N/D	L1	#N/D	#N/D	#N/D	30	M2028	Chemagic	2,431638463	29
MSM6J2Q9	USA	Female	24	HMP2	CD	20,9	0	#N/D	L1	#N/D	#N/D	#N/D	33	M2028	Chemagic	2,333431322	28
MSM6J2QB	USA	Female	24	HMP2	CD	20,9	0	#N/D	L1	#N/D	#N/D	#N/D	36	M2028	Chemagic	2,524885949	33
MSM6J2QD	USA	Female	24	HMP2	CD	20,9	0	#N/D	L1	#N/D	#N/D	#N/D	40	M2028	Chemagic	2,286135734	33

SampleID	country	gender	Age	study	disease	BMI	hbi	cai	cd localization	cd behaviour	uc extent	dysbiosisindex	Time point	patientID	extraction protocol	shannon	chao
MSM79H63	USA	Female	24	HMP2	CD	20,9	2	#N/D	L1	#N/D	#N/D	#N/D	41	M2028	Chemagic	2,285418178	32
MSM79H65	USA	Female	24	HMP2	CD	20,9	3	#N/D	L1	#N/D	#N/D	#N/D	41	M2028	Chemagic	2,674138312	35
MSM79H67	USA	Female	24	HMP2	CD	20,9	2	#N/D	L1	#N/D	#N/D	#N/D	44	M2028	Chemagic	2,094670189	33
MSM79H69	USA	Female	24	HMP2	CD	20,9	2	#N/D	L1	#N/D	#N/D	#N/D	47	M2028	Chemagic	2,496888475	37
MSM79H6B	USA	Female	24	HMP2	CD	20,9	1	#N/D	L1	#N/D	#N/D	#N/D	49	M2028	Chemagic	2,401847291	36
MSM5LLF2_P	USA	Female	22	HMP2	CD	31,2	1	#N/D	L3	#N/D	#N/D	#N/D	5	M2034	Chemagic	1,681774767	30
MSM5LLF4	USA	Female	22	HMP2	CD	31,2	2	#N/D	L3	#N/D	#N/D	#N/D	7	M2034	Chemagic	1,517548276	11
MSM5LLF6	USA	Female	22	HMP2	CD	31,2	6	#N/D	L3	#N/D	#N/D	#N/D	10	M2034	Chemagic	2,09359964	38
MSM5LLF8	USA	Female	22	HMP2	CD	31,2	1	#N/D	L3	#N/D	#N/D	#N/D	11	M2034	Chemagic	1,477192486	19
MSM6J2LH	USA	Female	22	HMP2	CD	31,2	4	#N/D	L3	#N/D	#N/D	#N/D	14	M2034	Chemagic	2,271410712	17
MSM6J2LJ	USA	Female	22	HMP2	CD	31,2	1	#N/D	L3	#N/D	#N/D	#N/D	15	M2034	Chemagic	1,855196512	18
MSM6J2LL	USA	Female	22	HMP2	CD	31,2	1	#N/D	L3	#N/D	#N/D	#N/D	17	M2034	Chemagic	2,407055952	20
MSM6J2LN	USA	Female	22	HMP2	CD	31,2	3	#N/D	L3	#N/D	#N/D	#N/D	19	M2034	Chemagic	2,428687299	23
MSM6J2LR	USA	Female	22	HMP2	CD	31,2	3	#N/D	L3	#N/D	#N/D	#N/D	24	M2034	Chemagic	1,931664317	28
MSM6J2MB	USA	Female	22	HMP2	CD	31,2	2	#N/D	L3	#N/D	#N/D	#N/D	25	M2034	Chemagic	2,403166649	26

SampleID	country	gender	Age	study	disease	BMI	hbi	cai	cd localization	cd behaviour	uc extent	dysbiosisindex	Time point	patientID	extraction protocol	shannon	chao
MSM6J2MD	USA	Female	22	HMP2	CD	31,2	6	#N/D	L3	#N/D	#N/D	#N/D	28	M2034	Chemagic	2,161722825	22
MSM6J2MF	USA	Female	22	HMP2	CD	31,2	3	#N/D	L3	#N/D	#N/D	#N/D	30	M2034	Chemagic	2,267109628	26
MSM6J2MH	USA	Female	22	HMP2	CD	31,2	0	#N/D	L3	#N/D	#N/D	#N/D	31	M2034	Chemagic	2,110613855	23
MSM6J2MJ	USA	Female	22	HMP2	CD	31,2	5	#N/D	L3	#N/D	#N/D	#N/D	33	M2034	Chemagic	2,021502338	21
MSM6J2ML	USA	Female	22	HMP2	CD	31,2	5	#N/D	L3	#N/D	#N/D	#N/D	36	M2034	Chemagic	2,125614605	14
MSM79HBN	USA	Female	22	HMP2	CD	31,2	2	#N/D	L3	#N/D	#N/D	#N/D	38	M2034	Chemagic	1,457308547	15
MSM79HBP	USA	Female	22	HMP2	CD	31,2	3	#N/D	L3	#N/D	#N/D	#N/D	39	M2034	Chemagic	2,814265041	41
MSM79HBR	USA	Female	22	HMP2	CD	31,2	2	#N/D	L3	#N/D	#N/D	#N/D	42	M2034	Chemagic	0,260410923	5
MSM79HBT	USA	Female	22	HMP2	CD	31,2	5	#N/D	L3	#N/D	#N/D	#N/D	43	M2034	Chemagic	1,041535012	8
MSM79HBV	USA	Female	22	HMP2	CD	31,2	1	#N/D	L3	#N/D	#N/D	#N/D	45	M2034	Chemagic	1,278148488	9
MSM79HBX_P	USA	Female	19	HMP2	CD	21,3	4	#N/D	L1	#N/D	#N/D	#N/D	0	M2068	Chemagic	2,048193304	38
MSM79HDK	USA	Female	19	HMP2	CD	21,3	3	#N/D	L1	#N/D	#N/D	#N/D	2	M2068	Chemagic	2,506850552	34
MSM79HDM	USA	Female	19	HMP2	CD	21,3	6	#N/D	L1	#N/D	#N/D	#N/D	3	M2068	Chemagic	2,429214124	36
MSM79HDO	USA	Female	19	HMP2	CD	21,3	2	#N/D	L1	#N/D	#N/D	#N/D	5	M2068	Chemagic	2,693936928	35
MSM79HDQ	USA	Female	19	HMP2	CD	21,3	5	#N/D	L1	#N/D	#N/D	#N/D	8	M2068	Chemagic	2,496557101	42

SampleID	country	gender	Age	study	disease	BMI	hbi	cai	cd localization	cd behaviour	uc extent	dysbiosisindex	Time point	patientID	extraction protocol	shannon	chao
MSM79HDQ_TR	USA	Female	19	HMP2	CD	21,3	5	#N/D	L1	#N/D	#N/D	#N/D	8	M2068	Chemagic	2,495815812	40
MSM79HDS	USA	Female	19	HMP2	CD	21,3	3	#N/D	L1	#N/D	#N/D	#N/D	9	M2068	Chemagic	2,630176559	39
MSM79HDU	USA	Female	19	HMP2	CD	21,3	3	#N/D	L1	#N/D	#N/D	#N/D	11	M2068	Chemagic	2,132329137	38
MSM79H98	USA	Female	19	HMP2	CD	21,3	3	#N/D	L1	#N/D	#N/D	#N/D	13	M2068	Chemagic	2,396104565	43
MSM9VZEK	USA	Female	19	HMP2	CD	21,3	4	#N/D	L1	#N/D	#N/D	#N/D	15	M2068	Chemagic	2,196358014	38
MSM9VZEK_TR	USA	Female	19	HMP2	CD	21,3	4	#N/D	L1	#N/D	#N/D	#N/D	15	M2068	Chemagic	2,656831573	31
MSM9VZEM	USA	Female	19	HMP2	CD	21,3	4	#N/D	L1	#N/D	#N/D	#N/D	17	M2068	Chemagic	1,749562963	33
MSM9VZEO	USA	Female	19	HMP2	CD	21,3	1	#N/D	L1	#N/D	#N/D	#N/D	20	M2068	Chemagic	1,762536172	32
MSM9VZES	USA	Female	19	HMP2	CD	21,3	1	#N/D	L1	#N/D	#N/D	#N/D	23	M2068	Chemagic	2,676588852	36
MSM7J16L	USA	Female	19	HMP2	CD	21,3	2	#N/D	L1	#N/D	#N/D	#N/D	28	M2068	Chemagic	2,107964214	45
MSM7J16N	USA	Female	19	HMP2	CD	21,3	3	#N/D	L1	#N/D	#N/D	#N/D	30	M2068	Chemagic	2,780987567	42
MSM7J16P	USA	Female	19	HMP2	CD	21,3	5	#N/D	L1	#N/D	#N/D	#N/D	32	M2068	Chemagic	2,489705805	44
MSM7J16R	USA	Female	19	HMP2	CD	21,3	5	#N/D	L1	#N/D	#N/D	#N/D	34	M2068	Chemagic	2,329858079	46
MSM9VZJB	USA	Female	19	HMP2	CD	21,3	4	#N/D	L1	#N/D	#N/D	#N/D	36	M2068	Chemagic	2,250858527	41
MSMA26AL	USA	Female	19	HMP2	CD	21,3	1	#N/D	L1	#N/D	#N/D	#N/D	39	M2068	Chemagic	2,861833481	46

SampleID	country	gender	Age	study	disease	BMI	hbi	cai	cd localization	cd behaviour	uc extent	dysbiosisindex	Time point	patientID	extraction protocol	shannon	chao
MSMA26AN	USA	Female	19	HMP2	CD	21,3	3	#N/D	L1	#N/D	#N/D	#N/D	42	M2068	Chemagic	2,408801773	41
MSMA26AP	USA	Female	19	HMP2	CD	21,3	3	#N/D	L1	#N/D	#N/D	#N/D	43	M2068	Chemagic	2,229149273	47
MSMA26AR	USA	Female	19	HMP2	CD	21,3	2	#N/D	L1	#N/D	#N/D	#N/D	46	M2068	Chemagic	2,631933788	44
MSMA26AT	USA	Female	19	HMP2	CD	21,3	3	#N/D	L1	#N/D	#N/D	#N/D	47	M2068	Chemagic	2,661439064	42
PSM6XBQM_P	USA	Male	11	HMP2	CD	14,3	6	#N/D	L3+L4	#N/D	#N/D	#N/D	0	P6005	Chemagic	2,148704671	38
PSM6XBQS	USA	Male	11	HMP2	CD	14,3	5	#N/D	L3+L4	#N/D	#N/D	#N/D	2	P6005	Chemagic	1,986792944	45
PSM6XBQU	USA	Male	11	HMP2	CD	14,3	1	#N/D	L3+L4	#N/D	#N/D	#N/D	4	P6005	Chemagic	2,48368289	48,5
PSM6XBQY	USA	Male	11	HMP2	CD	14,3	2	#N/D	L3+L4	#N/D	#N/D	#N/D	8	P6005	Chemagic	2,479587274	36
PSM6XBQY_TR	USA	Male	11	HMP2	CD	14,3	2	#N/D	L3+L4	#N/D	#N/D	#N/D	8	P6005	Chemagic	2,435531764	45
PSM6XBR1	USA	Male	11	HMP2	CD	14,3	1	#N/D	L3+L4	#N/D	#N/D	#N/D	10	P6005	Chemagic	2,553863522	42
PSM6XBSS	USA	Male	11	HMP2	CD	14,3	1	#N/D	L3+L4	#N/D	#N/D	#N/D	16	P6005	Chemagic	2,20899069	44
PSM6XBSU	USA	Male	11	HMP2	CD	14,3	0	#N/D	L3+L4	#N/D	#N/D	#N/D	18	P6005	Chemagic	2,315093421	41,33333333
PSM6XBT1	USA	Male	11	HMP2	CD	14,3	1	#N/D	L3+L4	#N/D	#N/D	#N/D	24	P6005	Chemagic	2,508500211	42
PSM7J1C8	USA	Male	11	HMP2	CD	14,3	0	#N/D	L3+L4	#N/D	#N/D	#N/D	26	P6005	Chemagic	2,235140225	43
PSM7J1CC	USA	Male	11	HMP2	CD	14,3	0	#N/D	L3+L4	#N/D	#N/D	#N/D	30	P6005	Chemagic	2,566981959	49

SampleID	country	gender	Age	study	disease	BMI	hbi	cai	cd localization	cd behaviour	uc extent	dysbiosisindex	Time point	patientID	extraction protocol	shannon	chao
PSM7J1CG	USA	Male	11	HMP2	CD	14,3	1	#N/D	L3+L4	#N/D	#N/D	#N/D	34	P6005	Chemagic	2,01807402	27,5
PSM7J1CI	USA	Male	11	HMP2	CD	14,3	0	#N/D	L3+L4	#N/D	#N/D	#N/D	37	P6005	Chemagic	2,300695462	33
PSM7J136	USA	Male	11	HMP2	CD	14,3	0	#N/D	L3+L4	#N/D	#N/D	#N/D	41	P6005	Chemagic	2,214427986	39
PSM6XBRK_P	USA	Male	16	HMP2	CD	22,1	3	#N/D	L2	#N/D	#N/D	#N/D	0	P6009	Chemagic	2,378084002	53
PSM6XBRK	USA	Male	16	HMP2	CD	22,1	3	#N/D	L2	#N/D	#N/D	#N/D	0	P6009	Chemagic	2,361515956	47
PSM6XBRK_TR	USA	Male	16	HMP2	CD	22,1	3	#N/D	L2	#N/D	#N/D	#N/D	0	P6009	Chemagic	2,489236027	47
PSM6XBS2_P	USA	Male	16	HMP2	CD	22,1	1	#N/D	L2	#N/D	#N/D	#N/D	1	P6009	Chemagic	2,136631097	48
PSM6XBS4_P	USA	Male	16	HMP2	CD	22,1	2	#N/D	L2	#N/D	#N/D	#N/D	3	P6009	Chemagic	2,344145583	45
PSM6XBS4	USA	Male	16	HMP2	CD	22,1	2	#N/D	L2	#N/D	#N/D	#N/D	3	P6009	Chemagic	2,418908857	42
PSM6XBS8	USA	Male	16	HMP2	CD	22,1	6	#N/D	L2	#N/D	#N/D	#N/D	7	P6009	Chemagic	2,000232078	43
PSM6XBSA	USA	Male	16	HMP2	CD	22,1	6	#N/D	L2	#N/D	#N/D	#N/D	10	P6009	Chemagic	2,433533354	44
PSM6XBSC_P	USA	Male	16	HMP2	CD	22,1	4	#N/D	L2	#N/D	#N/D	#N/D	11	P6009	Chemagic	2,446504022	42
PSM6XBTF_P	USA	Male	16	HMP2	CD	22,1	3	#N/D	L2	#N/D	#N/D	#N/D	13	P6009	Chemagic	2,197799583	54
PSM6XBTH_P	USA	Male	16	HMP2	CD	22,1	1	#N/D	L2	#N/D	#N/D	#N/D	15	P6009	Chemagic	2,473360022	50
PSM6XBTL	USA	Male	16	HMP2	CD	22,1	5	#N/D	L2	#N/D	#N/D	#N/D	19	P6009	Chemagic	2,245619999	50

SampleID	country	gender	Age	study	disease	BMI	hbi	cai	cd localization	cd behaviour	uc extent	dysbiosisindex	Time point	patientID	extraction protocol	shannon	chao
PSM6XBTN_P	USA	Male	16	HMP2	CD	22,1	2	#N/D	L2	#N/D	#N/D	#N/D	21	P6009	Chemagic	2,427429222	51
PSM6XBTP	USA	Male	16	HMP2	CD	22,1	4	#N/D	L2	#N/D	#N/D	#N/D	23	P6009	Chemagic	2,473148673	45
PSM7J1CK_P	USA	Male	16	HMP2	CD	22,1	5	#N/D	L2	#N/D	#N/D	#N/D	25	P6009	Chemagic	2,107241197	42
PSM7J1CS_P	USA	Male	16	HMP2	CD	22,1	5	#N/D	L2	#N/D	#N/D	#N/D	33	P6009	Chemagic	2,287408143	22
PSM7J1CU	USA	Male	16	HMP2	CD	22,1	4	#N/D	L2	#N/D	#N/D	#N/D	36	P6009	Chemagic	2,328110036	25
PSM7J13U_P	USA	Male	16	HMP2	CD	22,1	4	#N/D	L2	#N/D	#N/D	#N/D	39	P6009	Chemagic	2,019525843	19
PSM7J13Y_P	USA	Male	16	HMP2	CD	22,1	5	#N/D	L2	#N/D	#N/D	#N/D	43	P6009	Chemagic	2,531357322	28
PSM7J13Y	USA	Male	16	HMP2	CD	22,1	5	#N/D	L2	#N/D	#N/D	#N/D	43	P6009	Chemagic	2,54805501	28
PSM7J141	USA	Male	16	HMP2	CD	22,1	4	#N/D	L2	#N/D	#N/D	#N/D	44	P6009	Chemagic	2,168105329	28
PSM7J143_P	USA	Male	16	HMP2	CD	22,1	2	#N/D	L2	#N/D	#N/D	#N/D	46	P6009	Chemagic	2,164609925	29
PSM6XBRM_P	USA	Male	10	HMP2	CD	19	4	#N/D	L3+L4	#N/D	#N/D	#N/D	0	P6010	Chemagic	2,293756295	39
PSM6XBSI	USA	Male	10	HMP2	CD	19	7	#N/D	L3+L4	#N/D	#N/D	#N/D	2	P6010	Chemagic	2,279743548	42
PSM6XBSO	USA	Male	10	HMP2	CD	19	6	#N/D	L3+L4	#N/D	#N/D	#N/D	4	P6010	Chemagic	2,320543071	32
PSM6XBSM	USA	Male	10	HMP2	CD	19	3	#N/D	L3+L4	#N/D	#N/D	#N/D	6	P6010	Chemagic	2,609685894	43
PSM6XBSK	USA	Male	10	HMP2	CD	19	4	#N/D	L3+L4	#N/D	#N/D	#N/D	9	P6010	Chemagic	2,242644958	47

SampleID	country	gender	Age	study	disease	BMI	hbi	cai	cd localization	cd behaviour	uc extent	dysbiosisindex	Time point	patientID	extraction protocol	shannon	chao
PSM6XBV2	USA	Male	10	HMP2	CD	19	3	#N/D	L3+L4	#N/D	#N/D	#N/D	10	P6010	Chemagic	2,410555678	42
PSM6XBV4	USA	Male	10	HMP2	CD	19	3	#N/D	L3+L4	#N/D	#N/D	#N/D	12	P6010	Chemagic	0,839240511	22
PSM6XBUG	USA	Male	10	HMP2	CD	19	4	#N/D	L3+L4	#N/D	#N/D	#N/D	15	P6010	Chemagic	2,15409333	29
PSM6XBUI	USA	Male	10	HMP2	CD	19	8	#N/D	L3+L4	#N/D	#N/D	#N/D	17	P6010	Chemagic	2,287100177	26
PSM6XBUM	USA	Male	10	HMP2	CD	19	1	#N/D	L3+L4	#N/D	#N/D	#N/D	20	P6010	Chemagic	2,594035829	46
PSM6XBUK	USA	Male	10	HMP2	CD	19	3	#N/D	L3+L4	#N/D	#N/D	#N/D	21	P6010	Chemagic	2,534699628	40
PSM6XBUQ	USA	Male	10	HMP2	CD	19	6	#N/D	L3+L4	#N/D	#N/D	#N/D	23	P6010	Chemagic	2,311123928	41
PSM6XBUO	USA	Male	10	HMP2	CD	19	2	#N/D	L3+L4	#N/D	#N/D	#N/D	25	P6010	Chemagic	2,31845093	43
PSM7J18E	USA	Male	10	HMP2	CD	19	2	#N/D	L3+L4	#N/D	#N/D	#N/D	27	P6010	Chemagic	2,173450681	42
PSM7J18G	USA	Male	10	HMP2	CD	19	3	#N/D	L3+L4	#N/D	#N/D	#N/D	29	P6010	Chemagic	2,083838961	39
PSM7J18I	USA	Male	10	HMP2	CD	19	3	#N/D	L3+L4	#N/D	#N/D	#N/D	31	P6010	Chemagic	2,314262885	40
PSM7J18K	USA	Male	10	HMP2	CD	19	3	#N/D	L3+L4	#N/D	#N/D	#N/D	34	P6010	Chemagic	1,408635906	31
PSM7J18M	USA	Male	10	HMP2	CD	19	3	#N/D	L3+L4	#N/D	#N/D	#N/D	35	P6010	Chemagic	2,240155042	39
PSM7J14L	USA	Male	10	HMP2	CD	19	6	#N/D	L3+L4	#N/D	#N/D	#N/D	40	P6010	Chemagic	0,009115999	2
PSM7J14N	USA	Male	10	HMP2	CD	19	8	#N/D	L3+L4	#N/D	#N/D	#N/D	41	P6010	Chemagic	1,825566845	36

SampleID	country	gender	Age	study	disease	BMI	hbi	cai	cd localization	cd behaviour	uc extent	dysbiosisindex	Time point	patientID	extraction protocol	shannon	chao
PSM7J14P	USA	Male	10	HMP2	CD	19	6	#N/D	L3+L4	#N/D	#N/D	#N/D	44	P6010	Chemagic	2,259236433	39
PSM7J14R	USA	Male	10	HMP2	CD	19	5	#N/D	L3+L4	#N/D	#N/D	#N/D	45	P6010	Chemagic	2,493387768	46
PSM7J14T	USA	Male	10	HMP2	CD	19	2	#N/D	L3+L4	#N/D	#N/D	#N/D	47	P6010	Chemagic	2,805194531	50
PSM6XBVY_P	USA	Male	16	HMP2	CD	14,7	2	#N/D	L3+L4	#N/D	#N/D	#N/D	0	P6016	Chemagic	2,534495445	40
PSM7J199	USA	Male	16	HMP2	CD	14,7	2	#N/D	L3+L4	#N/D	#N/D	#N/D	2	P6016	Chemagic	1,914789478	33
PSM7J19B	USA	Male	16	HMP2	CD	14,7	1	#N/D	L3+L4	#N/D	#N/D	#N/D	4	P6016	Chemagic	2,227384277	39
PSM7J19F	USA	Male	16	HMP2	CD	14,7	1	#N/D	L3+L4	#N/D	#N/D	#N/D	8	P6016	Chemagic	2,11062285	31
PSM7J19J	USA	Male	16	HMP2	CD	14,7	0	#N/D	L3+L4	#N/D	#N/D	#N/D	12	P6016	Chemagic	2,356912278	36
PSM7J17L	USA	Male	16	HMP2	CD	14,7	1	#N/D	L3+L4	#N/D	#N/D	#N/D	16	P6016	Chemagic	2,530020577	39
PSM7J17T	USA	Male	16	HMP2	CD	14,7	0	#N/D	L3+L4	#N/D	#N/D	#N/D	24	P6016	Chemagic	2,390768536	32
PSM7J15A	USA	Male	16	HMP2	CD	14,7	1	#N/D	L3+L4	#N/D	#N/D	#N/D	28	P6016	Chemagic	2,306960646	33
PSM7J15G	USA	Male	16	HMP2	CD	14,7	0	#N/D	L3+L4	#N/D	#N/D	#N/D	34	P6016	Chemagic	1,93178877	33
PSM7J15I	USA	Male	16	HMP2	CD	14,7	0	#N/D	L3+L4	#N/D	#N/D	#N/D	36	P6016	Chemagic	2,392485962	37
PSMA265N	USA	Male	16	HMP2	CD	14,7	0	#N/D	L3+L4	#N/D	#N/D	#N/D	38	P6016	Chemagic	2,082562668	32
PSMA265T	USA	Male	16	HMP2	CD	14,7	0	#N/D	L3+L4	#N/D	#N/D	#N/D	45	P6016	Chemagic	2,210483686	30

SampleID	country	gender	Age	study	disease	BMI	hbi	cai	cd localization	cd behaviour	uc extent	dysbiosisindex	Time point	patientID	extraction protocol	shannon	chao
PSM7J1BJ	USA	Male	16	HMP2	CD	28,4	1	#N/D	L3+L4	#N/D	#N/D	#N/D	2	P6024	Chemagic	1,467435339	8
PSM7J1BL	USA	Male	16	HMP2	CD	28,4	1	#N/D	L3+L4	#N/D	#N/D	#N/D	4	P6024	Chemagic	0,621472827	4
PSM7J1BN_P	USA	Male	16	HMP2	CD	28,4	2	#N/D	L3+L4	#N/D	#N/D	#N/D	6	P6024	Chemagic	0,457234004	5
PSM7J1BP	USA	Male	16	HMP2	CD	28,4	2	#N/D	L3+L4	#N/D	#N/D	#N/D	8	P6024	Chemagic	0,675590689	6
PSM7J1BR	USA	Male	16	HMP2	CD	28,4	1	#N/D	L3+L4	#N/D	#N/D	#N/D	10	P6024	Chemagic	0,533100216	8
PSM7J12J	USA	Male	16	HMP2	CD	28,4	2	#N/D	L3+L4	#N/D	#N/D	#N/D	15	P6024	Chemagic	0,561648338	14
PSM7J12R	USA	Male	16	HMP2	CD	28,4	1	#N/D	L3+L4	#N/D	#N/D	#N/D	23	P6024	Chemagic	1,087258814	13
PSMA263M	USA	Male	16	HMP2	CD	28,4	0	#N/D	L3+L4	#N/D	#N/D	#N/D	25	P6024	Chemagic	1,111825326	11
PSMA263S	USA	Male	16	HMP2	CD	28,4	1	#N/D	L3+L4	#N/D	#N/D	#N/D	32	P6024	Chemagic	1,363370749	13
PSMA263U	USA	Male	16	HMP2	CD	28,4	1	#N/D	L3+L4	#N/D	#N/D	#N/D	35	P6024	Chemagic	1,461751897	12
PSMA263W	USA	Male	16	HMP2	CD	28,4	1	#N/D	L3+L4	#N/D	#N/D	#N/D	37	P6024	Chemagic	1,4447946	15
PSM7J1B3_P	USA	Male	9	HMP2	CD	15,9	6	#N/D	L1+L4	#N/D	#N/D	#N/D	0	P6028	Chemagic	2,386674585	62
PSM7J179	USA	Male	9	HMP2	CD	15,9	8	#N/D	L1+L4	#N/D	#N/D	#N/D	4	P6028	Chemagic	1,523025872	32
PSM7J17B	USA	Male	9	HMP2	CD	15,9	5	#N/D	L1+L4	#N/D	#N/D	#N/D	6	P6028	Chemagic	0	1
PSM7J17D	USA	Male	9	HMP2	CD	15,9	3	#N/D	L1+L4	#N/D	#N/D	#N/D	8	P6028	Chemagic	0,755282054	3

SampleID	country	gender	Age	study	disease	BMI	hbi	cai	cd localization	cd behaviour	uc extent	dysbiosisindex	Time point	patientID	extraction protocol	shannon	chao
PSM7J17F	USA	Male	9	HMP2	CD	15,9	3	#N/D	L1+L4	#N/D	#N/D	#N/D	10	P6028	Chemagic	0	1
PSM7J15W	USA	Male	9	HMP2	CD	15,9	0	#N/D	L1+L4	#N/D	#N/D	#N/D	19	P6028	Chemagic	2,667413244	48
PSM7J161	USA	Male	9	HMP2	CD	15,9	2	#N/D	L1+L4	#N/D	#N/D	#N/D	23	P6028	Chemagic	0,003029557	3
PSM7J163	USA	Male	9	HMP2	CD	15,9	2	#N/D	L1+L4	#N/D	#N/D	#N/D	25	P6028	Chemagic	0	1
PSM7J13I	USA	Male	15	HMP2	CD	16,4	3	#N/D	L3	#N/D	#N/D	#N/D	3	P6033	Chemagic	2,117764027	30
PSM7J13K	USA	Male	15	HMP2	CD	16,4	3	#N/D	L3	#N/D	#N/D	#N/D	4	P6033	Chemagic	2,063694025	25
PSM7J13Q	USA	Male	15	HMP2	CD	16,4	1	#N/D	L3	#N/D	#N/D	#N/D	8	P6033	Chemagic	1,829720891	28
PSM7J13M	USA	Male	15	HMP2	CD	16,4	2	#N/D	L3	#N/D	#N/D	#N/D	10	P6033	Chemagic	2,000381407	32
PSMA2651_P	USA	Male	15	HMP2	CD	16,4	2	#N/D	L3	#N/D	#N/D	#N/D	15	P6033	Chemagic	2,066656562	38
PSMA2653	USA	Male	15	HMP2	CD	16,4	2	#N/D	L3	#N/D	#N/D	#N/D	17	P6033	Chemagic	2,283036492	34
PSMA2659	USA	Male	15	HMP2	CD	16,4	1	#N/D	L3	#N/D	#N/D	#N/D	24	P6033	Chemagic	2,178791302	33
PSMA265B	USA	Male	15	HMP2	CD	16,4	0	#N/D	L3	#N/D	#N/D	#N/D	26	P6033	Chemagic	1,95590997	29
PSMA267D	USA	Male	15	HMP2	CD	16,4	3	#N/D	L3	#N/D	#N/D	#N/D	34	P6033	Chemagic	2,022233687	33
PSMA267F	USA	Male	15	HMP2	CD	16,4	3	#N/D	L3	#N/D	#N/D	#N/D	36	P6033	Chemagic	2,095474038	36
PSMA267H	USA	Male	15	HMP2	CD	16,4	3	#N/D	L3	#N/D	#N/D	#N/D	38	P6033	Chemagic	2,112904679	36

SampleID	country	gender	Age	study	disease	BMI	hbi	cai	cd localization	cd behaviour	uc extent	dysbiosisindex	Time point	patientID	extraction protocol	shannon	chao
V1.UC1.0	Spain	Female	31	Meta Hit	UC	17,15	#N/D	0	#N/D	#N/D	E2	#N/D	0	1UC	Godon	2,797715748	56
V1.UC2.0	Spain	Male	62	Meta Hit	UC	27,76	#N/D	2	#N/D	#N/D	E1	#N/D	0	2UC	Godon	3,300911234	58
V1.UC3.0	Spain	Male	44	Meta Hit	UC	20,57	#N/D	2	#N/D	#N/D	E3	#N/D	0	3UC	Godon	2,420217102	45
V1.UC4.0	Spain	Female	47	Meta Hit	UC	26,37	#N/D	1	#N/D	#N/D	E3	#N/D	0	4UC	Godon	2,731161115	53
V1.UC5.0	Spain	Female	30	Meta Hit	UC	20,32	#N/D	1	#N/D	#N/D	E3	#N/D	0	5UC	Godon	2,870276196	62
V1.UC10.0	Spain	Male	45	Meta Hit	UC	27,31	#N/D	1	#N/D	#N/D	E3	#N/D	0	10UC	Godon	2,814670244	52
V1.UC11.0	Spain	Female	48	Meta Hit	UC	22,32	#N/D	0	#N/D	#N/D	E3	#N/D	0	11UC	Godon	2,345326827	28
V1.UC12.0	Spain	Female	41	Meta Hit	UC	19,9	#N/D	0	#N/D	#N/D	E1	#N/D	0	12UC	Godon	3,24260934	57
V1.UC13.0	Spain	Female	51	Meta Hit	UC	28,51	#N/D	0	#N/D	#N/D	E1	#N/D	0	13UC	Godon	2,598355375	26
V1.UC14.0	Spain	Female	53	Meta Hit	UC	20,25	#N/D	0	#N/D	#N/D	E2	#N/D	0	14UC	Godon	2,640495435	35
V1.UC15.0	Spain	Female	25	Meta Hit	UC	22,77	#N/D	0	#N/D	#N/D	E2	#N/D	0	15UC	Godon	2,526215547	31
V1.UC17.0	Spain	Female	41	Meta Hit	UC	24,46	#N/D	0	#N/D	#N/D	E3	#N/D	0	17UC	Godon	2,685720029	41
V1.UC21.0	Spain	Male	62	Meta Hit	UC	25,21	#N/D	0	#N/D	#N/D	E3	#N/D	0	21UC	Godon	2,544795563	43
V1.UC23.0	Spain	Male	43	Meta Hit	UC	28,73	#N/D	0	#N/D	#N/D	E2	#N/D	0	23UC	Godon	3,193997841	57
V1.UC25.0	Spain	Female	52	Meta Hit	UC	23,44	#N/D	0	#N/D	#N/D	E2	#N/D	0	25UC	Godon	2,663219613	52,33333333

SampleID	country	gender	Age	study	disease	BMI	hbi	cai	cd localization	cd behaviour	uc extent	dysbiosisindex	Time point	patientID	extraction protocol	shannon	chao
V1.UC26.0	Spain	Female	36	Meta Hit	UC	28,58	#N/D	1	#N/D	#N/D	E2	#N/D	0	26UC	Godon	3,266292212	53
V1.UC31.0	Spain	Female	26	Meta Hit	UC	20,44	#N/D	0	#N/D	#N/D	E3	#N/D	0	31UC	Godon	3,089452278	47
V1.UC35.0	Spain	Female	33	Meta Hit	UC	19,43	#N/D	0	#N/D	#N/D	E3	#N/D	0	35UC	Godon	3,019426318	54
V1.UC38.0	Spain	Male	24	Meta Hit	UC	18,07	#N/D	0	#N/D	#N/D	E3	#N/D	0	38UC	Godon	2,859601968	57
V1.UC39.0	Spain	Male	26	Meta Hit	UC	22,4	#N/D	1	#N/D	#N/D	E1	#N/D	0	39UC	Godon	3,120342287	61
V1.UC40.0	Spain	Female	32	Meta Hit	UC	25,89	#N/D	0	#N/D	#N/D	E3	#N/D	0	40UC	Godon	2,888757717	51
V1.UC45.0	Spain	Female	50	Meta Hit	UC	23,05	#N/D	1	#N/D	#N/D	E2	#N/D	0	45UC	Godon	3,042320576	49
V1.UC47.0	Spain	Male	46	Meta Hit	UC	25,71	#N/D	0	#N/D	#N/D	E1	#N/D	0	47UC	Godon	3,434059666	72
V1.UC49.0	Spain	Female	25	Meta Hit	UC	19,83	#N/D	2	#N/D	#N/D	E2	#N/D	0	49UC	Godon	3,606389996	74
V1.UC51.0	Spain	Female	44	Meta Hit	UC	23,31	#N/D	0	#N/D	#N/D	E1	#N/D	0	51UC	Godon	3,082296476	62
V1.UC52.0	Spain	Female	43	Meta Hit	UC	29,34	#N/D	0	#N/D	#N/D	E1	#N/D	0	52UC	Godon	3,298653131	72
V1.UC53.0	Spain	Female	24	Meta Hit	UC	20,24	#N/D	0	#N/D	#N/D	E2	#N/D	0	53UC	Godon	2,206052662	44
V1.UC54.0	Spain	Male	32	Meta Hit	UC	35,62	#N/D	0	#N/D	#N/D	E3	#N/D	0	54UC	Godon	3,312240418	60
V1.UC55.0	Spain	Female	46	Meta Hit	UC	18,82	#N/D	0	#N/D	#N/D	E1	#N/D	0	55UC	Godon	2,842791515	63
V1.UC56.0	Spain	Female	35	Meta Hit	UC	18,83	#N/D	0	#N/D	#N/D	E3	#N/D	0	56UC	Godon	1,164323906	33

SampleID	country	gender	Age	study	disease	BMI	hbi	cai	cd localization	cd behaviour	uc extent	dysbiosisindex	Time point	patientID	extraction protocol	shannon	chao
V1.UC58.0	Spain	Female	41	Meta Hit	UC	28,35	#N/D	0	#N/D	#N/D	E1	#N/D	0	58UC	Godon	2,952670798	68
V1.UC59.0	Spain	Female	46	Meta Hit	UC	23,44	#N/D	1	#N/D	#N/D	E2	#N/D	0	59UC	Godon	2,724504819	49
V1.UC50.0	Spain	Female	45	Meta Hit	UC	20,71	#N/D	0	#N/D	#N/D	E2	#N/D	0	50UC	Godon	2,604461558	50
V1.UC2.4	Spain	Male	62	Meta Hit	UC	30,02	#N/D	8	#N/D	#N/D	#N/D	#N/D	4	2UC	Godon	2,972864485	53
V1.UC3.2	Spain	Male	44	Meta Hit	UC	19,27	#N/D	13	#N/D	#N/D	#N/D	#N/D	2	3UC	Godon	1,853661198	49
V1.UC4.5	Spain	Female	47	Meta Hit	UC	26,37	#N/D	0	#N/D	#N/D	#N/D	#N/D	5	4UC	Godon	3,28891998	60
V1.UC5.3	Spain	Female	30	Meta Hit	UC	20,7	#N/D	0	#N/D	#N/D	#N/D	#N/D	3	5UC	Godon	3,264083227	66
V1.UC10.2	Spain	Male	45	Meta Hit	UC	27,31	#N/D	7	#N/D	#N/D	#N/D	#N/D	2	10UC	Godon	3,049812638	62
V1.UC11.5	Spain	Female	48	Meta Hit	UC	23,03	#N/D	2	#N/D	#N/D	#N/D	#N/D	5	11UC	Godon	2,789321458	33
V1.UC12.4	Spain	Female	41	Meta Hit	UC	19,79	#N/D	6	#N/D	#N/D	#N/D	#N/D	4	12UC	Godon	2,803658753	60,5
V1.UC13.3	Spain	Female	51	Meta Hit	UC	28,95	#N/D	7	#N/D	#N/D	#N/D	#N/D	3	13UC	Godon	2,951054245	45
V1.UC14.1	Spain	Female	53	Meta Hit	UC	20,06	#N/D	7	#N/D	#N/D	#N/D	#N/D	1	14UC	Godon	1,376664792	35
V1.UC15.3	Spain	Female	25	Meta Hit	UC	23,19	#N/D	6	#N/D	#N/D	#N/D	#N/D	3	15UC	Godon	3,057833231	45
V1.UC17.2	Spain	Female	41	Meta Hit	UC	24,84	#N/D	9	#N/D	#N/D	#N/D	#N/D	2	17UC	Godon	2,680886832	52
V1.UC21.4	Spain	Male	62	Meta Hit	UC	26,26	#N/D	1	#N/D	#N/D	#N/D	#N/D	4	21UC	Godon	2,901271251	53

SampleID	country	gender	Age	study	disease	BMI	hbi	cai	cd localization	cd behaviour	uc extent	dysbiosisindex	Time point	patientID	extraction protocol	shannon	chao
V1.UC23.1	Spain	Male	43	Meta Hit	UC	26,49	#N/D	8	#N/D	#N/D	#N/D	#N/D	1	23UC	Godon	3,165887788	54
V1.UC25.1	Spain	Female	52	Meta Hit	UC	23,05	#N/D	7	#N/D	#N/D	#N/D	#N/D	1	25UC	Godon	2,735956674	50
V1.UC26.4	Spain	Female	36	Meta Hit	UC	26,29	#N/D	8	#N/D	#N/D	#N/D	#N/D	4	26UC	Godon	3,117060327	62
V1.UC31.4	Spain	Female	26	Meta Hit	UC	22,22	#N/D	2	#N/D	#N/D	#N/D	#N/D	4	31UC	Godon	2,932561692	47
V1.UC35.4	Spain	Female	33	Meta Hit	UC	20,43	#N/D	0	#N/D	#N/D	#N/D	#N/D	4	35UC	Godon	2,885361514	50
V1.UC38.4	Spain	Male	24	Meta Hit	UC	19,26	#N/D	0	#N/D	#N/D	#N/D	#N/D	4	38UC	Godon	2,806963905	45
V1.UC39.4	Spain	Male	26	Meta Hit	UC	21,5	#N/D	1	#N/D	#N/D	#N/D	#N/D	4	39UC	Godon	3,079564166	61,33333333
V1.UC40.1	Spain	Female	32	Meta Hit	UC	27,12	#N/D	7	#N/D	#N/D	#N/D	#N/D	1	40UC	Godon	3,018103673	53
V1.UC47.4	Spain	Male	46	Meta Hit	UC	26,45	#N/D	0	#N/D	#N/D	#N/D	#N/D	4	47UC	Godon	3,236368203	68,5
V1.UC49.1	Spain	Female	25	Meta Hit	UC	20,2	#N/D	4	#N/D	#N/D	#N/D	#N/D	1	49UC	Godon	2,887169992	47
V1.UC50.1	Spain	Female	45	Meta Hit	UC	20,71	#N/D	5	#N/D	#N/D	#N/D	#N/D	1	50UC	Godon	2,925314363	48,5
V1.UC51.4	Spain	Female	44	Meta Hit	UC	23,31	#N/D	0	#N/D	#N/D	#N/D	#N/D	4	51UC	Godon	3,327138938	74
V1.UC52.1	Spain	Female	43	Meta Hit	UC	29,34	#N/D	5	#N/D	#N/D	#N/D	#N/D	1	52UC	Godon	3,268116222	65
V1.UC53.4	Spain	Female	24	Meta Hit	UC	19,59	#N/D	0	#N/D	#N/D	#N/D	#N/D	4	53UC	Godon	1,287089572	44,5
V1.UC54.4	Spain	Male	32	Meta Hit	UC	35,93	#N/D	0	#N/D	#N/D	#N/D	#N/D	4	54UC	Godon	2,744145948	50

SampleID	country	gender	Age	study	disease	BMI	hbi	cai	cd localization	cd behaviour	uc extent	dysbiosisindex	Time point	patientID	extraction protocol	shannon	chao
V1.UC55.4	Spain	Female	46	Meta Hit	UC	18,82	#N/D	0	#N/D	#N/D	#N/D	#N/D	4	55UC	Godon	3,283319692	60
V1.UC56.1	Spain	Female	35	Meta Hit	UC	18,83	#N/D	6	#N/D	#N/D	#N/D	#N/D	1	56UC	Godon	1,357932495	19
V1.UC58.4	Spain	Female	41	Meta Hit	UC	28,76	#N/D	1	#N/D	#N/D	#N/D	#N/D	4	58UC	Godon	3,297809907	66
V1.UC59.4	Spain	Female	46	Meta Hit	UC	23,83	#N/D	8	#N/D	#N/D	#N/D	#N/D	4	59UC	Godon	2,012746834	12
V1.UC1.3	Spain	Female	31	Meta Hit	UC	18,48	#N/D	0	#N/D	#N/D	#N/D	#N/D	3	1UC	Godon	2,641906179	64
CSM5MCUQ_P	USA	Female	76	HMP2	UC	30,9	#N/D	1	#N/D	#N/D	#N/D	#N/D	1	C3005	Chemagic	2,453161381	51
CSM5MCUS_P	USA	Female	76	HMP2	UC	30,9	#N/D	0	#N/D	#N/D	#N/D	#N/D	4	C3005	Chemagic	2,591981963	46
CSM5MCUW_P	USA	Female	76	HMP2	UC	30,9	#N/D	1	#N/D	#N/D	#N/D	#N/D	7	C3005	Chemagic	2,315347303	47
CSM5MCUY_P	USA	Female	76	HMP2	UC	30,9	#N/D	0	#N/D	#N/D	#N/D	#N/D	9	C3005	Chemagic	2,270536019	51
CSM5MCY4	USA	Female	76	HMP2	UC	30,9	#N/D	1	#N/D	#N/D	#N/D	#N/D	12	C3005	Chemagic	2,272583136	45
CSM5MCY8	USA	Female	76	HMP2	UC	30,9	#N/D	0	#N/D	#N/D	#N/D	#N/D	15	C3005	Chemagic	2,277714479	44
CSM67UE3	USA	Female	76	HMP2	UC	30,9	#N/D	0	#N/D	#N/D	#N/D	#N/D	24	C3005	Chemagic	2,344731291	40
CSM67UE7	USA	Female	76	HMP2	UC	30,9	#N/D	0	#N/D	#N/D	#N/D	#N/D	25	C3005	Chemagic	2,293710661	38
CSM67UEA	USA	Female	76	HMP2	UC	30,9	#N/D	1	#N/D	#N/D	#N/D	#N/D	27	C3005	Chemagic	2,43431766	39
CSM67UEM	USA	Female	76	HMP2	UC	30,9	#N/D	1	#N/D	#N/D	#N/D	#N/D	33	C3005	Chemagic	2,149299371	23

SampleID	country	gender	Age	study	disease	BMI	hbi	cai	cd localization	cd behaviour	uc extent	dysbiosisindex	Time point	patientID	extraction protocol	shannon	chao
CSM67UEI	USA	Female	76	HMP2	UC	30,9	#N/D	0	#N/D	#N/D	#N/D	#N/D	36	C3005	Chemagic	2,577980902	28
CSM79HO1	USA	Female	76	HMP2	UC	30,9	#N/D	0	#N/D	#N/D	#N/D	#N/D	38	C3005	Chemagic	2,157540229	27
CSM5MCTZ_P	USA	Male	32	HMP2	UC	20,1	#N/D	2	#N/D	#N/D	#N/D	#N/D	0	C3006	Chemagic	2,560120352	55
CSM5MCUG_P	USA	Male	32	HMP2	UC	20,1	#N/D	3	#N/D	#N/D	#N/D	#N/D	1	C3006	Chemagic	2,224269499	54
CSM5MCUK_P	USA	Male	32	HMP2	UC	20,1	#N/D	1	#N/D	#N/D	#N/D	#N/D	6	C3006	Chemagic	2,37150385	56
CSM5MCUO	USA	Male	32	HMP2	UC	20,1	#N/D	1	#N/D	#N/D	#N/D	#N/D	9	C3006	Chemagic	2,53460765	49
CSM5MCX3	USA	Male	32	HMP2	UC	20,1	#N/D	1	#N/D	#N/D	#N/D	#N/D	15	C3006	Chemagic	2,589368568	53
CSM67UFV	USA	Male	32	HMP2	UC	20,1	#N/D	1	#N/D	#N/D	#N/D	#N/D	23	C3006	Chemagic	2,558603218	54
CSM67UFZ	USA	Male	32	HMP2	UC	20,1	#N/D	1	#N/D	#N/D	#N/D	#N/D	25	C3006	Chemagic	2,228051002	50
CSM67UG8	USA	Male	32	HMP2	UC	20,1	#N/D	0	#N/D	#N/D	#N/D	#N/D	33	C3006	Chemagic	2,518645033	51
CSM79HMN	USA	Male	32	HMP2	UC	20,1	#N/D	0	#N/D	#N/D	#N/D	#N/D	35	C3006	Chemagic	2,461754488	45
CSM79HMP	USA	Male	32	HMP2	UC	20,1	#N/D	0	#N/D	#N/D	#N/D	#N/D	37	C3006	Chemagic	2,605807594	51
CSM79HMT	USA	Male	32	HMP2	UC	20,1	#N/D	0	#N/D	#N/D	#N/D	#N/D	41	C3006	Chemagic	2,397595809	48
CSM5MCXB_P	USA	Female	37	HMP2	UC	41,5	#N/D	2	#N/D	#N/D	#N/D	#N/D	0	C3011	Chemagic	2,680012224	63
CSM5MCYI_P	USA	Female	37	HMP2	UC	41,5	#N/D	2	#N/D	#N/D	#N/D	#N/D	1	C3011	Chemagic	2,550469963	50

SampleID	country	gender	Age	study	disease	BMI	hbi	cai	cd localization	cd behaviour	uc extent	dysbiosisindex	Time point	patientID	extraction protocol	shannon	chao
CSM5MCYM_P	USA	Female	37	HMP2	UC	41,5	#N/D	2	#N/D	#N/D	#N/D	#N/D	3	C3011	Chemagic	2,787068626	60
CSM5MCYO_P	USA	Female	37	HMP2	UC	41,5	#N/D	3	#N/D	#N/D	#N/D	#N/D	6	C3011	Chemagic	2,967801263	75
CSM5MCYQ_P	USA	Female	37	HMP2	UC	41,5	#N/D	2	#N/D	#N/D	#N/D	#N/D	8	C3011	Chemagic	2,7377997	46
CSM67UEP_P	USA	Female	37	HMP2	UC	41,5	#N/D	4	#N/D	#N/D	#N/D	#N/D	10	C3011	Chemagic	2,692116178	43
CSM67UET_P	USA	Female	37	HMP2	UC	41,5	#N/D	4	#N/D	#N/D	#N/D	#N/D	12	C3011	Chemagic	2,356723085	34
CSM67UEW_TR	USA	Female	37	HMP2	UC	41,5	#N/D	2	#N/D	#N/D	#N/D	#N/D	14	C3011	Chemagic	2,475221565	42
CSM67UEW_P	USA	Female	37	HMP2	UC	41,5	#N/D	2	#N/D	#N/D	#N/D	#N/D	14	C3011	Chemagic	2,448591043	44
CSM67UEW	USA	Female	37	HMP2	UC	41,5	#N/D	2	#N/D	#N/D	#N/D	#N/D	14	C3011	Chemagic	2,520796583	42
CSM67UF1_P	USA	Female	37	HMP2	UC	41,5	#N/D	1	#N/D	#N/D	#N/D	#N/D	16	C3011	Chemagic	2,776865963	50
CSM67UF1	USA	Female	37	HMP2	UC	41,5	#N/D	1	#N/D	#N/D	#N/D	#N/D	16	C3011	Chemagic	2,793043487	44
CSM67UF5	USA	Female	37	HMP2	UC	41,5	#N/D	1	#N/D	#N/D	#N/D	#N/D	19	C3011	Chemagic	2,743021023	42
CSM79HM1	USA	Female	37	HMP2	UC	41,5	#N/D	1	#N/D	#N/D	#N/D	#N/D	24	C3011	Chemagic	2,637983531	49
CSM79HQT_P	USA	Female	37	HMP2	UC	41,5	#N/D	1	#N/D	#N/D	#N/D	#N/D	27	C3011	Chemagic	2,737040164	51
CSM79HM5_P	USA	Female	37	HMP2	UC	41,5	#N/D	2	#N/D	#N/D	#N/D	#N/D	31	C3011	Chemagic	2,434348438	32
CSM79HM7	USA	Female	37	HMP2	UC	41,5	#N/D	1	#N/D	#N/D	#N/D	#N/D	32	C3011	Chemagic	2,565883106	48,5

SampleID	country	gender	Age	study	disease	BMI	hbi	cai	cd localization	cd behaviour	uc extent	dysbiosisindex	Time point	patientID	extraction protocol	shannon	chao
CSM79HM9_P	USA	Female	37	HMP2	UC	41,5	#N/D	1	#N/D	#N/D	#N/D	#N/D	34	C3011	Chemagic	2,657237269	73
CSM7KOMP	USA	Female	37	HMP2	UC	41,5	#N/D	2	#N/D	#N/D	#N/D	#N/D	37	C3011	Chemagic	2,744186293	55
CSM7KOMR_P	USA	Female	37	HMP2	UC	41,5	#N/D	4	#N/D	#N/D	#N/D	#N/D	39	C3011	Chemagic	2,590312562	50
CSM7KOMT	USA	Female	37	HMP2	UC	41,5	#N/D	1	#N/D	#N/D	#N/D	#N/D	41	C3011	Chemagic	2,844404951	51
CSM7KOMV_P	USA	Female	37	HMP2	UC	41,5	#N/D	1	#N/D	#N/D	#N/D	#N/D	44	C3011	Chemagic	2,839986437	48
CSM5MCYU_P	USA	Female	26	HMP2	UC	22,2	#N/D	3	#N/D	#N/D	#N/D	#N/D	0	C3013	Chemagic	1,619155605	26
CSM67U9H_P	USA	Female	26	HMP2	UC	22,2	#N/D	3	#N/D	#N/D	#N/D	#N/D	2	C3013	Chemagic	0,963710957	34
CSM67U9H	USA	Female	26	HMP2	UC	22,2	#N/D	3	#N/D	#N/D	#N/D	#N/D	2	C3013	Chemagic	0,987230841	28
CSM67U9N	USA	Female	26	HMP2	UC	22,2	#N/D	8	#N/D	#N/D	#N/D	#N/D	6	C3013	Chemagic	2,463414326	22
CSM67U9P_P	USA	Female	26	HMP2	UC	22,2	#N/D	5	#N/D	#N/D	#N/D	#N/D	8	C3013	Chemagic	2,290701043	22
CSM67U9R_P	USA	Female	26	HMP2	UC	22,2	#N/D	3	#N/D	#N/D	#N/D	#N/D	10	C3013	Chemagic	2,108988283	26
CSM79HGD_P	USA	Female	26	HMP2	UC	22,2	#N/D	3	#N/D	#N/D	#N/D	#N/D	12	C3013	Chemagic	2,703319023	32
CSM79HGF_P	USA	Female	26	HMP2	UC	22,2	#N/D	3	#N/D	#N/D	#N/D	#N/D	14	C3013	Chemagic	2,656992821	35
CSM79HGF	USA	Female	26	HMP2	UC	22,2	#N/D	3	#N/D	#N/D	#N/D	#N/D	14	C3013	Chemagic	2,657011626	35
CSM79HGH_P	USA	Female	26	HMP2	UC	22,2	#N/D	3	#N/D	#N/D	#N/D	#N/D	17	C3013	Chemagic	2,478300323	22

SampleID	country	gender	Age	study	disease	BMI	hbi	cai	cd localization	cd behaviour	uc extent	dysbiosisindex	Time point	patientID	extraction protocol	shannon	chao
CSM79HGJ_P	USA	Female	26	HMP2	UC	22,2	#N/D	5	#N/D	#N/D	#N/D	#N/D	18	C3013	Chemagic	1,832001424	19
CSM79HGL_P	USA	Female	26	HMP2	UC	22,2	#N/D	4	#N/D	#N/D	#N/D	#N/D	20	C3013	Chemagic	2,5143392	27
CSM79HGN_P	USA	Female	26	HMP2	UC	22,2	#N/D	5	#N/D	#N/D	#N/D	#N/D	22	C3013	Chemagic	0,829953405	28
CSM79HPK	USA	Female	26	HMP2	UC	22,2	#N/D	6	#N/D	#N/D	#N/D	#N/D	24	C3013	Chemagic	0,837583405	34
CSM79HPM_P	USA	Female	26	HMP2	UC	22,2	#N/D	5	#N/D	#N/D	#N/D	#N/D	26	C3013	Chemagic	2,006003489	25
CSM79HPS	USA	Female	26	HMP2	UC	22,2	#N/D	7	#N/D	#N/D	#N/D	#N/D	31	C3013	Chemagic	1,327166948	34
CSM79HPQ_P	USA	Female	26	HMP2	UC	22,2	#N/D	9	#N/D	#N/D	#N/D	#N/D	32	C3013	Chemagic	1,085741208	34
CSM79HPO	USA	Female	26	HMP2	UC	22,2	#N/D	8	#N/D	#N/D	#N/D	#N/D	33	C3013	Chemagic	1,293687591	10
CSM79HPU	USA	Female	26	HMP2	UC	22,2	#N/D	9	#N/D	#N/D	#N/D	#N/D	34	C3013	Chemagic	0,874845286	4
CSM7KONS_P	USA	Female	26	HMP2	UC	22,2	#N/D	5	#N/D	#N/D	#N/D	#N/D	36	C3013	Chemagic	0,721394076	28
CSM7KONU	USA	Female	26	HMP2	UC	22,2	#N/D	7	#N/D	#N/D	#N/D	#N/D	38	C3013	Chemagic	2,037752966	18
CSM7KONW_P	USA	Female	26	HMP2	UC	22,2	#N/D	6	#N/D	#N/D	#N/D	#N/D	41	C3013	Chemagic	2,311938115	54
CSM67U9T_P	USA	Female	50	HMP2	UC	22,4	#N/D	3	#N/D	#N/D	#N/D	#N/D	0	C3015	Chemagic	2,0156189	32
CSM67UAK	USA	Female	50	HMP2	UC	22,4	#N/D	4	#N/D	#N/D	#N/D	#N/D	3	C3015	Chemagic	1,91222625	25
CSM67UAM	USA	Female	50	HMP2	UC	22,4	#N/D	0	#N/D	#N/D	#N/D	#N/D	4	C3015	Chemagic	1,922522703	24

SampleID	country	gender	Age	study	disease	BMI	hbi	cai	cd localization	cd behaviour	uc extent	dysbiosisindex	Time point	patientID	extraction protocol	shannon	chao
CSM67UAO	USA	Female	50	HMP2	UC	22,4	#N/D	1	#N/D	#N/D	#N/D	#N/D	6	C3015	Chemagic	1,906007139	35
CSM67UAQ	USA	Female	50	HMP2	UC	22,4	#N/D	0	#N/D	#N/D	#N/D	#N/D	8	C3015	Chemagic	1,768447809	25
CSM67UAS	USA	Female	50	HMP2	UC	22,4	#N/D	1	#N/D	#N/D	#N/D	#N/D	10	C3015	Chemagic	2,003495714	25
CSM79HID	USA	Female	50	HMP2	UC	22,4	#N/D	0	#N/D	#N/D	#N/D	#N/D	12	C3015	Chemagic	1,931869209	23
CSM79HIF	USA	Female	50	HMP2	UC	22,4	#N/D	1	#N/D	#N/D	#N/D	#N/D	14	C3015	Chemagic	1,746879534	28
CSM79HIH	USA	Female	50	HMP2	UC	22,4	#N/D	1	#N/D	#N/D	#N/D	#N/D	16	C3015	Chemagic	1,926346855	24
CSM79HIJ	USA	Female	50	HMP2	UC	22,4	#N/D	0	#N/D	#N/D	#N/D	#N/D	18	C3015	Chemagic	1,774107979	34
CSM79HIL	USA	Female	50	HMP2	UC	22,4	#N/D	0	#N/D	#N/D	#N/D	#N/D	20	C3015	Chemagic	1,544175727	24
CSM79HIN	USA	Female	50	HMP2	UC	22,4	#N/D	0	#N/D	#N/D	#N/D	#N/D	22	C3015	Chemagic	2,073510801	25
CSM7KOJQ	USA	Female	50	HMP2	UC	22,4	#N/D	1	#N/D	#N/D	#N/D	#N/D	24	C3015	Chemagic	1,818897098	25
CSM7KOJS	USA	Female	50	HMP2	UC	22,4	#N/D	0	#N/D	#N/D	#N/D	#N/D	26	C3015	Chemagic	1,63734571	26
CSM7KOJU	USA	Female	50	HMP2	UC	22,4	#N/D	0	#N/D	#N/D	#N/D	#N/D	28	C3015	Chemagic	1,456869046	28
CSM7KOJW	USA	Female	50	HMP2	UC	22,4	#N/D	0	#N/D	#N/D	#N/D	#N/D	30	C3015	Chemagic	2,090109594	25
CSM7KOJY	USA	Female	50	HMP2	UC	22,4	#N/D	0	#N/D	#N/D	#N/D	#N/D	32	C3015	Chemagic	1,646532886	29
CSM7KOK1	USA	Female	50	HMP2	UC	22,4	#N/D	0	#N/D	#N/D	#N/D	#N/D	34	C3015	Chemagic	1,62428657	28

SampleID	country	gender	Age	study	disease	BMI	hbi	cai	cd localization	cd behaviour	uc extent	dysbiosisindex	Time point	patientID	extraction protocol	shannon	chao
CSM7KOPG	USA	Female	50	HMP2	UC	22,4	#N/D	0	#N/D	#N/D	#N/D	#N/D	36	C3015	Chemagic	1,7206411	28
CSM7KOPI	USA	Female	50	HMP2	UC	22,4	#N/D	1	#N/D	#N/D	#N/D	#N/D	38	C3015	Chemagic	1,892373076	28
CSM7KOPM	USA	Female	50	HMP2	UC	22,4	#N/D	1	#N/D	#N/D	#N/D	#N/D	42	C3015	Chemagic	1,75321871	33
CSM7KOPO	USA	Female	50	HMP2	UC	22,4	#N/D	0	#N/D	#N/D	#N/D	#N/D	44	C3015	Chemagic	2,043961266	31
CSM7KOP6	USA	Female	42	HMP2	UC	21,7	#N/D	2	#N/D	#N/D	#N/D	#N/D	2	C3032	Chemagic	2,547624262	45
CSM7KOP8	USA	Female	42	HMP2	UC	21,7	#N/D	1	#N/D	#N/D	#N/D	#N/D	4	C3032	Chemagic	2,580582222	49,75
CSM7KOPE	USA	Female	42	HMP2	UC	21,7	#N/D	1	#N/D	#N/D	#N/D	#N/D	10	C3032	Chemagic	2,459710896	46
CSM7KOU7_P	USA	Female	42	HMP2	UC	21,7	#N/D	0	#N/D	#N/D	#N/D	#N/D	12	C3032	Chemagic	2,618399996	49
CSM7KOU9	USA	Female	42	HMP2	UC	21,7	#N/D	0	#N/D	#N/D	#N/D	#N/D	14	C3032	Chemagic	2,547407076	49
CSM7KOUB	USA	Female	42	HMP2	UC	21,7	#N/D	0	#N/D	#N/D	#N/D	#N/D	16	C3032	Chemagic	2,370162049	44
CSM9X1ZO	USA	Female	42	HMP2	UC	21,7	#N/D	1	#N/D	#N/D	#N/D	#N/D	24	C3032	Chemagic	2,609919855	53
CSM9X1ZQ	USA	Female	42	HMP2	UC	21,7	#N/D	0	#N/D	#N/D	#N/D	#N/D	26	C3032	Chemagic	2,347292445	47
CSM9X1ZY	USA	Female	42	HMP2	UC	21,7	#N/D	0	#N/D	#N/D	#N/D	#N/D	33	C3032	Chemagic	2,471896845	39
CSM9X22G	USA	Female	42	HMP2	UC	21,7	#N/D	0	#N/D	#N/D	#N/D	#N/D	37	C3032	Chemagic	2,501494725	54
CSM9X22I	USA	Female	42	HMP2	UC	21,7	#N/D	1	#N/D	#N/D	#N/D	#N/D	38	C3032	Chemagic	2,546876917	50

SampleID	country	gender	Age	study	disease	BMI	hbi	cai	cd localization	cd behaviour	uc extent	dysbiosisindex	Time point	patientID	extraction protocol	shannon	chao
CSM9X22K	USA	Female	42	HMP2	UC	21,7	#N/D	0	#N/D	#N/D	#N/D	#N/D	40	C3032	Chemagic	2,551051593	42
CSM79HQR_P	USA	Female	36	HMP2	UC	19,5	#N/D	6	#N/D	#N/D	#N/D	#N/D	0	C3034	Chemagic	2,404831121	29
CSM7KORO	USA	Female	36	HMP2	UC	19,5	#N/D	4	#N/D	#N/D	#N/D	#N/D	2	C3034	Chemagic	2,231601445	33
CSM7KORM	USA	Female	36	HMP2	UC	19,5	#N/D	2	#N/D	#N/D	#N/D	#N/D	4	C3034	Chemagic	2,3276769	43
CSM7KORU	USA	Female	36	HMP2	UC	19,5	#N/D	2	#N/D	#N/D	#N/D	#N/D	8	C3034	Chemagic	1,978010621	30
CSM7KORS	USA	Female	36	HMP2	UC	19,5	#N/D	2	#N/D	#N/D	#N/D	#N/D	10	C3034	Chemagic	2,156644058	30
CSM9X1XU	USA	Female	36	HMP2	UC	19,5	#N/D	2	#N/D	#N/D	#N/D	#N/D	14	C3034	Chemagic	2,031957675	26
CSM9X1Y3	USA	Female	36	HMP2	UC	19,5	#N/D	1	#N/D	#N/D	#N/D	#N/D	26	C3034	Chemagic	2,394354627	35
CSM9X21J	USA	Female	36	HMP2	UC	19,5	#N/D	2	#N/D	#N/D	#N/D	#N/D	34	C3034	Chemagic	2,25853244	42
CSM9X21L	USA	Female	36	HMP2	UC	19,5	#N/D	3	#N/D	#N/D	#N/D	#N/D	36	C3034	Chemagic	2,110202766	37
CSM9X21N	USA	Female	36	HMP2	UC	19,5	#N/D	3	#N/D	#N/D	#N/D	#N/D	38	C3034	Chemagic	2,083173943	38,33333333
CSM7KOUJ_P	USA	Female	46	HMP2	UC	22,4	#N/D	0	#N/D	#N/D	#N/D	#N/D	2	C3037	Chemagic	2,091443303	41
CSM7KOUL	USA	Female	46	HMP2	UC	22,4	#N/D	0	#N/D	#N/D	#N/D	#N/D	4	C3037	Chemagic	2,178693314	40
CSM7KOUN	USA	Female	46	HMP2	UC	22,4	#N/D	1	#N/D	#N/D	#N/D	#N/D	6	C3037	Chemagic	2,404037613	46
CSM9X219	USA	Female	46	HMP2	UC	22,4	#N/D	1	#N/D	#N/D	#N/D	#N/D	13	C3037	Chemagic	2,111027397	32

SampleID	country	gender	Age	study	disease	BMI	hbi	cai	cd localization	cd behaviour	uc extent	dysbiosisindex	Time point	patientID	extraction protocol	shannon	chao
CSM9X213	USA	Female	46	HMP2	UC	22,4	#N/D	0	#N/D	#N/D	#N/D	#N/D	14	C3037	Chemagic	2,421315938	48
CSM9X215	USA	Female	46	HMP2	UC	22,4	#N/D	0	#N/D	#N/D	#N/D	#N/D	16	C3037	Chemagic	2,169327671	38
CSM9X211	USA	Female	46	HMP2	UC	22,4	#N/D	0	#N/D	#N/D	#N/D	#N/D	22	C3037	Chemagic	2,374501816	35
CSM9X22S	USA	Female	46	HMP2	UC	22,4	#N/D	0	#N/D	#N/D	#N/D	#N/D	32	C3037	Chemagic	2,185375308	36
CSM9X22U	USA	Female	46	HMP2	UC	22,4	#N/D	2	#N/D	#N/D	#N/D	#N/D	34	C3037	Chemagic	2,062937044	34
CSM9X23H	USA	Female	46	HMP2	UC	22,4	#N/D	1	#N/D	#N/D	#N/D	#N/D	38	C3037	Chemagic	1,983595801	28
CSM9X23N	USA	Female	46	HMP2	UC	22,4	#N/D	3	#N/D	#N/D	#N/D	#N/D	44	C3037	Chemagic	1,952032106	29
ESM5MEEJ_P	USA	Female	7	HMP2	UC	16,3	#N/D	2	#N/D	#N/D	#N/D	#N/D	1	E5004	Chemagic	2,166442316	37
ESM5GEZ4_P	USA	Female	7	HMP2	UC	16,3	#N/D	1	#N/D	#N/D	#N/D	#N/D	3	E5004	Chemagic	2,272185558	45
ESM5GEZ6_P	USA	Female	7	HMP2	UC	16,3	#N/D	2	#N/D	#N/D	#N/D	#N/D	5	E5004	Chemagic	2,804020849	60
ESM5GEZA_P	USA	Female	7	HMP2	UC	16,3	#N/D	2	#N/D	#N/D	#N/D	#N/D	9	E5004	Chemagic	2,335322418	44
ESM5MEBS	USA	Female	7	HMP2	UC	16,3	#N/D	1	#N/D	#N/D	#N/D	#N/D	13	E5004	Chemagic	2,270946306	42
ESM5MEBU	USA	Female	7	HMP2	UC	16,3	#N/D	2	#N/D	#N/D	#N/D	#N/D	15	E5004	Chemagic	2,343475795	42
ESM5MEC3	USA	Female	7	HMP2	UC	16,3	#N/D	2	#N/D	#N/D	#N/D	#N/D	24	E5004	Chemagic	2,458563524	44
ESM5MEC5	USA	Female	7	HMP2	UC	16,3	#N/D	1	#N/D	#N/D	#N/D	#N/D	26	E5004	Chemagic	2,065373301	38

SampleID	country	gender	Age	study	disease	BMI	hbi	cai	cd localization	cd behaviour	uc extent	dysbiosisindex	Time point	patientID	extraction protocol	shannon	chao
ESM5MEDU	USA	Female	7	HMP2	UC	16,3	#N/D	2	#N/D	#N/D	#N/D	#N/D	32	E5004	Chemagic	2,389638271	42
ESM5MEC9	USA	Female	7	HMP2	UC	16,3	#N/D	0	#N/D	#N/D	#N/D	#N/D	36	E5004	Chemagic	1,72525392	22
ESM718SY	USA	Female	7	HMP2	UC	16,3	#N/D	1	#N/D	#N/D	#N/D	#N/D	39	E5004	Chemagic	2,268213584	44
ESM5ME9U	USA	Female	7	HMP2	UC	16,3	#N/D	2	#N/D	#N/D	#N/D	#N/D	42	E5004	Chemagic	2,42065955	42,5
HSM5MD87_P	USA	Male	13	HMP2	UC	22	#N/D	1	#N/D	#N/D	#N/D	#N/D	0	H4010	Chemagic	2,518078731	49
HSM5MD47_P	USA	Male	13	HMP2	UC	22	#N/D	1	#N/D	#N/D	#N/D	#N/D	2	H4010	Chemagic	1,957775136	21
HSM5MD44_P	USA	Male	13	HMP2	UC	22	#N/D	0	#N/D	#N/D	#N/D	#N/D	4	H4010	Chemagic	2,292416855	24
HSM5MD43_P	USA	Male	13	HMP2	UC	22	#N/D	0	#N/D	#N/D	#N/D	#N/D	6	H4010	Chemagic	2,143122597	49
HSM5MD43	USA	Male	13	HMP2	UC	22	#N/D	0	#N/D	#N/D	#N/D	#N/D	6	H4010	Chemagic	2,298831344	47
HSM5MD3Y	USA	Male	13	HMP2	UC	22	#N/D	0	#N/D	#N/D	#N/D	#N/D	11	H4010	Chemagic	2,591257477	41
HSM5MD41	USA	Male	13	HMP2	UC	22	#N/D	0	#N/D	#N/D	#N/D	#N/D	14	H4010	Chemagic	2,297730396	46
HSM6XRTM	USA	Male	13	HMP2	UC	22	#N/D	3	#N/D	#N/D	#N/D	#N/D	22	H4010	Chemagic	2,439158173	45
HSM6XRTQ	USA	Male	13	HMP2	UC	22	#N/D	0	#N/D	#N/D	#N/D	#N/D	26	H4010	Chemagic	2,054597855	35
HSM6XRTO	USA	Male	13	HMP2	UC	22	#N/D	0	#N/D	#N/D	#N/D	#N/D	33	H4010	Chemagic	2,352526117	45
HSM6XRTS	USA	Male	13	HMP2	UC	22	#N/D	0	#N/D	#N/D	#N/D	#N/D	35	H4010	Chemagic	2,391377615	45

SampleID	country	gender	Age	study	disease	BMI	hbi	cai	cd localization	cd behaviour	uc extent	dysbiosisindex	Time point	patientID	extraction protocol	shannon	chao
HSM67VGY	USA	Male	13	HMP2	UC	22	#N/D	0	#N/D	#N/D	#N/D	#N/D	36	H4010	Chemagic	2,427173558	49
HSM67VH1	USA	Male	13	HMP2	UC	22	#N/D	1	#N/D	#N/D	#N/D	#N/D	47	H4010	Chemagic	2,27783589	44
HSM6XRQE_P	USA	Female	11	HMP2	UC	16,9	#N/D	4	#N/D	#N/D	#N/D	#N/D	0	H4019	Chemagic	1,459223838	5
HSM6XRR5	USA	Female	11	HMP2	UC	16,9	#N/D	4	#N/D	#N/D	#N/D	#N/D	2	H4019	Chemagic	2,700551581	49
HSM6XRR7	USA	Female	11	HMP2	UC	16,9	#N/D	1	#N/D	#N/D	#N/D	#N/D	4	H4019	Chemagic	2,498286957	49
HSM6XRRB	USA	Female	11	HMP2	UC	16,9	#N/D	3	#N/D	#N/D	#N/D	#N/D	9	H4019	Chemagic	0,605329855	18
HSM6XRRD	USA	Female	11	HMP2	UC	16,9	#N/D	1	#N/D	#N/D	#N/D	#N/D	10	H4019	Chemagic	2,122841931	35
HSM6XRUX	USA	Female	11	HMP2	UC	16,9	#N/D	1	#N/D	#N/D	#N/D	#N/D	12	H4019	Chemagic	2,687029876	52
HSM6XRUZ	USA	Female	11	HMP2	UC	16,9	#N/D	1	#N/D	#N/D	#N/D	#N/D	14	H4019	Chemagic	2,501971806	42
HSM6XRV2	USA	Female	11	HMP2	UC	16,9	#N/D	1	#N/D	#N/D	#N/D	#N/D	16	H4019	Chemagic	2,729806019	46
HSM6XRV4	USA	Female	11	HMP2	UC	16,9	#N/D	2	#N/D	#N/D	#N/D	#N/D	18	H4019	Chemagic	2,43768627	46
HSM6XRV6	USA	Female	11	HMP2	UC	16,9	#N/D	1	#N/D	#N/D	#N/D	#N/D	20	H4019	Chemagic	2,65446762	52
HSM6XRV8	USA	Female	11	HMP2	UC	16,9	#N/D	1	#N/D	#N/D	#N/D	#N/D	22	H4019	Chemagic	2,635738394	56
HSM7J4PC	USA	Female	11	HMP2	UC	16,9	#N/D	0	#N/D	#N/D	#N/D	#N/D	24	H4019	Chemagic	2,619024148	46
HSM7J4PE	USA	Female	11	HMP2	UC	16,9	#N/D	0	#N/D	#N/D	#N/D	#N/D	28	H4019	Chemagic	2,436078314	47

SampleID	country	gender	Age	study	disease	BMI	hbi	cai	cd localization	cd behaviour	uc extent	dysbiosisindex	Time point	patientID	extraction protocol	shannon	chao
HSM7J4PG	USA	Female	11	HMP2	UC	16,9	#N/D	0	#N/D	#N/D	#N/D	#N/D	29	H4019	Chemagic	2,518994668	60
HSM7J4PI	USA	Female	11	HMP2	UC	16,9	#N/D	0	#N/D	#N/D	#N/D	#N/D	31	H4019	Chemagic	2,36299404	47
HSM7J4PK	USA	Female	11	HMP2	UC	16,9	#N/D	1	#N/D	#N/D	#N/D	#N/D	33	H4019	Chemagic	1,090568075	5
HSM7J4PM	USA	Female	11	HMP2	UC	16,9	#N/D	2	#N/D	#N/D	#N/D	#N/D	36	H4019	Chemagic	2,372630226	50
HSM7J4M4	USA	Female	11	HMP2	UC	16,9	#N/D	1	#N/D	#N/D	#N/D	#N/D	37	H4019	Chemagic	2,144106716	45
HSM7J4M6	USA	Female	11	HMP2	UC	16,9	#N/D	2	#N/D	#N/D	#N/D	#N/D	38	H4019	Chemagic	2,252528397	39
HSM7J4M8	USA	Female	11	HMP2	UC	16,9	#N/D	2	#N/D	#N/D	#N/D	#N/D	40	H4019	Chemagic	2,597631254	49
HSM7J4MA	USA	Female	11	HMP2	UC	16,9	#N/D	2	#N/D	#N/D	#N/D	#N/D	43	H4019	Chemagic	2,482174516	54
HSM7J4MC	USA	Female	11	HMP2	UC	16,9	#N/D	2	#N/D	#N/D	#N/D	#N/D	45	H4019	Chemagic	2,611776778	55
HSM7J4ME	USA	Female	11	HMP2	UC	16,9	#N/D	2	#N/D	#N/D	#N/D	#N/D	46	H4019	Chemagic	2,479389721	50
HSM7J4GR	USA	Male	15	HMP2	UC	14,9	#N/D	3	L2+L4	#N/D	#N/D	#N/D	17	H4027	Chemagic	1,828259634	50,5
HSM7J4NC	USA	Male	15	HMP2	UC	14,9	#N/D	5	L2+L4	#N/D	#N/D	#N/D	24	H4027	Chemagic	2,571594192	44
HSM7J4NE	USA	Male	15	HMP2	UC	14,9	#N/D	2	L2+L4	#N/D	#N/D	#N/D	25	H4027	Chemagic	2,412422546	43
HSM7J4NM	USA	Male	15	HMP2	UC	14,9	#N/D	0	L2+L4	#N/D	#N/D	#N/D	33	H4027	Chemagic	2,581883826	47
HSMA33O1	USA	Male	15	HMP2	UC	14,9	#N/D	1	L2+L4	#N/D	#N/D	#N/D	35	H4027	Chemagic	2,28549774	41

SampleID	country	gender	Age	study	disease	BMI	hbi	cai	cd localization	cd behaviour	uc extent	dysbiosisindex	Time point	patientID	extraction protocol	shannon	chao
HSMA33O3	USA	Male	15	HMP2	UC	14,9	#N/D	2	L2+L4	#N/D	#N/D	#N/D	37	H4027	Chemagic	1,978059882	29
HSM7CZ2X_P	USA	Male	16	HMP2	UC	31	#N/D	3	#N/D	#N/D	#N/D	#N/D	0	H4035	Chemagic	1,663803566	30
HSM7J4HM	USA	Male	16	HMP2	UC	31	#N/D	1	#N/D	#N/D	#N/D	#N/D	2	H4035	Chemagic	2,137944624	35
HSM7J4HO	USA	Male	16	HMP2	UC	31	#N/D	2	#N/D	#N/D	#N/D	#N/D	4	H4035	Chemagic	2,128552103	38
HSM7J4HQ	USA	Male	16	HMP2	UC	31	#N/D	1	#N/D	#N/D	#N/D	#N/D	6	H4035	Chemagic	1,410438434	41
HSM7J4HS	USA	Male	16	HMP2	UC	31	#N/D	1	#N/D	#N/D	#N/D	#N/D	8	H4035	Chemagic	2,18357655	31
HSM7J4HU	USA	Male	16	HMP2	UC	31	#N/D	2	#N/D	#N/D	#N/D	#N/D	10	H4035	Chemagic	2,069214851	28
HSM7J4JZ	USA	Male	16	HMP2	UC	31	#N/D	1	#N/D	#N/D	#N/D	#N/D	12	H4035	Chemagic	2,20794023	28
HSM7J4K2	USA	Male	16	HMP2	UC	31	#N/D	1	#N/D	#N/D	#N/D	#N/D	15	H4035	Chemagic	2,238312764	35
HSM7J4K4	USA	Male	16	HMP2	UC	31	#N/D	2	#N/D	#N/D	#N/D	#N/D	16	H4035	Chemagic	1,802423544	14
HSM7J4K6	USA	Male	16	HMP2	UC	31	#N/D	3	#N/D	#N/D	#N/D	#N/D	18	H4035	Chemagic	2,233108296	42
HSM7J4K8	USA	Male	16	HMP2	UC	31	#N/D	2	#N/D	#N/D	#N/D	#N/D	20	H4035	Chemagic	2,315383305	35
HSM7J4KA	USA	Male	16	HMP2	UC	31	#N/D	3	#N/D	#N/D	#N/D	#N/D	22	H4035	Chemagic	1,564975514	9
HSMA33OP	USA	Male	16	HMP2	UC	31	#N/D	2	#N/D	#N/D	#N/D	#N/D	24	H4035	Chemagic	2,05072313	27
HSMA33OR	USA	Male	16	HMP2	UC	31	#N/D	5	#N/D	#N/D	#N/D	#N/D	26	H4035	Chemagic	2,022857165	32

SampleID	country	gender	Age	study	disease	BMI	hbi	cai	cd localization	cd behaviour	uc extent	dysbiosisindex	Time point	patientID	extraction protocol	shannon	chao
HSMA33OT	USA	Male	16	HMP2	UC	31	#N/D	1	#N/D	#N/D	#N/D	#N/D	28	H4035	Chemagic	1,999355413	24
HSMA33OV	USA	Male	16	HMP2	UC	31	#N/D	0	#N/D	#N/D	#N/D	#N/D	30	H4035	Chemagic	2,359741087	42
HSMA33OX	USA	Male	16	HMP2	UC	31	#N/D	0	#N/D	#N/D	#N/D	#N/D	32	H4035	Chemagic	2,39164601	46
HSMA33OZ	USA	Male	16	HMP2	UC	31	#N/D	0	#N/D	#N/D	#N/D	#N/D	34	H4035	Chemagic	1,983357082	44
HSMA33MA	USA	Male	16	HMP2	UC	31	#N/D	1	#N/D	#N/D	#N/D	#N/D	37	H4035	Chemagic	2,568186625	48
HSMA33MC	USA	Male	16	HMP2	UC	31	#N/D	2	#N/D	#N/D	#N/D	#N/D	38	H4035	Chemagic	2,456205764	35
HSMA33ME	USA	Male	16	HMP2	UC	31	#N/D	1	#N/D	#N/D	#N/D	#N/D	40	H4035	Chemagic	2,208716533	30
HSMA33MG	USA	Male	16	HMP2	UC	31	#N/D	2	#N/D	#N/D	#N/D	#N/D	42	H4035	Chemagic	1,98569009	26
HSMA33MI	USA	Male	16	HMP2	UC	31	#N/D	1	#N/D	#N/D	#N/D	#N/D	44	H4035	Chemagic	1,520172739	17
HSMA33MK	USA	Male	16	HMP2	UC	31	#N/D	5	#N/D	#N/D	#N/D	#N/D	46	H4035	Chemagic	2,021495147	30
HSM7J4JT_P	USA	Female	17	HMP2	UC	18,6	#N/D	0	#N/D	#N/D	#N/D	#N/D	0	H4040	Chemagic	1,673234503	35
HSM7J4NO	USA	Female	17	HMP2	UC	18,6	#N/D	1	#N/D	#N/D	#N/D	#N/D	2	H4040	Chemagic	1,198567513	37
HSM7J4NS	USA	Female	17	HMP2	UC	18,6	#N/D	0	#N/D	#N/D	#N/D	#N/D	7	H4040	Chemagic	2,495579512	53
HSM7J4NU	USA	Female	17	HMP2	UC	18,6	#N/D	0	#N/D	#N/D	#N/D	#N/D	9	H4040	Chemagic	2,850288573	55
HSMA33OD	USA	Female	17	HMP2	UC	18,6	#N/D	1	#N/D	#N/D	#N/D	#N/D	16	H4040	Chemagic	2,514726763	48,5

SampleID	country	gender	Age	study	disease	BMI	hbi	cai	cd localization	cd behaviour	uc extent	dysbiosisindex	Time point	patientID	extraction protocol	shannon	chao
HSMA33OJ	USA	Female	17	HMP2	UC	18,6	#N/D	1	#N/D	#N/D	#N/D	#N/D	22	H4040	Chemagic	2,215712543	50
HSMA33OL	USA	Female	17	HMP2	UC	18,6	#N/D	3	#N/D	#N/D	#N/D	#N/D	26	H4040	Chemagic	1,469099814	44
HSMA33LX	USA	Female	17	HMP2	UC	18,6	#N/D	1	#N/D	#N/D	#N/D	#N/D	34	H4040	Chemagic	2,550321317	54
HSMA33LZ	USA	Female	17	HMP2	UC	18,6	#N/D	2	#N/D	#N/D	#N/D	#N/D	37	H4040	Chemagic	1,988425391	36
HSMA33M2	USA	Female	17	HMP2	UC	18,6	#N/D	2	#N/D	#N/D	#N/D	#N/D	38	H4040	Chemagic	2,53588151	48
HSMA33M8	USA	Female	17	HMP2	UC	18,6	#N/D	0	#N/D	#N/D	#N/D	#N/D	44	H4040	Chemagic	1,369725985	46
HSM7J4JV_P	USA	Male	15	HMP2	UC	30,7	#N/D	6	#N/D	#N/D	#N/D	#N/D	0	H4042	Chemagic	1,242664019	35
HSMA33MX	USA	Male	15	HMP2	UC	30,7	#N/D	7	#N/D	#N/D	#N/D	#N/D	2	H4042	Chemagic	0,867523054	4
HSMA33MZ	USA	Male	15	HMP2	UC	30,7	#N/D	7	#N/D	#N/D	#N/D	#N/D	4	H4042	Chemagic	0,528946916	28
HSMA33N4	USA	Male	15	HMP2	UC	30,7	#N/D	7	#N/D	#N/D	#N/D	#N/D	8	H4042	Chemagic	0,787053815	3
HSMA33IS	USA	Male	15	HMP2	UC	30,7	#N/D	8	#N/D	#N/D	#N/D	#N/D	25	H4042	Chemagic	2,62348336	39
HSMA33RF	USA	Male	15	HMP2	UC	30,7	#N/D	8	#N/D	#N/D	#N/D	#N/D	39	H4042	Chemagic	1,905236326	44
HSMA33NW	USA	Male	16	HMP2	UC	22,3	#N/D	0	#N/D	#N/D	#N/D	#N/D	0	H4044	Chemagic	2,401399669	40
HSMA33P2_P	USA	Male	16	HMP2	UC	22,3	#N/D	3	#N/D	#N/D	#N/D	#N/D	2	H4044	Chemagic	2,266742325	50
HSMA33IA	USA	Male	16	HMP2	UC	22,3	#N/D	1	#N/D	#N/D	#N/D	#N/D	6	H4044	Chemagic	1,92532612	33

SampleID	country	gender	Age	study	disease	BMI	hbi	cai	cd localization	cd behaviour	uc extent	dysbiosisindex	Time point	patientID	extraction protocol	shannon	chao
HSMA33LB	USA	Male	16	HMP2	UC	22,3	#N/D	3	#N/D	#N/D	#N/D	#N/D	16	H4044	Chemagic	1,842364576	22
HSMA33LJ	USA	Male	16	HMP2	UC	22,3	#N/D	3	#N/D	#N/D	#N/D	#N/D	24	H4044	Chemagic	2,301172628	35
MSM5LLIQ_P	USA	Female	21	HMP2	UC	20,3	#N/D	7	#N/D	#N/D	#N/D	#N/D	0	M2026	Chemagic	0,859936464	5
MSM5LLFK_P	USA	Female	21	HMP2	UC	20,3	#N/D	4	#N/D	#N/D	#N/D	#N/D	2	M2026	Chemagic	1,234708354	15
MSM5LLFM_P	USA	Female	21	HMP2	UC	20,3	#N/D	1	#N/D	#N/D	#N/D	#N/D	4	M2026	Chemagic	1,320528639	23
MSM5LLFO_P	USA	Female	21	HMP2	UC	20,3	#N/D	0	#N/D	#N/D	#N/D	#N/D	6	M2026	Chemagic	1,13264024	23
MSM5LLFU_P	USA	Female	21	HMP2	UC	20,3	#N/D	3	#N/D	#N/D	#N/D	#N/D	12	M2026	Chemagic	1,398048434	21
MSM6J2HD	USA	Female	21	HMP2	UC	20,3	#N/D	4	#N/D	#N/D	#N/D	#N/D	17	M2026	Chemagic	1,243735165	22
MSM6J2HF	USA	Female	21	HMP2	UC	20,3	#N/D	3	#N/D	#N/D	#N/D	#N/D	18	M2026	Chemagic	1,287177269	28
MSM6J2HH	USA	Female	21	HMP2	UC	20,3	#N/D	7	#N/D	#N/D	#N/D	#N/D	20	M2026	Chemagic	1,69076956	15
MSM6J2HJ	USA	Female	21	HMP2	UC	20,3	#N/D	0	#N/D	#N/D	#N/D	#N/D	22	M2026	Chemagic	1,771249478	21
MSM6J2HL	USA	Female	21	HMP2	UC	20,3	#N/D	0	#N/D	#N/D	#N/D	#N/D	24	M2026	Chemagic	1,5176113	20
MSM6J2OH	USA	Female	21	HMP2	UC	20,3	#N/D	0	#N/D	#N/D	#N/D	#N/D	26	M2026	Chemagic	1,419984502	17
MSM6J2OJ	USA	Female	21	HMP2	UC	20,3	#N/D	0	#N/D	#N/D	#N/D	#N/D	29	M2026	Chemagic	1,279390662	43
MSM6J2OL	USA	Female	21	HMP2	UC	20,3	#N/D	0	#N/D	#N/D	#N/D	#N/D	31	M2026	Chemagic	1,109261407	24

SampleID	country	gender	Age	study	disease	BMI	hbi	cai	cd localization	cd behaviour	uc extent	dysbiosisindex	Time point	patientID	extraction protocol	shannon	chao
MSM6J2ON	USA	Female	21	HMP2	UC	20,3	#N/D	0	#N/D	#N/D	#N/D	#N/D	33	M2026	Chemagic	1,083380761	22
MSM6J2OP	USA	Female	21	HMP2	UC	20,3	#N/D	0	#N/D	#N/D	#N/D	#N/D	40	M2026	Chemagic	1,135461261	23
MSM79HEA	USA	Female	21	HMP2	UC	20,3	#N/D	0	#N/D	#N/D	#N/D	#N/D	45	M2026	Chemagic	1,322273546	24
MSM79HF1_P	USA	Male	74	HMP2	UC	27,7	#N/D	4	#N/D	#N/D	#N/D	#N/D	0	M2064	Chemagic	2,502796324	55
MSM79HF3	USA	Male	74	HMP2	UC	27,7	#N/D	1	#N/D	#N/D	#N/D	#N/D	2	M2064	Chemagic	2,913746028	52
MSM79HF5	USA	Male	74	HMP2	UC	27,7	#N/D	3	#N/D	#N/D	#N/D	#N/D	4	M2064	Chemagic	2,953209113	58
MSM79HF9	USA	Male	74	HMP2	UC	27,7	#N/D	2	#N/D	#N/D	#N/D	#N/D	8	M2064	Chemagic	3,037544607	53
MSM79HF9_TR	USA	Male	74	HMP2	UC	27,7	#N/D	2	#N/D	#N/D	#N/D	#N/D	8	M2064	Chemagic	2,967285331	52
MSM79HAJ	USA	Male	74	HMP2	UC	27,7	#N/D	2	#N/D	#N/D	#N/D	#N/D	11	M2064	Chemagic	3,137117419	49
MSM79HAL	USA	Male	74	HMP2	UC	27,7	#N/D	3	#N/D	#N/D	#N/D	#N/D	14	M2064	Chemagic	2,823270809	53
MSM79HAN	USA	Male	74	HMP2	UC	27,7	#N/D	5	#N/D	#N/D	#N/D	#N/D	16	M2064	Chemagic	3,209334048	54
MSM79HAT	USA	Male	74	HMP2	UC	27,7	#N/D	3	#N/D	#N/D	#N/D	#N/D	20	M2064	Chemagic	3,032819877	47
MSM79HAR	USA	Male	74	HMP2	UC	27,7	#N/D	4	#N/D	#N/D	#N/D	#N/D	22	M2064	Chemagic	3,042188149	54
MSM9VZLL	USA	Male	74	HMP2	UC	27,7	#N/D	4	#N/D	#N/D	#N/D	#N/D	24	M2064	Chemagic	2,62836058	53
MSM9VZLN	USA	Male	74	HMP2	UC	27,7	#N/D	2	#N/D	#N/D	#N/D	#N/D	28	M2064	Chemagic	2,862310764	55

SampleID	country	gender	Age	study	disease	BMI	hbi	cai	cd localization	cd behaviour	uc extent	dysbiosisindex	Time point	patientID	extraction protocol	shannon	chao
MSM9VZLP	USA	Male	74	HMP2	UC	27,7	#N/D	3	#N/D	#N/D	#N/D	#N/D	28	M2064	Chemagic	2,943558586	51
MSM9VZLR	USA	Male	74	HMP2	UC	27,7	#N/D	5	#N/D	#N/D	#N/D	#N/D	30	M2064	Chemagic	3,030351349	54
MSM9VZLV	USA	Male	74	HMP2	UC	27,7	#N/D	4	#N/D	#N/D	#N/D	#N/D	33	M2064	Chemagic	2,641458329	60
MSM9VZLT	USA	Male	74	HMP2	UC	27,7	#N/D	5	#N/D	#N/D	#N/D	#N/D	37	M2064	Chemagic	3,242977513	54
MSMA26ER	USA	Male	74	HMP2	UC	27,7	#N/D	4	#N/D	#N/D	#N/D	#N/D	41	M2064	Chemagic	2,922060697	40
MSMA26ET	USA	Male	74	HMP2	UC	27,7	#N/D	2	#N/D	#N/D	#N/D	#N/D	50	M2064	Chemagic	2,121009891	45
MSMA26EZ	USA	Male	74	HMP2	UC	27,7	#N/D	3	#N/D	#N/D	#N/D	#N/D	50	M2064	Chemagic	2,386113672	42
MSM79HBZ	USA	Female	29	HMP2	UC	19,1	#N/D	8	#N/D	#N/D	#N/D	#N/D	0	M2069	Chemagic	1,337387369	5
MSM79HD8_P	USA	Female	29	HMP2	UC	19,1	#N/D	1	#N/D	#N/D	#N/D	#N/D	2	M2069	Chemagic	1,881468997	60
MSM79HDA	USA	Female	29	HMP2	UC	19,1	#N/D	3	#N/D	#N/D	#N/D	#N/D	4	M2069	Chemagic	1,187946703	10
MSM79HDC	USA	Female	29	HMP2	UC	19,1	#N/D	3	#N/D	#N/D	#N/D	#N/D	6	M2069	Chemagic	1,353701612	15
MSM79HDE	USA	Female	29	HMP2	UC	19,1	#N/D	2	#N/D	#N/D	#N/D	#N/D	8	M2069	Chemagic	1,515738884	20
MSM79HDI	USA	Female	29	HMP2	UC	19,1	#N/D	4	#N/D	#N/D	#N/D	#N/D	12	M2069	Chemagic	1,96156214	20
MSM9VZEU	USA	Female	29	HMP2	UC	19,1	#N/D	4	#N/D	#N/D	#N/D	#N/D	14	M2069	Chemagic	2,011195294	21
MSM9VZEW	USA	Female	29	HMP2	UC	19,1	#N/D	2	#N/D	#N/D	#N/D	#N/D	16	M2069	Chemagic	1,667502285	20

SampleID	country	gender	Age	study	disease	BMI	hbi	cai	cd localization	cd behaviour	uc extent	dysbiosisindex	Time point	patientID	extraction protocol	shannon	chao
MSM9VZEY	USA	Female	29	HMP2	UC	19,1	#N/D	2	#N/D	#N/D	#N/D	#N/D	18	M2069	Chemagic	1,793742841	22
MSM9VZF1	USA	Female	29	HMP2	UC	19,1	#N/D	3	#N/D	#N/D	#N/D	#N/D	20	M2069	Chemagic	1,817482072	20
MSM9VZF3	USA	Female	29	HMP2	UC	19,1	#N/D	7	#N/D	#N/D	#N/D	#N/D	22	M2069	Chemagic	1,94414306	24
MSM9VZF5	USA	Female	29	HMP2	UC	19,1	#N/D	4	#N/D	#N/D	#N/D	#N/D	24	M2069	Chemagic	1,88087661	23
MSM9VZOY	USA	Female	29	HMP2	UC	19,1	#N/D	3	#N/D	#N/D	#N/D	#N/D	26	M2069	Chemagic	1,884023961	19
MSM9VZOU	USA	Female	29	HMP2	UC	19,1	#N/D	4	#N/D	#N/D	#N/D	#N/D	28	M2069	Chemagic	1,321801562	16
MSM9VZOW	USA	Female	29	HMP2	UC	19,1	#N/D	4	#N/D	#N/D	#N/D	#N/D	30	M2069	Chemagic	1,17838642	16
MSM9VZOS	USA	Female	29	HMP2	UC	19,1	#N/D	5	#N/D	#N/D	#N/D	#N/D	32	M2069	Chemagic	1,339987547	17
MSM9VZP1	USA	Female	29	HMP2	UC	19,1	#N/D	4	#N/D	#N/D	#N/D	#N/D	35	M2069	Chemagic	1,245554866	16
MSM9VZP3	USA	Female	29	HMP2	UC	19,1	#N/D	12	#N/D	#N/D	#N/D	#N/D	36	M2069	Chemagic	1,225556004	14
MSMA26EH	USA	Female	29	HMP2	UC	19,1	#N/D	6	#N/D	#N/D	#N/D	#N/D	39	M2069	Chemagic	1,295572505	5
MSMA26EJ	USA	Female	29	HMP2	UC	19,1	#N/D	5	#N/D	#N/D	#N/D	#N/D	41	M2069	Chemagic	1,664866848	14
MSMA26EL	USA	Female	29	HMP2	UC	19,1	#N/D	6	#N/D	#N/D	#N/D	#N/D	43	M2069	Chemagic	1,328829915	25
MSMA26EN	USA	Female	29	HMP2	UC	19,1	#N/D	6	#N/D	#N/D	#N/D	#N/D	45	M2069	Chemagic	1,970070928	24
MSMA26EP	USA	Female	29	HMP2	UC	19,1	#N/D	9	#N/D	#N/D	#N/D	#N/D	46	M2069	Chemagic	0,488128022	2

SampleID	country	gender	Age	study	disease	BMI	hbi	cai	cd localization	cd behaviour	uc extent	dysbiosisindex	Time point	patientID	extraction protocol	shannon	chao
PSM6XBSE_P	USA	Female	16	HMP2	UC	21,1	#N/D	6	#N/D	#N/D	#N/D	#N/D	0	P6012	Chemagic	2,277635708	39
PSM6XBSE	USA	Female	16	HMP2	UC	21,1	#N/D	6	#N/D	#N/D	#N/D	#N/D	0	P6012	Chemagic	2,127605392	32
PSM6XBVI	USA	Female	16	HMP2	UC	21,1	#N/D	4	#N/D	#N/D	#N/D	#N/D	2	P6012	Chemagic	2,123777331	35
PSM6XBVK	USA	Female	16	HMP2	UC	21,1	#N/D	6	#N/D	#N/D	#N/D	#N/D	4	P6012	Chemagic	1,35258267	27
PSM6XBVM	USA	Female	16	HMP2	UC	21,1	#N/D	5	#N/D	#N/D	#N/D	#N/D	6	P6012	Chemagic	2,041362663	39
PSM6XBVO	USA	Female	16	HMP2	UC	21,1	#N/D	4	#N/D	#N/D	#N/D	#N/D	8	P6012	Chemagic	2,052826571	35
PSM6XBVQ	USA	Female	16	HMP2	UC	21,1	#N/D	4	#N/D	#N/D	#N/D	#N/D	10	P6012	Chemagic	1,834991082	37
PSM6XBVS	USA	Female	16	HMP2	UC	21,1	#N/D	3	#N/D	#N/D	#N/D	#N/D	12	P6012	Chemagic	1,908200471	33
PSM7J1B7	USA	Female	16	HMP2	UC	21,1	#N/D	4	#N/D	#N/D	#N/D	#N/D	15	P6012	Chemagic	2,003842984	38
PSM7J1B9	USA	Female	16	HMP2	UC	21,1	#N/D	5	#N/D	#N/D	#N/D	#N/D	17	P6012	Chemagic	2,066418664	38
PSM7J1BB	USA	Female	16	HMP2	UC	21,1	#N/D	4	#N/D	#N/D	#N/D	#N/D	19	P6012	Chemagic	2,237552392	40
PSM7J1BD	USA	Female	16	HMP2	UC	21,1	#N/D	7	#N/D	#N/D	#N/D	#N/D	23	P6012	Chemagic	2,405601847	35
PSM7J1BF	USA	Female	16	HMP2	UC	21,1	#N/D	7	#N/D	#N/D	#N/D	#N/D	23	P6012	Chemagic	2,514282039	39
PSM7J18Q	USA	Female	16	HMP2	UC	21,1	#N/D	3	#N/D	#N/D	#N/D	#N/D	35	P6012	Chemagic	2,263858166	38
PSM6XBSG_P	USA	Male	6	HMP2	UC	13,5	#N/D	2	#N/D	#N/D	#N/D	#N/D	0	P6013	Chemagic	2,335214863	39

SampleID	country	gender	Age	study	disease	BMI	hbi	cai	cd localization	cd behaviour	uc extent	dysbiosisindex	Time point	patientID	extraction protocol	shannon	chao
PSM6XBT3	USA	Male	6	HMP2	UC	13,5	#N/D	3	#N/D	#N/D	#N/D	#N/D	2	P6013	Chemagic	1,973614103	24
PSM6XBT5	USA	Male	6	HMP2	UC	13,5	#N/D	3	#N/D	#N/D	#N/D	#N/D	4	P6013	Chemagic	1,554442882	21
PSM6XBT7	USA	Male	6	HMP2	UC	13,5	#N/D	3	#N/D	#N/D	#N/D	#N/D	6	P6013	Chemagic	2,095450525	27
PSM6XBT9	USA	Male	6	HMP2	UC	13,5	#N/D	2	#N/D	#N/D	#N/D	#N/D	8	P6013	Chemagic	1,959082775	19
PSM6XBTB	USA	Male	6	HMP2	UC	13,5	#N/D	3	#N/D	#N/D	#N/D	#N/D	10	P6013	Chemagic	1,817652303	33
PSM7J1AM	USA	Male	6	HMP2	UC	13,5	#N/D	3	#N/D	#N/D	#N/D	#N/D	14	P6013	Chemagic	2,091110958	27
PSM7J1AS	USA	Male	6	HMP2	UC	13,5	#N/D	3	#N/D	#N/D	#N/D	#N/D	16	P6013	Chemagic	1,94853297	30
PSM7J1AU	USA	Male	6	HMP2	UC	13,5	#N/D	3	#N/D	#N/D	#N/D	#N/D	18	P6013	Chemagic	1,709170664	25
PSM7J1AO	USA	Male	6	HMP2	UC	13,5	#N/D	3	#N/D	#N/D	#N/D	#N/D	20	P6013	Chemagic	1,809776859	25
PSM7J1AQ	USA	Male	6	HMP2	UC	13,5	#N/D	2	#N/D	#N/D	#N/D	#N/D	22	P6013	Chemagic	1,796568513	25
PSM7J12B	USA	Male	6	HMP2	UC	13,5	#N/D	2	#N/D	#N/D	#N/D	#N/D	33	P6013	Chemagic	1,785525422	25
PSM7J12D	USA	Male	6	HMP2	UC	13,5	#N/D	2	#N/D	#N/D	#N/D	#N/D	35	P6013	Chemagic	1,810604264	27
PSM7J12F	USA	Male	6	HMP2	UC	13,5	#N/D	3	#N/D	#N/D	#N/D	#N/D	36	P6013	Chemagic	1,630818323	29
PSM7J169	USA	Male	6	HMP2	UC	13,5	#N/D	3	#N/D	#N/D	#N/D	#N/D	38	P6013	Chemagic	2,074387789	24
PSM7J16F	USA	Male	6	HMP2	UC	13,5	#N/D	2	#N/D	#N/D	#N/D	#N/D	40	P6013	Chemagic	1,906690634	27

SampleID	country	gender	Age	study	disease	BMI	hbi	cai	cd localization	cd behaviour	uc extent	dysbiosisindex	Time point	patientID	extraction protocol	shannon	chao
PSM7J16B	USA	Male	6	HMP2	UC	13,5	#N/D	6	#N/D	#N/D	#N/D	#N/D	44	P6013	Chemagic	0,990636029	8
PSM7J16H	USA	Male	6	HMP2	UC	13,5	#N/D	6	#N/D	#N/D	#N/D	#N/D	46	P6013	Chemagic	0	1
PSM7J1BV	USA	Male	17	HMP2	UC	19,6	#N/D	1	#N/D	#N/D	#N/D	#N/D	2	P6025	Chemagic	2,257837341	44
PSM7J1BX	USA	Male	17	HMP2	UC	19,6	#N/D	0	#N/D	#N/D	#N/D	#N/D	4	P6025	Chemagic	2,276188871	42
PSM7J1C2_P	USA	Male	17	HMP2	UC	19,6	#N/D	0	#N/D	#N/D	#N/D	#N/D	8	P6025	Chemagic	1,467921105	10
PSM7J1C4	USA	Male	17	HMP2	UC	19,6	#N/D	2	#N/D	#N/D	#N/D	#N/D	10	P6025	Chemagic	2,685159724	52
PSM7J12V	USA	Male	17	HMP2	UC	19,6	#N/D	0	#N/D	#N/D	#N/D	#N/D	16	P6025	Chemagic	2,704312028	47
PSM7J12Z	USA	Male	17	HMP2	UC	19,6	#N/D	0	#N/D	#N/D	#N/D	#N/D	23	P6025	Chemagic	2,866358458	48
PSMA264K	USA	Male	17	HMP2	UC	19,6	#N/D	2	#N/D	#N/D	#N/D	#N/D	38	P6025	Chemagic	1,669951643	28
V1.UC6.0	Spain	Female	38	Meta Hit	Healthy	23,18	#N/D	#N/D	#N/D	#N/D	#N/D	#N/D	0	6HR	Godon	3,263142935	53
V1.UC7.0	Spain	Female	19	Meta Hit	Healthy	23,05	#N/D	#N/D	#N/D	#N/D	#N/D	#N/D	0	7HR	Godon	1,735960342	39
V1.UC8.0	Spain	Male	22	Meta Hit	Healthy	25,4	#N/D	#N/D	#N/D	#N/D	#N/D	#N/D	0	8HR	Godon	3,046943802	40
V1.UC9.0	Spain	Male	32	Meta Hit	Healthy	30,37	#N/D	#N/D	#N/D	#N/D	#N/D	#N/D	0	9HR	Godon	2,970131546	49
V1.UC16.0	Spain	Male	25	Meta Hit	Healthy	23,88	#N/D	#N/D	#N/D	#N/D	#N/D	#N/D	0	16HR	Godon	2,12101997	30
V1.UC18.0	Spain	Female	63	Meta Hit	Healthy	28,67	#N/D	#N/D	#N/D	#N/D	#N/D	#N/D	0	18HR	Godon	2,74862683	37

SampleID	country	gender	Age	study	disease	BMI	hbi	cai	cd localization	cd behaviour	uc extent	dysbiosisindex	Time point	patientID	extraction protocol	shannon	chao
V1.UC19.0	Spain	Female	37	MetaHit	Healthy	21,19	#N/D	#N/D	#N/D	#N/D	#N/D	#N/D	0	19HR	Godon	2,96159651	46
V1.UC24.0	Spain	Female	62	MetaHit	Healthy	33,33	#N/D	#N/D	#N/D	#N/D	#N/D	#N/D	0	24HR	Godon	3,163260646	65
V1.UC27.0	Spain	Male	66	MetaHit	Healthy	26,67	#N/D	#N/D	#N/D	#N/D	#N/D	#N/D	0	27HR	Godon	3,29203197	61
V1.UC28.0	Spain	Female	61	MetaHit	Healthy	24,56	#N/D	#N/D	#N/D	#N/D	#N/D	#N/D	0	28HR	Godon	2,550661388	58
V1.UC29.0	Spain	Male	46	MetaHit	Healthy	24,38	#N/D	#N/D	#N/D	#N/D	#N/D	#N/D	0	29HR	Godon	2,595050664	54
V1.UC30.0	Spain	Female	41	MetaHit	Healthy	23,26	#N/D	#N/D	#N/D	#N/D	#N/D	#N/D	0	30HR	Godon	2,597213849	56
V1.UC32.0	Spain	Male	56	MetaHit	Healthy	25,71	#N/D	#N/D	#N/D	#N/D	#N/D	#N/D	0	32HR	Godon	2,921958213	56
V1.UC33.0	Spain	Female	53	MetaHit	Healthy	26,53	#N/D	#N/D	#N/D	#N/D	#N/D	#N/D	0	33HR	Godon	3,180433252	62
V1.UC34.0	Spain	Male	31	MetaHit	Healthy	23,05	#N/D	#N/D	#N/D	#N/D	#N/D	#N/D	0	34HR	Godon	3,033820907	49
V1.UC36.0	Spain	Female	34	MetaHit	Healthy	20,43	#N/D	#N/D	#N/D	#N/D	#N/D	#N/D	0	36HR	Godon	2,988074535	67
V1.UC37.0	Spain	Male	19	MetaHit	Healthy	23,99	#N/D	#N/D	#N/D	#N/D	#N/D	#N/D	0	37HR	Godon	3,367804783	53
V1.UC41.0	Spain	Female	54	MetaHit	Healthy	23,92	#N/D	#N/D	#N/D	#N/D	#N/D	#N/D	0	41HR	Godon	2,483574893	44
V1.UC42.0	Spain	Female	32	MetaHit	Healthy	19,56	#N/D	#N/D	#N/D	#N/D	#N/D	#N/D	0	42HR	Godon	3,172396901	57
V1.UC43.0	Spain	Female	27	MetaHit	Healthy	17,71	#N/D	#N/D	#N/D	#N/D	#N/D	#N/D	0	43HR	Godon	3,290774725	63
V1.UC44.0	Spain	Male	20	MetaHit	Healthy	20,29	#N/D	#N/D	#N/D	#N/D	#N/D	#N/D	0	44HR	Godon	3,066570363	63

SampleID	country	gender	Age	study	disease	BMI	hbi	cai	cd localization	cd behaviour	uc extent	dysbiosisindex	Time point	patientID	extraction protocol	shannon	chao
V1.UC46.0	Spain	Female	55	Meta Hit	Healthy	35,03	#N/D	#N/D	#N/D	#N/D	#N/D	#N/D	0	46HR	Godon	3,191030991	69
V1.UC48.0	Spain	Male	26	Meta Hit	Healthy	20,9	#N/D	#N/D	#N/D	#N/D	#N/D	#N/D	0	48HR	Godon	3,574320519	77
V1.UC57.0	Spain	Female	32	Meta Hit	Healthy	19,98	#N/D	#N/D	#N/D	#N/D	#N/D	#N/D	0	57HR	Godon	3,391965721	65
V1.UC60.0	Spain	Female	25	Meta Hit	Healthy	24,17	#N/D	#N/D	#N/D	#N/D	#N/D	#N/D	0	60HR	Godon	3,078541707	57
V1.UC61.0	Spain	Male	33	Meta Hit	Healthy	27,78	#N/D	#N/D	#N/D	#N/D	#N/D	#N/D	0	61HR	Godon	2,465861174	55
V1.UC62.0	Spain	Female	37	Meta Hit	Healthy	22,58	#N/D	#N/D	#N/D	#N/D	#N/D	#N/D	0	62HR	Godon	3,099274776	62
V1.UC63.0	Spain	Male	45	Meta Hit	Healthy	26,3	#N/D	#N/D	#N/D	#N/D	#N/D	#N/D	0	63HR	Godon	3,356429193	61
V1.UC64.0	Spain	Female	41	Meta Hit	Healthy	25,61	#N/D	#N/D	#N/D	#N/D	#N/D	#N/D	0	64HR	Godon	3,204374279	61
V1.UC22.1	Spain	Male	37	Meta Hit	Healthy	22,53	#N/D	#N/D	#N/D	#N/D	#N/D	#N/D	1	22HR	Godon	3,268245369	64
V1.CD2.0.PN	Spain	Female	22	Meta Hit	Healthy	23,92	#N/D	#N/D	#N/D	#N/D	#N/D	#N/D	0	2PNHealthy	Godon	2,663818703	34
V1.CD4.0.PN	Spain	Female	60	Meta Hit	Healthy	19,98	#N/D	#N/D	#N/D	#N/D	#N/D	#N/D	0	4PNHealthy	Godon	2,714805808	57
V1.CD6.0.PN	Spain	Male	69	Meta Hit	Healthy	28,09	#N/D	#N/D	#N/D	#N/D	#N/D	#N/D	0	6PNHealthy	Godon	2,952982744	60
V1.CD8.0.PN	Spain	Male	20	Meta Hit	Healthy	21,55	#N/D	#N/D	#N/D	#N/D	#N/D	#N/D	0	8PNHealthy	Godon	3,085867473	63,5
V1.CD6.0.PT	Spain	Female	51	Meta Hit	Healthy	23,03	#N/D	#N/D	#N/D	#N/D	#N/D	#N/D	0	6PTHealthy	Godon	3,123338397	60
V1.CD2.0	Spain	Male	49	Meta Hit	Healthy	27,76	#N/D	#N/D	#N/D	#N/D	#N/D	#N/D	0	2Healthy	Godon	1,126718455	29

SampleID	country	gender	Age	study	disease	BMI	hbi	cai	cd localization	cd behaviour	uc extent	dysbiosisindex	Time point	patientID	extraction protocol	shannon	chao
V1.CD3.0	Spain	Female	18	MetaHit	Healthy	21,51	#N/D	#N/D	#N/D	#N/D	#N/D	#N/D	0	3Healthy	Godon	2,70288975	40
V1.CD4.0	Spain	Female	46	MetaHit	Healthy	29,69	#N/D	#N/D	#N/D	#N/D	#N/D	#N/D	0	4Healthy	Godon	2,868753498	43
V1.CD8.0	Spain	Male	51	MetaHit	Healthy	29,38	#N/D	#N/D	#N/D	#N/D	#N/D	#N/D	0	8Healthy	Godon	2,724112839	35
V1.CD9.0	Spain	Female	48	MetaHit	Healthy	27,55	#N/D	#N/D	#N/D	#N/D	#N/D	#N/D	0	9Healthy	Godon	2,584287428	37
V1.CD11.0	Spain	Female	62	MetaHit	Healthy	35,46	#N/D	#N/D	#N/D	#N/D	#N/D	#N/D	0	11Healthy	Godon	2,39094708	20
V1.CD13.0	Spain	Male	68	MetaHit	Healthy	25,69	#N/D	#N/D	#N/D	#N/D	#N/D	#N/D	0	13Healthy	Godon	3,102111366	58
V1.CD14.0	Spain	Female	41	MetaHit	Healthy	23,12	#N/D	#N/D	#N/D	#N/D	#N/D	#N/D	0	14Healthy	Godon	3,209290601	55
V1.CD16.0	Spain	Female	68	MetaHit	Healthy	21,61	#N/D	#N/D	#N/D	#N/D	#N/D	#N/D	0	16Healthy	Godon	3,199905229	66
V1.CD19.0	Spain	Male	42	MetaHit	Healthy	25,95	#N/D	#N/D	#N/D	#N/D	#N/D	#N/D	0	19Healthy	Godon	3,152639903	65
V1.CD22.0	Spain	Male	68	MetaHit	Healthy	24,24	#N/D	#N/D	#N/D	#N/D	#N/D	#N/D	0	22Healthy	Godon	3,420446757	67
V1.CD24.0	Spain	Female	41	MetaHit	Healthy	20,58	#N/D	#N/D	#N/D	#N/D	#N/D	#N/D	0	24Healthy	Godon	3,318386607	63
V1.CD27.0	Spain	Male	36	MetaHit	Healthy	24,81	#N/D	#N/D	#N/D	#N/D	#N/D	#N/D	0	27Healthy	Godon	3,050270556	55
V1.CD28.0	Spain	Male	24	MetaHit	Healthy	21,46	#N/D	#N/D	#N/D	#N/D	#N/D	#N/D	0	28Healthy	Godon	1,79416156	48
V1.CD29.0	Spain	Male	60	MetaHit	Healthy	31,25	#N/D	#N/D	#N/D	#N/D	#N/D	#N/D	0	29Healthy	Godon	3,05735297	68
V1.CD30.0	Spain	Female	59	MetaHit	Healthy	19,82	#N/D	#N/D	#N/D	#N/D	#N/D	#N/D	0	30Healthy	Godon	3,535785994	71

SampleID	country	gender	Age	study	disease	BMI	hbi	cai	cd localization	cd behaviour	uc extent	dysbiosisindex	Time point	patientID	extraction protocol	shannon	chao
V1.CD31.0	Spain	Female	28	MetaHit	Healthy	22,6	#N/D	#N/D	#N/D	#N/D	#N/D	#N/D	0	31Healthy	Godon	3,048358644	66
V1.CD34.0	Spain	Female	54	MetaHit	Healthy	23,61	#N/D	#N/D	#N/D	#N/D	#N/D	#N/D	0	34Healthy	Godon	2,012032474	53
V1.CD36.0	Spain	Male	20	MetaHit	Healthy	22,04	#N/D	#N/D	#N/D	#N/D	#N/D	#N/D	0	36Healthy	Godon	2,856757928	46
V1.CD38.0	Spain	Female	63	MetaHit	Healthy	26,84	#N/D	#N/D	#N/D	#N/D	#N/D	#N/D	0	38Healthy	Godon	2,830511334	56
V1.CD40.0	Spain	Female	46	MetaHit	Healthy	23,73	#N/D	#N/D	#N/D	#N/D	#N/D	#N/D	0	40Healthy	Godon	2,270039881	66
V1.CD42.0	Spain	Male	48	MetaHit	Healthy	24,82	#N/D	#N/D	#N/D	#N/D	#N/D	#N/D	0	42Healthy	Godon	2,021513743	46
V1.CD43.0	Spain	Female	45	MetaHit	Healthy	19,53	#N/D	#N/D	#N/D	#N/D	#N/D	#N/D	0	43Healthy	Godon	2,871823419	61
V1.CD45.0	Spain	Male	18	MetaHit	Healthy	29,6	#N/D	#N/D	#N/D	#N/D	#N/D	#N/D	0	45Healthy	Godon	1,890214217	61
V1.CD49.0	Spain	Female	52	MetaHit	Healthy	39,96	#N/D	#N/D	#N/D	#N/D	#N/D	#N/D	0	49Healthy	Godon	3,295866027	61
V1.CD50.0	Spain	Female	18	MetaHit	Healthy	21,48	#N/D	#N/D	#N/D	#N/D	#N/D	#N/D	0	50Healthy	Godon	3,147817407	63
V1.CD52.0	Spain	Male	55	MetaHit	Healthy	32,49	#N/D	#N/D	#N/D	#N/D	#N/D	#N/D	0	52Healthy	Godon	3,30981305	68
V1.CD55.0	Spain	Female	59	MetaHit	Healthy	32,46	#N/D	#N/D	#N/D	#N/D	#N/D	#N/D	0	55Healthy	Godon	3,206507515	71,5
V1.CD4.0.PT	Spain	Male	61	MetaHit	Healthy	22,99	#N/D	#N/D	#N/D	#N/D	#N/D	#N/D	0	4PTHealthy	Godon	0,571058518	20
V1.CD8.0.PT	Spain	Female	62	MetaHit	Healthy	28,4	#N/D	#N/D	#N/D	#N/D	#N/D	#N/D	0	8PTHealthy	Godon	3,207133704	64
V1.CD23.0	Spain	Female	66	MetaHit	Healthy	24,39	#N/D	#N/D	#N/D	#N/D	#N/D	#N/D	0	23Healthy	Godon	3,090641041	66

SampleID	country	gender	Age	study	disease	BMI	hbi	cai	cd localization	cd behaviour	uc extent	dysbiosisindex	Time point	patientID	extraction protocol	shannon	chao
V1.CD33.0	Spain	Male	26	Meta Hit	Healthy	23,78	#N/D	#N/D	#N/D	#N/D	#N/D	#N/D	0	33Healthy	Godon	2,720716478	65
HSM5MD8A_P	USA	Female	13	HMP2	Healthy	35,6	#N/D	#N/D	#N/D	#N/D	#N/D	#N/D	0	H4008	Chemagic	2,89528403	53
HSM5MD57_P	USA	Female	13	HMP2	Healthy	35,6	#N/D	#N/D	#N/D	#N/D	#N/D	#N/D	2	H4008	Chemagic	2,689600835	56
HSM5MD59_P	USA	Female	13	HMP2	Healthy	35,6	#N/D	#N/D	#N/D	#N/D	#N/D	#N/D	4	H4008	Chemagic	2,477425403	55
HSM5MD5B_P	USA	Female	13	HMP2	Healthy	35,6	#N/D	#N/D	#N/D	#N/D	#N/D	#N/D	6	H4008	Chemagic	2,461900868	55
HSM5MD5B	USA	Female	13	HMP2	Healthy	35,6	#N/D	#N/D	#N/D	#N/D	#N/D	#N/D	6	H4008	Chemagic	2,903412422	55
HSM5MD5D_P	USA	Female	13	HMP2	Healthy	35,6	#N/D	#N/D	#N/D	#N/D	#N/D	#N/D	8	H4008	Chemagic	2,682011869	52
HSM5MD5D	USA	Female	13	HMP2	Healthy	35,6	#N/D	#N/D	#N/D	#N/D	#N/D	#N/D	8	H4008	Chemagic	2,760354911	51
HSM5MD5F_P	USA	Female	13	HMP2	Healthy	35,6	#N/D	#N/D	#N/D	#N/D	#N/D	#N/D	10	H4008	Chemagic	2,904481691	61
HSM6XRSX_P	USA	Female	13	HMP2	Healthy	35,6	#N/D	#N/D	#N/D	#N/D	#N/D	#N/D	11	H4008	Chemagic	2,767871108	57
HSM6XRSX	USA	Female	13	HMP2	Healthy	35,6	#N/D	#N/D	#N/D	#N/D	#N/D	#N/D	11	H4008	Chemagic	2,801852503	55
HSM6XRT6_P	USA	Female	13	HMP2	Healthy	35,6	#N/D	#N/D	#N/D	#N/D	#N/D	#N/D	14	H4008	Chemagic	3,097899305	55
HSM6XRSZ_P	USA	Female	13	HMP2	Healthy	35,6	#N/D	#N/D	#N/D	#N/D	#N/D	#N/D	16	H4008	Chemagic	2,450693828	51
HSM6XRT4_P	USA	Female	13	HMP2	Healthy	35,6	#N/D	#N/D	#N/D	#N/D	#N/D	#N/D	18	H4008	Chemagic	2,444597344	53
HSM6XRT2_P	USA	Female	13	HMP2	Healthy	35,6	#N/D	#N/D	#N/D	#N/D	#N/D	#N/D	20	H4008	Chemagic	2,451738267	55

SampleID	country	gender	Age	study	disease	BMI	hbi	cai	cd localization	cd behaviour	uc extent	dysbiosisindex	Time point	patientID	extraction protocol	shannon	chao
HSM67VFX_P	USA	Female	13	HMP2	Healthy	35,6	#N/D	#N/D	#N/D	#N/D	#N/D	#N/D	24	H4008	Chemagic	2,502814249	50
HSM67VFX	USA	Female	13	HMP2	Healthy	35,6	#N/D	#N/D	#N/D	#N/D	#N/D	#N/D	24	H4008	Chemagic	2,563807764	49
HSM67VFZ	USA	Female	13	HMP2	Healthy	35,6	#N/D	#N/D	#N/D	#N/D	#N/D	#N/D	26	H4008	Chemagic	2,544312251	53,5
HSM67VG2_P	USA	Female	13	HMP2	Healthy	35,6	#N/D	#N/D	#N/D	#N/D	#N/D	#N/D	28	H4008	Chemagic	1,640557831	45
HSM67VG6_P	USA	Female	13	HMP2	Healthy	35,6	#N/D	#N/D	#N/D	#N/D	#N/D	#N/D	32	H4008	Chemagic	2,971749731	55
HSM67VG8	USA	Female	13	HMP2	Healthy	35,6	#N/D	#N/D	#N/D	#N/D	#N/D	#N/D	34	H4008	Chemagic	2,355474732	51
CSM7CZ2F_P	USA	Female	13	HMP2	Healthy	35,6	#N/D	#N/D	#N/D	#N/D	#N/D	#N/D	36	H4008	Chemagic	2,519846813	37
HSM7CZ2H	USA	Female	13	HMP2	Healthy	35,6	#N/D	#N/D	#N/D	#N/D	#N/D	#N/D	38	H4008	Chemagic	2,563661862	47
HSM7CZ2J_P	USA	Female	13	HMP2	Healthy	35,6	#N/D	#N/D	#N/D	#N/D	#N/D	#N/D	40	H4008	Chemagic	2,418460684	39
HSM7CZ2L_P	USA	Female	13	HMP2	Healthy	35,6	#N/D	#N/D	#N/D	#N/D	#N/D	#N/D	42	H4008	Chemagic	2,421059195	48
HSM5MD82_P	USA	Female	6	HMP2	Healthy	14	#N/D	#N/D	#N/D	#N/D	#N/D	#N/D	0	H4009	Chemagic	2,433990431	51
HSM5MD8P_P	USA	Female	6	HMP2	Healthy	14	#N/D	#N/D	#N/D	#N/D	#N/D	#N/D	1	H4009	Chemagic	1,390164086	42
HSM5MD8N_P	USA	Female	6	HMP2	Healthy	14	#N/D	#N/D	#N/D	#N/D	#N/D	#N/D	3	H4009	Chemagic	2,581148038	47
HSM5MD8L_P	USA	Female	6	HMP2	Healthy	14	#N/D	#N/D	#N/D	#N/D	#N/D	#N/D	5	H4009	Chemagic	1,23336772	38
HSM5MD8B_P	USA	Female	6	HMP2	Healthy	14	#N/D	#N/D	#N/D	#N/D	#N/D	#N/D	7	H4009	Chemagic	1,854966618	50

SampleID	country	gender	Age	study	disease	BMI	hbi	cai	cd localization	cd behaviour	uc extent	dysbiosisindex	Time point	patientID	extraction protocol	shannon	chao
HSM5MD8D_P	USA	Female	6	HMP2	Healthy	14	#N/D	#N/D	#N/D	#N/D	#N/D	#N/D	9	H4009	Chemagic	1,277558467	37
HSM6XRT8	USA	Female	6	HMP2	Healthy	14	#N/D	#N/D	#N/D	#N/D	#N/D	#N/D	11	H4009	Chemagic	2,66903332	45
HSM6XRTA_P	USA	Female	6	HMP2	Healthy	14	#N/D	#N/D	#N/D	#N/D	#N/D	#N/D	13	H4009	Chemagic	2,758623705	48
HSM6XRTC_P	USA	Female	6	HMP2	Healthy	14	#N/D	#N/D	#N/D	#N/D	#N/D	#N/D	15	H4009	Chemagic	1,957065157	39
HSM6XRTE_P	USA	Female	6	HMP2	Healthy	14	#N/D	#N/D	#N/D	#N/D	#N/D	#N/D	17	H4009	Chemagic	2,883043028	57
HSM6XRTG_P	USA	Female	6	HMP2	Healthy	14	#N/D	#N/D	#N/D	#N/D	#N/D	#N/D	19	H4009	Chemagic	1,334091033	42
HSM6XRTG	USA	Female	6	HMP2	Healthy	14	#N/D	#N/D	#N/D	#N/D	#N/D	#N/D	19	H4009	Chemagic	1,334456168	40
HSM67VGA_P	USA	Female	6	HMP2	Healthy	14	#N/D	#N/D	#N/D	#N/D	#N/D	#N/D	23	H4009	Chemagic	2,655410572	47
HSM67VGA	USA	Female	6	HMP2	Healthy	14	#N/D	#N/D	#N/D	#N/D	#N/D	#N/D	23	H4009	Chemagic	2,823966118	51
HSM67VGC	USA	Female	6	HMP2	Healthy	14	#N/D	#N/D	#N/D	#N/D	#N/D	#N/D	27	H4009	Chemagic	2,542013384	44
HSM67VGE_P	USA	Female	6	HMP2	Healthy	14	#N/D	#N/D	#N/D	#N/D	#N/D	#N/D	29	H4009	Chemagic	2,571607746	71
HSM67VGG	USA	Female	6	HMP2	Healthy	14	#N/D	#N/D	#N/D	#N/D	#N/D	#N/D	31	H4009	Chemagic	2,524372573	45
HSM67VGI_P	USA	Female	6	HMP2	Healthy	14	#N/D	#N/D	#N/D	#N/D	#N/D	#N/D	33	H4009	Chemagic	2,292457643	31
HSM67VGK	USA	Female	6	HMP2	Healthy	14	#N/D	#N/D	#N/D	#N/D	#N/D	#N/D	35	H4009	Chemagic	1,451651458	41
HSM67VGK_TR	USA	Female	6	HMP2	Healthy	14	#N/D	#N/D	#N/D	#N/D	#N/D	#N/D	35	H4009	Chemagic	1,485526213	42

SampleID	country	gender	Age	study	disease	BMI	hbi	cai	cd localization	cd behaviour	uc extent	dysbiosisindex	Time point	patientID	extraction protocol	shannon	chao
HSM7CYYR_P	USA	Female	6	HMP2	Healthy	14	#N/D	#N/D	#N/D	#N/D	#N/D	#N/D	37	H4009	Chemagic	2,112655746	32
HSM7CYYV_P	USA	Female	6	HMP2	Healthy	14	#N/D	#N/D	#N/D	#N/D	#N/D	#N/D	41	H4009	Chemagic	1,792418555	33
HSM5MD8H_P	USA	Male	8	HMP2	Healthy	16,1	#N/D	#N/D	#N/D	#N/D	#N/D	#N/D	0	H4013	Chemagic	2,369811887	39
HSM5MD6E_P	USA	Male	8	HMP2	Healthy	16,1	#N/D	#N/D	#N/D	#N/D	#N/D	#N/D	2	H4013	Chemagic	1,836092679	41
HSM5MD6I_P	USA	Male	8	HMP2	Healthy	16,1	#N/D	#N/D	#N/D	#N/D	#N/D	#N/D	5	H4013	Chemagic	1,601006401	35
HSM5MD6I	USA	Male	8	HMP2	Healthy	16,1	#N/D	#N/D	#N/D	#N/D	#N/D	#N/D	5	H4013	Chemagic	1,796038688	38
HSM5MD6K_P	USA	Male	8	HMP2	Healthy	16,1	#N/D	#N/D	#N/D	#N/D	#N/D	#N/D	7	H4013	Chemagic	1,785485892	37
HSM5MD6K	USA	Male	8	HMP2	Healthy	16,1	#N/D	#N/D	#N/D	#N/D	#N/D	#N/D	7	H4013	Chemagic	1,863951563	37
HSM5MD6M	USA	Male	8	HMP2	Healthy	16,1	#N/D	#N/D	#N/D	#N/D	#N/D	#N/D	9	H4013	Chemagic	1,496048629	25
HSM6XRRJ	USA	Male	8	HMP2	Healthy	16,1	#N/D	#N/D	#N/D	#N/D	#N/D	#N/D	15	H4013	Chemagic	1,329805334	21
HSM6XRVA	USA	Male	8	HMP2	Healthy	16,1	#N/D	#N/D	#N/D	#N/D	#N/D	#N/D	24	H4013	Chemagic	1,881259187	39
HSM6XRVC	USA	Male	8	HMP2	Healthy	16,1	#N/D	#N/D	#N/D	#N/D	#N/D	#N/D	25	H4013	Chemagic	2,0610954	41
HSM6XRVC_TR	USA	Male	8	HMP2	Healthy	16,1	#N/D	#N/D	#N/D	#N/D	#N/D	#N/D	25	H4013	Chemagic	1,622756069	35
HSM6XRVK	USA	Male	8	HMP2	Healthy	16,1	#N/D	#N/D	#N/D	#N/D	#N/D	#N/D	31	H4013	Chemagic	1,865452614	40
HSM7J4QZ	USA	Male	8	HMP2	Healthy	16,1	#N/D	#N/D	#N/D	#N/D	#N/D	#N/D	36	H4013	Chemagic	2,52812241	42

SampleID	country	gender	Age	study	disease	BMI	hbi	cai	cd localization	cd behaviour	uc extent	dysbiosisindex	Time point	patientID	extraction protocol	shannon	chao
HSM7J4R2	USA	Male	8	HMP2	Healthy	16,1	#N/D	#N/D	#N/D	#N/D	#N/D	#N/D	41	H4013	Chemagic	1,904460149	41
HSM5MD5Z_P	USA	Female	10	HMP2	Healthy	13,5	#N/D	#N/D	#N/D	#N/D	#N/D	#N/D	0	H4016	Chemagic	1,873135808	11
HSM5MD6O_P	USA	Female	10	HMP2	Healthy	13,5	#N/D	#N/D	#N/D	#N/D	#N/D	#N/D	2	H4016	Chemagic	2,112544598	27
HSM5MD6Q	USA	Female	10	HMP2	Healthy	13,5	#N/D	#N/D	#N/D	#N/D	#N/D	#N/D	5	H4016	Chemagic	2,443044598	41
HSM5MD6S_P	USA	Female	10	HMP2	Healthy	13,5	#N/D	#N/D	#N/D	#N/D	#N/D	#N/D	5	H4016	Chemagic	2,28206144	38
HSM5MD6U_P	USA	Female	10	HMP2	Healthy	13,5	#N/D	#N/D	#N/D	#N/D	#N/D	#N/D	8	H4016	Chemagic	2,387659775	56
HSM5MD6W	USA	Female	10	HMP2	Healthy	13,5	#N/D	#N/D	#N/D	#N/D	#N/D	#N/D	10	H4016	Chemagic	1,432664766	26
HSM67VF3	USA	Female	10	HMP2	Healthy	13,5	#N/D	#N/D	#N/D	#N/D	#N/D	#N/D	15	H4016	Chemagic	1,503663286	24
HSM7CYXE	USA	Female	10	HMP2	Healthy	13,5	#N/D	#N/D	#N/D	#N/D	#N/D	#N/D	24	H4016	Chemagic	1,636600264	29,5
HSM7CYXG	USA	Female	10	HMP2	Healthy	13,5	#N/D	#N/D	#N/D	#N/D	#N/D	#N/D	26	H4016	Chemagic	1,850183887	33
HSM7CYXI	USA	Female	10	HMP2	Healthy	13,5	#N/D	#N/D	#N/D	#N/D	#N/D	#N/D	27	H4016	Chemagic	1,678824643	18
HSM7CYXO	USA	Female	10	HMP2	Healthy	13,5	#N/D	#N/D	#N/D	#N/D	#N/D	#N/D	34	H4016	Chemagic	1,530235614	23
HSM7J4I3	USA	Female	10	HMP2	Healthy	13,5	#N/D	#N/D	#N/D	#N/D	#N/D	#N/D	35	H4016	Chemagic	1,834228625	36
HSM7J4KO	USA	Female	10	HMP2	Healthy	13,5	#N/D	#N/D	#N/D	#N/D	#N/D	#N/D	38	H4016	Chemagic	1,816700588	36
HSM7J4KQ	USA	Female	10	HMP2	Healthy	13,5	#N/D	#N/D	#N/D	#N/D	#N/D	#N/D	47	H4016	Chemagic	1,878233615	35

SampleID	country	gender	Age	study	disease	BMI	hbi	cai	cd localization	cd behaviour	uc extent	dysbiosisindex	Time point	patientID	extraction protocol	shannon	chao
HSM6XRQC_P	USA	Female	13	HMP2	Healthy	25,2	#N/D	#N/D	#N/D	#N/D	#N/D	#N/D	0	H4018	Chemagic	1,261316244	17
HSM6XRQS	USA	Female	13	HMP2	Healthy	25,2	#N/D	#N/D	#N/D	#N/D	#N/D	#N/D	4	H4018	Chemagic	1,641609024	21
HSM6XRQU	USA	Female	13	HMP2	Healthy	25,2	#N/D	#N/D	#N/D	#N/D	#N/D	#N/D	6	H4018	Chemagic	1,636016436	26
HSM6XRQW	USA	Female	13	HMP2	Healthy	25,2	#N/D	#N/D	#N/D	#N/D	#N/D	#N/D	8	H4018	Chemagic	1,258389572	19
HSM6XRQY	USA	Female	13	HMP2	Healthy	25,2	#N/D	#N/D	#N/D	#N/D	#N/D	#N/D	10	H4018	Chemagic	1,601460607	27
HSM67VFR	USA	Female	13	HMP2	Healthy	25,2	#N/D	#N/D	#N/D	#N/D	#N/D	#N/D	18	H4018	Chemagic	1,472679597	25
HSM7CYYF	USA	Female	13	HMP2	Healthy	25,2	#N/D	#N/D	#N/D	#N/D	#N/D	#N/D	24	H4018	Chemagic	1,556969536	28
HSM7CYYH	USA	Female	13	HMP2	Healthy	25,2	#N/D	#N/D	#N/D	#N/D	#N/D	#N/D	26	H4018	Chemagic	1,850474007	28
HSM7CYYP	USA	Female	13	HMP2	Healthy	25,2	#N/D	#N/D	#N/D	#N/D	#N/D	#N/D	34	H4018	Chemagic	1,269167286	18
HSM7J4LD	USA	Female	13	HMP2	Healthy	25,2	#N/D	#N/D	#N/D	#N/D	#N/D	#N/D	36	H4018	Chemagic	1,409574221	27
HSM7J4LF	USA	Female	13	HMP2	Healthy	25,2	#N/D	#N/D	#N/D	#N/D	#N/D	#N/D	38	H4018	Chemagic	1,366934115	15
HSM7J4LN	USA	Female	13	HMP2	Healthy	25,2	#N/D	#N/D	#N/D	#N/D	#N/D	#N/D	46	H4018	Chemagic	1,874698164	21
HSM67VDX_P	USA	Female	9	HMP2	Healthy	18,9	#N/D	#N/D	#N/D	#N/D	#N/D	#N/D	0	H4022	Chemagic	2,970393751	55
HSM67VEO	USA	Female	9	HMP2	Healthy	18,9	#N/D	#N/D	#N/D	#N/D	#N/D	#N/D	2	H4022	Chemagic	2,430593821	51
HSM67VEQ	USA	Female	9	HMP2	Healthy	18,9	#N/D	#N/D	#N/D	#N/D	#N/D	#N/D	4	H4022	Chemagic	2,925759752	50

SampleID	country	gender	Age	study	disease	BMI	hbi	cai	cd localization	cd behaviour	uc extent	dysbiosisindex	Time point	patientID	extraction protocol	shannon	chao
HSM67VES	USA	Female	9	HMP2	Healthy	18,9	#N/D	#N/D	#N/D	#N/D	#N/D	#N/D	6	H4022	Chemagic	2,624667415	52
HSM67VEU	USA	Female	9	HMP2	Healthy	18,9	#N/D	#N/D	#N/D	#N/D	#N/D	#N/D	8	H4022	Chemagic	2,686609246	57
HSM67VEW	USA	Female	9	HMP2	Healthy	18,9	#N/D	#N/D	#N/D	#N/D	#N/D	#N/D	10	H4022	Chemagic	2,829141439	52
HSM7CYZJ	USA	Female	9	HMP2	Healthy	18,9	#N/D	#N/D	#N/D	#N/D	#N/D	#N/D	14	H4022	Chemagic	2,915874752	54
HSM7CYZL	USA	Female	9	HMP2	Healthy	18,9	#N/D	#N/D	#N/D	#N/D	#N/D	#N/D	16	H4022	Chemagic	2,868144136	52
HSM7CYZR	USA	Female	9	HMP2	Healthy	18,9	#N/D	#N/D	#N/D	#N/D	#N/D	#N/D	22	H4022	Chemagic	2,662526512	50
HSM7J4RE	USA	Female	9	HMP2	Healthy	18,9	#N/D	#N/D	#N/D	#N/D	#N/D	#N/D	26	H4022	Chemagic	2,672625201	53,33333333
HSM7J4G1	USA	Female	9	HMP2	Healthy	18,9	#N/D	#N/D	#N/D	#N/D	#N/D	#N/D	28	H4022	Chemagic	2,416361117	54
HSM7J4G8	USA	Female	9	HMP2	Healthy	18,9	#N/D	#N/D	#N/D	#N/D	#N/D	#N/D	34	H4022	Chemagic	2,657819911	51
HSM7J4IU	USA	Female	9	HMP2	Healthy	18,9	#N/D	#N/D	#N/D	#N/D	#N/D	#N/D	36	H4022	Chemagic	2,788759139	50
HSM7J4IW	USA	Female	9	HMP2	Healthy	18,9	#N/D	#N/D	#N/D	#N/D	#N/D	#N/D	38	H4022	Chemagic	2,740871592	53
HSM67VDR_P	USA	Male	16	HMP2	Healthy	18,8	#N/D	#N/D	#N/D	#N/D	#N/D	#N/D	0	H4023	Chemagic	2,428792179	48
HSM6XRUL	USA	Male	16	HMP2	Healthy	18,8	#N/D	#N/D	#N/D	#N/D	#N/D	#N/D	1	H4023	Chemagic	2,325517845	43
HSM6XRUN	USA	Male	16	HMP2	Healthy	18,8	#N/D	#N/D	#N/D	#N/D	#N/D	#N/D	3	H4023	Chemagic	2,358821701	37
HSM6XRUR	USA	Male	16	HMP2	Healthy	18,8	#N/D	#N/D	#N/D	#N/D	#N/D	#N/D	7	H4023	Chemagic	0,608875751	40

SampleID	country	gender	Age	study	disease	BMI	hbi	cai	cd localization	cd behaviour	uc extent	dysbiosisindex	Time point	patientID	extraction protocol	shannon	chao
HSM6XRQ8	USA	Male	16	HMP2	Healthy	18,8	#N/D	#N/D	#N/D	#N/D	#N/D	#N/D	9	H4023	Chemagic	1,391614844	44
HSM7CZ16	USA	Male	16	HMP2	Healthy	18,8	#N/D	#N/D	#N/D	#N/D	#N/D	#N/D	11	H4023	Chemagic	1,579355566	49
HSM7CZ18	USA	Male	16	HMP2	Healthy	18,8	#N/D	#N/D	#N/D	#N/D	#N/D	#N/D	13	H4023	Chemagic	2,642178411	54
HSM7CZ1A	USA	Male	16	HMP2	Healthy	18,8	#N/D	#N/D	#N/D	#N/D	#N/D	#N/D	15	H4023	Chemagic	2,689538295	60
HSM7CZ1C	USA	Male	16	HMP2	Healthy	18,8	#N/D	#N/D	#N/D	#N/D	#N/D	#N/D	17	H4023	Chemagic	2,724255134	53
HSM7CZ1E	USA	Male	16	HMP2	Healthy	18,8	#N/D	#N/D	#N/D	#N/D	#N/D	#N/D	19	H4023	Chemagic	0,778370211	42
HSM7CZ1G	USA	Male	16	HMP2	Healthy	18,8	#N/D	#N/D	#N/D	#N/D	#N/D	#N/D	21	H4023	Chemagic	1,448280681	46
HSM7J4HA	USA	Male	16	HMP2	Healthy	18,8	#N/D	#N/D	#N/D	#N/D	#N/D	#N/D	23	H4023	Chemagic	2,233561586	51
HSM7J4HC	USA	Male	16	HMP2	Healthy	18,8	#N/D	#N/D	#N/D	#N/D	#N/D	#N/D	24	H4023	Chemagic	2,420711277	49,5
HSM7J4HE	USA	Male	16	HMP2	Healthy	18,8	#N/D	#N/D	#N/D	#N/D	#N/D	#N/D	25	H4023	Chemagic	1,189173103	43,5
HSM7J4HG	USA	Male	16	HMP2	Healthy	18,8	#N/D	#N/D	#N/D	#N/D	#N/D	#N/D	29	H4023	Chemagic	2,459673923	49
HSM7J4HI	USA	Male	16	HMP2	Healthy	18,8	#N/D	#N/D	#N/D	#N/D	#N/D	#N/D	31	H4023	Chemagic	2,448144738	48
HSM7J4HK	USA	Male	16	HMP2	Healthy	18,8	#N/D	#N/D	#N/D	#N/D	#N/D	#N/D	33	H4023	Chemagic	2,88190356	50
HSM7J4KC	USA	Male	16	HMP2	Healthy	18,8	#N/D	#N/D	#N/D	#N/D	#N/D	#N/D	35	H4023	Chemagic	0,937984699	41
HSM7J4KG	USA	Male	16	HMP2	Healthy	18,8	#N/D	#N/D	#N/D	#N/D	#N/D	#N/D	39	H4023	Chemagic	2,034152744	52

SampleID	country	gender	Age	study	disease	BMI	hbi	cai	cd localization	cd behaviour	uc extent	dysbiosisindex	Time point	patientID	extraction protocol	shannon	chao
HSM7J4KI	USA	Male	16	HMP2	Healthy	18,8	#N/D	#N/D	#N/D	#N/D	#N/D	#N/D	41	H4023	Chemagic	2,739033499	58
HSM7J4KK	USA	Male	16	HMP2	Healthy	18,8	#N/D	#N/D	#N/D	#N/D	#N/D	#N/D	43	H4023	Chemagic	2,888892271	56
HSM7J4KM	USA	Male	16	HMP2	Healthy	18,8	#N/D	#N/D	#N/D	#N/D	#N/D	#N/D	45	H4023	Chemagic	2,170334941	50
HSM67VDT_P	USA	Male	11	HMP2	Healthy	15,4	#N/D	#N/D	#N/D	#N/D	#N/D	#N/D	0	H4024	Chemagic	2,11741016	49
HSM67VDT	USA	Male	11	HMP2	Healthy	15,4	#N/D	#N/D	#N/D	#N/D	#N/D	#N/D	0	H4024	Chemagic	2,105774375	48,5
HSM67VI3	USA	Male	11	HMP2	Healthy	15,4	#N/D	#N/D	#N/D	#N/D	#N/D	#N/D	2	H4024	Chemagic	2,207460652	45
HSM67VI5	USA	Male	11	HMP2	Healthy	15,4	#N/D	#N/D	#N/D	#N/D	#N/D	#N/D	3	H4024	Chemagic	2,389028617	49,5
HSM67VI7	USA	Male	11	HMP2	Healthy	15,4	#N/D	#N/D	#N/D	#N/D	#N/D	#N/D	6	H4024	Chemagic	2,206257265	47
HSM67VI9	USA	Male	11	HMP2	Healthy	15,4	#N/D	#N/D	#N/D	#N/D	#N/D	#N/D	7	H4024	Chemagic	2,2723142	52
HSM67VIB	USA	Male	11	HMP2	Healthy	15,4	#N/D	#N/D	#N/D	#N/D	#N/D	#N/D	9	H4024	Chemagic	2,216333269	49
HSM7CZ24	USA	Male	11	HMP2	Healthy	15,4	#N/D	#N/D	#N/D	#N/D	#N/D	#N/D	12	H4024	Chemagic	2,20833344	48
HSM7CZ26	USA	Male	11	HMP2	Healthy	15,4	#N/D	#N/D	#N/D	#N/D	#N/D	#N/D	14	H4024	Chemagic	2,389215196	44
HSM7CZ28	USA	Male	11	HMP2	Healthy	15,4	#N/D	#N/D	#N/D	#N/D	#N/D	#N/D	15	H4024	Chemagic	2,440189791	51
HSM7CZ2A	USA	Male	11	HMP2	Healthy	15,4	#N/D	#N/D	#N/D	#N/D	#N/D	#N/D	18	H4024	Chemagic	2,328108884	43
HSM7CZ2E	USA	Male	11	HMP2	Healthy	15,4	#N/D	#N/D	#N/D	#N/D	#N/D	#N/D	21	H4024	Chemagic	2,324014788	44

SampleID	country	gender	Age	study	disease	BMI	hbi	cai	cd localization	cd behaviour	uc extent	dysbiosisindex	Time point	patientID	extraction protocol	shannon	chao
HSM7J4HW	USA	Male	11	HMP2	Healthy	15,4	#N/D	#N/D	#N/D	#N/D	#N/D	#N/D	24	H4024	Chemagic	2,629137162	45
HSM7J4HY	USA	Male	11	HMP2	Healthy	15,4	#N/D	#N/D	#N/D	#N/D	#N/D	#N/D	25	H4024	Chemagic	2,203926137	44
HSM7J4I5	USA	Male	11	HMP2	Healthy	15,4	#N/D	#N/D	#N/D	#N/D	#N/D	#N/D	29	H4024	Chemagic	2,493354587	43
HSM7J4I7	USA	Male	11	HMP2	Healthy	15,4	#N/D	#N/D	#N/D	#N/D	#N/D	#N/D	32	H4024	Chemagic	2,498720052	44
HSM7J4I9	USA	Male	11	HMP2	Healthy	15,4	#N/D	#N/D	#N/D	#N/D	#N/D	#N/D	34	H4024	Chemagic	2,598550129	45
HSM7J4O1	USA	Male	11	HMP2	Healthy	15,4	#N/D	#N/D	#N/D	#N/D	#N/D	#N/D	38	H4024	Chemagic	2,677909861	47
HSM7J4NY	USA	Male	11	HMP2	Healthy	15,4	#N/D	#N/D	#N/D	#N/D	#N/D	#N/D	39	H4024	Chemagic	2,536351566	50
HSM7J4O3	USA	Male	11	HMP2	Healthy	15,4	#N/D	#N/D	#N/D	#N/D	#N/D	#N/D	40	H4024	Chemagic	2,609558705	44
HSM7J4O5	USA	Male	11	HMP2	Healthy	15,4	#N/D	#N/D	#N/D	#N/D	#N/D	#N/D	42	H4024	Chemagic	2,449047596	39
HSM7J4O7	USA	Male	11	HMP2	Healthy	15,4	#N/D	#N/D	#N/D	#N/D	#N/D	#N/D	44	H4024	Chemagic	2,517442403	48
HSM7J4O9	USA	Male	11	HMP2	Healthy	15,4	#N/D	#N/D	#N/D	#N/D	#N/D	#N/D	46	H4024	Chemagic	2,599134113	47
HSMA33NY	USA	Female	14	HMP2	Healthy	33,8	#N/D	#N/D	#N/D	#N/D	#N/D	#N/D	0	H4045	Chemagic	2,463202573	48
HSMA33J1_P	USA	Female	14	HMP2	Healthy	33,8	#N/D	#N/D	#N/D	#N/D	#N/D	#N/D	1	H4045	Chemagic	2,384054393	46
HSMA33J3	USA	Female	14	HMP2	Healthy	33,8	#N/D	#N/D	#N/D	#N/D	#N/D	#N/D	3	H4045	Chemagic	1,581856932	46
HSMA33J5	USA	Female	14	HMP2	Healthy	33,8	#N/D	#N/D	#N/D	#N/D	#N/D	#N/D	5	H4045	Chemagic	1,720340774	49

SampleID	country	gender	Age	study	disease	BMI	hbi	cai	cd localization	cd behaviour	uc extent	dysbiosisindex	Time point	patientID	extraction protocol	shannon	chao
HSMA33J7	USA	Female	14	HMP2	Healthy	33,8	#N/D	#N/D	#N/D	#N/D	#N/D	#N/D	7	H4045	Chemagic	2,526697345	48
HSMA33J9	USA	Female	14	HMP2	Healthy	33,8	#N/D	#N/D	#N/D	#N/D	#N/D	#N/D	10	H4045	Chemagic	2,7262155	49
HSMA33MS	USA	Female	14	HMP2	Healthy	33,8	#N/D	#N/D	#N/D	#N/D	#N/D	#N/D	16	H4045	Chemagic	1,807469028	44
HSMA33QM	USA	Female	14	HMP2	Healthy	33,8	#N/D	#N/D	#N/D	#N/D	#N/D	#N/D	23	H4045	Chemagic	2,599246431	53
HSMA33QO	USA	Female	14	HMP2	Healthy	33,8	#N/D	#N/D	#N/D	#N/D	#N/D	#N/D	25	H4045	Chemagic	1,208538684	42
HSMA33SE	USA	Female	14	HMP2	Healthy	33,8	#N/D	#N/D	#N/D	#N/D	#N/D	#N/D	33	H4045	Chemagic	2,808957621	51
HSMA33SG	USA	Female	14	HMP2	Healthy	33,8	#N/D	#N/D	#N/D	#N/D	#N/D	#N/D	35	H4045	Chemagic	2,774767882	50
HSMA33SI	USA	Female	14	HMP2	Healthy	33,8	#N/D	#N/D	#N/D	#N/D	#N/D	#N/D	37	H4045	Chemagic	2,901926246	51
HSMA33SK	USA	Female	14	HMP2	Healthy	33,8	#N/D	#N/D	#N/D	#N/D	#N/D	#N/D	39	H4045	Chemagic	2,758787574	50
CSM6J2H9_P	USA	Female	40	HMP2	Healthy	40,6	#N/D	#N/D	#N/D	#N/D	#N/D	#N/D	0	M2039	Chemagic	2,48516756	51
MSM6J2HN	USA	Female	40	HMP2	Healthy	40,6	#N/D	#N/D	#N/D	#N/D	#N/D	#N/D	2	M2039	Chemagic	2,114915584	42
MSM6J2HP	USA	Female	40	HMP2	Healthy	40,6	#N/D	#N/D	#N/D	#N/D	#N/D	#N/D	5	M2039	Chemagic	2,649484119	48
MSM6J2HR	USA	Female	40	HMP2	Healthy	40,6	#N/D	#N/D	#N/D	#N/D	#N/D	#N/D	7	M2039	Chemagic	2,242093023	40
MSM6J2HT	USA	Female	40	HMP2	Healthy	40,6	#N/D	#N/D	#N/D	#N/D	#N/D	#N/D	10	M2039	Chemagic	2,117325937	39
MSM6J2QL	USA	Female	40	HMP2	Healthy	40,6	#N/D	#N/D	#N/D	#N/D	#N/D	#N/D	16	M2039	Chemagic	2,299779026	40

SampleID	country	gender	Age	study	disease	BMI	hbi	cai	cd localization	cd behaviour	uc extent	dysbiosisindex	Time point	patientID	extraction protocol	shannon	chao
MSM6J2QH	USA	Female	40	HMP2	Healthy	40,6	#N/D	#N/D	#N/D	#N/D	#N/D	#N/D	18	M2039	Chemagic	2,432815386	38,5
MSM6J2QP	USA	Female	40	HMP2	Healthy	40,6	#N/D	#N/D	#N/D	#N/D	#N/D	#N/D	20	M2039	Chemagic	2,402267904	33,5
MSM6J2QJ	USA	Female	40	HMP2	Healthy	40,6	#N/D	#N/D	#N/D	#N/D	#N/D	#N/D	24	M2039	Chemagic	2,361459271	42
MSM6J2QF	USA	Female	40	HMP2	Healthy	40,6	#N/D	#N/D	#N/D	#N/D	#N/D	#N/D	26	M2039	Chemagic	2,222207987	34
MSM79H5Q	USA	Female	40	HMP2	Healthy	40,6	#N/D	#N/D	#N/D	#N/D	#N/D	#N/D	31	M2039	Chemagic	2,252089428	41
MSM79H5U	USA	Female	40	HMP2	Healthy	40,6	#N/D	#N/D	#N/D	#N/D	#N/D	#N/D	33	M2039	Chemagic	2,473315259	40
MSM79H5Y	USA	Female	40	HMP2	Healthy	40,6	#N/D	#N/D	#N/D	#N/D	#N/D	#N/D	35	M2039	Chemagic	2,286966575	42
MSM79H5S	USA	Female	40	HMP2	Healthy	40,6	#N/D	#N/D	#N/D	#N/D	#N/D	#N/D	37	M2039	Chemagic	2,542419841	50
MSM79HAH	USA	Female	40	HMP2	Healthy	40,6	#N/D	#N/D	#N/D	#N/D	#N/D	#N/D	48	M2039	Chemagic	2,252781347	40
MSM6J2JF_P	USA	Male	55	HMP2	Healthy	22,7	#N/D	#N/D	#N/D	#N/D	#N/D	#N/D	0	M2041	Chemagic	2,453497693	61
MSM6J2JN	USA	Male	55	HMP2	Healthy	22,7	#N/D	#N/D	#N/D	#N/D	#N/D	#N/D	2	M2041	Chemagic	2,251316346	48
MSM6J2JP	USA	Male	55	HMP2	Healthy	22,7	#N/D	#N/D	#N/D	#N/D	#N/D	#N/D	3	M2041	Chemagic	1,715128513	45
MSM6J2JR	USA	Male	55	HMP2	Healthy	22,7	#N/D	#N/D	#N/D	#N/D	#N/D	#N/D	8	M2041	Chemagic	2,201028876	46
MSM6J2JT	USA	Male	55	HMP2	Healthy	22,7	#N/D	#N/D	#N/D	#N/D	#N/D	#N/D	9	M2041	Chemagic	2,078719345	41
MSM6J2LV	USA	Male	55	HMP2	Healthy	22,7	#N/D	#N/D	#N/D	#N/D	#N/D	#N/D	16	M2041	Chemagic	2,621128026	45

SampleID	country	gender	Age	study	disease	BMI	hbi	cai	cd localization	cd behaviour	uc extent	dysbiosisindex	Time point	patientID	extraction protocol	shannon	chao
MSM6J2SE	USA	Male	55	HMP2	Healthy	22,7	#N/D	#N/D	#N/D	#N/D	#N/D	#N/D	18	M2041	Chemagic	2,56344078	46
MSM6J2SI	USA	Male	55	HMP2	Healthy	22,7	#N/D	#N/D	#N/D	#N/D	#N/D	#N/D	24	M2041	Chemagic	2,634400755	44
MSM6J2SK	USA	Male	55	HMP2	Healthy	22,7	#N/D	#N/D	#N/D	#N/D	#N/D	#N/D	25	M2041	Chemagic	1,715748888	42
MSM79HES	USA	Male	55	HMP2	Healthy	22,7	#N/D	#N/D	#N/D	#N/D	#N/D	#N/D	33	M2041	Chemagic	2,873730507	48
MSM79HEU	USA	Male	55	HMP2	Healthy	22,7	#N/D	#N/D	#N/D	#N/D	#N/D	#N/D	35	M2041	Chemagic	2,146944343	43
MSM79HEW	USA	Male	55	HMP2	Healthy	22,7	#N/D	#N/D	#N/D	#N/D	#N/D	#N/D	36	M2041	Chemagic	2,3066504	45
MSM79HEY	USA	Male	55	HMP2	Healthy	22,7	#N/D	#N/D	#N/D	#N/D	#N/D	#N/D	39	M2041	Chemagic	1,865034973	44
MSM79HB6	USA	Male	55	HMP2	Healthy	22,7	#N/D	#N/D	#N/D	#N/D	#N/D	#N/D	50	M2041	Chemagic	2,440671805	45
MSM6J2JH_P	USA	Male	44	HMP2	Healthy	24,5	#N/D	#N/D	#N/D	#N/D	#N/D	#N/D	0	M2042	Chemagic	1,647670692	48
MSM6J2JZ	USA	Male	44	HMP2	Healthy	24,5	#N/D	#N/D	#N/D	#N/D	#N/D	#N/D	1	M2042	Chemagic	1,508851863	48
MSM6J2K2	USA	Male	44	HMP2	Healthy	24,5	#N/D	#N/D	#N/D	#N/D	#N/D	#N/D	2	M2042	Chemagic	1,254805412	52
MSM6J2K4	USA	Male	44	HMP2	Healthy	24,5	#N/D	#N/D	#N/D	#N/D	#N/D	#N/D	3	M2042	Chemagic	2,225857127	54
MSM6J2K6	USA	Male	44	HMP2	Healthy	24,5	#N/D	#N/D	#N/D	#N/D	#N/D	#N/D	4	M2042	Chemagic	1,626635253	54
MSM6J2K8	USA	Male	44	HMP2	Healthy	24,5	#N/D	#N/D	#N/D	#N/D	#N/D	#N/D	5	M2042	Chemagic	2,909597555	47
MSM6J2KA	USA	Male	44	HMP2	Healthy	24,5	#N/D	#N/D	#N/D	#N/D	#N/D	#N/D	6	M2042	Chemagic	2,756035525	56

SampleID	country	gender	Age	study	disease	BMI	hbi	cai	cd localization	cd behaviour	uc extent	dysbiosisindex	Time point	patientID	extraction protocol	shannon	chao
MSM6J2RK	USA	Male	44	HMP2	Healthy	24,5	#N/D	#N/D	#N/D	#N/D	#N/D	#N/D	12	M2042	Chemagic	2,510130956	53
MSM6J2RM	USA	Male	44	HMP2	Healthy	24,5	#N/D	#N/D	#N/D	#N/D	#N/D	#N/D	14	M2042	Chemagic	1,946975783	46
MSM6J2RO	USA	Male	44	HMP2	Healthy	24,5	#N/D	#N/D	#N/D	#N/D	#N/D	#N/D	15	M2042	Chemagic	1,497578229	57
MSM6J2RQ	USA	Male	44	HMP2	Healthy	24,5	#N/D	#N/D	#N/D	#N/D	#N/D	#N/D	16	M2042	Chemagic	2,343708926	49
MSM6J2RU	USA	Male	44	HMP2	Healthy	24,5	#N/D	#N/D	#N/D	#N/D	#N/D	#N/D	19	M2042	Chemagic	2,752721106	57
MSM6J2RS	USA	Male	44	HMP2	Healthy	24,5	#N/D	#N/D	#N/D	#N/D	#N/D	#N/D	20	M2042	Chemagic	0,593643975	41
MSM79H6D	USA	Male	44	HMP2	Healthy	24,5	#N/D	#N/D	#N/D	#N/D	#N/D	#N/D	26	M2042	Chemagic	2,884550104	56
MSM79H6F	USA	Male	44	HMP2	Healthy	24,5	#N/D	#N/D	#N/D	#N/D	#N/D	#N/D	27	M2042	Chemagic	2,954286495	33
MSM79H6J	USA	Male	44	HMP2	Healthy	24,5	#N/D	#N/D	#N/D	#N/D	#N/D	#N/D	28	M2042	Chemagic	2,589349056	52
MSM79H6H	USA	Male	44	HMP2	Healthy	24,5	#N/D	#N/D	#N/D	#N/D	#N/D	#N/D	29	M2042	Chemagic	1,780740675	43
MSM79H6L	USA	Male	44	HMP2	Healthy	24,5	#N/D	#N/D	#N/D	#N/D	#N/D	#N/D	30	M2042	Chemagic	2,547603648	50
MSM79H6N	USA	Male	44	HMP2	Healthy	24,5	#N/D	#N/D	#N/D	#N/D	#N/D	#N/D	31	M2042	Chemagic	2,587990378	53,33333333
MSM79H9Y	USA	Male	44	HMP2	Healthy	24,5	#N/D	#N/D	#N/D	#N/D	#N/D	#N/D	34	M2042	Chemagic	2,672021588	48
MSM79HA1	USA	Male	44	HMP2	Healthy	24,5	#N/D	#N/D	#N/D	#N/D	#N/D	#N/D	35	M2042	Chemagic	2,271332059	46
MSM79HA3	USA	Male	44	HMP2	Healthy	24,5	#N/D	#N/D	#N/D	#N/D	#N/D	#N/D	40	M2042	Chemagic	1,293929629	42

SampleID	country	gender	Age	study	disease	BMI	hbi	cai	cd localization	cd behaviour	uc extent	dysbiosisindex	Time point	patientID	extraction protocol	shannon	chao
MSM79HA7	USA	Male	44	HMP2	Healthy	24,5	#N/D	#N/D	#N/D	#N/D	#N/D	#N/D	44	M2042	Chemagic	2,560258827	50,5
MSM6J2PO	USA	Male	57	HMP2	Healthy	25,1	#N/D	#N/D	#N/D	#N/D	#N/D	#N/D	0	M2047	Chemagic	2,517021609	44
MSM6J2PQ_P	USA	Male	57	HMP2	Healthy	25,1	#N/D	#N/D	#N/D	#N/D	#N/D	#N/D	2	M2047	Chemagic	3,247843469	54
MSM6J2PS	USA	Male	57	HMP2	Healthy	25,1	#N/D	#N/D	#N/D	#N/D	#N/D	#N/D	4	M2047	Chemagic	2,665250283	60
MSM6J2PU	USA	Male	57	HMP2	Healthy	25,1	#N/D	#N/D	#N/D	#N/D	#N/D	#N/D	7	M2047	Chemagic	2,756638131	40
MSM6J2PW	USA	Male	57	HMP2	Healthy	25,1	#N/D	#N/D	#N/D	#N/D	#N/D	#N/D	10	M2047	Chemagic	2,146582349	46
MSM79HC4	USA	Male	57	HMP2	Healthy	25,1	#N/D	#N/D	#N/D	#N/D	#N/D	#N/D	15	M2047	Chemagic	2,062852881	52
MSM79HC8	USA	Male	57	HMP2	Healthy	25,1	#N/D	#N/D	#N/D	#N/D	#N/D	#N/D	20	M2047	Chemagic	2,140856735	53
MSM79H7O	USA	Male	57	HMP2	Healthy	25,1	#N/D	#N/D	#N/D	#N/D	#N/D	#N/D	25	M2047	Chemagic	2,246715216	47
MSM79H7Q	USA	Male	57	HMP2	Healthy	25,1	#N/D	#N/D	#N/D	#N/D	#N/D	#N/D	28	M2047	Chemagic	2,422165521	53
MSM79H7W	USA	Male	57	HMP2	Healthy	25,1	#N/D	#N/D	#N/D	#N/D	#N/D	#N/D	32	M2047	Chemagic	2,431846355	53
MSM79H7Y	USA	Male	57	HMP2	Healthy	25,1	#N/D	#N/D	#N/D	#N/D	#N/D	#N/D	33	M2047	Chemagic	2,647220725	56
MSM9VZNH	USA	Male	57	HMP2	Healthy	25,1	#N/D	#N/D	#N/D	#N/D	#N/D	#N/D	35	M2047	Chemagic	2,838159948	53
MSM9VZNH_TR	USA	Male	57	HMP2	Healthy	25,1	#N/D	#N/D	#N/D	#N/D	#N/D	#N/D	35	M2047	Chemagic	2,757591689	51
MSM9VZNL	USA	Male	57	HMP2	Healthy	25,1	#N/D	#N/D	#N/D	#N/D	#N/D	#N/D	39	M2047	Chemagic	2,816756763	62

SampleID	country	gender	Age	study	disease	BMI	hbi	cai	cd localization	cd behaviour	uc extent	dysbiosisindex	Time point	patientID	extraction protocol	shannon	chao
MSM79H52_P	USA	Female	62	HMP2	Healthy	20,2	#N/D	#N/D	#N/D	#N/D	#N/D	#N/D	2	M2060	Chemagic	2,126555841	30
MSM79H54	USA	Female	62	HMP2	Healthy	20,2	#N/D	#N/D	#N/D	#N/D	#N/D	#N/D	4	M2060	Chemagic	2,106437469	33
MSM79H58	USA	Female	62	HMP2	Healthy	20,2	#N/D	#N/D	#N/D	#N/D	#N/D	#N/D	8	M2060	Chemagic	2,389364274	40
MSM79H5A	USA	Female	62	HMP2	Healthy	20,2	#N/D	#N/D	#N/D	#N/D	#N/D	#N/D	10	M2060	Chemagic	2,157055521	41
MSM79H9A	USA	Female	62	HMP2	Healthy	20,2	#N/D	#N/D	#N/D	#N/D	#N/D	#N/D	14	M2060	Chemagic	2,314393329	39
MSM79H9C	USA	Female	62	HMP2	Healthy	20,2	#N/D	#N/D	#N/D	#N/D	#N/D	#N/D	16	M2060	Chemagic	2,214542943	37
MSM79H9G	USA	Female	62	HMP2	Healthy	20,2	#N/D	#N/D	#N/D	#N/D	#N/D	#N/D	22	M2060	Chemagic	2,13385746	33
MSM79H9K	USA	Female	62	HMP2	Healthy	20,2	#N/D	#N/D	#N/D	#N/D	#N/D	#N/D	23	M2060	Chemagic	2,325407829	40
MSM9VZF7	USA	Female	62	HMP2	Healthy	20,2	#N/D	#N/D	#N/D	#N/D	#N/D	#N/D	27	M2060	Chemagic	2,197055993	35
MSM9VZFF	USA	Female	62	HMP2	Healthy	20,2	#N/D	#N/D	#N/D	#N/D	#N/D	#N/D	34	M2060	Chemagic	2,226521662	39
MSM9VZFH	USA	Female	62	HMP2	Healthy	20,2	#N/D	#N/D	#N/D	#N/D	#N/D	#N/D	36	M2060	Chemagic	2,187454376	45
MSM9VZPT	USA	Female	62	HMP2	Healthy	20,2	#N/D	#N/D	#N/D	#N/D	#N/D	#N/D	38	M2060	Chemagic	2,753979353	41
MSM79H5E	USA	Male	56	HMP2	Healthy	26,3	#N/D	#N/D	#N/D	#N/D	#N/D	#N/D	2	M2061	Chemagic	2,82088692	52
MSM79H5G	USA	Male	56	HMP2	Healthy	26,3	#N/D	#N/D	#N/D	#N/D	#N/D	#N/D	4	M2061	Chemagic	2,653217818	52
MSM79H5K	USA	Male	56	HMP2	Healthy	26,3	#N/D	#N/D	#N/D	#N/D	#N/D	#N/D	8	M2061	Chemagic	3,016021158	55

SampleID	country	gender	Age	study	disease	BMI	hbi	cai	cd localization	cd behaviour	uc extent	dysbiosisindex	Time point	patientID	extraction protocol	shannon	chao
MSM79H5M	USA	Male	56	HMP2	Healthy	26,3	#N/D	#N/D	#N/D	#N/D	#N/D	#N/D	10	M2061	Chemagic	3,158379728	55
MSM79H9M	USA	Male	56	HMP2	Healthy	26,3	#N/D	#N/D	#N/D	#N/D	#N/D	#N/D	15	M2061	Chemagic	2,501742187	51
MSM79H9Q	USA	Male	56	HMP2	Healthy	26,3	#N/D	#N/D	#N/D	#N/D	#N/D	#N/D	19	M2061	Chemagic	2,189315899	51
MSM79H9W	USA	Male	56	HMP2	Healthy	26,3	#N/D	#N/D	#N/D	#N/D	#N/D	#N/D	25	M2061	Chemagic	2,905973803	56
MSM9VZGO	USA	Male	56	HMP2	Healthy	26,3	#N/D	#N/D	#N/D	#N/D	#N/D	#N/D	30	M2061	Chemagic	2,715338324	56
MSM9VZGS	USA	Male	56	HMP2	Healthy	26,3	#N/D	#N/D	#N/D	#N/D	#N/D	#N/D	34	M2061	Chemagic	2,779029038	52
MSM9VZGU	USA	Male	56	HMP2	Healthy	26,3	#N/D	#N/D	#N/D	#N/D	#N/D	#N/D	36	M2061	Chemagic	2,670776655	52
MSM9VZLJ	USA	Male	56	HMP2	Healthy	26,3	#N/D	#N/D	#N/D	#N/D	#N/D	#N/D	38	M2061	Chemagic	2,887913998	51
MSM9VZHB	USA	Male	56	HMP2	Healthy	26,3	#N/D	#N/D	#N/D	#N/D	#N/D	#N/D	46	M2061	Chemagic	2,281509388	47
MSM9VZHF	USA	Male	56	HMP2	Healthy	26,3	#N/D	#N/D	#N/D	#N/D	#N/D	#N/D	51	M2061	Chemagic	3,21414296	53
MSM79HCG	USA	Male	51	HMP2	Healthy	28,7	#N/D	#N/D	#N/D	#N/D	#N/D	#N/D	0	M2072	Chemagic	1,34701835	34
MSM79HCI	USA	Male	51	HMP2	Healthy	28,7	#N/D	#N/D	#N/D	#N/D	#N/D	#N/D	4	M2072	Chemagic	1,342523831	30
MSM79HCK	USA	Male	51	HMP2	Healthy	28,7	#N/D	#N/D	#N/D	#N/D	#N/D	#N/D	5	M2072	Chemagic	1,268627616	21
MSM79HCN_P	USA	Male	51	HMP2	Healthy	28,7	#N/D	#N/D	#N/D	#N/D	#N/D	#N/D	6	M2072	Chemagic	1,245410843	21
MSM79HCP	USA	Male	51	HMP2	Healthy	28,7	#N/D	#N/D	#N/D	#N/D	#N/D	#N/D	7	M2072	Chemagic	1,348698949	30

SampleID	country	gender	Age	study	disease	BMI	hbi	cai	cd localization	cd behaviour	uc extent	dysbiosisindex	Time point	patientID	extraction protocol	shannon	chao
MSM79HCR	USA	Male	51	HMP2	Healthy	28,7	#N/D	#N/D	#N/D	#N/D	#N/D	#N/D	9	M2072	Chemagic	1,170165779	29
MSM79H81	USA	Male	51	HMP2	Healthy	28,7	#N/D	#N/D	#N/D	#N/D	#N/D	#N/D	11	M2072	Chemagic	1,776940464	36
MSM79H83	USA	Male	51	HMP2	Healthy	28,7	#N/D	#N/D	#N/D	#N/D	#N/D	#N/D	13	M2072	Chemagic	1,191461284	36
MSM79H85	USA	Male	51	HMP2	Healthy	28,7	#N/D	#N/D	#N/D	#N/D	#N/D	#N/D	15	M2072	Chemagic	1,504920383	34
MSM79H87	USA	Male	51	HMP2	Healthy	28,7	#N/D	#N/D	#N/D	#N/D	#N/D	#N/D	17	M2072	Chemagic	1,294433768	34
MSM79H89	USA	Male	51	HMP2	Healthy	28,7	#N/D	#N/D	#N/D	#N/D	#N/D	#N/D	19	M2072	Chemagic	1,947363484	41
MSM79H8B	USA	Male	51	HMP2	Healthy	28,7	#N/D	#N/D	#N/D	#N/D	#N/D	#N/D	21	M2072	Chemagic	1,599033994	32
MSM9VZOG	USA	Male	51	HMP2	Healthy	28,7	#N/D	#N/D	#N/D	#N/D	#N/D	#N/D	24	M2072	Chemagic	1,916152238	46,5
MSM9VZOI	USA	Male	51	HMP2	Healthy	28,7	#N/D	#N/D	#N/D	#N/D	#N/D	#N/D	25	M2072	Chemagic	1,37393885	38
MSM9VZOK	USA	Male	51	HMP2	Healthy	28,7	#N/D	#N/D	#N/D	#N/D	#N/D	#N/D	27	M2072	Chemagic	2,203093791	36
MSM9VZOM	USA	Male	51	HMP2	Healthy	28,7	#N/D	#N/D	#N/D	#N/D	#N/D	#N/D	29	M2072	Chemagic	1,188723517	33
MSM9VZOO	USA	Male	51	HMP2	Healthy	28,7	#N/D	#N/D	#N/D	#N/D	#N/D	#N/D	31	M2072	Chemagic	1,532810241	35
MSM9VZOQ	USA	Male	51	HMP2	Healthy	28,7	#N/D	#N/D	#N/D	#N/D	#N/D	#N/D	33	M2072	Chemagic	1,746643373	34
MSM9VZHJ	USA	Male	51	HMP2	Healthy	28,7	#N/D	#N/D	#N/D	#N/D	#N/D	#N/D	35	M2072	Chemagic	1,750182455	32
MSM9VZHL	USA	Male	51	HMP2	Healthy	28,7	#N/D	#N/D	#N/D	#N/D	#N/D	#N/D	37	M2072	Chemagic	2,064031155	28

SampleID	country	gender	Age	study	disease	BMI	hbi	cai	cd localization	cd behaviour	uc extent	dysbiosisindex	Time point	patientID	extraction protocol	shannon	chao
MSM9VZHN	USA	Male	51	HMP2	Healthy	28,7	#N/D	#N/D	#N/D	#N/D	#N/D	#N/D	39	M2072	Chemagic	1,699496845	38
MSM9VZHP	USA	Male	51	HMP2	Healthy	28,7	#N/D	#N/D	#N/D	#N/D	#N/D	#N/D	41	M2072	Chemagic	1,916427651	32
MSM9VZHR	USA	Male	51	HMP2	Healthy	28,7	#N/D	#N/D	#N/D	#N/D	#N/D	#N/D	43	M2072	Chemagic	1,910325545	41
MSM9VZHT	USA	Male	51	HMP2	Healthy	28,7	#N/D	#N/D	#N/D	#N/D	#N/D	#N/D	45	M2072	Chemagic	2,051189794	38
MSM79H8D	USA	Male	61	HMP2	Healthy	27,2	#N/D	#N/D	#N/D	#N/D	#N/D	#N/D	0	M2075	Chemagic	2,469075927	50,33333333
MSM79H8F	USA	Male	61	HMP2	Healthy	27,2	#N/D	#N/D	#N/D	#N/D	#N/D	#N/D	2	M2075	Chemagic	1,997458718	33
MSM79H8H	USA	Male	61	HMP2	Healthy	27,2	#N/D	#N/D	#N/D	#N/D	#N/D	#N/D	3	M2075	Chemagic	2,729699487	46
MSM79H8J_P	USA	Male	61	HMP2	Healthy	27,2	#N/D	#N/D	#N/D	#N/D	#N/D	#N/D	5	M2075	Chemagic	2,447894756	57
MSM79H8L	USA	Male	61	HMP2	Healthy	27,2	#N/D	#N/D	#N/D	#N/D	#N/D	#N/D	7	M2075	Chemagic	2,320016513	43
MSM79H8N	USA	Male	61	HMP2	Healthy	27,2	#N/D	#N/D	#N/D	#N/D	#N/D	#N/D	9	M2075	Chemagic	1,767018568	39
MSM9VZJZ	USA	Male	61	HMP2	Healthy	27,2	#N/D	#N/D	#N/D	#N/D	#N/D	#N/D	14	M2075	Chemagic	2,282702413	50
MSM9VZGW	USA	Male	61	HMP2	Healthy	27,2	#N/D	#N/D	#N/D	#N/D	#N/D	#N/D	24	M2075	Chemagic	2,482799836	48
MSM9VZGY	USA	Male	61	HMP2	Healthy	27,2	#N/D	#N/D	#N/D	#N/D	#N/D	#N/D	26	M2075	Chemagic	2,378089305	43
MSM9VZH7	USA	Male	61	HMP2	Healthy	27,2	#N/D	#N/D	#N/D	#N/D	#N/D	#N/D	34	M2075	Chemagic	2,56305813	46
MSMAPC7J	USA	Male	61	HMP2	Healthy	27,2	#N/D	#N/D	#N/D	#N/D	#N/D	#N/D	37	M2075	Chemagic	2,663945165	50

SampleID	country	gender	Age	study	disease	BMI	hbi	cai	cd localization	cd behaviour	uc extent	dysbiosisindex	Time point	patientID	extraction protocol	shannon	chao
MSM9VZMM	USA	Female	23	HMP2	Healthy	21,6	#N/D	#N/D	#N/D	#N/D	#N/D	#N/D	0	M2084	Chemagic	2,438301341	42,5
MSM9VZMO	USA	Female	23	HMP2	Healthy	21,6	#N/D	#N/D	#N/D	#N/D	#N/D	#N/D	2	M2084	Chemagic	2,787827566	45
MSM9VZMS	USA	Female	23	HMP2	Healthy	21,6	#N/D	#N/D	#N/D	#N/D	#N/D	#N/D	6	M2084	Chemagic	2,862326103	46
MSM9VZMU	USA	Female	23	HMP2	Healthy	21,6	#N/D	#N/D	#N/D	#N/D	#N/D	#N/D	8	M2084	Chemagic	2,89667942	46
MSM9VZMW	USA	Female	23	HMP2	Healthy	21,6	#N/D	#N/D	#N/D	#N/D	#N/D	#N/D	10	M2084	Chemagic	2,832931763	43,5
MSM9VZL7	USA	Female	23	HMP2	Healthy	21,6	#N/D	#N/D	#N/D	#N/D	#N/D	#N/D	12	M2084	Chemagic	2,809390119	43
MSM9VZL9	USA	Female	23	HMP2	Healthy	21,6	#N/D	#N/D	#N/D	#N/D	#N/D	#N/D	14	M2084	Chemagic	2,821714855	54
MSM9VZLB	USA	Female	23	HMP2	Healthy	21,6	#N/D	#N/D	#N/D	#N/D	#N/D	#N/D	16	M2084	Chemagic	2,966558148	51
MSM9VZLD	USA	Female	23	HMP2	Healthy	21,6	#N/D	#N/D	#N/D	#N/D	#N/D	#N/D	18	M2084	Chemagic	2,874753324	42
MSM9VZLF	USA	Female	23	HMP2	Healthy	21,6	#N/D	#N/D	#N/D	#N/D	#N/D	#N/D	21	M2084	Chemagic	2,822433501	51
MSM9VZLH	USA	Female	23	HMP2	Healthy	21,6	#N/D	#N/D	#N/D	#N/D	#N/D	#N/D	23	M2084	Chemagic	2,886158575	46
MSMA26BB	USA	Female	23	HMP2	Healthy	21,6	#N/D	#N/D	#N/D	#N/D	#N/D	#N/D	25	M2084	Chemagic	2,814161245	52
MSMA26BD	USA	Female	23	HMP2	Healthy	21,6	#N/D	#N/D	#N/D	#N/D	#N/D	#N/D	27	M2084	Chemagic	2,926824084	38
MSMA26BF	USA	Female	23	HMP2	Healthy	21,6	#N/D	#N/D	#N/D	#N/D	#N/D	#N/D	29	M2084	Chemagic	2,778486232	41
MSMA26BH	USA	Female	23	HMP2	Healthy	21,6	#N/D	#N/D	#N/D	#N/D	#N/D	#N/D	31	M2084	Chemagic	2,795932293	41

SampleID	country	gender	Age	study	disease	BMI	hbi	cai	cd localization	cd behaviour	uc extent	dysbiosisindex	Time point	patientID	extraction protocol	shannon	chao
MSMA26BJ	USA	Female	23	HMP2	Healthy	21,6	#N/D	#N/D	#N/D	#N/D	#N/D	#N/D	33	M2084	Chemagic	2,848710028	50
MSMA26BL	USA	Female	23	HMP2	Healthy	21,6	#N/D	#N/D	#N/D	#N/D	#N/D	#N/D	35	M2084	Chemagic	2,597394355	48
MSMAPC6K	USA	Female	23	HMP2	Healthy	21,6	#N/D	#N/D	#N/D	#N/D	#N/D	#N/D	37	M2084	Chemagic	2,879964534	51
MSMAPC6M	USA	Female	23	HMP2	Healthy	21,6	#N/D	#N/D	#N/D	#N/D	#N/D	#N/D	39	M2084	Chemagic	2,895881925	46
MSMAPC6O	USA	Female	23	HMP2	Healthy	21,6	#N/D	#N/D	#N/D	#N/D	#N/D	#N/D	41	M2084	Chemagic	2,91616134	49
MSMB4LZZ	USA	Female	23	HMP2	Healthy	21,6	#N/D	#N/D	#N/D	#N/D	#N/D	#N/D	43	M2084	Chemagic	2,828292809	47
MSMB4LZV	USA	Female	23	HMP2	Healthy	21,6	#N/D	#N/D	#N/D	#N/D	#N/D	#N/D	45	M2084	Chemagic	2,707357232	46
MSMB4LZX	USA	Female	23	HMP2	Healthy	21,6	#N/D	#N/D	#N/D	#N/D	#N/D	#N/D	47	M2084	Chemagic	2,846203953	49
PSM6XBTR	USA	Male	15	HMP2	Healthy	23,8	#N/D	#N/D	#N/D	#N/D	#N/D	#N/D	2	P6014	Chemagic	2,043992206	46
PSM6XBTT	USA	Male	15	HMP2	Healthy	23,8	#N/D	#N/D	#N/D	#N/D	#N/D	#N/D	3	P6014	Chemagic	2,379991655	52
PSM6XBTX	USA	Male	15	HMP2	Healthy	23,8	#N/D	#N/D	#N/D	#N/D	#N/D	#N/D	8	P6014	Chemagic	2,259707238	49
PSM6XBTZ_P	USA	Male	15	HMP2	Healthy	23,8	#N/D	#N/D	#N/D	#N/D	#N/D	#N/D	9	P6014	Chemagic	2,176450925	41
PSM6XBU2	USA	Male	15	HMP2	Healthy	23,8	#N/D	#N/D	#N/D	#N/D	#N/D	#N/D	12	P6014	Chemagic	2,321257722	55
PSM7J1DF	USA	Male	15	HMP2	Healthy	23,8	#N/D	#N/D	#N/D	#N/D	#N/D	#N/D	17	P6014	Chemagic	2,676347978	49
PSM7J1DL	USA	Male	15	HMP2	Healthy	23,8	#N/D	#N/D	#N/D	#N/D	#N/D	#N/D	22	P6014	Chemagic	2,69412721	56

SampleID	country	gender	Age	study	disease	BMI	hbi	cai	cd localization	cd behaviour	uc extent	dysbiosisindex	Time point	patientID	extraction protocol	shannon	chao
PSM7J14X	USA	Male	15	HMP2	Healthy	23,8	#N/D	#N/D	#N/D	#N/D	#N/D	#N/D	28	P6014	Chemagic	2,840875719	55
PSM7J154	USA	Male	15	HMP2	Healthy	23,8	#N/D	#N/D	#N/D	#N/D	#N/D	#N/D	33	P6014	Chemagic	2,52679218	51
PSM7J156	USA	Male	15	HMP2	Healthy	23,8	#N/D	#N/D	#N/D	#N/D	#N/D	#N/D	36	P6014	Chemagic	2,454688691	48
PSM6XBW3	USA	Female	17	HMP2	Healthy	20,9	#N/D	#N/D	#N/D	#N/D	#N/D	#N/D	0	P6018	Chemagic	1,897762404	13
PSM7J19X_P	USA	Female	17	HMP2	Healthy	20,9	#N/D	#N/D	#N/D	#N/D	#N/D	#N/D	2	P6018	Chemagic	1,546102783	11
PSM7J19Z	USA	Female	17	HMP2	Healthy	20,9	#N/D	#N/D	#N/D	#N/D	#N/D	#N/D	4	P6018	Chemagic	2,084837745	12
PSM7J1A2	USA	Female	17	HMP2	Healthy	20,9	#N/D	#N/D	#N/D	#N/D	#N/D	#N/D	6	P6018	Chemagic	2,138736287	13
PSM7J1A4	USA	Female	17	HMP2	Healthy	20,9	#N/D	#N/D	#N/D	#N/D	#N/D	#N/D	8	P6018	Chemagic	2,597344062	42
PSM7J1A6	USA	Female	17	HMP2	Healthy	20,9	#N/D	#N/D	#N/D	#N/D	#N/D	#N/D	10	P6018	Chemagic	1,042157682	5
PSM7J1A8	USA	Female	17	HMP2	Healthy	20,9	#N/D	#N/D	#N/D	#N/D	#N/D	#N/D	12	P6018	Chemagic	1,166152332	12
PSM7J17V	USA	Female	17	HMP2	Healthy	20,9	#N/D	#N/D	#N/D	#N/D	#N/D	#N/D	14	P6018	Chemagic	1,487635455	14
PSM7J17X	USA	Female	17	HMP2	Healthy	20,9	#N/D	#N/D	#N/D	#N/D	#N/D	#N/D	16	P6018	Chemagic	1,183295316	15
PSM7J17Z	USA	Female	17	HMP2	Healthy	20,9	#N/D	#N/D	#N/D	#N/D	#N/D	#N/D	18	P6018	Chemagic	1,582903838	16
PSM7J182	USA	Female	17	HMP2	Healthy	20,9	#N/D	#N/D	#N/D	#N/D	#N/D	#N/D	20	P6018	Chemagic	1,537408995	20
PSM7J184	USA	Female	17	HMP2	Healthy	20,9	#N/D	#N/D	#N/D	#N/D	#N/D	#N/D	22	P6018	Chemagic	1,19159811	18

SampleID	country	gender	Age	study	disease	BMI	hbi	cai	cd localization	cd behaviour	uc extent	dysbiosisindex	Time point	patientID	extraction protocol	shannon	chao
PSM7J186	USA	Female	17	HMP2	Healthy	20,9	#N/D	#N/D	#N/D	#N/D	#N/D	#N/D	24	P6018	Chemagic	1,270470941	19
PSM7J15K	USA	Female	17	HMP2	Healthy	20,9	#N/D	#N/D	#N/D	#N/D	#N/D	#N/D	26	P6018	Chemagic	1,70838706	18
PSM7J15M	USA	Female	17	HMP2	Healthy	20,9	#N/D	#N/D	#N/D	#N/D	#N/D	#N/D	28	P6018	Chemagic	1,711399765	20
PSM7J15O	USA	Female	17	HMP2	Healthy	20,9	#N/D	#N/D	#N/D	#N/D	#N/D	#N/D	30	P6018	Chemagic	1,602861522	21
PSM7J15Q	USA	Female	17	HMP2	Healthy	20,9	#N/D	#N/D	#N/D	#N/D	#N/D	#N/D	32	P6018	Chemagic	2,19459922	21
PSM7J15S	USA	Female	17	HMP2	Healthy	20,9	#N/D	#N/D	#N/D	#N/D	#N/D	#N/D	34	P6018	Chemagic	2,296466429	28
PSM7J15U	USA	Female	17	HMP2	Healthy	20,9	#N/D	#N/D	#N/D	#N/D	#N/D	#N/D	36	P6018	Chemagic	1,69500422	23
PSMA265D	USA	Female	17	HMP2	Healthy	20,9	#N/D	#N/D	#N/D	#N/D	#N/D	#N/D	38	P6018	Chemagic	1,428583735	22
PSMA265F	USA	Female	17	HMP2	Healthy	20,9	#N/D	#N/D	#N/D	#N/D	#N/D	#N/D	40	P6018	Chemagic	1,169302161	20
PSMA265L	USA	Female	17	HMP2	Healthy	20,9	#N/D	#N/D	#N/D	#N/D	#N/D	#N/D	42	P6018	Chemagic	2,207616503	22
PSMA265J	USA	Female	17	HMP2	Healthy	20,9	#N/D	#N/D	#N/D	#N/D	#N/D	#N/D	44	P6018	Chemagic	1,753259332	22
PSMA265J_TR	USA	Female	17	HMP2	Healthy	20,9	#N/D	#N/D	#N/D	#N/D	#N/D	#N/D	44	P6018	Chemagic	1,753259332	22
PSMA265H	USA	Female	17	HMP2	Healthy	20,9	#N/D	#N/D	#N/D	#N/D	#N/D	#N/D	46	P6018	Chemagic	1,672972702	23
