# Supplementary figures and images for: Intercontinental Gut Microbiome Variances in IBD
Source: Int J Mol Sci. 2022 Sep 17;23(18):10868. doi: 10.3390/ijms231810868 (PMC9506019; doi:10.3390/ijms231810868)

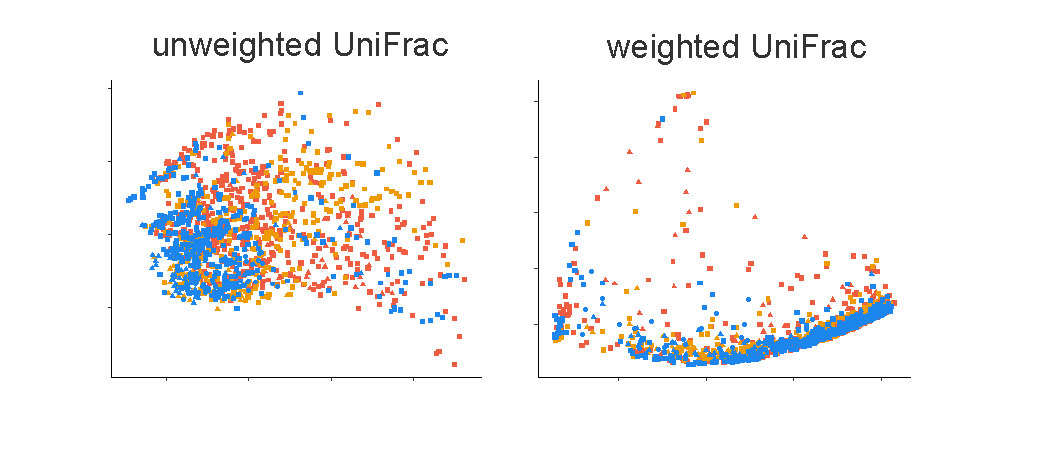

Supplement: Supplementary file 1 [file ijms-23-10868-s001.zip › ijms-1914741-supplementary/Supplementary_FigS1.png]

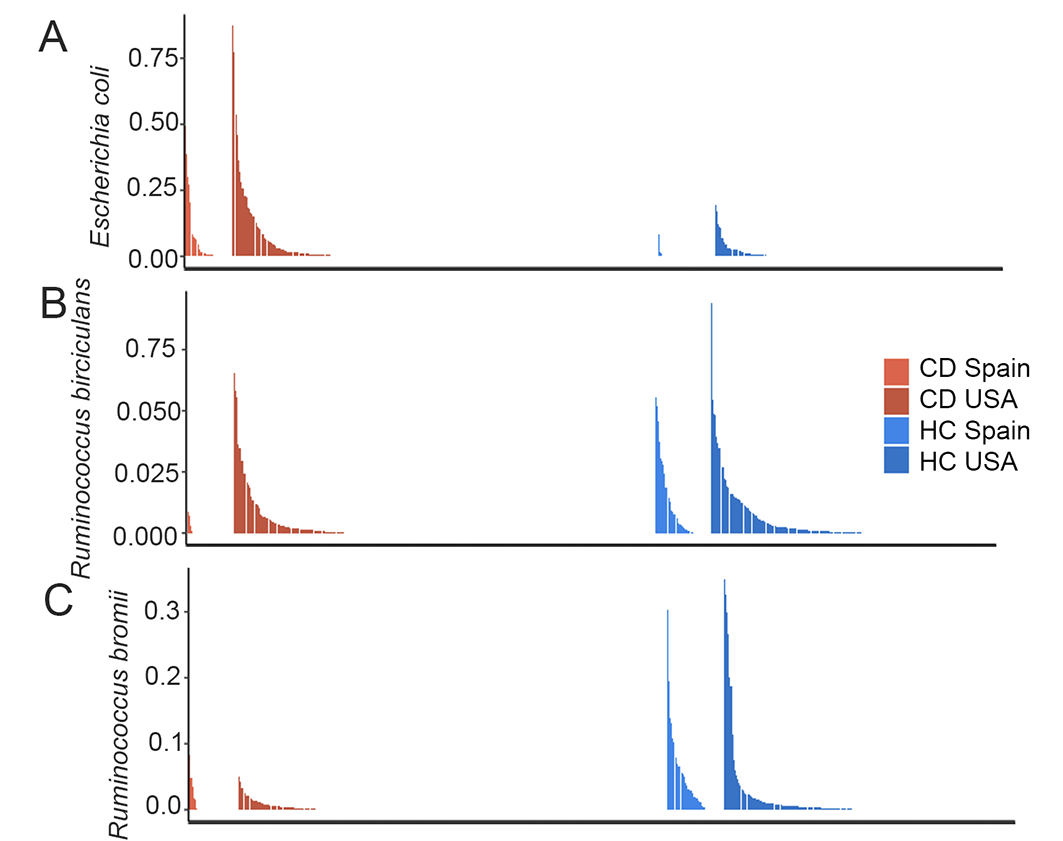

Supplement: Supplementary file 1 [file ijms-23-10868-s001.zip › ijms-1914741-supplementary/Supplementary_FigS2.png]
